# A Versatile and Efficient Plant Protoplast Platform for Genome Editing by Cas9 RNPs

**DOI:** 10.3389/fgeed.2021.719190

**Published:** 2021-12-22

**Authors:** Wenzhi Jiang, Jenifer Bush, Jen Sheen

**Affiliations:** Department of Molecular Biology and Center for Computational and Integrative Biology, Massachusetts General Hospital, and Department of Genetics, Harvard Medical School, Boston, MA, United States

**Keywords:** plant protoplasts, Cas9 RNP, NHEJ, HDR, ssODN donor, prime editor RNP, *Arabidopsis thaliana*, *Nicotiana benthamiana*

## Abstract

The ultimate goal of technology development in genome editing is to enable precisely targeted genomic changes in any cells or organisms. Here we describe protoplast systems for precise and efficient DNA sequence changes with preassembled Cas9 ribonucleoprotein (RNP) complexes in *Arabidopsis thaliana*, *Nicotiana benthamiana, Brassica rapa,* and *Camelina sativa*. Cas9 RNP-mediated gene disruption with dual gRNAs could reach ∼90% indels in Arabidopsis protoplasts. To facilitate facile testing of any Cas9 RNP designs, we developed two GFP reporter genes, which led to sensitive detection of nonhomologous end joining (NHEJ) and homology-directed repair (HDR), with editing efficiency up to 85 and 50%, respectively. When co-transfected with an optimal single-stranded oligodeoxynucleotide (ssODN) donor, precise editing of the *AtALS* gene *via* HDR reached 7% by RNPs. Significantly, precise mutagenesis mediated by preassembled primer editor (PE) RNPs led to 50% GFP reporter gene recovery in protoplasts and up to 4.6% editing frequency for the specific *AtPDS* mutation in the genome. The rapid, versatile and efficient gene editing by CRISPR RNP variants in protoplasts provides a valuable platform for development, evaluation and optimization of new designs and tools in gene and genomic manipulation and is applicable in diverse plant species.

## Introduction

Successful CRISPR-Cas9-gRNA-mediated genome editing in plant cells was first demonstrated in protoplasts isolated from Arabidopsis, tobacco, rice and wheat with varying degrees of efficiency for various targets in different plants (Li et al., 2013; Shan et al., 2013). Despite widespread applications of CRISPR technologies *via* NHEJ-mediated mutagenesis in basic plant research and crop improvement (Zhu et al., 2020), the underlying mechanisms for variable editing efficiency and limited precise editing through HDR remain unresolved challenges for future advances (Chechik et al., 2020; Zhang et al., 2021). Recent CRISPR-Cas9 innovations have significantly expanded the range of genetic variants by PEs (Anzalone et al., 2019; Lin et al., 2020, Jin et al., 2021) and enabled targeted insertion and replacement (Lu et al., 2020). However, the DNA- and transgene-based approaches using *Agrobacterium-* and particle bombardment-mediated plant transformation still face many restrictions (Lin et al., 2020; Lu et al., 2020). The emerging and flexible CRISPR RNPs may offer multiple advantages in DNA-, cloning- and transgene-free genome editing with higher efficiency and precision, minimal off targets, and reduced toxicity. Furthermore, the versatile plant protoplast platform with high transfection efficiency (Yoo et al., 2007; Li et al., 2013, 2014, 2015; Marx, 2016) is highly suitable in supporting the systematic efforts required for developing and testing new CRISPR-Cas9 designs and elucidating relevant molecular mechanisms in broad plant species (Woo et al., 2015; Jin et al., 2021; Zhang et al., 2021).

In this study, we integrated Cas9 RNPs with highly efficient protoplast transfection systems for rapid analyses of gene and genomic editing efficiency. Two reporter genes were developed to facilitate sensitive detection of NHEJ or HDR leading to the recovery of GFP fluorescence in transfected protoplasts within 24 h. We demonstrated that this versatile protoplast platform could be applied to four plant species and support precise and efficient mutagenesis by HDR using relatively short ssODN donors without costly modifications. When combined with a specific ssODN and dual gRNAs, Cas9 RNPs enabled the generation of a precise mutation in the *AtALS gene*, not previously feasible in Arabidopsis protoplasts featuring low editing efficiency with plasmid DNA despite high cell quality and transfection efficiency (Yoo et al., 2007; Li et al., 2013). Importantly, we provided the first evidence that precise mutations free of unintended indels (Jin et al., 2021) could be efficiently generated by PE-mediated editing in the GFP reporter and in the *Arabidopsis* genome using the preassembled nCas9-RT RNP complexes without double-strand break, donor DNA or the additional DNA nick. This versatile and efficient protoplast platform will help enable plant researchers aiming to test novel Cas9-gRNA variants or create new CRISPR designs and tools for facile genome manipulation in model or crop plants.

## Materials and Methods

### Plant Materials and Growth Conditions

Seeds of wild-type *Arabidopsis thaliana* (*A. thaliana*) Col-0, tobacco *Nicotiana benthamiana* (*N*. *benthamiana*)*, Brassica rapa* (*B. rapa*) and *Camelina sativa* (*C. sativa*) were germinated and grown on Jiffy-7 peat pellets (Jiffy group) except that *N*. *benthamiana* seedlings (2 weeks) were transferred to ProMix BK25 soil (Premier Tech Horticulture) in a controlled environment growth room at 12 h light, 23°C/12 h dark, 20°C under low light (75 μE m^−2^ s^−1^) and 60% relative humidity. *N*. *benthamiana* plants were fertilized with 1/4X Hoagland solution once per week, beginning at 3 weeks. Vigorously growing and well-expanded leaves from 24 to 32 days (leaf 1–6) were chosen for mesophyll protoplast isolation. Optimal leaves were systematically tested and selected for high quality mesophyll protoplast isolation with high transfection efficiency. For example, we used leaf 5 & 6 at 30 days for *A. thaliana*, leaf 5 & 6 at 32 days for *N. benthamiana*, leaf 1 & 2 at 24 days for *B. rapa* and leaf 3 & 4 at 29 days for *C. sativa*. Older leaves or plants recovered from stress conditions yielded low quality protoplasts with low transfection efficiency, hence low gene and genome editing efficiency (Yoo et al., 2007; Li et al., 2013, 2014, 2015).

### Plasmid Construction

For constructing the NHEJ *GFP* reporter gene (*NHEJ-GFP*), the *DOF1-GFP* gene (Yanagisawa and Sheen (1998) driven by the *AtUBQ10* promoter with the *NOS* terminator in a pUC19 cloning vector was used to initiate the design. We inserted a 20-bp target sequence plus a PAM sequence (AGG) (GCG​CTT​CAA​GGT​GCA​CAT​GGA​GG) at the 5’ end of the *GFP* gene, which led to an out-of-frame shift in the coding region. A double-strand cleavage within this target region by Cas9-gRNA will generate some indels leading to in-frame shifts in the mutated *GFP* coding region allowing the recovery of the GFP fluorescence after NHEJ-mediated DNA repair in transfected protoplasts (Figure 3A). For the construction of the HDR *GFP* reporter gene (*HDR-GFP*), the chromophore 65TYG67 (Chiu et al., 1996) was mutated to 65TLR67 (nucleotide sequence from ACC TAC GGC to ACC TTA CGC) to inactivate GFP. Only a precise DNA sequence editing by Cas9 or PE RNPs, in which the mutation is converted back to its original chromophore 65TYG67 from 65TLR67 (from ACC TTA CGC to ACC TAC GGC) can recover the GFP fluorescence in protoplasts (Figure 3B). Full coding region sequences of the *NHEJ-GFP* and *HDR-GFP* reporter genes are listed in Supplementary Materials.

To construct *Streptococcus pyogenes* Cas9 protein expression plasmid, the codon optimized *Cas9* sequence for *E. coli* expression with a monopartite nuclear localization signal (NLS) (PKKKRKV) at the C terminus with 6XHIS tag in the pET15 expression vector (D’Astolfo et al., 2015) was fused to a bipartite NLS (KRPAATKKAGQAKKKK) at the N terminus to generate *bNLSCas9*. For constructing the plasmid to express PE in *E. coli*, we mutated *bNLSCas9* to a nickase (H840A) and fused the engineered M-MLV reverse transcriptase (RT) and the linker from PE2 (Anzalone et al., 2019) to the C terminus to generate *nCas9-RT*. The coding sequences of *bNLSCas9* and *nCas9-RT* plasmids are listed in Supplementary Materials.

### Protein Expression and Purification

The *bNLSCas9* or *nCas9-RT* plasmid was transformed into *E. coli* BL21 (DE3) cells and single colony was picked and grown overnight at 37°C in 5 ml Terrific Broth medium. This 5 ml culture was further propagated in 2 L flask containing 500 ml of LB medium and grown at 220 rpm in a 37°C shaker. When cell density reached OD_600_ = 0.5 after 3–4 h, the culture was transferred into 180 rpm and 30°C for another 30 min before adding 1 mM IPTG to induce protein expression. The culture was kept at 180 rpm and 18°C for 48 h. Cells were then harvested and the pellet was re-suspended in 10 ml buffer, 50 mM NaH_2_PO_4_ pH 8, 1 M NaCl, 10 mM 2-mercaptoethanol and 2.5 mM MgCl_2_ and protease inhibitors (cOmplete, EDTA-free, Roche 05056489001). The 10 ml sample was then sonicated for 6 min (10″ on and 10″ off, 30% amplitude) on ice using a Q700 sonicator (Qsonica). After sonication, the sample was spun down for 30 min at 20,000× g, and 4°C. The supernatant was collected and applied onto a 35 ml column filled with 1 ml High Affinity Nickel-Charged resin (GenScript L00223). The sample was washed twice with the re-suspension buffer. Proteins were eluted from column by using a re-suspension buffer containing 200–250 mM imidazole. Elutes were pooled together and concentrated to ∼5 μg/μl by Amicon Ultra-15 with 10 KDa cutoff. The buffer for concentrated protein was exchanged by dialysis using 3500 MWCO cassette (Slide-A-Lyzer 3.5K Dialysis Cassettes, Thermo Scientific 66,330) at 4°C for 16 h. The storage buffer contains 20 mM HEPES pH 7.5, 150 mM KCI, 2 mM DTT and 10% glycerol. Purity of proteins was examined by protein gel electrophoresis and staining before storage at −80°C.

### Design of gRNA and pegRNA and *in Vitro* Synthesis

Design of gRNAs and pegRNAs followed the methods described previously (Li et al., 2013, 2015; Anzalone et al., 2019; Jin et al., 2021). For *in vitro* synthesis of the gRNA or pegRNA targeting the GFP reporter gene or different sites in the genome, DNA from a plasmid containing the sequence of full-length scaffold gRNA was used as the common template, and a full-length DNA template was PCR amplified using a pair of designed forward and reverse primers (see Supplementary Tables S1, S2, S4). *In vitro* gRNA synthesis was undertaken using RNA synthesis kit HiScribe™ T7 Quick High Yield RNA Synthesis (NEB) and purified by RNeasy Mini Kit (Qiagen).

### Analysis of Cas9 RNP Activity *in Vitro*

Cas9 RNP activities were determined *in vitro* using 20 µl reactions containing 2 µl NEB CutSmart Buffer, 1 µg Cas9 protein, 1 µg gRNA for preassembly at 23°C for 10 min. Linearized plasmid DNA (0.2 µg) with the designed *AtBON*1 (At5G61900) target site (Li et al., 2015) was then added and incubated at 37°C for 30 min before visualization by running a 2% agarose gel and staining with ethidium bromide for UV imaging.

### Preassembly of Cas9 RNPs

For preassembly of Cas9 RNPs, 2 µl NEB 10X Buffer 3, 20 µg gRNA or pegRNA and 40 µg of bNLSCas9 protein or nCas9-RT protein was mixed to a final volume of 20 µl. The preassembly of Cas9 RNPs was performed at 30°C for 30 min without visible precipitation in the mix.

### Protoplast Isolation and Transfection

Methods for mesophyll protoplast isolation from leaves and transfection with optimal concentration of plasmid DNA to express Cas9 and gRNA were described previously (Yoo et al., 2007; Li et al., 2013; Li et al., 2015) with minor modifications. The analyses of GFP reporter editing and genome editing were conducted after dark incubation of transfected protoplasts at 24 h instead of 36 h based on extensive comparison of prior and current results (Li et al., 2013; Li et al., 2015). Longer incubation of transfected protoplasts beyond 24 h did not significantly increase the editing efficiency with both plasmid DNA or RNPs. Specific plant age and leaf number for four plant species are described in plant materials and growth conditions and in the Results. Plasmid DNA or ssODN was added to protoplasts and mixed well before adding Cas RNPs for PEG-calcium-mediated transfection. In the case when preassembled Cas9 RNPs were used, protoplasts were incubated at 30°C for 20 min after the PEG-calcium treatment. Protoplasts were washed twice using W5 buffer before incubation in WI buffer. Typically, 100 μl cells at 3 × 10^5^ ml^−1^, 15 µg GFP reporter plasmid DNA, 30 µg ssODN, 20 µg gRNA or pegRNA, and 40 µg Cas9 protein were used in each transfection.

### Editing Efficiency Evaluation by GFP Imaging in Protoplasts

Evaluation of editing efficiency for NHEJ or HDR mediated by Cas9-gRNA expressed from transfected plasmid DNA or Cas9-RNPs was quantified by counting the percentage recovery of GFP in plant protoplasts. *UBQ10-NLS-tdTomato* (Emonet et al., 2021) was co-transfected and served as a control to mark the transfected protoplasts and determine the transfection efficiency. Percentage recovery of the GFP reporter gene in protoplasts was quantified by fluorescence microscopy as a ratio of cells with visible GFP signal to 100 total transfected cells expressing nuclear tdTomato.

### Editing Efficiency Evaluation in the Genome

Protoplasts were harvested 24 h after transfection by pelleting protoplasts, resuspending the cells in 20 μl TE buffer and boiling at 95°C for 10 min. PCR amplification of target regions of 200–240 bp spanning individual Cas9-gRNA target sequences was performed using Phusion high-fidelity DNA polymerase (NEB) with 2 μl protoplast lysates (see Supplementary Table S3, S5 and S6 for primers used for PCR). PCR amplicons (200–240 bp) were analyzed by next-generation sequencing (NGS) paired-end reads yielding typically up to 80,000 reads for each sample. The percentage of WT and each variant sequence was determined in order to estimate the editing efficiency. The amplicon NGS data were submitted to NCBI (PRJNA781623) and presented in Supplementary Figure S5.

For the analysis of off-target frequency, off-target sites were predicted using Cas-OFFinder tool (www.rgenome.net). Primers for PCR amplifying target regions are listed in Supplementary Table S6. PCR amplicons were analyzed by NGS described as above.

## Results

### Optimal Leaf Selection for Protoplast Isolation and Transfection

**FIGURE 1:**
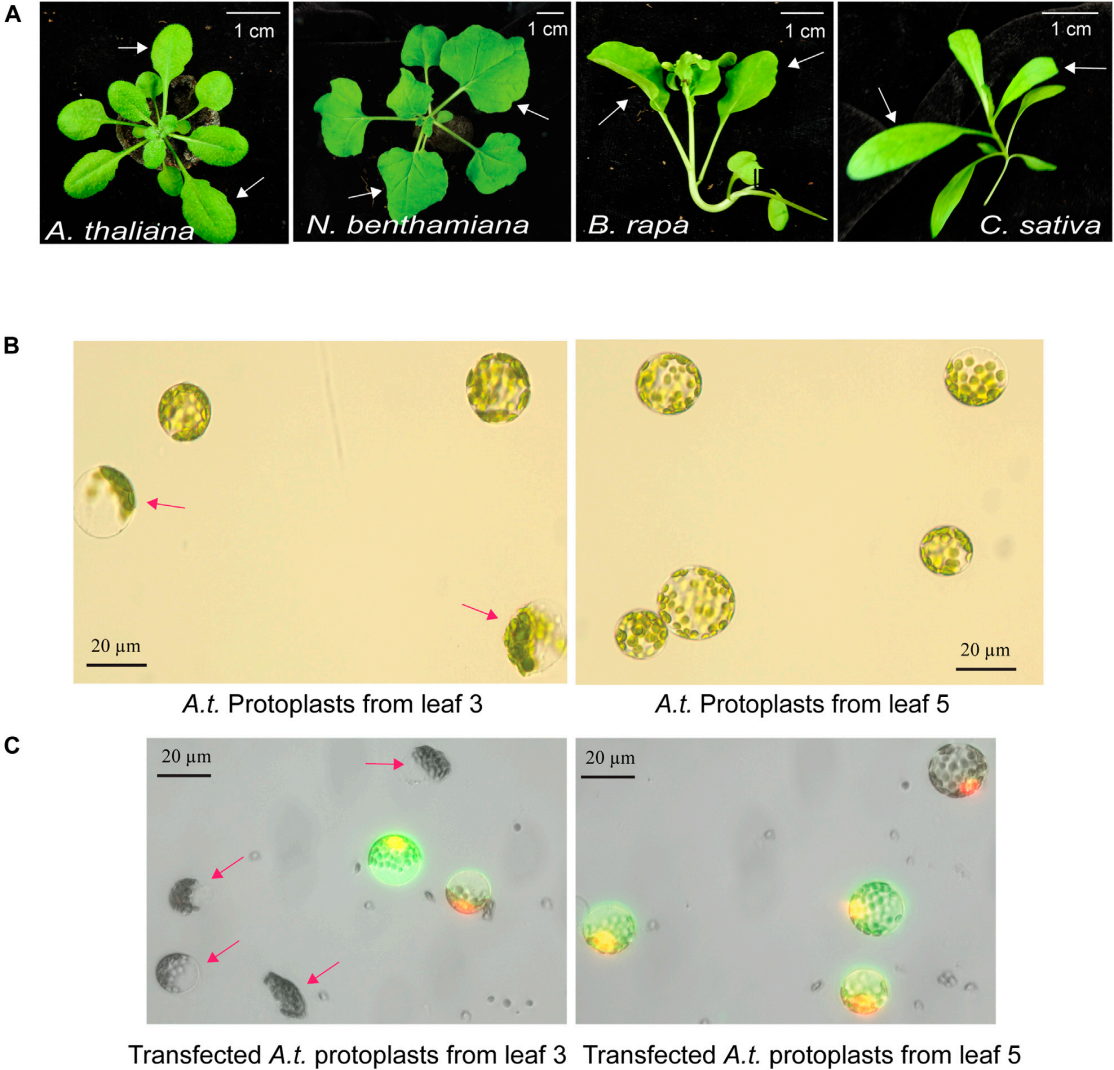
Optimal leaf selection for the mesophyll protoplast platform. **(A)** Representative leaves for high quality protoplast isolation with high transfection efficiency. *A. thaliana* (leaf 5 & 6 at 30 days), *N. benthamiana* (leaf 5 & 6 at 32 days), *B. rapa* (leaf 1 & 2 at 24 days) and *C. sativa* (leaf 3 & 4 at 29 days). **(B)** Integrity of protoplasts isolated from older leaf 3 and younger leaf 5 in the same *A. thaliana* plants. **(C)** Transfection efficiency and integrity of protoplasts isolated from older leaf 3 and younger leaf 5. Transfected protoplasts show GFP fluorescence. Red arrows indicate low quality protoplasts from older leaf 3. Reproducible results were obtained from at least two independent biological experiments.

The quality of mesophyll protoplasts isolated from plant leaves is critical for highly efficient transfection of DNA, RNA and proteins, and is essential to the level of gene expression and gene editing efficiency by Cas9-gRNA (Yoo et al., 2007; Li et al., 2013). Environmental factors, such as light, photoperiod, temperature, water, nutrients, soil, and relative humidity, are key to support healthy plant growth and determine the developmental timing and stage of each leaf. The physiological conditions and the age of the leaves strongly influence the quality of isolated protoplasts. Four different plant species, *A. thaliana, N. benthamiana*, *B. rapa* and *C. sativa,* were used in this study to illustrate the selection of optimal leaves at a specific age grown in a defined environmental condition (Figure 1A). For example, young and actively expanding leaf 5 and leaf 6, but not older leaf 3, from the same 30-day-old Arabidopsis plants grown in a defined environment (details in MATERIALS AND METHODS) provided active protoplasts for gene expression based on numerous studies (Yoo et al., 2007; Li et al., 2013). As shown in Figure 1B, many protoplasts isolated from older leaf 3 exhibited compromised integrity but not protoplasts isolated from younger leaf 5 (leaf 3 integrity of 60.1%, ±7.1 vs. leaf 5 integrity of 93.0%, ±2.6; *n* = 3, *p <* 0.01). Transfection efficiency of protoplasts isolated from older leaf 3 was also much lower based on the expression of the GFP reporter (leaf 3 transfection efficiency of 42.5%, ±1.26 vs. leaf 5 transfection efficiency of 90.0%, ±0.88; *n* = 3, *p <* 0.001) (Figure 1C). It was clear that comparing leaves at different developmental ages in the same plants for protoplast integrity and transfection efficiency identified the optimal leaves for the most robust mesophyll protoplasts suitable for transient expression experiments. Using a similar growth condition and protoplast tests, leaf 5 & 6 at 32 days, leaf 1 & 2 at 24 days, and leaf 3 & 4 at 29 days were optimal for protoplast isolation in *N. benthamiana, B. rapa* and *C. sativa*, respectively (Figure 1A).

### Validation of Cas9-RNP Activity *in Vitro* and *in Vivo*

**FIGURE 2:**
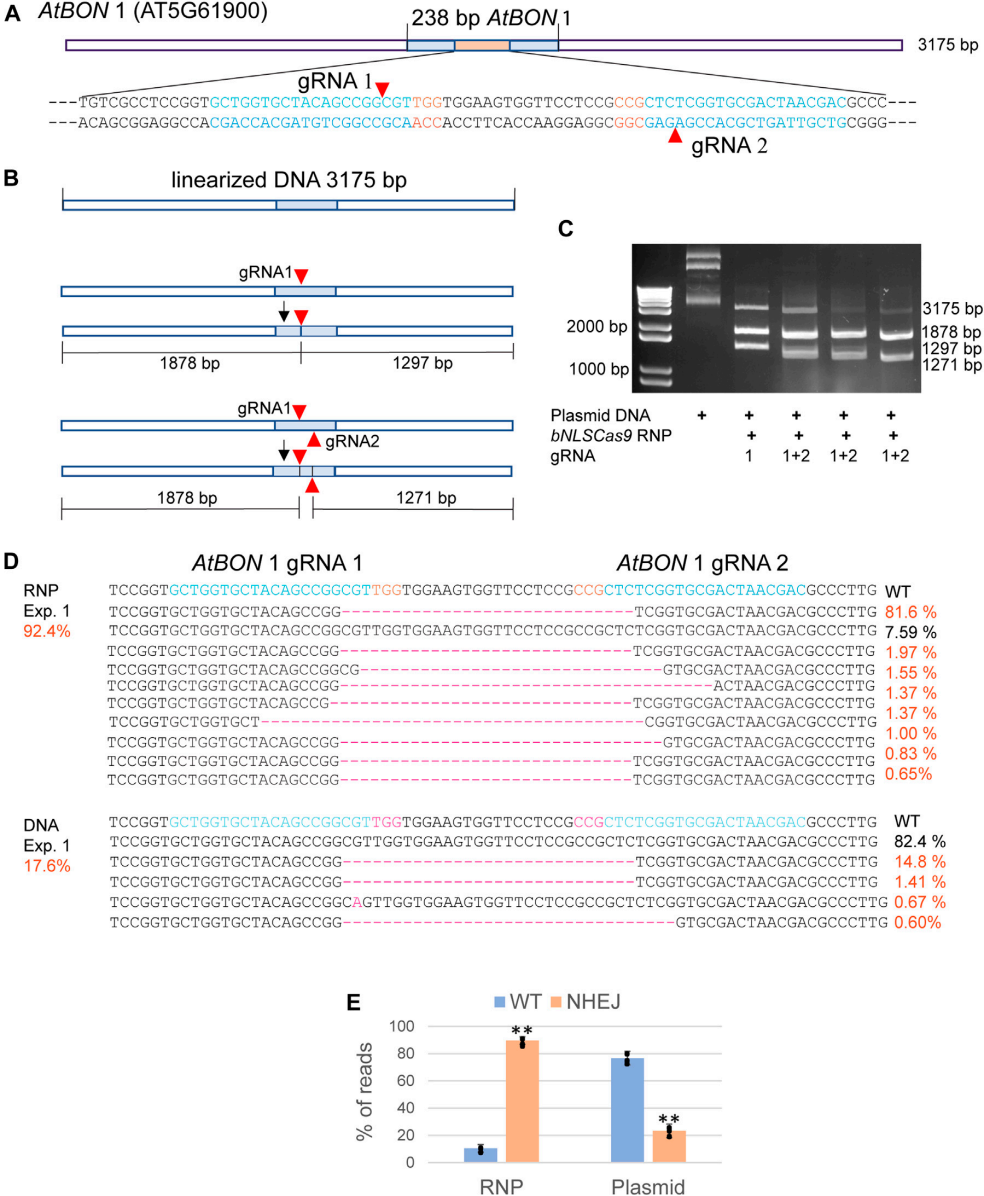
Analyses of bNLSCas9 RNP *in vitro* and in protoplasts. **(A)** Schematic presentation of the linearized plasmid carrying the *AtBON* 1 editing target region. The sequence of 80 bp with two gRNAs is shown with the PAM sequence in red. **(B)** Schematic presentation of *in vitro AtBON* 1 editing. Red arrowheads indicate the double-strand break sites. **(C)** bNLSCas9 RNP-mediated double-strand break at *AtBON* 1 *in vitro* and predicted plasmid DNA fragments are shown. **(D)** Comparison of *AtBON* 1 editing *via* NHEJ by bNLSCas9 RNP or DNA transfection in Arabidopsis protoplasts. Indel (red) % of one representative biological experiment is shown by NGS analyses of the amplicons generated by PCR using genomic DNA isolated from transfected protoplasts. **(E)** The *AtBON1* editing efficiency from three biological repeats (**p < 0.01; error bars, s.d., *n* = 3).

Abundant bNLSCas9 protein was successfully overexpressed in *E. coli*, purified by one-step high affinity Nickel-charged resin (D’Astolfo et al., 2015), and tested for *in vitro* and *in vivo* activities using the previously validated dual gRNA target sites in the *AtBON*1 gene (AT5G61900) (Figure 2A) (Li et al., 2015). For the *in vitro* assay, the 3,175 bp plasmid DNA carrying the 238-bp insert from *AtBON*1 was linearized and subjected to double-strand cleavage by preassembled Cas9-RNPs with one or two gRNAs (Figure 2B). The results showed that Cas9-RNPs generated the predicted 1878-bp and 1297-bp DNA fragments with gRNA1, which was coupled with the significant reduction of the 3175-bp linear plasmid DNA. Two gRNAs supported even higher cleavage efficiency (Figure 2C). In *Arabidopsis* protoplasts transfected with Cas9-RNPs preassembled with dual gRNAs targeting the *ATBON*1 gene, NGS analyses revealed a remarkably high editing efficiency up to 92.4, 89.5 and 86.7% in three biologically independent experiments. There was 3.8-fold increase when the editing efficiency was compared between Cas9-RNPs and Cas9-gRNA expression by conventional plasmid DNA transfection (Figure 2D **,** Supplementary Figure S1), consistent with the previously reported 20% editing efficiency with dual gRNAs in *AtBON*1 by DNA transfection in Arabidopsis protoplasts (Li et al., 2015).

We also tested and compared the editing efficiency with plasmid DNA or RNP using dual gRNAs targeting *AtPDS* (AT4G14210) and *AtFAD2* (AT3G12120, referred to as *AtFAD* below in the text) in *Arabidopsis* protoplasts. Three independent biological experiments with triplicate samples for each independent experiment were conducted to determine the editing efficiency mediated by NHEJ. NGS analyses revealed a significantly higher RNP editing efficiency at 61.3% ± 5.1 and 62.6% ± 1.7 in *AtPDS* and *AtFAD,* respectively, than those of DNA at 5.9% ± 3.4 and 12.1% ± 1.9, respectively (Supplementary Figures S2, S3). The results suggested that the preassembled RNPs could be more efficient in genome editing of three independent Arabidopsis genes using protoplast assays.

To investigate whether Cas9 RNP could induce undesired edits at the genome level in *Arabidopsis* protoplasts, Cas-OFFinder was used to predict off-target sites for the gRNAs targeting *AtBON1*. We did not find any off-targets by PCR and sequencing analyses of the predicted off-target sites for *AtBON1* gRNAs (data not shown).

### Cas9-RNP Activity in Editing *NHEJ-GFP* and *HDR-GFP* Reporter Genes

**FIGURE 3:**
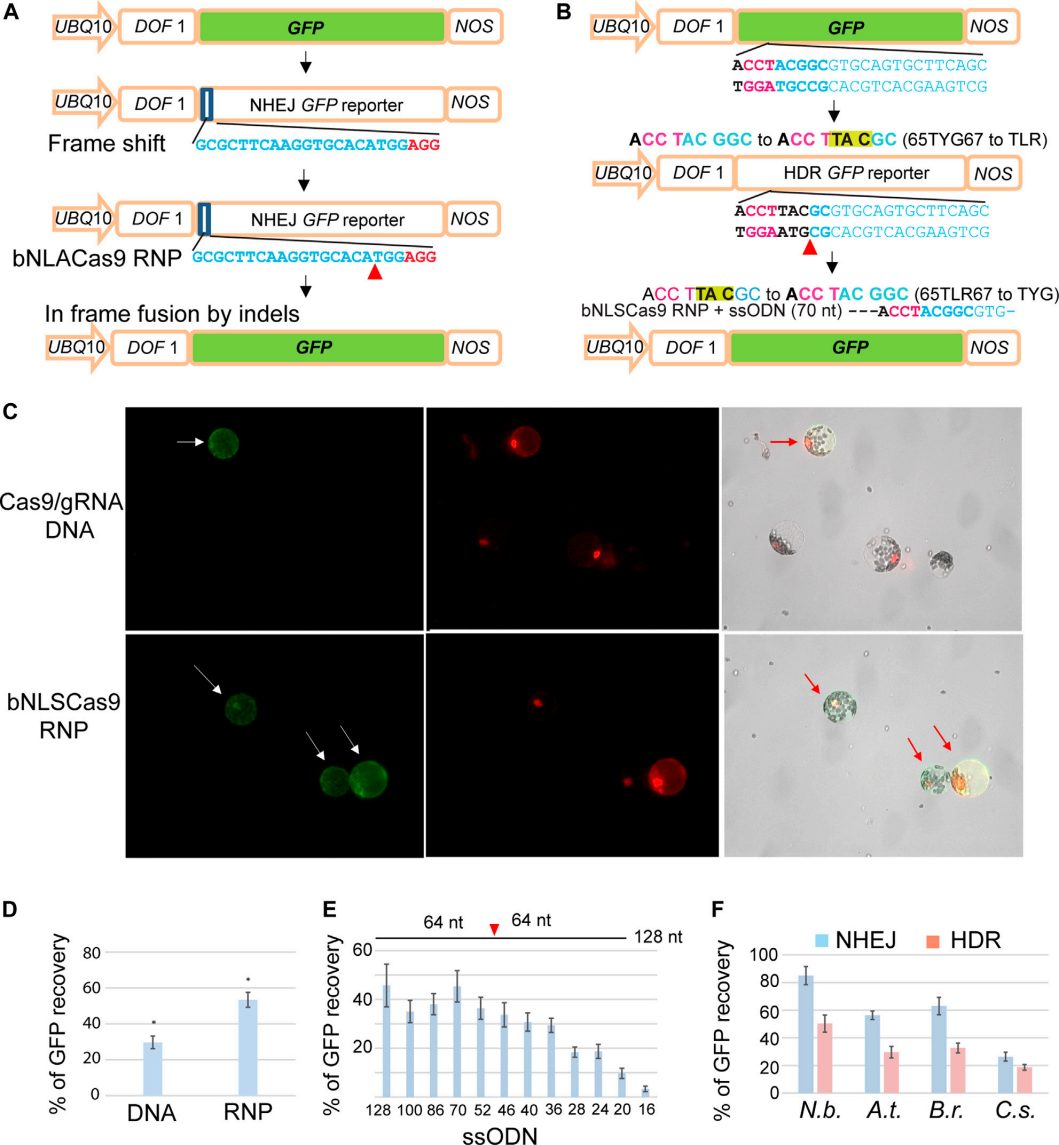
Facile GFP reporter genes for sensitive NHEJ and HDR detection in protoplasts. **(A)** The design and analysis of the NHEJ GFP reporter gene. **(B)** The design and analysis of the HDR GFP reporter gene. **(C)** Comparison of GFP recovery efficiency by NHEJ *via* DNA transfection or bNLSCas9 RNP in Arabidopsis protoplasts. Protoplasts were co-transfected with *UBQ10-tdTomato* for normalization. **(D)** Quantitative analysis of GFP recovery by DNA- or RNP-mediated gene editing. **(E)** The length of ssODN donor determines HDR editing efficiency by bNLSCas9 RNP in tobacco protoplasts. **(F)** Efficient editing of GFP reporter genes in four protoplast systems. Blue bar, NHEJ. Red bar, HDR. SD is shown (n = 3), *p* < 0.001. The sequences of gRNAs are shown with the PAM sequence in red. Red arrowhead indicates the double-strand break site in the GFP reporter gene. *UBQ10*, the *A.t. UBIQUITIN10* gene promoter. *DOF1*, the nuclear localization signal. *NOS*, the *NOS* gene terminator. *At*, *A. thaliana, N.b., N. benthamiana*, *B. r., B. rapa*, and *C.s., C. sativa*.

Highly efficient and rapid transient expression analyses in mesophyll protoplasts provide an excellent platform to rapidly evaluate gene editing efficiency of emerging Cas9-gRNA variants (Yoo et al., 2007; Li et al., 2013, 2014; Lin et al., 2020; Lin et al., 2021; Zhang et al., 2021). To further simplify and lower the cost for sensitive detection of gene editing mediated *via* NHEJ or HDR by Cas9 complexes, we developed two GFP reporter genes, *NHEJ-GFP* (Figure 3A) and *HDR-GFP* (Figure 3B)*,* that can be easily applied in a broad range of established or new protoplast systems for diverse plant species. For example, gene editing by Cas9-gRNA variants could generate a double-strand break and in-frame shift *via* NHEJ or the precise 65TLR67 to 65TYG67 mutation by HDR with a ssODN donor, leading to the recovery of GFP fluorescence. *UBQ10-NLS-tdTomato* was co-transfected and served as a control to determine the transfection efficiency (Figure 3C). Quantitative analyses determined by the ratio of GFP/*tdTomato* in Arabidopsis protoplasts demonstrated a significantly higher gene editing efficiency by Cas9 RNP (53.3%) than by DNA transfection to express Cas9-gRNA (29.6%) (Figure 3D). To test GFP reporter recovery by NHEJ-mediated repair in different plant species, protoplasts isolated from *N. benthamiana, A. thaliana, B. rapa* and *C. sativa* (Figure 1A) were co-transfected with Cas9 RNP and *NHJE-GFP* (Figure 3A). The recovered GFP signal mediated by Cas9-RNP was detected in the nuclei of the transfected protoplasts of *N. benthamiana, A. thaliana, B. rapa and C. sativa* at the editing efficiency of 85, 56, 63 and 26%, respectively (Figure 3F).

To detect precise gene editing *via* HDR, we co-transfected ssODN complementary to the non-target strand as the DNA donor and *HDR-GFP* (Figure 3B) with preassembled Cas9 RNP. We first determined the efficacy of the length of individual ssODN for HDR-mediated GFP recovery in *N. benthamiana* protoplasts, which appeared to exhibit the highest efficiency in genome editing from previous studies (Li et al., 2013). Longer ssODN up to 70 nt as the donor template for HDR promoted the repair of *GFP* at the highest efficiency of 45%. However, even a 36-nt short symmetric ssODN complementary to the non-target strand produced sufficiently high HDR-mediated gene editing (Figure 3E) (Richardson et al., 2016). The same 70 nt ssODN template was further tested for the efficiency of HDR-directed mutant GFP recovery in the protoplasts isolated from *N. benthamiana, A. thaliana*, *B. rapa* and *C. sativa* and transfected with Cas9 RNP. Recovered GFP signal was detected in 50, 30, 33 and 19% of the transfected protoplasts of *N. benthamiana, A. thaliana, B. rapa and C. sativa*, respectively (Figure 3F). The findings suggested that the sensitive GFP reporter genes will be valuable and facile tools for the development of CRISPR technologies and for advancing our understanding of the key molecular mechanisms in plants.

### Cas9-RNP-ssODN Mediates HDR in the Genome

**FIGURE 4:**
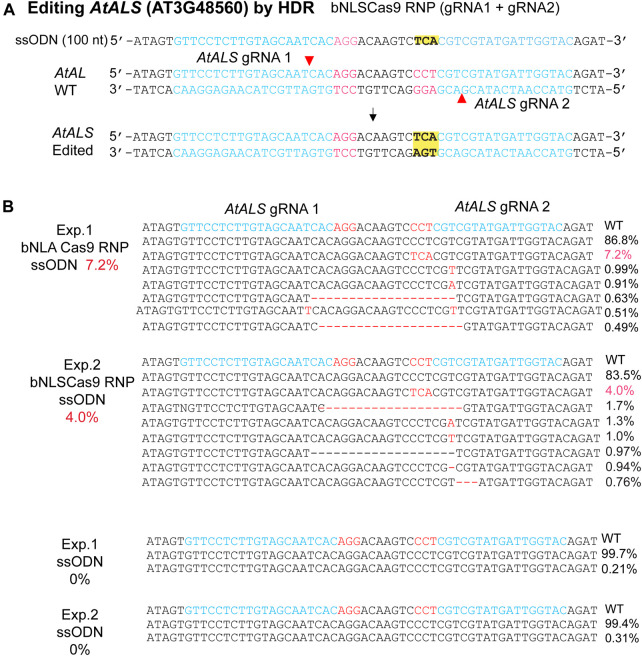
Precise bNLSCas9 RNP-mediated mutagenesis. **(A)** The experimental design for precise mutagenesis *via* HDR by bNLSCas9 RNP and ssODN. **(B)** Specific *AtALS* mutation generated by bNLSCas9 RNP and ssODN in Arabidopsis protoplasts. Protoplasts were transfected with ssODN without RNP served as the background control. Editing efficiency (red %) was shown by NGS analyses of the amplicons generated by PCR using genomic DNA isolated from transfected protoplasts.

The efficacy of Cas9-RNP/ssODN for HDR-mediated precise gene editing was further tested in the genome. *AtALS* (AT3G48560) is an ideal gene for the HDR efficiency testing in plants because change of only one amino acid at the defined site confers herbicide resistance (Haughn, et al., 1988). To enhance the efficiency, we generated two neighboring gRNAs on the opposite strand (Li et al., 2015) and synthesized the ssODN to mediate the specific Pro/CCT to Ser/TCA mutation with or without Cas9 RNPs (Figure 4A). The results showed 7.2 and 4.0% HDR-mediated genomic editing efficiency at the target site in two independent biological experiments, whereas no HDR was detected when Cas9 RNPs was absent (Figure 4B). The findings paved the way for generating precisely targeted genomic changes in plants regenerated from engineered protoplasts (Lin et al., 2018; Hsu et al., 2021; Zhang et al., 2021).

### PE RNPs Mediate Precise Prime Editing

**FIGURE 5:**
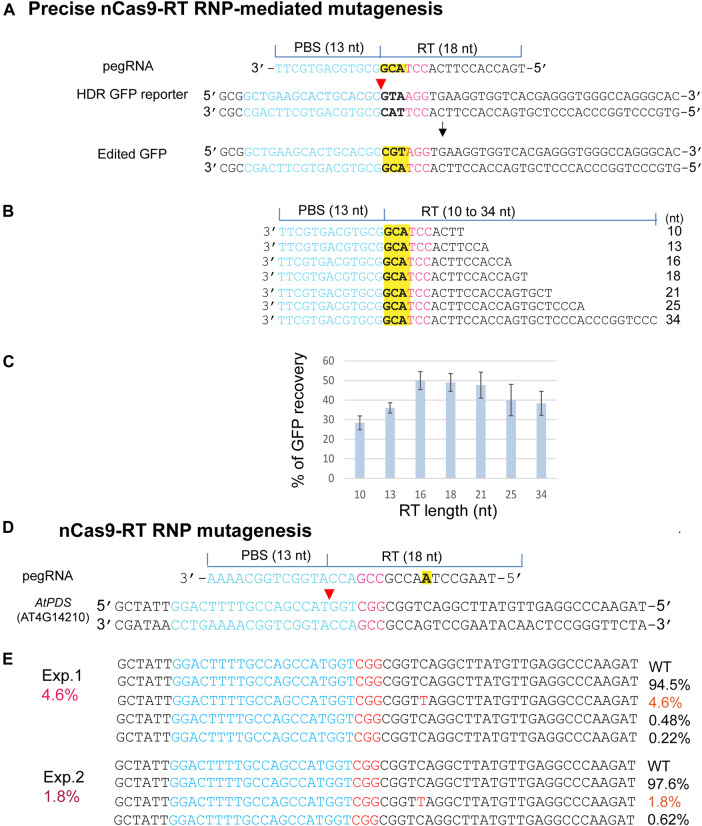
Precise PE RNP-mediated mutagenesis. **(A)** The experimental design for precise mutagenesis by PE RNP. **(B)** Analyses of pegRNA variants. **(C)** RT template length affects PE RNP-mediated editing in the HDR GFP reporter gene. **(D)** The experimental design to generate a precise *AtPDS* mutation by PE RNP-mediated editing in the genome. **(E)** A specific *AtPDS* mutation generated by PE RNP in Arabidopsis protoplasts. Editing efficiency (red %) was shown by NGS analyses of the amplicons generated by PCR using genomic DNA isolated from transfected protoplasts.

The emerging PEs for versatile genome editing without double-strand breaks and donor DNA were derived from a protein fusion with Cas9 nickase (H840A) and the mammalian viral M-MLV RTase (nCas9-RT). PE3b was most active in human tumor culture cells (HEK293T), but its requirement for a second gRNA-mediated nick appeared to enhance unintended indels in mouse embryos (Anzalone et al., 2019; Aida et al., 2020; Lin et al., 2021). We expressed an nCas9-RT derived from PE2 and bNLSCas9 in *E. coli* for the PE-RNP-mediated gene editing assays using *HDR-GFP* in *N. benthamiana* protoplasts (Figure 5A). For optimizing PE-RNP-mediated HDR efficiency, primer binding site (PBS), which was kept at 13 nt, and the RT template (RTT) were tested from 10 to 34 nt in pegRNAs (Figure 5B). GFP recovery in transfected *N. benthamiana* protoplasts showed that the ideal RTT length was between 16–18 nt to reach 50% efficiency in precise gene editing (Figure 5C). We also tested the efficacy of RT RNPs with a specific pegRNA in precisely editing the *AtPDS* gene in the genome. The change from C to T introduced a stop codon TAG in the *AtPDS* coding region. NGS analyses of the amplicons detected up to 4.6% precise base replacement caused by PE RNPs (Figure 5D). The off-target frequency caused by PE RNP was very low at 0–0.02% in Arabidopsis protoplasts (Supplementary Figure S4). This finding is consistent with the result reported for PE-mediated genome editing in rice protoplasts with off-target frequency at 0.00–0.23% (Jin et al., 2021). The rapid tests with remarkably high efficiency and RTT length optimization based on the *HDR-GFP* reporter gene provided insightful information to support the experimental design for successful exploration of new PE RNPs not previously validated at the genomic level in any organisms.

## Discussion

By combining multiple advantages of the superior flexibility and high efficacy of Cas9 RNPs with rapid and versatile transient assays, our studies demonstrated that the plant protoplast platform is simple, robust, economical and generally applicable in multiple plant species to support the rapidly evolving advances and innovations of CRISPR technologies. Easy guidelines and universal principles are provided for the selection of optimal leaves as the sources of high-quality protoplasts with desirable transfection efficiency in a wide range of model and crop plants. Unlike the commonly used mammalian cell lines, convenient seed storage, germination and easy plant care provide the most reliable and abundant primary plant materials without cell culture maintenance. Moreover, cell cycle could be manipulated in mesophyll protoplasts (Xiong et al., 2013) to potentially enhance precision genome editing *via* HDR with short ssODN by Cas9 RNPs or PE RNPs without double-strand break and donor DNA. The protoplast systems integrating Cas9 RNPs or PE RNPs with highly efficient protoplast transfection using sensitive and versatile GFP reporter genes enable rapid screens and analyses of NHEJ-, HDR- or RT-mediated gene editing efficiency. The platform is most suitable for comprehensive testing and systematic evaluation of various Cas9-gRNA tools and new designs for multiplex and precision genome editing, as well as for further research to elucidate the underlying molecular mechanisms.

We fully recognize that the production and purification of high-quality Cas proteins and synthetic gRNAs and pegRNAs to achieve high genome editing efficiency in protoplasts is still a significant challenge for many plant labs (Zhang et al., 2021). Our intention is to share our accumulated experiences with promising results using RNPs in four different eudicot protoplast systems. We hope that the new positive findings may encourage others to try RNPs in their favorite plant systems to potentially obtain higher genome editing efficiency not previously possible using plasmid DNA. For successful genome editing experiments in protoplasts, paying attention to every detail (MATERIALS and MATHODS; Yoo et al., 2007; Li et al., 2013, 2015) from plant growth condition, leaf age and selection, protoplast isolation and transfection, to RNP production and assembly, gRNA design and quality, and NLS-tag are all crucial in the development of highly efficient protoplast systems with different plant species.

Current applications of CRISPR technologies in basic plant and agricultural research are mostly limited to the error-prone NHEJ-generated mutants using *Agrobacterium*- or particle bombardment-mediated plant transformation, often yielding unpredictable outcomes or traits. The protoplast platform will facilitate the development of precision genome editing at high efficiency using Cas9 RNPs or PE RNPs without transgenes or selection markers. The platform is a prerequisite for ongoing efforts to advance genome edited protoplasts to regenerated plants without genome instability (Woo et al., 2015; Lin et al., 2018; Hsu et al., 2021; Zhang et al., 2021). Although plant regeneration can be very difficult in some plants, with the development of protoplast methodology for plant regeneration of various species, the protoplast platform has broad application prospects. Future advances in the development of new delivery strategies, such as the promising and comprehensive nanoparticle technologies (Kumar et al., 2020; Lv et al., 2020) for introducing Cas9 RNPs or PE RNPs directly into pollen, zygotes, embryos and regenerating meristems (Toda et al., 2019; Zhang et al., 2021), will offer powerful alternatives for versatile and precise genetic engineering in diverse plants.

## Data Availability

The original contributions presented in the study are included in the article/Supplementary Materials, further inquiries can be directed to the corresponding author.
