# Supplementary material for: A Versatile and Efficient Plant Protoplast Platform for Genome Editing by Cas9 RNPs
Source: Front Genome Ed. 2021 Dec 22;3:719190. doi: 10.3389/fgeed.2021.719190 (PMC8729822; doi:10.3389/fgeed.2021.719190)
Supplement: Supplementary file 1 [file DataSheet2.pdf]

SUPPLEMENTAL MATERIAL for "A Versatile and Efficient Plant Protoplast Platform for Genome Editing by Cas9 RNPs"

*AtBON*1 gRNA1 — *AtBON*1 gRNA2

RNP Exp. 2 — 89.5 %

Sequence	
TCCGGTGCTGGTGCTACAGCCGGCGTTGGTGGAAGTGGTTCCTCCGCCGCTCTCGGTGCGACTAACGACGCCCTTG	WT
TCCGGTGCTGGTGCTACAGCCGG-----------------------------TCGGTGCGACTAACGACGCCCTTG	87.5%
TCCGGTGCTGGTGCTACAGCCGGCGTTGGTGGAAGTGGTTCCTCCGCCGCTCTCGGTGCGACTAACGACGCCCTTG	10.3 %
TCCGGTGCTGGTGCTACAGCCGGCG------------------------------GTGCGACTAACGACGCCCTTG	2.0 %

*AtBON*1 gRNA1 — *AtBON*1 gRNA2

RNP Exp. 3 — 86.7 %

Sequence	
TCCGGTGCTGGTGCTACAGCCGGCGTTGGTGGAAGTGGTTCCTCCGCCGCTCTCGGTGCGACTAACGACGCCCTTG	WT
TCCGGTGCTGGTGCTACAGCCGG-----------------------------TCGGTGCGACTAACGACGCCCTTG	70.4 %
TCCGGTGCTGGTGCTACAGCCGGCGTTGGTGGAAGTGGTTCCTCCGCCGCTCTCGGTGCGACTAACGACGCCCTTG	13.3 %
TCCGGTGCTGGTGCTACAGCCG------------------------------TCGGTGCGACTAACGACGCCCTTG	3.5 %
TCCGGTGCTGGTGCTACAGCCGGCG------------------------------GTGCGACTAACGACGCCCTTG	3.2 %
TCCGGTGCTGGTGCTACAGCCGG-----------------------------TCGGTGCGACTAACGACGCCCTTG	1.8 %
TCCGGTGCTGGTGCTACAGCC-------------------------------TCGGTGCGACTAACGACGCCCTTG	1.4 %
TCCGGTGCTGGTGCTACAGCCGG--------------------------------GTGCGACTAACGACGCCCTTG	1.2 %
TCCGGTGCTGGTGCTACAGCCGG-----------------------------TCGGTGCGACTAACGACGCCCTTG	1.0 %

*AtBON*1 gRNA1 — *AtBON*1 gRNA2

DNA Exp. 2 — 25.9%

Sequence	
TCCGGTGCTGGTGCTACAGCCGGCGTTGGTGGAAGTGGTTCCTCCGCCGCTCTCGGTGCGACTAACGACGCCCTTG	WT
TCCGGTGCTGGTGCTACAGCCGGCGTTGGTGGAAGTGGTTCCTCCGCCGCTCTCGGTGCGACTAACGACGCCCTTG	73.7 %
TCCGGTGCTGGTGCTACAGCCGG-----------------------------TCGGTGCGACTAACGACGCCCTTG	23.6 %
TCCGGTGCTGGTGCTACAGCCGGCG------------------------------GTGCGACTAACGACGCCCTTG	1.0 %
TCCGGTGCTGGTGCTACAGCCG------------------------------TCGGTGCGACTAACGACGCCCTTG	0.67 %
TCCGGTGCTGGTGCTACAGCCGG----------------------------TCTGGTGCGACTAACGACGCCCTTG	0.45 %
TCCGGTGCTGGTGCTACAGCCGGCGTTGGTGGAAGTGGTTCCTCCGCCGCTCTCGGTGCGACTAACGACGCCCTTG	0.42 %

*AtBON*1 gRNA1 — *AtBON*1 gRNA2

DNA Exp. 3 — 26.5%

Sequence	
TCCGGTGCTGGTGCTACAGCCGGCGTTGGTGGAAGTGGTTCCTCCGCCGCTCTCGGTGCGACTAACGACGCCCTTG	WT
TCCGGTGCTGGTGCTACAGCCGGCGTTGGTGGAAGTGGTTCCTCCGCCGCTCTCGGTGCGACTAACGACGCCCTTG	73.5 %
TCCGGTGCTGGTGCTACAGCCGG-----------------------------TCGGTGCGACTAACGACGCCCTTG	13.7 %
TCCGGTGCTGGTGCTACAGCCGGCGTTGGTGGAAGTGGTTCCTCCGCCGCTCTTTCGGTGCGACTAACGACGCCCTTG	3.5 %
TCCGGTGCTGGTGCTACAGCCGGCGTTGGTGGAAGTGGTTCCTCCGCCGCTCATCGGTGCGACTAACGACGCCCTTG	1.1 %

FIGURE S1 | Comparison of *AtBON*1 editing efficiency via NHEJ by bNLSCas9 RNP or DNA transfection in Arabidopsis protoplasts. Indel (red) % of two additional biological experiments (Exp. 2 and 3) is shown by NGS analyses of the amplicons generated by PCR using genomic DNA isolated from transfected protoplasts. The *AtBON1* editing efficiency from three biological repeats is presented in FIGURE 2E.

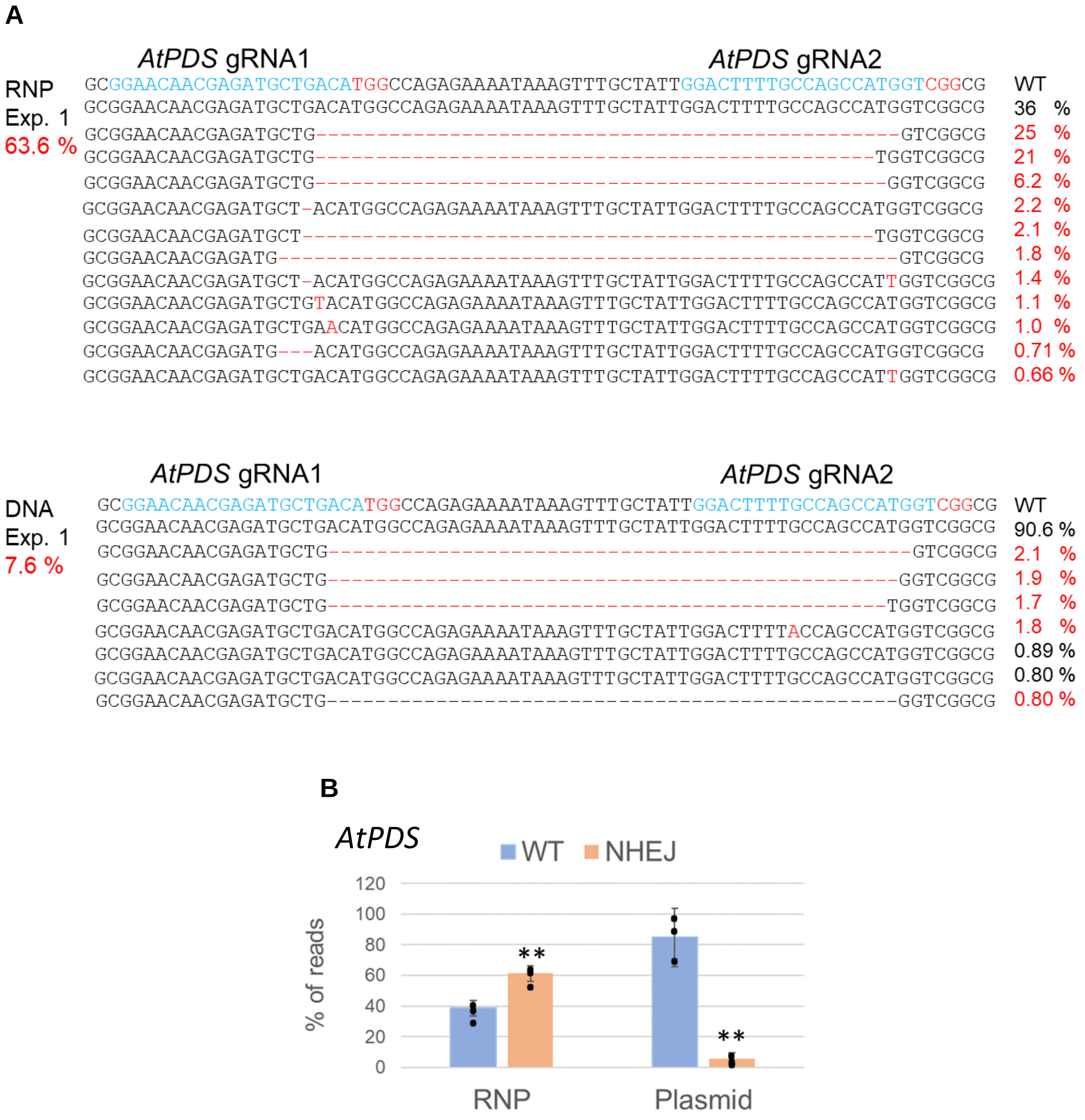

FIGURE S2 | Comparison of *AtPDS* editing efficiency via NHEJ by bNLSCas9 RNP or DNA transfection in Arabidopsis protoplasts. **(A)** Indel (red) % of one representative biological experiment is shown. **(B)** The *AtPDS* editing efficiency from three biological repeats (** $p < 0.01$; error bars, s.d., n=3).

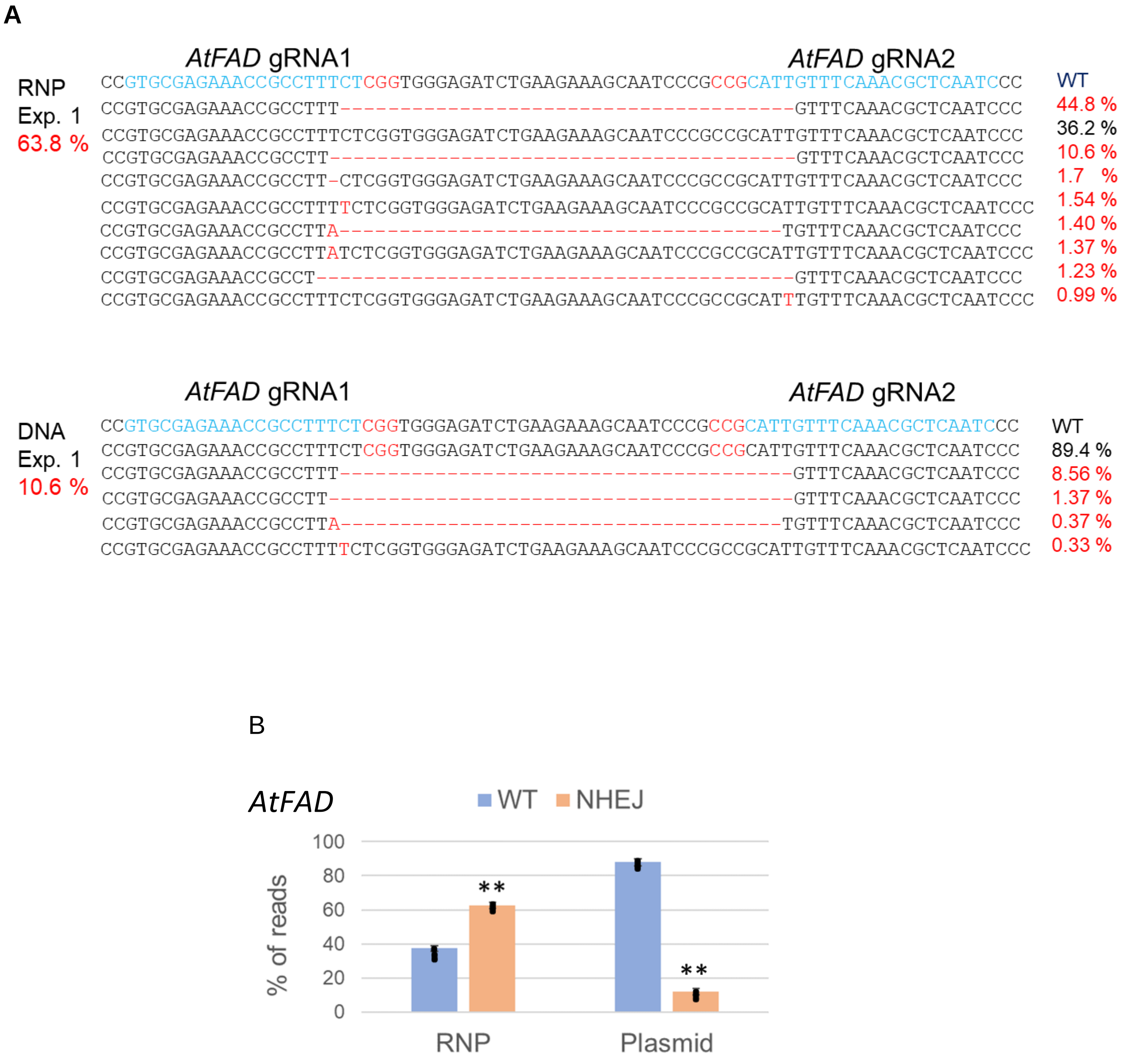

FIGURE S3 | Comparison of *AtFAD* editing effieincy via NHEJ by bNLSCas9 RNP or DNA transfection in Arabidopsis protoplasts. **(A)** Indel (red) % of one representative biological experiment is shown. **(B)** The *AtFAD* editing efficiency from three biological repeats (** $p < 0.01$; error bars, s.d., n=3).

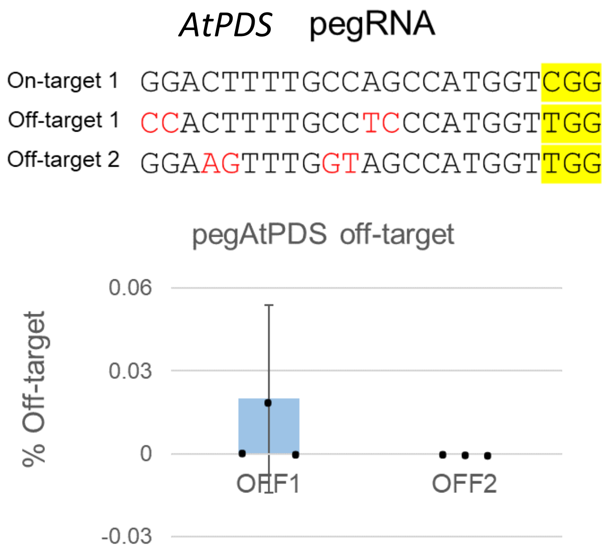

FIGURE S4 | Analyses of off-target site mutations mediated by PE RNP in Arabidopsis protoplasts. Red letter: predicted mismatch; Yellow highlight: PAM. Data from three biological repeats for two predicted off-target sites (*n*=3).

TABLE S1 | Primers used for T7 *in vitro* synthesis of gRNAs.

Gene	Forward primer sequence from 5' to 3'	Reverse primer from 5' to 3'
GFP	GAAATTAATACGACTCACTATAGGCGCTTCAAGGTGCACATGGGTTTTAGAGCTAGAAATAGCAAG	GCACCGACTCGGTGCCACTTTTTCAAGTTGATAACG
GFP	GAAATTAATACGACTCACTATAGGCTGAAGCACTGC*ACGCGT*AGTTTTAGAGCTAGAAATAGCAAG	GCACCGACTCGGTGCCACTTTTTCAAGTTGATAACG
AtBON 1	GAAATTAATACGACTCACTATAGGCTGGTGCTACAGCCGGCGTGTTTTAGAGCTAGAAATAGCAAG	GCACCGACTCGGTGCCACTTTTTCAAGTTGATAACG
AtBON 1	GAAATTAATACGACTCACTATAGGTCGTTAGTCGCACCGAGAGGTTTTAGAGCTAGAAATAGCAAG	GCACCGACTCGGTGCCACTTTTTCAAGTTGATAACG
AtALS	GAAATTAATACGACTCACTATAGGTTCCTCTTGTAGCAATCACGTTTTAGAGCTAGAAATAGCAAG	GCACCGACTCGGTGCCACTTTTTCAAGTTGATAACG
AtALS	GAAATTAATACGACTCACTATAGGTACCAATCATACGACGGTTTTAGAGCTAGAAATAGCAAG	GCACCGACTCGGTGCCACTTTTTCAAGTTGATAACG

TABLE S2 | Primers used for T7 *in vitro* synthesis of pegRNAs.

Gene	Forward primer sequence from 5' to 3'	Reverse primer from 5' to 3'
GFP	GAAATTAATACGACTCACTATAGGCTGAAGCACTGC*ACGCGT*AGTTTTAGAGCTAGAAATAGCAAG	AAGCACTGCACGCCGTAGGTGAAGCACCGACTCGGTGCCAC
GFP	GAAATTAATACGACTCACTATAGGCTGAAGCACTGC*ACGCGT*AGTTTTAGAGCTAGAAATAGCAAG	AAGCACTGCACGCCGTAGGTGAAGGTGCACCGACTCGGTGCCAC
GFP	GAAATTAATACGACTCACTATAGGCTGAAGCACTGC*ACGCGT*AGTTTTAGAGCTAGAAATAGCAAG	AAGCACTGCACGCCGTAGGTGAAGGTGGTGCACCGACTCGGTGCCAC
GFP	GAAATTAATACGACTCACTATAGGCTGAAGCACTGC*ACGCGT*AGTTTTAGAGCTAGAAATAGCAAG	AAGCACTGCACGCCGTAGGTGAAGGTGGTCAGCACCGACTCGGTGCCAC
GFP	GAAATTAATACGACTCACTATAGGCTGAAGCACTGC*ACGCGT*AGTTTTAGAGCTAGAAATAGCAAG	AAGCACTGCACGCCGTAGGTGAAGGTGGTCACGAGCACCGACTCGGTGCCAC
GFP	GAAATTAATACGACTCACTATAGGCTGAAGCACTGC*ACGCGT*AGTTTTAGAGCTAGAAATAGCAAG	AAGCACTGCACGCCGTAGGTGAAGGTGGTCACGAGGGTGCACCGACTCGGTGCCAC
GFP	GAAATTAATACGACTCACTATAGGCTGAAGCACTGC*ACGCGT*AGTTTTAGAGCTAGAAATAGCAAG	AAGCACTGCACGCCGTAGGTGAAGGTGGTCACGAGGGTGGGCCAGGGGCACCGACTCGGTGCCAC
AtPDS	GAAATTAATACGACTCACTATAGGGACTTTTGCCAGCCATGGTGTTTTAGAGCTAGAAATAGCAAG	TTTTGCCAGCCATGGTCGGCGGTTAGGCTTAGCACCGACTCGGTGC

TABLE S3 | Primers used for PCR amplification of the targeting sequences in the Arabidopsis genome.

Gene	Forward primer sequence from 5' to 3'	Reverse primer from 5' to 3'
AtBON 1	ATGCGTTGTTCGATCCAGGTACTTTTGAATC	ACGAGAGATAGATGATAGTGAAGGAAC
AtALS	ATGCCAGCAGAAGGATACGCTCGATCCTCAG	CTAAAAAGAAAGCTTCCTCAATAATCC
AtPDS	GTTGTTGCTGTTGGATTTACG	CACAGAAACTGTGAACTCAATAGCCTAC

TABLE S4 | Primers used for T7 *in vitro* synthesis of gRNAs.

Gene	Forward primer sequence from 5' to 3'	Reverse primer from 5' to 3'
AtPDS	GAAATTAATACGACTCACTATAGGGAACAACGAGATGCTGACAGTTTT AGAGCTAGAAATAGCAAG	GCACCGACTCGGTGCCACTTTTTCA AGTTGATAACG
AtPDS	GAAATTAATACGACTCACTATAGGGACTTTTGCCAGCCATGGTGTTTT AGAGCTAGAAATAGCAAG	GCACCGACTCGGTGCCACTTTTTCA AGTTGATAACG
AtFAD	GAAATTAATACGACTCACTATAGGTGCGAGAAACCGCCTTTCTGTTTT AGAGCTAGAAATAGCAAG	GCACCGACTCGGTGCCACTTTTTCA AGTTGATAACG
AtFAD	GAAATTAATACGACTCACTATAGGATTGAGCGTTTGAAACAATGGTTT TAGAGCTAGAAATAGCAAG	GCACCGACTCGGTGCCACTTTTTCA AGTTGATAACG

TABLE S5 | Primers used for PCR amplification of the targeting sequences in the *Arabidopsis* genome.

Gene	Forward primer sequence from 5' to 3'	Reverse primer from 5' to 3'
AtPDS	AAAAGTTGTTGCTGTTGGATTTACG	CACAGAAACTGTGAACTCAATAGCC
AtPDS	TTTTGTTGTTGCTGTTGGATTTACG	CACAGAAACTGTGAACTCAATAGCC
AtPDS	GGGGGTTGTTGCTGTTGGATTTACG	CACAGAAACTGTGAACTCAATAGCC
AtFAD	ATGCATGGGTGCAGGTGGAAGAATGC	AAGCATGAGGCTATAATGATGTC
AtFAD	ATCGATGGGTGCAGGTGGAAGAATGC	AAGCATGAGGCTATAATGATGTC
AtFAD	AGCTATGGGTGCAGGTGGAAGAATGC	AAGCATGAGGCTATAATGATGTC

TABLE S6 | Primers used for PCR amplification of predicted off-target site sequences.

Gene	Forward primer sequence from 5' to 3'	Reverse primer from 5' to 3'
AtBON1	GCTGTCGTTGCGCTTATC	CCTTCCTCAGTGTTGTTAGC
AtBON1	CCGTCTCACTCCCACCTC	GTCAAACCCTAGCGGCGGTG
AtBON1	GCTCCAGCTCCAGCTCCCTC	GTATTCGAACTTCACATAAG
AtPDS	CTTCCACACCCTTCTTGC	CAAGCCAGGCATCTTGCC
AtPDS	CCTAGGATTTCAAGTTGG	CAAAGATAACTTCTTGCTC
AtPDS	GATCTGTTTCTCTATAATC	GAATGTGTTCAAGCTGCG

DNA SEQUENCES

NHEJ GFP

GCGCTTCAAGGTGCACATGGAGGAAAAATCCATGGTGAGCAAGGGCGAGGAGCT
GTTCACCGGGGTGGTGCCCATCCTGGTCGAGCTGGACGGCGACGTAAACGGCCA

CAAGTTCAGCGTGTCCGGCGAGGGCGAGGGCGATGCCACCTACGGCAAGCTGAC
CCTGAAGTTCATCTGCACCACCGGCAAGCTGCCCGTGCCCTGGCCCACCCTCGTG
ACCACCTTCACCTACGGCGTGCAGTGCTTCAGCCGCTACCCCGACCACATGAAGC
AGCACGACTTCTTCAAGTCCGCCATGCCCGAAGGCTACGTCCAGGAGCGCACCAT
CTTCTTCAAGGACGACGGCAACTACAAGACCCGCGCCGAGGTGAAGTTCGAGGG
CGACACCCTGGTGAACCGCATCGAGCTGAAGGGCATCGACTTCAAGGAGGACGG
CAACATCCTGGGGCACAAGCTGGAGTACAACTACAACAGCCACAACGTCTATATCA
TGGCCGACAAGCAGAAGAACGGCATCAAGGTGAACTTCAAGATCCGCCACAACAT
CGAGGACGGCAGCGTGCAGCTCGCCGACCACTACCAGCAGAACACCCCCATCGG
CGACGGCCCCGTGCTGCTGCCCGACAACCACTACCTGAGCACCCAGTCCGCCCT
GAGCAAAGACCCCAACGAGAAGCGCGATCACATGGTCCTGCTGGAGTTCGTGACC
GCCGCCGGGATCACTCACGGCATGGACGAGCTGTACAAGTAA

Black: GFP

Blue: target sequence

Red: PAM

HDR GFP

ATGGTGAGCAAGGGCGAGGAGCTGTTCACCGGGGTGGTGCCCATCCTGGTCGAG
CTGGACGGCGACGTAAACGGCCACAAGTTCAGCGTGTCCGGCGAGGGCGAGGGC
GATGCCACCTACGGCAAGCTGACCCTGAAGTTCATCTGCACCACCGGCAAGCTGC
CCGTGCCCTGGCCCACCCTCGTGACCACCTTCACCTTACGCGTGCAGTGCTTCAG
CCGCTACCCCGACCACATGAAGCAGCACGACTTCTTCAAGTCCGCCATGCCCGAA
GGCTACGTCCAGGAGCGCACCATCTTCTTCAAGGACGACGGCAACTACAAGACCC
GCGCCGAGGTGAAGTTCGAGGGCGACACCCTGGTGAACCGCATCGAGCTGAAGG
GCATCGACTTCAAGGAGGACGGCAACATCCTGGGGCACAAGCTGGAGTACAACTA
CAACAGCCACAACGTCTATATCATGGCCGACAAGCAGAAGAACGGCATCAAGGTG
AACTTCAAGATCCGCCACAACATCGAGGACGGCAGCGTGCAGCTCGCCGACCACT
ACCAGCAGAACACCCCCATCGGCGACGGCCCCGTGCTGCTGCCCGACAACCACT
ACCTGAGCACCCAGTCCGCCCTGAGCAAAGACCCCAACGAGAAGCGCGATCACAT
GGTCCTGCTGGAGTTCGTGACCGCCGCCGGGATCACTCACGGCATGGACGAGCT
GTACAAGTAA

Black GFP

Blue: target sequence

Red: PAM

Yellow highlight: 65TLR67

bNLSCas9

ATGAAGAGACCAGCTGCTACCAAGAAGGCTGGACAGGCTAAGAAGAAGAAGGCCA
TGGATAAAAAATACAGCATTGGTCTGGACATTGGCACGAATAGCGTTGGTTGGGCA
GTGATTACCGATGAATACAAAGTCCCGTCGAAAAAATTCAAAGTGCTGGGTAACAC
CGATCGCCATAGCATTAAGAAAAACCTGATCGGTGCGCTGCTGTTTGATTCTGGCG
AAACCGCGGAAGCAACGCGTCTGAAACGTACCGCACGTCGCCGTTACACGCGCC
GTAAAAATCGTATTTGCTATCTGCAGGAAATCTTTAGCAACGAAATGGCGAAAGTC
GATGACTCATTTTTCCACCGCCTGGAAGAATCGTTTCTGGTGGAAGAAGATAAAAA
ACATGAACGTCACCCGATTTTCGGCAATATCGTTGATGAAGTCGCGTACCATGAAA
AATATCCGACGATTTACCACCTGCGTAAAAAACTGGTGGATTCTACCGACAAAGCC
GATCTGCGCCTGATTTATCTGGCACTGGCTCATATGATCAAATTTCGTGGTCACTTC
CTGATTGAAGGCGACCTGAACCCGGATAATAGTGACGTCGATAAACTGTTTATTCA
GCTGGTGCAAACCTATAATCAGCTGTTCGAAGAAAACCCGATCAATGCAAGTGGTG
TTGATGCGAAAGCCATTCTGTCCGCTCGCCTGAGTAAATCCCGCCGTCTGGAAAA
CCTGATTGCACAGCTGCCGGGTGAAAAGAAAAACGGTCTGTTTGGCAATCTGATC
GCTCTGTCACTGGGCCTGACGCCGAACTTTAAATCGAATTTCGACCTGGCAGAAG
ATGCTAAACTGCAGCTGAGCAAAGATACCTACGATGACGATCTGGACAACCTGCTG
GCGCAAATTGGCGACCAGTATGCCGACCTGTTTCTGGCGGCCAAAAATCTGTCAG
ATGCCATTCTGCTGTCGGACATCCTGCGCGTGAACACCGAAATCACGAAAGCGCC
GCTGTCAGCCTCGATGATTAAACGCTACGATGAACATCACCAGGACCTGACCCTG
CTGAAAGCACTGGTTCGTCAGCAACTGCCGGAAAAATACAAAGAAATTTTCTTTGA
CCAAAGTAAAAATGGTTATGCAGGCTACATCGATGGCGGTGCTTCCCAGGAAGAAT
TCTACAAATTCATCAAACCGATCCTGGAAAAAATGGATGGTACGGAAGAACTGCTG
GTGAAACTGAATCGTGAAGATCTGCTGCGTAAACAACGCACCTTTGACAACGGTAG
CATTCCGCATCAGATCCACCTGGGCGAACTGCATGCGATTCTGCGCCGTCAGGAA
GATTTTTATCCGTTCCTGAAAGACAACCGTGAAAAAATCGAAAAAATCCTGACGTTT
CGCATCCCGTATTACGTTGGTCCGCTGGCACGTGGTAATAGCCGCTTCGCATGGA
TGACCCGCAAATCTGAAGAAACCATTACGCCGTGGAACTTTGAAGAAGTGGTTGAT
AAAGGCGCAAGCGCTCAGTCTTTTATCGAACGTATGACCAATTTCGATAAAAACCT
GCCGAATGAAAAAGTGCTGCCGAAACATTCTCTGCTGTATGAATACTTTACCGTTTA
CAACGAACTGACGAAAGTGAAATATGTTACCGAGGGTATGCGCAAACCGGCGTTT
CTGAGTGGCGAACAGAAAAAAGCCATTGTGGATCTGCTGTTCAAAACCAATCGTAA
AGTTACGGTCAAACAGCTGAAAGAAGATTACTTCAAGAAAATTGAATGTTTCGACA
GCGTGGAAATTTCTGGTGTTGAAGATCGTTTCAACGCCTCTCTGGGCACCTATCAT
GACCTGCTGAAAATCATCAAAGACAAAGATTTTCTGGATAACGAAGAAAACGAAGA
CATTCTGGAAGATATCGTGCTGACCCTGACGCTGTTCGAAGATCGTGAAATGATTG
AAGAACGCCTGAAAACGTACGCACACCTGTTTGACGATAAAGTTATGAAACAGCTG
AAACGCCGTCGCTATACCGGTTGGGGCCGTCTGAGCCGCAAACTGATTAATGGTA
TCCGCGATAAACAATCAGGCAAAACGATTCTGGATTTCCTGAAATCGGACGGCTTT

GCCAACCGTAATTTCATGCAGCTGATCCATGACGATTCCCTGACCTTTAAAGAAGA
CATTCAGAAAGCACAAGTGTCAGGTCAAGGCGATTCGCTGCATGAACACATTGCG
AACCTGGCCGGTTCACCGGCTATCAAAAAAGGCATCCTGCAGACCGTGAAAGTCG
TGGATGAACTGGTGAAAGTTATGGGTCGTCACAAACCGGAAAACATTGTTATCGAA
ATGGCGCGCGAAAATCAGACCACGCAAAAAGGCCAGAAAAACTCGCGTGAACGCA
TGAAACGCATTGAAGAAGGTATCAAAGAACTGGGCAGCCAGATTCTGAAAGAACAT
CCGGTCGAAAACACCCAGCTGCAAAATGAAAAACTGTACCTGTATTACCTGCAAAA
TGGTCGTGACATGTATGTGGATCAGGAACTGGACATCAACCGCCTGTCTGACTATG
ATGTCGACCACATTGTGCCGCAGAGCTTTCTGAAAGACGATTCTATCGATAACAAA
GTTCTGACCCGTAGTGATAAAAACCGCGGCAAAAGCGACAATGTCCCGTCTGAAG
AAGTTGTGAAGAAAATGAAAAACTACTGGCGTCAACTGCTGAATGCGAAACTGATT
ACGCAGCGTAAATTCGATAACCTGACCAAAGCGGAACGCGGCGGTCTGTCCGAAC
TGGATAAAGCCGGTTTTATCAAACGTCAACTGGTTGAAACCCGCCAGATTACGAAA
CATGTCGCCCAGATCCTGGATTCACGCATGAACACGAAATACGACGAAAACGATAA
ACTGATCCGTGAAGTCAAAGTGATCACCCTGAAAAGTAAACTGGTTTCCGATTTCC
GTAAAGACTTTCAGTTCTACAAAGTCCGCGAAATTAACAATTACCATCACGCACAC
GATGCTTATCTGAATGCAGTGGTTGGTACCGCTCTGATCAAAAAATATCCGAAACT
GGAAAGCGAATTTGTGTATGGCGATTACAAAGTCTATGACGTGCGCAAAATGATTG
CGAAATCCGAACAGGAAATCGGCAAAGCGACCGCCAAATACTTTTTCTATTCAAAC
ATCATGAACTTTTTCAAAACCGAAATTACGCTGGCAAATGGTGAAATTCGTAAACGC
CCGCTGATCGAAACCAACGGTGAAACGGGCGAAATTGTGTGGGATAAAGGCCGTG
ACTTCGCGACCGTTCGCAAAGTCCTGTCGATGCCGCAAGTGAATATCGTGAAGAA
AACCGAAGTGCAGACGGGCGGTTTTAGTAAAGAATCCATCCTGCCGAAACGTAAC
AGCGATAAACTGATTGCGCGCAAAAAAGATTGGGACCCGAAAAAATACGGCGGTT
TTGATAGTCCGACGGTTGCATATTCCGTCCTGGTCGTGGCTAAAGTCGAAAAAGGT
AAAAGTAAAAAACTGAAATCCGTGAAAGAACTGCTGGGCATTACCATCATGGAACG
TAGCTCTTTTGAGAAAAACCCGATTGACTTCCTGGAAGCCAAAGGTTACAAAGAAG
TGAAAAAAGATCTGATCATCAAACTGCCGAAATATAGCCTGTTCGAACTGGAAAAC
GGCCGTAAACGCATGCTGGCATCTGCTGGTGAACTGCAGAAAGGCAATGAACTGG
CACTGCCGAGTAAATATGTTAACTTTCTGTACCTGGCTAGCCATTATGAAAAACTGA
AAGGTTCTCCGGAAGATAACGAACAGAAACAACTGTTCGTCGAACAACATAAACAC
TACCTGGATGAAATCATCGAACAGATCTCAGAATTCTCGAAACGCGTGATTCTGGC
GGATGCCAATCTGGACAAAGTTCTGAGCGCGTATAACAAACATCGTGATAAACCGA
TTCGCGAACAGGCCGAAAATATTATCCACCTGTTTACCCTGACGAACCTGGGCGCA
CCGGCAGCTTTTAAATACTTCGATACCACGATCGACCGTAAACGCTATACCTCAAC
GAAAGAAGTTCTGGATGCTACCCTGATTCATCAATCGATCACCGGTCTGTATGAAA
CGCGTATTGATCTGAGTCAGCTGGGCGGTGACGGCGGTGGCTCCGGCACCCGTC
TGCCGAAAAAGAAACGCAAAGTGGGCGGTGGCTCG*CATCATCATCATCATCAC*TA
A

Green: bipartite nuclear localization signal

Black underlined: *Cas9*
Green underlined: monopartite NLS
Black italic and underlined: 6XHIS tag

nCas9-RT

AAGCGCCCGGCTGCGACCAAGAAAGCGGGACAAGCTAAGAAGAAAAAAGCCATG
GATAAAAAATACAGCATTGGTCTGGACATTGGCACGAATAGCGTTGGTTGGGCAGT
GATTACCGATGAATACAAAGTCCCGTCGAAAAAATTCAAAGTGCTGGGTAACACCG
ATCGCCATAGCATTAAGAAAAACCTGATCGGTGCGCTGCTGTTTGATTCTGGCGAA
ACCGCGGAAGCAACGCGTCTGAAACGTACCGCACGTCGCCGTTACACGCGCCGT
AAAAATCGTATTTGCTATCTGCAGGAAATCTTTAGCAACGAAATGGCGAAAGTCGA
TGACTCATTTTTCCACCGCCTGGAAGAATCGTTTCTGGTGGAAGAAGATAAAAAAC
ATGAACGTCACCCGATTTTCGGCAATATCGTTGATGAAGTCGCGTACCATGAAAAA
TATCCGACGATTTACCACCTGCGTAAAAAACTGGTGGATTCTACCGACAAAGCCGA
TCTGCGCCTGATTTATCTGGCACTGGCTCATATGATCAAATTTCGTGGTCACTTCCT
GATTGAAGGCGACCTGAACCCGGATAATAGTGACGTCGATAAACTGTTTATTCAGC
TGGTGCAAACCTATAATCAGCTGTTCGAAGAAAACCCGATCAATGCAAGTGGTGTT
GATGCGAAAGCCATTCTGTCCGCTCGCCTGAGTAAATCCCGCCGTCTGGAAAACC
TGATTGCACAGCTGCCGGGTGAAAAGAAAAACGGTCTGTTTGGCAATCTGATCGCT
CTGTCACTGGGCCTGACGCCGAACTTTAAATCGAATTTCGACCTGGCAGAAGATG
CTAAACTGCAGCTGAGCAAAGATACCTACGATGACGATCTGGACAACCTGCTGGC
GCAAATTGGCGACCAGTATGCCGACCTGTTTCTGGCGGCCAAAAATCTGTCAGAT
GCCATTCTGCTGTCGGACATCCTGCGCGTGAACACCGAAATCACGAAAGCGCCGC
TGTCAGCCTCGATGATTAAACGCTACGATGAACATCACCAGGACCTGACCCTGCTG
AAAGCACTGGTTCGTCAGCAACTGCCGGAAAAATACAAAGAAATTTTCTTTGACCA
AAGTAAAAATGGTTATGCAGGCTACATCGATGGCGGTGCTTCCCAGGAAGAATTCT
ACAAATTCATCAAACCGATCCTGGAAAAAATGGATGGTACGGAAGAACTGCTGGTG
AAACTGAATCGTGAAGATCTGCTGCGTAAACAACGCACCTTTGACAACGGTAGCAT
TCCGCATCAGATCCACCTGGGCGAACTGCATGCGATTCTGCGCCGTCAGGAAGAT
TTTTATCCGTTCCTGAAAGACAACCGTGAAAAAATCGAAAAAATCCTGACGTTTCGC
ATCCCGTATTACGTTGGTCCGCTGGCACGTGGTAATAGCCGCTTCGCATGGATGA
CCCGCAAATCTGAAGAAACCATTACGCCGTGGAACTTTGAAGAAGTGGTTGATAAA
GGCGCAAGCGCTCAGTCTTTTATCGAACGTATGACCAATTTCGATAAAAACCTGCC
GAATGAAAAAGTGCTGCCGAAACATTCTCTGCTGTATGAATACTTTACCGTTTACAA
CGAACTGACGAAAGTGAAATATGTTACCGAGGGTATGCGCAAACCGGCGTTTCTG
AGTGGCGAACAGAAAAAAGCCATTGTGGATCTGCTGTTCAAAACCAATCGTAAAGT
TACGGTCAAACAGCTGAAAGAAGATTACTTCAAGAAAATTGAATGTTTCGACAGCG
TGGAAATTTCTGGTGTTGAAGATCGTTTCAACGCCTCTCTGGGCACCTATCATGAC
CTGCTGAAAATCATCAAAGACAAAGATTTTCTGGATAACGAAGAAAACGAAGACATT

CTGGAAGATATCGTGCTGACCCTGACGCTGTTCGAAGATCGTGAAATGATTGAAGA
ACGCCTGAAAACGTACGCACACCTGTTTGACGATAAAGTTATGAAACAGCTGAAAC
GCCGTCGCTATACCGGTTGGGGCCGTCTGAGCCGCAAACTGATTAATGGTATCCG
CGATAAACAATCAGGCAAAACGATTCTGGATTTCCTGAAATCGGACGGCTTTGCCA
ACCGTAATTTCATGCAGCTGATCCATGACGATTCCCTGACCTTTAAAGAAGACATTC
AGAAAGCACAAGTGTCAGGTCAAGGCGATTCGCTGCATGAACACATTGCGAACCT
GGCCGGTTCACCGGCTATCAAAAAAGGCATCCTGCAGACCGTGAAAGTCGTGGAT
GAACTGGTGAAAGTTATGGGTCGTCACAAACCGGAAAACATTGTTATCGAAATGGC
GCGCGAAAATCAGACCACGCAAAAAGGCCAGAAAAACTCGCGTGAACGCATGAAA
CGCATTGAAGAAGGTATCAAAGAACTGGGCAGCCAGATTCTGAAAGAACATCCGG
TCGAAAACACCCAGCTGCAAAATGAAAAACTGTACCTGTATTACCTGCAAAATGGT
CGTGACATGTATGTGGATCAGGAACTGGACATCAACCGCCTGTCTGACTATGATGT
CGACGCCATTGTGCCGCAGAGCTTTCTGAAAGACGATTCTATCGATAACAAAGTTC
TGACCCGTAGTGATAAAAACCGCGGCAAAAGCGACAATGTCCCGTCTGAAGAAGT
TGTGAAGAAAATGAAAAACTACTGGCGTCAACTGCTGAATGCGAAACTGATTACGC
AGCGTAAATTCGATAACCTGACCAAAGCGGAACGCGGCGGTCTGTCCGAACTGGA
TAAAGCCGGTTTTATCAAACGTCAACTGGTTGAAACCCGCCAGATTACGAAACATG
TCGCCCAGATCCTGGATTCACGCATGAACACGAAATACGACGAAAACGATAAACTG
ATCCGTGAAGTCAAAGTGATCACCCTGAAAAGTAAACTGGTTTCCGATTTCCGTAA
AGACTTTCAGTTCTACAAAGTCCGCGAAATTAACAATTACCATCACGCACACGATG
CTTATCTGAATGCAGTGGTTGGTACCGCTCTGATCAAAAAATATCCGAAACTGGAA
AGCGAATTTGTGTATGGCGATTACAAAGTCTATGACGTGCGCAAAATGATTGCGAA
ATCCGAACAGGAAATCGGCAAAGCGACCGCCAAATACTTTTTCTATTCAAACATCA
TGAACTTTTTCAAAACCGAAATTACGCTGGCAAATGGTGAAATTCGTAAACGCCCG
CTGATCGAAACCAACGGTGAAACGGGCGAAATTGTGTGGGATAAAGGCCGTGACT
TCGCGACCGTTCGCAAAGTCCTGTCGATGCCGCAAGTGAATATCGTGAAGAAAAC
CGAAGTGCAGACGGGCGGTTTTAGTAAAGAATCCATCCTGCCGAAACGTAACAGC
GATAAACTGATTGCGCGCAAAAAAGATTGGGACCCGAAAAAATACGGCGGTTTTGA
TAGTCCGACGGTTGCATATTCCGTCCTGGTCGTGGCTAAAGTCGAAAAAGGTAAAA
GTAAAAAACTGAAATCCGTGAAAGAACTGCTGGGCATTACCATCATGGAACGTAGC
TCTTTTGAGAAAAACCCGATTGACTTCCTGGAAGCCAAAGGTTACAAAGAAGTGAA
AAAAGATCTGATCATCAAACTGCCGAAATATAGCCTGTTCGAACTGGAAAACGGCC
GTAAACGCATGCTGGCATCTGCTGGTGAACTGCAGAAAGGCAATGAACTGGCACT
GCCGAGTAAATATGTTAACTTTCTGTACCTGGCTAGCCATTATGAAAAACTGAAAG
GTTCTCCGGAAGATAACGAACAGAAACAACTGTTCGTCGAACAACATAAACACTAC
CTGGATGAAATCATCGAACAGATCTCAGAATTCTCGAAACGCGTGATTCTGGCGGA
TGCCAATCTGGACAAAGTTCTGAGCGCGTATAACAAACATCGTGATAAACCGATTC
GCGAACAGGCCGAAAATATTATCCACCTGTTTACCCTGACGAACCTGGGCGCACC
GGCAGCTTTTAAATACTTCGATACCACGATCGACCGTAAACGCTATACCTCAACGA
AAGAAGTTCTGGATGCTACCCTGATTCATCAATCGATCACCGGTCTGTATGAAACG

CGTATTGATCTGAGTCAGCTGGGCGGTGACGGCGGTGGCTCCATG**TCTGGAGGA**
TCTAGCGGAGGATCCTCTGGCAGCGAGACACCAGGAACAAGCGAGTCAGCAAC
ACCAGAGAGCAGTGGCGGCAGCAGCGGCGGCAGCAGC*ACCCTAAATATAGAAG*
ATGAGTATCGGCTACATGAGACCTCAAAAGAGCCAGATGTTTCTCTAGGGTCCACA
TGGCTGTCTGATTTTCCTCAGGCCTGGGCGGAAACCGGGGGCATGGGACTGGCA
GTTCGCCAAGCTCCTCTGATCATACCTCTGAAAGCAACCTCTACCCCCGTGTCCAT
AAAACAATACCCCATGTCACAAGAAGCCAGACTGGGGATCAAGCCCCACATACAG
AGACTGTTGGACCAGGGAATACTGGTACCCTGCCAGTCCCCCTGGAACACGCCCC
TGCTACCCGTTAAGAAACCAGGGACTAATGATTATAGGCCTGTCCAGGATCTGAGA
GAAGTCAACAAGCGGGTGGAAGACATCCACCCCACCGTGCCCAACCCTTACAACC
TCTTGAGCGGGCTCCCACCGTCCCACCAGTGGTACACTGTGCTTGATTTAAAGGAT
GCCTTTTTCTGCCTGAGACTCCACCCCACCAGTCAGCCTCTCTTCGCCTTTGAGTG
GAGAGATCCAGAGATGGGAATCTCAGGACAATTGACCTGGACCAGACTCCCACAG
GGTTTCAAAAACAGTCCCACCCTGTTTAATGAGGCACTGCACAGAGACCTAGCAGA
CTTCCGGATCCAGCACCCAGACTTGATCCTGCTACAGTACGTGGATGACTTACTGC
TGGCCGCCACTTCTGAGCTAGACTGCCAACAAGGTACTCGGGCCCTGTTACAAAC
CCTAGGGAACCTCGGGTATCGGGCCTCGGCCAAGAAAGCCCAAATTTGCCAGAAA
CAGGTCAAGTATCTGGGGTATCTTCTAAAAGAGGGTCAGAGATGGCTGACTGAGG
CCAGAAAAGAGACTGTGATGGGGCAGCCTACTCCGAAGACCCCTCGACAACTAAG
GGAGTTCCTAGGGAAGGCAGGCTTCTGTCGCCTCTTCATCCCTGGGTTTGCAGAA
ATGGCAGCCCCCCTGTACCCTCTCACCAAACCGGGGACTCTGTTTAATTGGGGCC
CAGACCAACAAAAGGCCTATCAAGAAATCAAGCAAGCTCTTCTAACTGCCCCAGCC
CTGGGGTTGCCAGATTTGACTAAGCCCTTTGAACTCTTTGTCGACGAGAAGCAGG
GCTACGCCAAAGGTGTCCTAACGCAAAAACTGGGACCTTGGCGTCGGCCGGTGG
CCTACCTGTCCAAAAAGCTAGACCCAGTAGCAGCTGGGTGGCCCCCTTGCCTACG
GATGGTAGCAGCCATTGCCGTACTGACAAAGGATGCAGGCAAGCTAACCATGGGA
CAGCCACTAGTCATTCTGGCCCCCCATGCAGTAGAGGCACTAGTCAAACAACCCC
CCGACCGCTGGCTTTCCAACGCCCGGATGACTCACTATCAGGCCTTGCTTTTGGA
CACGGACCGGGTCCAGTTCGGACCGGTGGTAGCCCTGAACCCGGCTACGCTGCT
CCCACTGCCTGAGGAAGGGCTGCAACACAACTGCCTTGATATCCTGGCCGAAGCC
CACGGAACCCGACCCGACCTAACGGACCAGCCGCTCCCAGACGCCGACCACACC
TGGTACACGGATGGAAGCAGTCTCTTACAAGAGGGACAGCGTAAGGCGGGAGCT
GCGGTGACCACCGAGACCGAGGTAATCTGGGCTAAAGCCCTGCCAGCCGGGACA
TCCGCTCAGCGGGCTGAACTGATAGCACTCACCCAGGCCCTAAAGATGGCAGAAG
GTAAGAAGCTAAATGTTTATACTGATAGCCGTTATGCTTTTGCTACTGCCCATATCC
ATGGAGAAATATACAGAAGGCGTGGGTGGCTCACATCAGAAGGCAAAGAGATCAA
AAATAAAGACGAGATCTTGGCCCTACTAAAAGCCCTCTTTCTGCCCAAAAGACTTA
GCATAATCCATTGTCCAGGACATCAAAAGGGACACAGCGCCGAGGCTAGAGGCAA
CCGGATGGCTGACCAAGCGGCCCGAAAGGCAGCCATCACAGAGACTCCAGACAC

*CTCTACCCTCCTCATAGAAAATTCATCACCC*ATTGGCACCCGTCTGCCGAAAAAGAAACGCAAAGTGGGCGGTGGCTCG*CATCATCATCATCATCAC*TAA

Green: bipartite nuclear localization signal

Black underlined: *SpCas9*

Black bold: linker

Black italic M-MLV

Green underlined: monopartite NLS

Black italic and underlined: 6XHIS tag
