# Supplementary material for: A Versatile and Efficient Plant Protoplast Platform for Genome Editing by Cas9 RNPs
Source: Front Genome Ed. 2021 Dec 22;3:719190. doi: 10.3389/fgeed.2021.719190 (PMC8729822; doi:10.3389/fgeed.2021.719190)
Supplement: Supplementary file 2 [file DataSheet1.pdf]

SUPPLEMENTAL MATERIAL for "A Versatile and Efficient Plant Protoplast Platform for Genome Editing by Cas9 RNPs"

Supplementary Figure S5

AtBON1 DNA 1
>BPCR1_NG6_CONTIG_243_p1   20866 pairs of NGS reads, 35.62%
AACGAGAGATAGATGATAGTGAAGGAACCTCTATCTGCGAGAAGAGTCCGTTGAAACCTTTGGAC
TTGAGATAGTAATCAAGGGCGTCGTTAGTCgcaccgagagcggcggaggaaccacttccaccaac
gccggctgtagcaccagCACCGGAGGCGACATCGGAGCAACAATTCCCCATAATTTTTGATTTCC
CAACAAAAAAAAACTGCGGATTCAAAAGTACCTGGATCGAACAACCGAT
>BPCR1_NG6_CONTIG_243_p2   15614 pairs of NGS reads, 26.65%
AACGAGAGATAGATGATAGTGAAGGAACCTCTATCTGCGAGAAGAGTCCGTTGAAACCTTTGGAC
TTGAGATAGTAATCAAGGGCGTCGTTAGTCGCAccgagagcggcggaggaaccacttccaccaac
gccggctgtagcaccagCACCGGAGGCGACATCGGAGCAACAATTCCCCATAATTTTTGATTTCC
CAACAAAAAAAAACTGCGGATTCAAAAGTACCTGGATCGAACAACAGCT
>BPCR1_NG6_CONTIG_243_p3   7165 pairs of NGS reads, 12.23%
AACGAGAGATAGATGATAGTGAAGGAACCTCTATCTGCGAGAAGAGTCCGTTGAAACCTTTGGAC
TTGAGATAGTAATCAAGGGCGTCGTTAGTCGCACcgagagcggcggaggaaccacttccaccaac
gccggctgtagcaccagCACCGGAGGCGACATCGGAGCAACAATTCCCCATAATTTTTGATTTCC
CAACAAAAAAAAACTGCGGATTCAAAAGTACCTGGATCGAACAACGCAT
>BPCR1_NG6_CONTIG_213_p4   4391 pairs of NGS reads, 7.49%
ACGAGAGATAGATGATAGTGAAGGAACCTCTATCTGCGAGAAGAGTCCGTTGAAACCTTTGGACT
TGagatagtaatcaagggcgtcgttagtcgcaccgaccggctgtagcaccagcaccggaggcgac
atcggagcaacaattcCCCATAATTTTTGATTTCCCAACAAAAAAAAACTGCGGATTCAAAAGTAC
CTGGATCGAACAACCGAT
>BPCR1_NG6_CONTIG_213_p5   2629 pairs of NGS reads, 4.48%
ACGAGAGATAGATGATAGTGAAGGAACCTCTATCTGCGAGAAGAGTCCGTTGAAACCTTTGGACT
TGAGatagtaatcaagggcgtcgttagtcgcaccgaccggctgtagcaccagcaccggaggcgac
atcggagcaacaattcCCCATAATTTTTGATTTCCCAACAAAAAAAAACTGCGGATTCAAAAGTAC
CTGGATCGAACAACAGCT
>BPCR1_NG6_CONTIG_213_p6   1292 pairs of NGS reads, 2.2%
ACGAGAGATAGATGATAGTGAAGGAACCTCTATCTGCGAGAAGAGTCCGTTGAAACCTTTGGACT
TGAGAtagtaatcaagggcgtcgttagtcgcaccgaccggctgtagcaccagcaccggaggcgac
atcggagcaacaattcCCCATAATTTTTGATTTCCCAACAAAAAAAAACTGCGGATTCAAAAGTAC
CTGGATCGAACAACGCAT
>BPCR1_NG6_CONTIG_166_p7   308 pairs of NGS reads, 0.52%
ACGAGAGATAGATGATAGTGAAGGaacaagatgttataccttctcaagctcctcaagaacagact
tggcaacttcacccttttgttcaaaaagagcatcaagcgtcatcattgggaccaaagccctaacc
actatgattcaaAAGTACCTGGATCGAACAACCGAT
>BPCR1_NG6_CONTIG_243_p8   318 pairs of NGS reads, 0.54%
ACGAGAGATAGATGATAGTGAAGGAACCTCTATCTGCGAGAAGAGTCCGTTGAAACCTTTGGACT
TGAGATAGTAATCAAGGGCGTCGTTAGTCGCACCGAagagcggcggaggaaccacttccaccaac
gccggctgtagcACCAGCACCGGAGGCGACATCGGAGCAACAATTCCCCATAATTTTTGATTTCC
CAACAAAAAAAAACTGCGGATTCAAAAGTACCTGGATCGAACAACCGAT
>BPCR1_NG6_CONTIG_212_p9   221 pairs of NGS reads, 0.37%
ACGAGAGATAGATGATAGTGAAGGAACCTCTATCTGCGAGAAGAGTCCGTTGAAACCTTTGGACT
TGAGAtagtaatcaagggcgtcgttagtcgcaccgccggctgtagcaccagcaccggaggcgaca
tcggagcaacaaTTCCCCATAATTTTTGATTTCCCAACAAAAAAAAACTGCGGATTCAAAAGTACC

TGGATCGAACAACCGAT
>BPCR1_NG6_CONTIG_243_p10   197 pairs of NGS reads, 0.33%
ACGAGAGATAGATGATAGTGAAGGAACCTCTATCTGCGAGAAGAGTCCGTTGAAACCTTTGGACT
TGAGATAGTAATCAAGGGCGTCGTTAGTCGCACccgagagcggcggaggaaccacttccaccaac
gccggctgtagcACCAGCACCGGAGGCGACATCGGAGCAACAATTCCCCATAATTTTTGATTTCC
CAACAAAAAAAAACTGCGGATTCAAAAGTACCTGGATCGAACAACAGCT
>BPCR1_NG6_CONTIG_243_p11   172 pairs of NGS reads, 0.29%
ACGAGAGATAGATGATAGTGAAGGAACCTCTATCTGCGAGAAGAGTCCGTTGAAACCTTTGGACT
TGAGATAGTAATCAAGGGCGTCGTTAGTCGCACCGAgagcggcggaggaaccacttccaccaacg
accggctgtagcACCAGCACCGGAGGCGACATCGGAGCAACAATTCCCCATAATTTTTGATTTCC
CAACAAAAAAAAACTGCGGATTCAAAAGTACCTGGATCGAACAACCGAT
>BPCR1_NG6_CONTIG_166_p12   170 pairs of NGS reads, 0.29%
ACGAGAGATAGATGATAGTGAAGGaacaagatgttataccttctcaagctcctcaagaacagact
tggcaacttcaccctttgttcaaaaagagcatcaagcgtcatcattgggaccaaagccctaacc
actatgattcaaAAGTACCTGGATCGAACAACAGCT
>BPCR1_NG6_CONTIG_244_p13   139 pairs of NGS reads, 0.23%
AACGAGAGATAGATGATAGTGAAGGAACCTCTATCTGCGAGAAGAGTCCGTTGAAACCTTTGGAC
TTGAGATAGTAATCAAGGGCGTCGTTAGTCGCACCGAgagcggcggaggaaccacttccaccaac
gccggctgtagcaccagCACCGGAGGCGACATCGGAGCAACAATTCCCCATAATTTTTGATTTCC
CAACAAAAAAAAAACTGCGGATTCAAAAGTACCTGGATCGAACAACCGAT
>BPCR1_NG6_CONTIG_243_p14   127 pairs of NGS reads, 0.21%
ACGAGAGATAGATGATAGTGAAGGAACCTCTATCTGCGAGAAGAGTCCGTTGAAACCTTTGGACT
TGAGATAGTAATCAAGGGCGTCGTTAGTCGCACCcgagagcggcggaggaaccacttccaccaac
gccggctgtagcACCAGCACCGGAGGCGACATCGGAGCAACAATTCCCCATAATTTTTGATTTCC
CAACAAAAAAAAACTGCGGATTCAAAAGTACCTGGATCGAACAACGCAT
>BPCR1_NG6_CONTIG_243_p15   115 pairs of NGS reads, 0.19%
ACGAGAGATAGATGATAGTGAAGGAACCTCTATCTGCGAGAAGAGTCCGTTGAAACCTTTGGACT
TGAGATAGTAATCAAGGGCGTCGTTAGTCGCACCGAgagcggcggaggaaccacttccaccaacg
accggctgtagcACCAGCACCGGAGGCGACATCGGAGCAACAATTCCCCATAATTTTTGATTTCC
CAACAAAAAAAAACTGCGGATTCAAAAGTACCTGGATCGAACAACAGCT
>BPCR1_NG6_CONTIG_243_p16   109 pairs of NGS reads, 0.18%
ACGAGAGATAGATGATAGTGAAGGAACCTCTATCTGCGAGAAGAGTCCGTTGAAACCTTTGGACT
TGAGATAGTAATCAAGGGCGTCGTTAGTCGCACCGAgagcggcggaggaaccacttccaccaacg
tccggctgtagcACCAGCACCGGAGGCGACATCGGAGCAACAATTCCCCATAATTTTTGATTTCC
CAACAAAAAAAAACTGCGGATTCAAAAGTACCTGGATCGAACAACCGAT
>BPCR1_NG6_CONTIG_244_p17   105 pairs of NGS reads, 0.17%
AACGAGAGATAGATGATAGTGAAGGAACCTCTATCTGCGAGAAGAGTCCGTTGAAACCTTTGGAC
TTGAGATAGTAATCAAGGGCGTCGTTAGTCGCACCGAgagcggcggaggaaccacttccaccaac
gccggctgtagcaccagCACCGGAGGCGACATCGGAGCAACAATTCCCCATAATTTTTGATTTCC
CAACAAAAAAAAAACTGCGGATTCAAAAGTACCTGGATCGAACAACAGCT
>BPCR1_NG6_CONTIG_212_p18   103 pairs of NGS reads, 0.17%
ACGAGAGATAGATGATAGTGAAGGAACCTCTATCTGCGAGAAGAGTCCGTTGAAACCTTTGGACT
TGAGAtagtaatcaagggcgtcgttagtcgcaccgacggctgtagcaccagcaccggaggcgaca
tcggagcaacaaTTCCCCATAATTTTTGATTTCCCAACAAAAAAAAACTGCGGATTCAAAAGTACC
TGGATCGAACAACCGAT
>BPCR1_NG6_CONTIG_212_p19   99 pairs of NGS reads, 0.16%
ACGAGAGATAGATGATAGTGAAGGAACCTCTATCTGCGAGAAGAGTCCGTTGAAACCTTTGGACT
TGAGAtagtaatcaagggcgtcgttagtcgcaccgccggctgtagcaccagcaccggaggcgaca
tcggagcaacaaTTCCCCATAATTTTTGATTTCCCAACAAAAAAAAACTGCGGATTCAAAAGTACC
TGGATCGAACAACAGCT
>BPCR1_NG6_CONTIG_243_p20   93 pairs of NGS reads, 0.15%
ACGAGAGATAGATGATAGTGAAGGAACCTCTATCTGCGAGAAGAGTCCGTTGAAACCTTTGGACT

TGAGATAGTAATCAAGGGCGTCGTTAGTCGCACCGAtgagcggcggaggaaccacttccaccaac
gccggctgtagcACCAGCACCGGAGGCGACATCGGAGCAACAATTCCCCATAATTTTTGATTTCC
CAACAAAAAAAACTGCGGATTCAAAAGTACCTGGATCGAACAACCGAT
>BPCR1_NG6_CONTIG_243_p21   89 pairs of NGS reads, 0.15%
ACGAGAGATAGATGATAGTGAAGGAACCTCTATCTGCGAGAAGAGTCCGTTGAAACCTTTGGACT
TGAGATAGTAATCAAGGGCGTCGTTAGTCGCACCGAtgagcggcggaggaaccacttccaccaac
gccggctgtagcACCAGCACCGGAGGCGACATCGGAGCAACAATTCCCCATAATTTTTGATTTCC
CAACAAAAAAAACTGCGGATTCAAAAGTACCTGGATCGAACAACAGCT
>BPCR1_NG6_CONTIG_243_p22   84 pairs of NGS reads, 0.14%
ACGAGAGATAGATGATAGTGAAGGAACCTCTATCTGCGAGAAGAGTCCGTTGAAACCTTTGGACT
TGAGATAGTAATCAAGGGCGTCGTTAGTCGCACCGAagagcggcggaggaaccacttccaccaacg
tccggctgtagcACCAGCACCGGAGGCGACATCGGAGCAACAATTCCCCATAATTTTTGATTTCC
CAACAAAAAAAACTGCGGATTCAAAAGTACCTGGATCGAACAACAGCT
>BPCR1_NG6_CONTIG_241_p23   76 pairs of NGS reads, 0.12%
ACGAGAGATAGATGATAGTGAAGGAACCTCTATCTGCGAGAAGAGTCCGTTGAAACCTTTGGACT
TGAGATAGTAATCAAGGGCGTCGTTAGTCGCACCgagagcggcggaggaaccacttccaccaacc
cggctgtagcacCAGCACCGGAGGCGACATCGGAGCAACAATTCCCCATAATTTTTGATTTCCCA
ACAAAAAAAACTGCGGATTCAAAAGTACCTGGATCGAACAACCGAT
>BPCR1_NG6_CONTIG_241_p24   74 pairs of NGS reads, 0.12%
ACGAGAGATAGATGATAGTGAAGGAACCTCTATCTGCGAGAAGAGTCCGTTGAAACCTTTGGACT
TGAGATAGTAATCAAGGGCGTCGTTAGTCGCACCgagagcggcggaggaaccacttccaccaacg
cggctgtagcacCAGCACCGGAGGCGACATCGGAGCAACAATTCCCCATAATTTTTGATTTCCCA
ACAAAAAAAACTGCGGATTCAAAAGTACCTGGATCGAACAACCGAT
>BPCR1_NG6_CONTIG_210_p25   70 pairs of NGS reads, 0.11%
ACGAGAGATAGATGATAGTGAAGGAACCTCTATCTGCGAGAAGAGTCCGTTGAAACCTTTGGACT
TGAgatagtaatcaagggcgtcgttagtcgcacccggctgtagcaccagcaccggaggcgacatc
ggagcaacaattCCCCATAATTTTTGATTTCCCAACAAAAAAAACTGCGGATTCAAAAGTACCTG
GATCGAACAACCGAT
>BPCR1_NG6_CONTIG_240_p26   70 pairs of NGS reads, 0.11%
ACGAGAGATAGATGATAGTGAAGGAACCTCTATCTGCGAGAAGAGTCCGTTGAAACCTTTGGACT
TGAGATAGTAATCAAGGGCGTCGTTAGTCGCACcgagagcggcggaggaaccacttccaccaacc
ggctgtagcaccAGCACCGGAGGCGACATCGGAGCAACAATTCCCCATAATTTTTGATTTCCCAA
CAAAAAAAACTGCGGATTCAAAAGTACCTGGATCGAACAACCGAT
>BPCR1_NG6_CONTIG_242_p27   69 pairs of NGS reads, 0.11%
AACGAGAGATAGATGATAGTGAAGGAACCTCTATCTGCGAGAAGAGTCCGTTGAAACCTTTGGAC
TTGAGATAGTAATCAAGGGCGTCGTTAGTCGCACCgagagcggcggaggaaccacttccaccaac
gccggctgtagcaccagCACCGGAGGCGACATCGGAGCAACAATTCCCCATAATTTTTGATTTCC
CAACAAAAAAACTGCGGATTCAAAAGTACCTGGATCGAACAACCGAT
>BPCR1_NG6_CONTIG_240_p28   67 pairs of NGS reads, 0.11%
ACGAGAGATAGATGATAGTGAAGGAACCTCTATCTGCGAGAAGAGTCCGTTGAAACCTTTGGACT
TGAGATAGTAATCAAGGGCGTCGTTAGTCGCACcgagcggcggaggaaccacttccaccaacgcc
ggctgtagcaccAGCACCGGAGGCGACATCGGAGCAACAATTCCCCATAATTTTTGATTTCCCAA
CAAAAAAAACTGCGGATTCAAAAGTACCTGGATCGAACAACAGCT
>BPCR1_NG6_CONTIG_241_p29   65 pairs of NGS reads, 0.11%
ACGAGAGATAGATGATAGTGAAGGAACCTCTATCTGCGAGAAGAGTCCGTTGAAACCTTTGGACT
TGAGATAGTAATCAAGGGCGTCGTTAGTCGCACCggagcggcggaggaaccacttccaccaacgc
cggctgtagcacCAGCACCGGAGGCGACATCGGAGCAACAATTCCCCATAATTTTTGATTTCCCA
ACAAAAAAAACTGCGGATTCAAAAGTACCTGGATCGAACAACCGAT
>BPCR1_NG6_CONTIG_243_p30   60 pairs of NGS reads, 0.1%
ACGAGAGATAGATGATAGTGAAGGAACCTCTATCTGCGAGAAGAGTCCGTTGAAACCTTTGGACT
TGAGATAGTAATCAAGGGCGTCGTTAGTCGCACCGAagagcggcggaggaaccacttccaccaacg
tccggctgtagcACCAGCACCGGAGGCGACATCGGAGCAACAATTCCCCATAATTTTTGATTTCC

CAACAAAAAAAACTGCGGATTCAAAAGTACCTGGATCGAACAACGCAT
>BPCR1_NG6_CONTIG_212_p31   60 pairs of NGS reads, 0.1%
ACGAGAGATAGATGATAGTGAAGGAACCTCTATCTGCGAGAAGAGTCCGTTGAAACCTTTGGACT
TGAGAtagtaatcaagggcgtcgttagtcgcaccgacggctgtagcaccagcaccggaggcgaca
tcggagcaacaaTTCCCCATAATTTTTGATTTCCCAACAAAAAAAACTGCGGATTCAAAAGTACC
TGGATCGAACAACAGCT

AtBON1 DNA 2

>ABC_NH9_CONTIG_243_p1   19571 pairs of NGS reads, 22.79%
AACGAGAGATAGATGATAGTGAAGGAACCTCTATCTGCGAGAAGAGTCCGTTGAAACCTTTGGAC
TTGAGATAGTAATCAAGGGCGTCGTTAGTcgcaccgagagcggcggaggaaccacttccaccaac
gccggctgtagcaccagcacCGGAGGCGACATCGGAGCAACAATTCCCCATAATTTTTGATTTCC
CAACAAAAAAAACTGCGGATTCAAAAGTACCTGGATCGAACAACAGCT
>ABC_NH9_CONTIG_243_p2   15390 pairs of NGS reads, 17.92%
AACGAGAGATAGATGATAGTGAAGGAACCTCTATCTGCGAGAAGAGTCCGTTGAAACCTTTGGAC
TTGAGATAGTAATCAAGGGCGTCGTTAGTCGCAccgagagcggcggaggaaccacttccaccaac
gccggctgtagcaccagcacCGGAGGCGACATCGGAGCAACAATTCCCCATAATTTTTGATTTCC
CAACAAAAAAAACTGCGGATTCAAAAGTACCTGGATCGAACAACCGAT
>ABC_NH9_CONTIG_243_p3   14104 pairs of NGS reads, 16.42%
AACGAGAGATAGATGATAGTGAAGGAACCTCTATCTGCGAGAAGAGTCCGTTGAAACCTTTGGAC
TTGAGATAGTAATCAAGGGCGTCGTTAGTCGCAccgagagcggcggaggaaccacttccaccaac
gccggctgtagcaccagcacCGGAGGCGACATCGGAGCAACAATTCCCCATAATTTTTGATTTCC
CAACAAAAAAAACTGCGGATTCAAAAGTACCTGGATCGAACAACGCAT
>ABC_NH9_CONTIG_213_p4   6241 pairs of NGS reads, 7.27%
ACGAGAGATAGATGATAGTGAAGGAACCTCTATCTGCGAGAAGAGTCCGTTGAAACCTTTGGACT
TGagatagtaatcaagggcgtcgttagtcgcaccgaccggctgtagcaccagcaccggaggcgac
atcggagcaacaattcCCCATAATTTTTGATTTCCCAACAAAAAAAACTGCGGATTCAAAAGTAC
CTGGATCGAACAACAGCT
>ABC_NH9_CONTIG_213_p5   6254 pairs of NGS reads, 7.28%
ACGAGAGATAGATGATAGTGAAGGAACCTCTATCTGCGAGAAGAGTCCGTTGAAACCTTTGGACT
TGAGatagtaatcaagggcgtcgttagtcgcaccgaccggctgtagcaccagcaccggaggcgac
atcggagcaacaattcCCCATAATTTTTGATTTCCCAACAAAAAAAACTGCGGATTCAAAAGTAC
CTGGATCGAACAACGCAT
>ABC_NH9_CONTIG_213_p6   5730 pairs of NGS reads, 6.67%
ACGAGAGATAGATGATAGTGAAGGAACCTCTATCTGCGAGAAGAGTCCGTTGAAACCTTTGGACT
TGAgatagtaatcaagggcgtcgttagtcgcaccgaccggctgtagcaccagcaccggaggcgac
atcggagcaacaattcCCCATAATTTTTGATTTCCCAACAAAAAAAACTGCGGATTCAAAAGTAC
CTGGATCGAACAACCGAT
>ABC_NH9_CONTIG_166_p7   3906 pairs of NGS reads, 4.55%
ACGAGAGATAGATGATAGTGaaggaacaagatgttataccttctcaagctcctcaagaacagact
tggcaacttcaccctttttgttcaaaaagagcatcaagcgtcatcattgggaccaaagccctaacc
actatgattcaaaagtACCTGGATCGAACAACAGCT
>ABC_NH9_CONTIG_166_p8   3208 pairs of NGS reads, 3.73%
ACGAGAGATAGATGATAGTGAAggaacaagatgttataccttctcaagctcctcaagaacagact
tggcaacttcaccctttttgttcaaaaagagcatcaagcgtcatcattgggaccaaagccctaacc
actatgattcaaaagtACCTGGATCGAACAACGCAT
>ABC_NH9_CONTIG_166_p9   2881 pairs of NGS reads, 3.35%
ACGAGAGATAGATGATAGTGAaggaacaagatgttataccttctcaagctcctcaagaacagact
tggcaacttcaccctttttgttcaaaaagagcatcaagcgtcatcattgggaccaaagccctaacc
actatgattcaaaagtACCTGGATCGAACAACCGAT

>ABC_NH9_CONTIG_212_p10   303 pairs of NGS reads, 0.35%
ACGAGAGATAGATGATAGTGAAGGAACCTCTATCTGCGAGAAGAGTCCGTTGAAACCTTTGGACT
TGAGatagtaatcaagggcgtcgttagtcgcaccgccggctgtagcaccagcaccggaggcgaca
tcggagcaacaatTCCCCATAATTTTTGATTTCCCAACAAAAAAAACTGCGGATTCAAAAGTACC
TGGATCGAACAACGCAT
>ABC_NH9_CONTIG_212_p11   298 pairs of NGS reads, 0.34%
ACGAGAGATAGATGATAGTGAAGGAACCTCTATCTGCGAGAAGAGTCCGTTGAAACCTTTGGACT
TGAGAtagtaatcaagggcgtcgttagtcgcaccgccggctgtagcaccagcaccggaggcgaca
tcggagcaacaatTCCCCATAATTTTTGATTTCCCAACAAAAAAAACTGCGGATTCAAAAGTACC
TGGATCGAACAACAGCT
>ABC_NH9_CONTIG_212_p12   281 pairs of NGS reads, 0.32%
ACGAGAGATAGATGATAGTGAAGGAACCTCTATCTGCGAGAAGAGTCCGTTGAAACCTTTGGACT
TGAGAtagtaatcaagggcgtcgttagtcgcaccgccggctgtagcaccagcaccggaggcgaca
tcggagcaacaatTCCCCATAATTTTTGATTTCCCAACAAAAAAAACTGCGGATTCAAAAGTACC
TGGATCGAACAACCGAT
>ABC_NH9_CONTIG_212_p13   185 pairs of NGS reads, 0.21%
ACGAGAGATAGATGATAGTGAAGGAACCTCTATCTGCGAGAAGAGTCCGTTGAAACCTTTGGACT
TGAGAtagtaatcaagggcgtcgttagtcgcaccgacggctgtagcaccagcaccggaggcgaca
tcggagcaacaaTTCCCCATAATTTTTGATTTCCCAACAAAAAAAACTGCGGATTCAAAAGTACC
TGGATCGAACAACAGCT
>ABC_NH9_CONTIG_243_p14   144 pairs of NGS reads, 0.16%
ACGAGAGATAGATGATAGTGAAGGAACCTCTATCTGCGAGAAGAGTCCGTTGAAACCTTTGGACT
TGAGATAGTAATCAAGGGCGTCGTTAGTCGCACccgagagcggcggaggaaccacttccaccaac
gccggctgtagcACCAGCACCGGAGGCGACATCGGAGCAACAATTCCCCATAATTTTTGATTTCC
CAACAAAAAAAACTGCGGATTCAAAAGTACCTGGATCGAACAACGCAT
>ABC_NH9_CONTIG_243_p15   135 pairs of NGS reads, 0.15%
ACGAGAGATAGATGATAGTGAAGGAACCTCTATCTGCGAGAAGAGTCCGTTGAAACCTTTGGACT
TGAGATAGTAATCAAGGGCGTCGTTAGTCGCACccgagagcggcggaggaaccacttccaccaac
gccggctgtagcACCAGCACCGGAGGCGACATCGGAGCAACAATTCCCCATAATTTTTGATTTCC
CAACAAAAAAAACTGCGGATTCAAAAGTACCTGGATCGAACAACCGAT
>ABC_NH9_CONTIG_212_p16   131 pairs of NGS reads, 0.15%
ACGAGAGATAGATGATAGTGAAGGAACCTCTATCTGCGAGAAGAGTCCGTTGAAACCTTTGGACT
TGAGAtagtaatcaagggcgtcgttagtcgcaccgacggctgtagcaccagcaccggaggcgaca
tcggagcaacaaTTCCCCATAATTTTTGATTTCCCAACAAAAAAAACTGCGGATTCAAAAGTACC
TGGATCGAACAACGCAT
>ABC_NH9_CONTIG_212_p17   131 pairs of NGS reads, 0.15%
ACGAGAGATAGATGATAGTGAAGGAACCTCTATCTGCGAGAAGAGTCCGTTGAAACCTTTGGACT
TGAGAtagtaatcaagggcgtcgttagtcgcaccgacggctgtagcaccagcaccggaggcgaca
tcggagcaacaaTTCCCCATAATTTTTGATTTCCCAACAAAAAAAACTGCGGATTCAAAAGTACC
TGGATCGAACAACCGAT
>ABC_NH9_CONTIG_243_p18   126 pairs of NGS reads, 0.14%
ACGAGAGATAGATGATAGTGAAGGAACCTCTATCTGCGAGAAGAGTCCGTTGAAACCTTTGGACT
TGAGATAGTAATCAAGGGCGTCGTTAGTCGCACCGAagagcggcggaggaaccacttccaccaac
gccggctgtagcACCAGCACCGGAGGCGACATCGGAGCAACAATTCCCCATAATTTTTGATTTCC
CAACAAAAAAAACTGCGGATTCAAAAGTACCTGGATCGAACAACAGCT
>ABC_NH9_CONTIG_244_p19   117 pairs of NGS reads, 0.13%
AACGAGAGATAGATGATAGTGAAGGAACCTCTATCTGCGAGAAGAGTCCGTTGAAACCTTTGGAC
TTGAGATAGTAATCAAGGGCGTCGTTAGTCGCACCGAgagcggcggaggaaccacttccaccaac
gccggctgtagcaccagcacCGGAGGCGACATCGGAGCAACAATTCCCCATAATTTTTGATTTCC
CAACAAAAAAAAACTGCGGATTCAAAAGTACCTGGATCGAACAACAGCT
>ABC_NH9_CONTIG_243_p20   114 pairs of NGS reads, 0.13%
ACGAGAGATAGATGATAGTGAAGGAACCTCTATCTGCGAGAAGAGTCCGTTGAAACCTTTGGACT

TGAGATAGTAATCAAGGGCGTCGTTAGTCGCACCGAgagcggcggaggaaccacttccaccaacg
accggctgtagcACCAGCACCGGAGGCGACATCGGAGCAACAATTCCCCATAATTTTTGATTTCC
CAACAAAAAAAAACTGCGGATTCAAAAGTACCTGGATCGAACAACCGAT
>ABC_NH9_CONTIG_244_p21   107 pairs of NGS reads, 0.12%
AACGAGAGATAGATGATAGTGAAGGAACCTCTATCTGCGAGAAGAGTCCGTTGAAACCTTTGGAC
TTGAGATAGTAATCAAGGGCGTCGTTAGTCGCACCGAgagcggcggaggaaccacttccaccaac
gccggctgtagcaccagcacCGGAGGCGACATCGGAGCAACAATTCCCCATAATTTTTGATTTCC
CAACAAAAAAAAAACTGCGGATTCAAAAGTACCTGGATCGAACAACCGAT
>ABC_NH9_CONTIG_242_p22   102 pairs of NGS reads, 0.11%
ACGAGAGATAGATGATAGTGAAGGAACCTCTATCTGCGAGAAGAGTCCGTTGAAACCTTTGGACT
TGAGATAGTAATCAAGGGCGTCGTTAGTCGCACCGagagcggcggaggaaacacttccaccaacg
ccggctgtagcaCCAGCACCGGAGGCGACATCGGAGCAACAATTCCCCATAATTTTTGATTTCCC
AACAAAAAAAAACTGCGGATTCAAAAGTACCTGGATCGAACAACAGCT
>ABC_NH9_CONTIG_243_p23   101 pairs of NGS reads, 0.11%
ACGAGAGATAGATGATAGTGAAGGAACCTCTATCTGCGAGAAGAGTCCGTTGAAACCTTTGGACT
TGAGATAGTAATCAAGGGCGTCGTTAGTCGCACCGAgagcggcggaggaaccacttccaccaacg
tccggctgtagcACCAGCACCGGAGGCGACATCGGAGCAACAATTCCCCATAATTTTTGATTTCC
CAACAAAAAAAAACTGCGGATTCAAAAGTACCTGGATCGAACAACGCAT
>ABC_NH9_CONTIG_252_p24   100 pairs of NGS reads, 0.11%
ACGAGAGATAGATGATAGTGAAGGAACTCTGTAGTTTTTGGGGTTAACTCGTATTTGTAAGGCTA
GTTTATTATTCATCTCGTTGCTTGAAGCAAACCGATGCATAAGTGtttttttgttgttgggaccat
gcatggttctaaCAATCTATGCACTGGTTAAAGCATGTTCTAACACAATTAGAACTGTGTTACAA
AATCAATAGGACAAACATCACATTTGCATTCAAAAGTACCTGGATCGAACAACGCAT
>ABC_NH9_CONTIG_125_p25   91 pairs of NGS reads, 0.1%
acgagagatagatgatagtgaaggaaccgagaaagtaagccaagcggcgtagaccaataatcaat
gtagactttgacacctaacaattgcactagattcaaaagtacctggatcgaacaacgcat
>ABC_NH9_CONTIG_244_p26   88 pairs of NGS reads, 0.1%
AACGAGAGATAGATGATAGTGAAGGAACCTCTATCTGCGAGAAGAGTCCGTTGAAACCTTTGGAC
TTGAGATAGTAATCAAGGGCGTCGTTAGTCGCACCGAgagcggcggaggaaccacttccaccaac
gccggctgtagcaccagcacCGGAGGCGACATCGGAGCAACAATTCCCCATAATTTTTGATTTCC
CAACAAAAAAAAAACTGCGGATTCAAAAGTACCTGGATCGAACAACGCAT

AtBON1 DNA 3

>1_AF12_CONTIG_189_p1   58926 pairs of NGS reads, 73.53%
GAATCCGCAGTTTTTTTTGTTGGGAAATCAAAAATTATGGGGAATtgttgctccgatgtcgcctc
cggtgctggtgctacagccggcgttggtggaagtggttcctccgccgctctcggtgcgactaacg
acgcccttgattacTATCTCAAGTCCAAAGGTTTCAACGGACTCTTCTCGCAGATAGAG
>1_AF12_CONTIG_160_p2   11027 pairs of NGS reads, 13.76%
GAATCCGCAGTTTTTTTTtgttgggaaatcaaaaattatggggaattgttgctccgatgtcgcctc
cggtgctggtgctacagccggtcggtgcgactaacgacgcccttgattactatctcaagtccaaa
ggtttcaacggaCTCTTCTCGCAGATAGAG
>1_AF12_CONTIG_190_p3   2794 pairs of NGS reads, 3.48%
GAATCCGCAGTTTTTTTTGTTGGGAAATCAAAAATTATGGGGAATTGttgctccgatgtcgcctc
cggtgctggtgctacagccggcgttggtggaagtggttcctccgccgctcttcggtgcgactaac
gacgcccttgatTACTATCTCAAGTCCAAAGGTTTCAACGGACTCTTCTCGCAGATAGAG
>1_AF12_CONTIG_198_p4   1317 pairs of NGS reads, 1.64%
GAATCCGCAGTTTTTTTTGTTGGCCGAATCGGACCTGATCCTGGTACTGCTGTCGctgatcgaca
tggcgctggtgggcggcctgctggtgatggtgatgatctccggctacgaaaacttcgtctcggaa
ctgaacatcgacGAGGGTAAAGAGAAGCTCAGCTGGCTGGGCAAGATGGACTCTTCTCGCAGATA
GAG

>1_AF12_CONTIG_190_p5   892 pairs of NGS reads, 1.11%
GAATCCGCAGTTTTTTTTGTTGGGAAATCAAAAATTATGGGGAATTGTtgctccgatgtcgcctc
cggtgctggtgctacagccggcgttggtggaagtggttcctccgccgctcatcggtgcgactaac
gacgcccttgatTACTATCTCAAGTCCAAAGGTTTCAACGGACTCTTCTCGCAGATAGAG

AtBON RNP 1

>17C_BF11_CONTIG_224_p1   1898 pairs of NGS reads, 16.63%
AAAAACGAGAGATAGATGATAGTGAAGGAACCTCTATCTGAGAGAAGAGTCCGTTGAAACCTTTG
GACTTGAGATagtaatcaagggcgtcgttagtcgcaccgaccggctgtagcaccagcaccggagg
cgacatcggagcaacaaTTCCCCATAATTTTTGATTTCCCAACAAAAAAAACTGCGGATTCAAAA
GTACCTGGATCGAACAACGAGCTCAAAAA
>17C_BF11_CONTIG_224_p2   1559 pairs of NGS reads, 13.66%
TTTTACGAGAGATAGATGATAGTGAAGGAACCTCTATCTGAGAGAAGAGTCCGTTGAAACCTTTG
GACTTGAGATagtaatcaagggcgtcgttagtcgcaccgaccggctgtagcaccagcaccggagg
cgacatcggagcaacAATTCCCCATAATTTTTGATTTCCCAACAAAAAAAACTGCGGATTCAAAA
GTACCTGGATCGAACAACGAGCTCAAAAA
>17C_BF11_CONTIG_253_p3   733 pairs of NGS reads, 6.42%
CCCCACGAGAGATAGATGATAGTGAAGGAACCTCTATCTGAGAGAAGAGTCCGTTGAAACCTTTG
GACTTGAGATAGTAATCAAGGGCGTCGTTAGTCGCACCGAGAGCggcggaggaagcacttccacc
aacgccggctgtAGCACCAGCACCGGAGGCGACATCGGAGCAACAATTCCCCATAATTTTTGATT
TCCCAACAAAAAAAACTGCGGATTCAAAAGTACCTGGATCGAACAACGAGCTCAAAAA
>17C_BF11_CONTIG_253_p4   520 pairs of NGS reads, 4.55%
GGGGACGAGAGATAGATGATAGTGAAGGAACCTCTATCTGAGAGAAGAGTCCGTTGAAACCTTTG
GACTTGAGATAGTAATCAAGGGCGTCGTTAGTCGCACCGAGAGCggcggaggaagcacttccacc
aacgccggctgtAGCACCAGCACCGGAGGCGACATCGGAGCAACAATTCCCCATAATTTTTGATT
TCCCAACAAAAAAAACTGCGGATTCAAAAGTACCTGGATCGAACAACGAGCTCAAAAA
>17C_BF11_CONTIG_246_p5   1237 pairs of NGS reads, 10.84%
TTTTACGAGAGATAGATGATAGTGAAGGAACCTCTATCTGAGAGAAGAGTCCGTTGAAACCTTTG
GACTTGAGATAGTAATCAAGGGCGTCGTTAGTCGCACCgagagcggcggaggaagcacttccacc
aacgccggctgtagcaccAGCACCGGAGGCGACATCGGAGCAACAATTCCCCATAATTTTTGATT
TCCCAACAAAAAAAACTGCGGATTCAAAAGTACCTGGATCGAACAACTTTT
>17C_BF11_CONTIG_253_p6   358 pairs of NGS reads, 3.13%
AAAAACGAGAGATAGATGATAGTGAAGGAACCTCTATCTGAGAGAAGAGTCCGTTGAAACCTTTG
GACTTGAGATAGTAATCAAGGGCGTCGTTAGTCGCACCGAGAGCggcggaggaagcacttccacc
aacgccggctgtAGCACCAGCACCGGAGGCGACATCGGAGCAACAATTCCCCATAATTTTTGATT
TCCCAACAAAAAAAACTGCGGATTCAAAAGTACCTGGATCGAACAACGAGCTCAAAAA
>17C_BF11_CONTIG_246_p7   466 pairs of NGS reads, 4.08%
TTTTACGAGAGATAGATGATAGTGAAGGAACCTCTATCTGAGAGAAGAGTCCGTTGAAACCTTTG
GACTTGAGATAGTAATCAAGGGCGTCGTTAGTCGCACCgagagcggcggaggaagcacttccacc
aacgccggctggaGCACCAGCACCGGAGGCGACATCGGAGCAACAATTCCCCATAATTTTTGATT
TCCCAACAAAAAAAACTGCGGATTCAAAAGTACCTGGATCGAACAACTTTT
>17C_BF11_CONTIG_252_p8   294 pairs of NGS reads, 2.57%
CCCCACGAGAGATAGATGATAGTGAAGGAACCTCTATCTGAGAGAAGAGTCCGTTGAAACCTTTG
GACTTGAGATAGTAATCAAGGGCGTCGTTAGTCGCACCGAGAGCGgcggaggaacctcatccacc
aacgccggctgtAGCACCAGCACCGGAGGCGACATCGGAGCAACAATTCCCCATAATTTTTGATT
TCCCAACAAAAAAAACTGCGGATTCAAAAGTACCTGGATCGAACAACGAGCTCAAAA
>17C_BF11_CONTIG_291_p9   131 pairs of NGS reads, 1.14%
CCCCACGAGAGATAGATGATAGTGAAGGAACCTCTATCTGAGAGAAGAGTCCGTTGAAACCTTTG
GACTTGAGATAGTAATCAAGGGCGTCGTTAGTCGCACCGAGAGCGGCGGAGGAAGCACTTCCACC

AACGCCGGCCGGNNNNNGGCGGAGGAAGCACTTCCACCAACGCCGGCTGTAGCACCAGCACCGGA
GGCGACATCGGAGCAACAATTCCCCATAATTTTTGATTTCCCAACAAAAAAAACTGCGGATTCAA
AAGTACCTGGATCGAACAACGAGCTCAAAAA
>17C_BF11_CONTIG_246_p10   118 pairs of NGS reads, 1.03%
TTTTACGAGAGATAGATGATAGTGAAGGAACCTCTATCTGAGAGAAGAGTCCGTTGAAACCTTTG
GACTTGAGATAGTAATCAAGGGCGTCGTTAGTCGCACCgagagcggcggaggaagcacttccacc
aacgccggccggaGCACCAGCACCGGAGGCGACATCGGAGCAACAATTCCCCATAATTTTTGATT
TCCCAACAAAAAAAACTGCGGATTCAAAAGTACCTGGATCGAACAACTTTT
>17C_BF11_CONTIG_246_p11   179 pairs of NGS reads, 1.56%
TTTTACGAGAGATAGATGATAGTGAAGGAACCTCTATCTGAGAGAAGAGTCCGTTGAAACCTTTG
GACTTGAGATAGTAATCAAGGGCGTCGTTAGTCGCACCgagagcggcggaggaagcacttccacc
aacgccggccgtaGCACCAGCACCGGAGGCGACATCGGAGCAACAATTCCCCATAATTTTTGATT
TCCCAACAAAAAAAACTGCGGATTCAAAAGTACCTGGATCGAACAACTTTT
>17C_BF11_CONTIG_291_p12   105 pairs of NGS reads, 0.92%
GGGGACGAGAGATAGATGATAGTGAAGGAACCTCTATCTGAGAGAAGAGTCCGTTGAAACCTTTG
GACTTGAGATAGTAATCAAGGGCGTCGTTAGTCGCACCGAGAGCGGCGGAGGAAGCACTTCCACC
AACGCCGGCCGGNNNNNGGCGGAGGAAGCACTTCCACCAACGCCGGCTGTAGCACCAGCACCGGA
GGCGACATCGGAGCAACAATTCCCCATAATTTTTGATTTCCCAACAAAAAAAACTGCGGATTCAA
AAGTACCTGGATCGAACAACGAGCTCAAAAA
>17C_BF11_CONTIG_222_p13   87 pairs of NGS reads, 0.76%
AAAAACGAGAGATAGATGATAGTGAAGGAACCTCTATCTGAGAGAAGAGTCCGTTGAAACCTTTG
GACTTGAGATAGTAAtcaagggcgtcgttagtcgcaccgacggctgtagcaccagcaccggaggc
gacatcggagcaACAATTCCCCATAATTTTTGATTTCCCAACAAAAAAAACTGCGGATTCAAAAG
TACCTGGATCGAACAACGAGCTCAAAA
>17C_BF11_CONTIG_222_p14   83 pairs of NGS reads, 0.72%
AAAAACGAGAGATAGATGATAGTGAAGGAACCTCTATCTGAGAGAAGAGTCCGTTGAAACCTTTG
GACTTGAGATAGTAAtcaagggcgtcgttagtcgcaccgccggctgtagcaccagcaccggaggc
gacatcggagcaACAATTCCCCATAATTTTTGATTTCCCAACAAAAAAAACTGCGGATTCAAAAG
TACCTGGATCGAACAACGAGCTCAAAA
>17C_BF11_CONTIG_290_p15   78 pairs of NGS reads, 0.68%
TTTTACGAGAGATAGATGATAGTGAAGGAACCTCTATCTGAGAGAAGAGTCCGTTGAAACCTTTG
GACTTGAGATAGTAATCAAGGGCGTCGTTAGTCGCACCGAGAGCGGCGGAGGAAGCACTTCCACC
AACGCCGGCCGGANNNNNGGAGCGGCGGAGGAAGCACTTCCACCAACGCCGGCTGTAGCACCAGC
ACCGGAGGCGACATCGGAGCAACAATTCCCCATAATTTTTGATTTCCCAACAAAAAAAACTGCGG
ATTCAAAAGTACCTGGATCGAACAACTTTT
>17C_BF11_CONTIG_252_p16   78 pairs of NGS reads, 0.68%
GGGGACGAGAGATAGATGATAGTGAAGGAACCTCTATCTGAGAGAAGAGTCCGTTGAAACCTTTG
GACTTGAGATAGTAATCAAGGGCGTCGTTAGTCGCACCGAGAGCGgcggaggaacctcatccacc
aacgccggctgtAGCACCAGCACCGGAGGCGACATCGGAGCAACAATTCCCCATAATTTTTGATT
TCCCAACAAAAAAAACTGCGGATTCAAAAGTACCTGGATCGAACAACGAGCTCAAAA
>17C_BF11_CONTIG_170_p17   67 pairs of NGS reads, 0.58%
AAAAGTTGTTCGATCCAGGTACTTTTGaatcatagtggttagggctttggtcccaatgatgacgc
ttgatgctcttttgaacaaaagggtgaagttgccaagtctgttcttgaggagcttgagaaggta
taacatcttgttCCTTCACTATCATCTATCTCTCGTAAAA
>17C_BF11_CONTIG_253_p18   145 pairs of NGS reads, 1.27%
TTTTACGAGAGATAGATGATAGTGAAGGAACCTCTATCTGAGAGAAGAGTCCGTTGAAACCTTTG
GACTTGAGATAGTAATCAAGGGCGTCGTTAGTCGCACCGAGAGCggcggaggaagcacttccacc
aacgccggctgtaGCACCAGCACCGGAGGCGACATCGGAGCAACAATTCCCCATAATTTTTGATT
TCCCAACAAAAAAAACTGCGGATTCAAAAGTACCTGGATCGAACAACGAGCTCAAAAA
>17C_BF11_CONTIG_224_p19   55 pairs of NGS reads, 0.48%
GGGGACGAGAGATAGATGATAGTGAAGGAACCTCTATCTGAGAGAAGAGTCCGTTGAAACCTTTG
GACTTGAGATagtaatcaagggcgtcgttagtcgcaccgaccggctgtagcaccagcaccggagg

cgacatcggagcAACAATTCCCCATAATTTTTGATTTCCCAACAAAAAAAACTGCGGATTCAAAA
GTACCTGGATCGAACAACGAGCTCAAAAA
>17C_BF11_CONTIG_223_p20   50 pairs of NGS reads, 0.43%
AAAAACGAGAGATAGATGATAGTGAAGGAACCTCTATCTGAGAGAAGAGTCCGTTGAAACCTTTG
GACTTGAGATAGTAATaaagggcgtcgttagtcgcaccgaccggctgtagcaccagcaccggagg
cgacatcggagcaacaaTTCCCCATAATTTTTGATTTCCCAACAAAAAAAACTGCGGATTCAAAA
GTACCTGGATCGAACAACGAGCTCAAAA
>17C_BF11_CONTIG_291_p21   50 pairs of NGS reads, 0.43%
AAAAACGAGAGATAGATGATAGTGAAGGAACCTCTATCTGAGAGAAGAGTCCGTTGAAACCTTTG
GACTTGAGATAGTAATCAAGGGCGTCGTTAGTCGCACCGAGAGCGGCGGAGGAAGCACTTCCACC
AACGCCGGCCGGNNNNNGGCGGAGGAAGCACTTCCACCAACGCCGGCTGTAGCACCAGCACCGGA
GGCGACATCGGAGCAACAATTCCCCATAATTTTTGATTTCCCAACAAAAAAAACTGCGGATTCAA
AAGTACCTGGATCGAACAACGAGCTCAAAAA
>17C_BF11_CONTIG_289_p22   40 pairs of NGS reads, 0.35%
CCCCACGAGAGATAGATGATAGTGAAGGAACCTCTATCTGAGAGAAGAGTCCGTTGAAACCTTTG
GACTTGAGATAGTAATCAAGGGCGTCGTTAGTCGCACCGAGAGCGGCGGAGGAACCTCATCCACC
AACGCCGGCCGGNNNNNGCGGAGGAACCTCATCCACCAACGCCGGCTGTAGCACCAGCACCGGAG
GCGACATCGGAGCAACAATTCCCCATAATTTTTGATTTCCCAACAAAAAAAACTGCGGATTCAAA
AGTACCTGGATCGAACAACGAGCTCAAAA
>17C_BF11_CONTIG_221_p23   38 pairs of NGS reads, 0.33%
AAAAACGAGAGATAGATGATAGTGAAGGAACCTCTATCTGAGAGAAGAGTCCGTTGAAACCTTTG
GACTTGAGATAGTAatcaagggcgtcgttagtcgcaccgaggctgtagcaccagcaccggaggcg
acatcggagcaaCAATTCCCCATAATTTTTGATTTCCCAACAAAAAAAACTGCGGATTCAAAAGT
ACCTGGATCGAACAACGAGCTCAAAA
>17C_BF11_CONTIG_223_p24   38 pairs of NGS reads, 0.33%
TTTTACGAGAGATAGATGATAGTGAAGGAACCTCTATCTGAGAGAAGAGTCCGTTGAAACCTTTG
GACTTGAGATAGTAATaaagggcgtcgttagtcgcaccgaccggctgtagcaccagcaccggagg
cgacatcggagcaacAATTCCCCATAATTTTTGATTTCCCAACAAAAAAAACTGCGGATTCAAAA
GTACCTGGATCGAACAACGAGCTCAAAA
>17C_BF11_CONTIG_246_p25   36 pairs of NGS reads, 0.31%
TTTTACGAGAGATAGATGATAGTGAAGGAACCTCTATCTGAGAGAAGAGTCCGTTGAAACCTTTG
GACTTGAGATAGTAATCAAGGGCGTCGTTAGTCGCACCgagagcggcggaggaagcacttccccc
aacgccggctgtaGCACCAGCACCGGAGGCGACATCGGAGCAACAATTCCCCATAATTTTTGATT
TCCCAACAAAAAAAACTGCGGATTCAAAAGTACCTGGATCGAACAACTTTT
>17C_BF11_CONTIG_224_p26   36 pairs of NGS reads, 0.31%
CCCCACGAGAGATAGATGATAGTGAAGGAACCTCTATCTGAGAGAAGAGTCCGTTGAAACCTTTG
GACTTGAGATagtaatcaagggcgtcgttagtcgcaccgaccggctgtagcaccagcaccggagg
cgacatcggagcAACAATTCCCCATAATTTTTGATTTCCCAACAAAAAAAACTGCGGATTCAAAA
GTACCTGGATCGAACAACGAGCTCAAAAA
>17C_BF11_CONTIG_290_p27   35 pairs of NGS reads, 0.3%
TTTTACGAGAGATAGATGATAGTGAAGGAACCTCTATCTGAGAGAAGAGTCCGTTGAAACCTTTG
GACTTGAGATAGTAATCAAGGGCGTCGTTAGTCGCACCGAGAGCGGCGGAGGAAGCACTTCCACC
AACGCCCGCCGGNNNNNGAGAGCGGCGGAGGAAGCACTTCCACCAACGCCGGCTGTAGCACCAGC
ACCGGAGGCGACATCGGAGCAACAATTCCCCATAATTTTTGATTTCCCAACAAAAAAAACTGCGG
ATTCAAAAGTACCTGGATCGAACAACTTTT
>17C_BF11_CONTIG_246_p28   34 pairs of NGS reads, 0.29%
TTTTACGAGAGATAGATGATAGTGAAGGAACCTCTATCTGAGAGAAGAGTCCGTTGAAACCTTTG
GACTTGAGATAGTAATCAAGGGCGTCGTTAGTCGCACCGagagcggcggaggaagcacttccacc
aacgccggctgtagcaccAGCACCGGAGGCGACATCGGAGCAACAATTCCCCATAATTTTTGATT
TCCCAACAAAAAAAATTGCGGATTCAAAAGTACCTGGATCGAACAACTTTT
>17C_BF11_CONTIG_220_p29   33 pairs of NGS reads, 0.28%
AAAAACGAGAGATAGATGATAGTGAAGGAACCTCTATCTGAGAGAAGAGTCCGTTGAAACCTTTG

GACTTGAGATAGTaatcaagggcgtcgttagtcgcacccggctgtagcaccagcaccggaggcga
catcggagcaacAATTCCCCATAATTTTTGATTTCCCAACAAAAAAAACTGCGGATTCAAAAGTA
CCTGGATCGAACAACGAGCTCAAAA
>17C_BF11_CONTIG_291_p30   32 pairs of NGS reads, 0.28%
CCCCACGAGAGATAGATGATAGTGAAGGAACCTCTATCTGAGAGAAGAGTCCGTTGAAACCTTTG
GACTTGAGATAGTAATCAAGGGCGTCGTTAGTCGCACCGAGAGCGGCGGAGGAAGCACCTCCACC
AACGCCGGCCGGNNNNNGGCGGAGGAAGCACTTCCACCAACGCCGGCTGTAGCACCAGCACCGGA
GGCGACATCGGAGCAACAATTCCCCATAATTTTTGATTTCCCAACAAAAAAAACTGCGGATTCAA
AAGTACCTGGATCGAACAACGAGCTCAAAAA
>17C_BF11_CONTIG_222_p31   30 pairs of NGS reads, 0.26%
TTTTACGAGAGATAGATGATAGTGAAGGAACCTCTATCTGAGAGAAGAGTCCGTTGAAACCTTTG
GACTTGAGATAGTAAtcaagggcgtcgttagtcgcaccgccggctgtagcaccagcaccggaggc
gacatcggagcaACAATTCCCCATAATTTTTGATTTCCCAACAAAAAAAACTGCGGATTCAAAAG
TACCTGGATCGAACAACGAGCTCAAAA
>17C_BF11_CONTIG_223_p32   28 pairs of NGS reads, 0.24%
AAAAACGAGAGATAGATGATAGTGAAGGAACCTCTATCTGAGAGAAGAGTCCGTTGAAACCTTTG
GACTTGAGATAGTAATcaaggggtcgttagtcgcaccgaccggctgtagcaccagcaccggagg
cgacatcggagcaacaaTTCCCCATAATTTTTGATTTCCCAACAAAAAAAACTGCGGATTCAAAA
GTACCTGGATCGAACAACGAGCTCAAAA
>17C_BF11_CONTIG_246_p33   28 pairs of NGS reads, 0.24%
TTTTACGAGAGATAGATGATAGTGAAGGAACCTCTATCTGAGAGAAGAGTCCGTTGAAACCTTTG
GACTTGAGATAGTAATCAAGGGCGTCGTTAGTCGCACCgagagcggcggaggaagcacctccacc
aacgccggctggAGCACCAGCACCGGAGGCGACATCGGAGCAACAATTCCCCATAATTTTTGATT
TCCCAACAAAAAAAACTGCGGATTCAAAAGTACCTGGATCGAACAACTTTT
>17C_BF11_CONTIG_246_p34   27 pairs of NGS reads, 0.23%
TTTTACGAGAGATAGATGATAGTGAAGGAACCTCTATCTGAGAGAAGAGTCCGTTGAAACCTTTG
GACTTGAGATAGTAATCAAGGGCGTCGTTAGTCGCACCgagagcggcggagggagcacttccacc
aacgccggctgtAGCACCAGCACCGGAGGCGACATCGGAGCAACAATTCCCCATAATTTTTGATT
TCCCAACAAAAAAAACTGCGGATTCAAAAGTACCTGGATCGAACAACTTTT
>17C_BF11_CONTIG_215_p35   27 pairs of NGS reads, 0.23%
TTTTACGAGAGATAGATGATAGTGAAGGAACCTCTATCTGAGAGAAGAGTCCGTTGAAACCTTTG
GACTTGAGatagtaatcaagggcgtcgttagtccggctgtagcaccagcaccggaggcgacatcg
gagcaacaattcCCCATAATTTTTGATTTCCCAACAAAAAAAACTGCGGATTCAAAAGTACCTGG
ATCGAACAACGAGCTCAAAA
>17C_BF11_CONTIG_222_p36   27 pairs of NGS reads, 0.23%
TTTTACGAGAGATAGATGATAGTGAAGGAACCTCTATCTGAGAGAAGAGTCCGTTGAAACCTTTG
GACTTGAGATAGTAAtcaagggcgtcgttagtcgcaccgacggctgtagcaccagcaccggaggc
gacatcggagcaACAATTCCCCATAATTTTTGATTTCCCAACAAAAAAAACTGCGGATTCAAAAG
TACCTGGATCGAACAACGAGCTCAAAA
>17C_BF11_CONTIG_219_p37   27 pairs of NGS reads, 0.23%
AAAAACGAGAGATAGATGATAGTGAAGGAACCTCTATCTGAGAGAAGAGTCCGTTGAAACCTTTG
GACTTGAGATAGtaatcaagggcgtcgttagtcgcaccggctgtagcaccagcaccggaggcgac
atcggagcaacaATTCCCCATAATTTTTGATTTCCCAACAAAAAAAACTGCGGATTCAAAAGTAC
CTGGATCGAACAACGAGCTCAAAA
>17C_BF11_CONTIG_291_p38   27 pairs of NGS reads, 0.23%
CCCCACGAGAGATAGATGATAGTGAAGGAACCTCTATCTGAGAGAAGAGTCCGTTGAAACCTTTG
GACTTGAGATAGTAATCAAGGGCGTCGTTAGTCGCACCGAGAGCGGCGGAGGAAACACTTCCACC
AACGCCGGCCGGNNNNNGGCGGAGGAAGCACTTCCACCAACGCCGGCTGTAGCACCAGCACCGGA
GGCGACATCGGAGCAACAATTCCCCATAATTTTTGATTTCCCAACAAAAAAAACTGCGGATTCAA
AAGTACCTGGATCGAACAACGAGCTCAAAAA
>17C_BF11_CONTIG_290_p39   26 pairs of NGS reads, 0.22%
TTTTACGAGAGATAGATGATAGTGAAGGAACCTCTATCTGAGAGAAGAGTCCGTTGAAACCTTTG

GACTTGAGATAGTAATCAAGGGCGTCGTTAGTCGCACCGAGAGCGGCGGAGGAAGCACCTCCACC
AACGCCGGCCGGNNNNNGAGAGCGGCGGAGGAAGCACTTCCACCAACGCCGGCTGTAGCACCAGC
ACCGGAGGCGACATCGGAGCAACAATTCCCCATAATTTTTGATTTCCCAACAAAAAAAACTGCGG
ATTCAAAAGTACCTGGATCGAACAACTTTT
>17C_BF11_CONTIG_246_p40   26 pairs of NGS reads, 0.22%
TTTTACGAGAGATAGATGATAGTGAAGGAACCTCTATCTGAGAGAAGAGTCCGTTGAAACCTTTG
GACTTGAGATAGTAATCAAGGGCGTCGTTAGTCGCACCgagagcggcggaggaagcacttccacc
aacgcccgctggAGCACCAGCACCGGAGGCGACATCGGAGCAACAATTCCCCATAATTTTTGATT
TCCCAACAAAAAAAACTGCGGATTCAAAAGTACCTGGATCGAACAACTTTT
>17C_BF11_CONTIG_252_p41   25 pairs of NGS reads, 0.21%
AAAAACGAGAGATAGATGATAGTGAAGGAACCTCTATCTGAGAGAAGAGTCCGTTGAAACCTTTG
GACTTGAGATAGTAATCAAGGGCGTCGTTAGTCGCACCGAGAGCGgcggaggaacctcatccacc
aacgccggctgtAGCACCAGCACCGGAGGCGACATCGGAGCAACAATTCCCCATAATTTTTGATT
TCCCAACAAAAAAAACTGCGGATTCAAAAGTACCTGGATCGAACAACGAGCTCAAAA
>17C_BF11_CONTIG_246_p42   25 pairs of NGS reads, 0.21%
TTTTACGAGAGATAGATGATAGTGAAGGAACCTCTATCTGAGAGAAGAGTCCGTTGAAACCTTTG
GACTTGAGATAGTAATCAAGGGCGTCGTTAGTCGCACCgagagcggcggaggaagcacctccacc
aacgccggctgtAGCACCAGCACCGGAGGCGACATCGGAGCAACAATTCCCCATAATTTTTGATT
TCCCAACAAAAAAAACTGCGGATTCAAAAGTACCTGGATCGAACAACTTTT
>17C_BF11_CONTIG_291_p43   25 pairs of NGS reads, 0.21%
GGGGACGAGAGATAGATGATAGTGAAGGAACCTCTATCTGAGAGAAGAGTCCGTTGAAACCTTTG
GACTTGAGATAGTAATCAAGGGCGTCGTTAGTCGCACCGAGAGCGGCGGAGGAAACACTTCCACC
AACGCCGGCTGGNNNNNGGCGGAGGAAGCACTTCCACCAACGCCGGCTGTAGCACCAGCACCGGA
GGCGACATCGGAGCAACAATTCCCCATAATTTTTGATTTCCCAACAAAAAAAACTGCGGATTCAA
AAGTACCTGGATCGAACAACGAGCTCAAAAA
>17C_BF11_CONTIG_291_p44   24 pairs of NGS reads, 0.21%
CCCCACGAGAGATAGATGATAGTGAAGGAACCTCTATCTGAGAGAAGAGTCCGTTGAAACCTTTG
GACTTGAGATAGTAATCAAGGGCGTCGTTAGTCGCACCGAGAGCGGCGGAGGAAACACTTCCACC
AACGCCGGCTGGNNNNNGGCGGAGGAAGCACTTCCACCAACGCCGGCTGTAGCACCAGCACCGGA
GGCGACATCGGAGCAACAATTCCCCATAATTTTTGATTTCCCAACAAAAAAAACTGCGGATTCAA
AAGTACCTGGATCGAACAACGAGCTCAAAAA
>17C_BF11_CONTIG_222_p45   23 pairs of NGS reads, 0.2%
AAAAACGAGAGATAGATGATAGTGAAGGAACCTCTATCTGAGAGAAGAGTCCGTTGAAACCTTTG
GACTTGAGATAGTAAccaagggcgtcgttagtcgcaccgccggctgtagcaccagcaccggaggc
gacatcggagcaACAATTCCCCATAATTTTTGATTTCCCAACAAAAAAAACTGCGGATTCAAAAG
TACCTGGATCGAACAACGAGCTCAAAA
>17C_BF11_CONTIG_291_p46   23 pairs of NGS reads, 0.2%
CCCCACGAGAGATAGATGATAGTGAAGGAACCTCTATCTGAGAGAAGAGTCCGTTGAAACCTTTG
GACTTGAGATAGTAATCAAGGGCGTCGTTAGTCGCACCGAGAGCGGCGGAGGAAGCACTTCCACC
CACGCCGGCCGGNNNNNGGCGGAGGAAGCACTTCCACCAACGCCGGCTGTAGCACCAGCACCGGA
GGCGACATCGGAGCAACAATTCCCCATAATTTTTGATTTCCCAACAAAAAAAACTGCGGATTCAA
AAGTACCTGGATCGAACAACGAGCTCAAAAA
>17C_BF11_CONTIG_289_p47   23 pairs of NGS reads, 0.2%
TTTTACGAGAGATAGATGATAGTGAAGGAACCTCTATCTGAGAGAAGAGTCCGTTGAAACCTTTG
GACTTGAGATAGTAATCAAGGGCGTCGTTAGTCGCACCGAGAGCGGCGGAGGAAACACTTCCACC
AACGCCGGCTGGNNNNNGGAGCGGCGGAGGAAGCACTTCCACCAACGCCGGCTGTAGCACCAGCA
CCGGAGGCGACATCGGAGCAACAATTCCCCATAATTTTTGATTTCCCAACAAAAAAAACTGCGGA
TTCAAAAGTACCTGGATCGAACAACTTTT
>17C_BF11_CONTIG_291_p48   23 pairs of NGS reads, 0.2%
CCCCACGAGAGATAGATGATAGTGAAGGAACCTCTATCTGAGAGAAGAGTCCGTTGAAACCTTTG
GACTTGAGATAGTAATCAAGGGCGTCGTTAGTCGCACCGAGAGCGGCGGAGGAAGCACTTCCCCC
AACGCCGGCTGGNNNNNGGCGGAGGAAGCACTTCCACCAACGCCGGCTGTAGCACCAGCACCGGA

GGCGACATCGGAGCAACAATTCCCCATAATTTTTGATTTCCCAACAAAAAAAACTGCGGATTCAA
AAGTACCTGGATCGAACAACGAGCTCAAAAA
>17C_BF11_CONTIG_214_p49   22 pairs of NGS reads, 0.19%
TTTTACGAGAGATAGATGATAGTGAAGGAACCTCTATCTGAGAGAAGAGTCCGTTGAAACCTTTG
GACTTGAgatagtaatcaagggcgtcgttagtcgcaccgagcaccagcaccggaggcgacatcgg
agcaacaattccCCATAATTTTTGATTTCCCAACAAAAAAAACTGCGGATTCAAAAGTACCTGGA
TCGAACAACGAGCTCAAAA
>17C_BF11_CONTIG_291_p50   22 pairs of NGS reads, 0.19%
CCCCACGAGAGATAGATGATAGTGAAGGAACCTCTATCTGAGAGAAGAGTCCGTTGAAACCTTTG
GACTTGAGATAGTAATCAAGGGCGTCGTTAGTCGCACCGAGAGCGGCGGAGGAAGCACTTCCACC
CACGCCGGCTGGNNNNNNGGCGGAGGAAGCACTTCCACCAACGCCGGCTGTAGCACCAGCACCGGA
GGCGACATCGGAGCAACAATTCCCCATAATTTTTGATTTCCCAACAAAAAAAACTGCGGATTCAA
AAGTACCTGGATCGAACAACGAGCTCAAAAA
>17C_BF11_CONTIG_290_p51   21 pairs of NGS reads, 0.18%
TTTTACGAGAGATAGATGATAGTGAAGGAACCTCTATCTGAGAGAAGAGTCCGTTGAAACCTTTG
GACTTGAGATAGTAATCAAGGGCGTCGTTAGTCGCACCGAGAGCGGCGGAGGAAGCACTTCCCCC
AACGCCGGCCGGNNNNNGAGAGCGGCGGAGGAAGCACTTCCACCAACGCCGGCTGTAGCACCAGC
ACCGGAGGCGACATCGGAGCAACAATTCCCCATAATTTTTGATTTCCCAACAAAAAAAACTGCGG
ATTCAAAAGTACCTGGATCGAACAACTTTT
>17C_BF11_CONTIG_217_p52   21 pairs of NGS reads, 0.18%
AAAAACGAGAGATAGATGATAGTGAAGGAACCTCTATCTGAGAGAAGAGTCCGTTGAAACCTTTG
GACTTGAGATagtaatcaagggcgtcgttagtcgcaccgaccggctgtagcaccagcaccggagg
cgacatcggagcaacaaTTCCCCATAATTTTTGATTTCCCAACAAAAAAAACTGCGGATTCAAAA
GTACCTGGATCGAACAACTTTT
>17C_BF11_CONTIG_217_p53   21 pairs of NGS reads, 0.18%
TTTTACGAGAGATAGATGATAGTGAAGGAACCTCTATCTGAGAGAAGAGTCCGTTGAAACCTTTG
GACTTGAGATagtaatcaagggcgtcgttagtcgcaccgaccggctgtagcaccagcaccggagg
cgacatcggagcaacAATTCCCCATAATTTTTGATTTCCCAACAAAAAAAACTGCGGATTCAAAA
GTACCTGGATCGAACAACTTTT
>17C_BF11_CONTIG_291_p54   21 pairs of NGS reads, 0.18%
CCCCACGAGAGATAGATGATAGTGAAGGAACCTCTATCTGAGAGAAGAGTCCGTTGAAACCTTTG
GACTTGAGATAGTAATCAAGGGCGTCGTTAGTCGCACCGAGAGCGGCGGAGGAAACACTTCCACC
AACGCCGGCCGTNNNNNGGCGGAGGAAGCACTTCCACCAACGCCGGCTGTAGCACCAGCACCGGA
GGCGACATCGGAGCAACAATTCCCCATAATTTTTGATTTCCCAACAAAAAAAACTGCGGATTCAA
AAGTACCTGGATCGAACAACGAGCTCAAAAA
>17C_BF11_CONTIG_291_p55   21 pairs of NGS reads, 0.18%
GGGGACGAGAGATAGATGATAGTGAAGGAACCTCTATCTGAGAGAAGAGTCCGTTGAAACCTTTG
GACTTGAGATAGTAATCAAGGGCGTCGTTAGTCGCACCGAGAGCGGCGGAGGAAGCACTTCCACC
AACGCCGGCGGGNNNNNGGCGGAGGAAGCACTTCCACCAACGCCGGCTGTAGCACCAGCACCGGA
GGCGACATCGGAGCAACAATTCCCCATAATTTTTGATTTCCCAACAAAAAAAACTGCGGATTCAA
AAGTACCTGGATCGAACAACGAGCTCAAAAA
>17C_BF11_CONTIG_220_p56   20 pairs of NGS reads, 0.17%
TTTTACGAGAGATAGATGATAGTGAAGGAACCTCTATCTGAGAGAAGAGTCCGTTGAAACCTTTG
GACTTGAGATAGTaatcaagggcgtcgttagtcgcacccggctgtagcaccagcaccggaggcga
catcggagcaacAATTCCCCATAATTTTTGATTTCCCAACAAAAAAAACTGCGGATTCAAAAGTA
CCTGGATCGAACAACGAGCTCAAAA
>17C_BF11_CONTIG_221_p57   20 pairs of NGS reads, 0.17%
AAAAACGAGAGATAGATGATAGTGAAGGAACCTCTATCTGAGAGAAGAGTCCGTTGAAACCTTTG
GACTTGAGATAGTAatcaagggcgtcgttagtcgcaccccggctgtagcaccagcaccggaggcg
acatcggagcaaCAATTCCCCATAATTTTTGATTTCCCAACAAAAAAAACTGCGGATTCAAAAGT
ACCTGGATCGAACAACGAGCTCAAAA
>17C_BF11_CONTIG_291_p58   20 pairs of NGS reads, 0.17%

GGGGACGAGAGATAGATGATAGTGAAGGAACCTCTATCTGAGAGAAGAGTCCGTTGAAACCTTTG
GACTTGAGATAGTAATCAAGGGCGTCGTTAGTCGCACCGAGAGCGGCGGAGGAAGCACTTCCACC
AACGCCCGCCGGNNNNNGGCGGAGGAAGCACTTCCACCAACGCCGGCTGTAGCACCAGCACCGGA
GGCGACATCGGAGCAACAATTCCCCATAATTTTTGATTTCCCAACAAAAAAAACTGCGGATTCAA
AAGTACCTGGATCGAACAACGAGCTCAAAAA
>17C_BF11_CONTIG_245_p59   20 pairs of NGS reads, 0.17%
TTTACGAGAGATAGATGATAGTGAAGGAACCTCTATCTGAGAGAAGAGTCCATTGAAACCTTTGG
ACTTGAGATAGTAATCAAGGGCGTCGTTAGTCGCACCGagagcggcggaggaagcacttccacca
acgccggctgtaGCACCAGCACCGGAGGCGACATCGGAGCAACAATTCCCCATAATTTTTGATTT
CCCAACAAAAAAAATTGCGGATTCAAAAGTACCTGGATCGAACAACTTTT
>17C_BF11_CONTIG_291_p60   19 pairs of NGS reads, 0.16%
CCCCACGAGAGATAGATGATAGTGAAGGAACCTCTATCTGAGAGAAGAGTCCGTTGAAACCTTTG
GACTTGAGATAGTAATCAAGGGCGTCGTTAGTCGCACCGAGAGCGGCGGAGGAAGCACTTCCACC
AACGCCGGCGGGNNNNNGGCGGAGGAAGCACTTCCACCAACGCCGGCTGTAGCACCAGCACCGGA
GGCGACATCGGAGCAACAATTCCCCATAATTTTTGATTTCCCAACAAAAAAAACTGCGGATTCAA
AAGTACCTGGATCGAACAACGAGCTCAAAAA
>17C_BF11_CONTIG_290_p61   19 pairs of NGS reads, 0.16%
TTTTACGAGAGATAGATGATAGTGAAGGAACCTCTATCTGAGAGAAGAGTCCGTTGAAACCTTTG
GACTTGAGATAGTAATCAAGGGCGTCGTTAGTCGCACCGAGAGCGGCGGAGGAAACACTTCCACC
AACGCCGGCCGGNNNNNGAGAGCGGCGGAGGAAGCACTTCCACCAACGCCGGCTGTAGCACCAGC
ACCGGAGGCGACATCGGAGCAACAATTCCCCATAATTTTTGATTTCCCAACAAAAAAAACTGCGG
ATTCAAAAGTACCTGGATCGAACAACTTTT
>17C_BF11_CONTIG_254_p62   18 pairs of NGS reads, 0.15%
AAAAACGAGAGATAGATGATAGTGAAGGAACCTCTATCTGAGAGAAGAGTCCGTTGAAACCTTTG
GACTTGAGATAGTAATCAAGGGCGTCGTTAGTCGCACCGAAGAGCggcggaggaagcacttccac
caacgccggctgTAGCACCAGCACCGGAGGCGACATCGGAGCAACAATTCCCCATAATTTTTGAT
TTCCCAACAAAAAAAACTGCGGATTCAAAAGTACCTGGATCGAACAACGAGCTCAAAAA
>17C_BF11_CONTIG_291_p63   18 pairs of NGS reads, 0.15%
CCCCACGAGAGATAGATGATAGTGAAGGAACCTCTATCTGAGAGAAGAGTCCGTTGAAACCTTTG
GACTTGAGATAGTAATCAAGGGCGTCGTTAGTCGCACCGAGAGCGGCGGAGGGAGCACTTCCACC
AACGCCGGCCGGNNNNNGGCGGAGGAAGCACTTCCACCAACGCCGGCTGTAGCACCAGCACCGGA
GGCGACATCGGAGCAACAATTCCCCATAATTTTTGATTTCCCAACAAAAAAAACTGCGGATTCAA
AAGTACCTGGATCGAACAACGAGCTCAAAAA
>17C_BF11_CONTIG_291_p64   18 pairs of NGS reads, 0.15%
GGGGACGAGAGATAGATGATAGTGAAGGAACCTCTATCTGAGAGAAGAGTCCGTTGAAACCTTTG
GACTTGAGATAGTAATCAAGGGCGTCGTTAGTCGCACCGAGAGCGGCGGAGGAAGCACCTCCACC
AACGCCGGCTGGNNNNNGGCGGAGGAAGCACTTCCACCAACGCCGGCTGTAGCACCAGCACCGGA
GGCGACATCGGAGCAACAATTCCCCATAATTTTTGATTTCCCAACAAAAAAAACTGCGGATTCAA
AAGTACCTGGATCGAACAACGAGCTCAAAAA
>17C_BF11_CONTIG_289_p65   18 pairs of NGS reads, 0.15%
TTTTACGAGAGATAGATGATAGTGAAGGAACCTCTATCTGAGAGAAGAGTCCGTTGAAACCTTTG
GACTTGAGATAGTAATCAAGGGCGTCGTTAGTCGCACCGAGAGCGGCGGAGGAAGCACCTCCACC
AACGCCGGCTGGNNNNNGGAGCGGCGGAGGAAGCACTTCCACCAACGCCGGCTGTAGCACCAGCA
CCGGAGGCGACATCGGAGCAACAATTCCCCATAATTTTTGATTTCCCAACAAAAAAAACTGCGGA
TTCAAAAGTACCTGGATCGAACAACTTTT
>17C_BF11_CONTIG_291_p66   18 pairs of NGS reads, 0.15%
GGGGACGAGAGATAGATGATAGTGAAGGAACCTCTATCTGAGAGAAGAGTCCGTTGAAACCTTTG
GACTTGAGATAGTAATCAAGGGCGTCGTTAGTCGCACCGAGAGCGGCGGAGGGAGCACTTCCACC
AACGCCGGCTGGNNNNNGGCGGAGGAAGCACTTCCACCAACGCCGGCTGTAGCACCAGCACCGGA
GGCGACATCGGAGCAACAATTCCCCATAATTTTTGATTTCCCAACAAAAAAAACTGCGGATTCAA
AAGTACCTGGATCGAACAACGAGCTCAAAAA
>17C_BF11_CONTIG_291_p67   18 pairs of NGS reads, 0.15%

CCCCACGAGAGATAGATGATAGTGAAGGAACCTCTATCTGAGAGAAGAGTCCGTTGAAACCTTTG
GACTTGAGATAGTAATCAAGGGCGTCGTTAGTCGCACCGAGAGCGGCGGAGGGAGCACTTCCACC
AACGCCGGCTGGNNNNNGGCGGAGGAAGCACTTCCACCAACGCCGGCTGTAGCACCAGCACCGGA
GGCGACATCGGAGCAACAATTCCCCATAATTTTTGATTTCCCAACAAAAAAAACTGCGGATTCAA
AAGTACCTGGATCGAACAACGAGCTCAAAAA

>17C_BF11_CONTIG_224_p68   18 pairs of NGS reads, 0.15%
AAAAACGAGAGATAGATGATAGTGAAGGAACCTCTATCTGAGAGAAGAGTCCGTTGAAACCTTTG
GACTTGAGATAGTAATCaagggcgtcgttagtcgcaccgaccggctgtagcaccagcaccggagg
cgacatcggagcaacaaTTCCCCATAATTTTTGATTTCCCAACAAAAAAAAACTGCGGATTCAAA
AGTACCTGGATCGAACAACGAGCTCAAAA

>17C_BF11_CONTIG_289_p69   17 pairs of NGS reads, 0.14%
TTTTACGAGAGATAGATGATAGTGAAGGAACCTCTATCTGAGAGAAGAGTCCGTTGAAACCTTTG
GACTTGAGATAGTAATCAAGGGCGTCGTTAGTCGCACCGAGAGCGGCGGAGGAAACACTTCCACC
AACGCCGGCCGGNNNNNGGAGCGGCGGAGGAAGCACTTCCACCAACGCCGGCTGTAGCACCAGCA
CCGGAGGCGACATCGGAGCAACAATTCCCCATAATTTTTGATTTCCCAACAAAAAAAACTGCGGA
TTCAAAAGTACCTGGATCGAACAACTTTT

>17C_BF11_CONTIG_292_p70   17 pairs of NGS reads, 0.14%
TTTTACGAGAGATAGATGATAGTGAAGGAACCTCTATCTGAGAGAAGAGTCCGTTGAAACCTTTG
GACTTGAGATAGTAATCAAGGGCGTCGTTAGTCGCACCGAGAGCGGCGGAGGAAGCACTTCCACC
AACGCCGGCCGGANNNNNGGCGGAGGAAGCACTTCCACCAACGCCGGCTGTAGCACCAGCACCGG
AGGCGACATCGGAGCAACAATTCCCCATAATTTTTGATTTCCCAACAAAAAAAACTGCGGATTCA
AAAGTACCTGGATCGAACAACGAGCTCAAAAA

>17C_BF11_CONTIG_291_p71   17 pairs of NGS reads, 0.14%
CCCCACGAGAGATAGATGATAGTGAAGGAACCTCTATCTGAGAGAAGAGTCCGTTGAAACCTTTG
GACTTGAGATAGTAATCAAGGGCGTCGTTAGTCGCACCGAGAGCGGCGGAGGAAGCACTTCCACC
AAAGCCGGCTGGNNNNNGGCGGAGGAAGCACTTCCACCAACGCCGGCTGTAGCACCAGCACCGGA
GGCGACATCGGAGCAACAATTCCCCATAATTTTTGATTTCCCAACAAAAAAAACTGCGGATTCAA
AAGTACCTGGATCGAACAACGAGCTCAAAAA

>17C_BF11_CONTIG_291_p72   17 pairs of NGS reads, 0.14%
GGGGACGAGAGATAGATGATAGTGAAGGAACCTCTATCTGAGAGAAGAGTCCGTTGAAACCTTTG
GACTTGAGATAGTAATCAAGGGCGTCGTTAGTCGCACCGAGAGCGGCGGAGGAAGCACCTCCACC
AACGCCGGCCGGNNNNNGGCGGAGGAAGCACTTCCACCAACGCCGGCTGTAGCACCAGCACCGGA
GGCGACATCGGAGCAACAATTCCCCATAATTTTTGATTTCCCAACAAAAAAAACTGCGGATTCAA
AAGTACCTGGATCGAACAACGAGCTCAAAAA

>17C_BF11_CONTIG_223_p73   17 pairs of NGS reads, 0.14%
AAAAACGAGAGATAGATGATAGTGAAGGAACCTCTATCTGAGAGAAGAGTCCGTTGAAACCTTTG
GACTTGAGATAGTAATcaagggcgtcgttagtcgccccgaccggctgtagcaccagcaccggagg
cgacatcggagcaacaaTTCCCCATAATTTTTGATTTCCCAACAAAAAAAACTGCGGATTCAAAA
GTACCTGGATCGAACAACGAGCTCAAAA

>17C_BF11_CONTIG_223_p74   17 pairs of NGS reads, 0.14%
TTTTACGAGAGATAGATGATAGTGAAGGAACCTCTATCTGAGAGAAGAGTCCGTTGAAACCTTTG
GACTTGAGATAGTAATcaagggcgtcgttagtcgccccgaccggctgtagcaccagcaccggagg
cgacatcggagcaacAATTCCCCATAATTTTTGATTTCCCAACAAAAAAAACTGCGGATTCAAAA
GTACCTGGATCGAACAACGAGCTCAAAA

>17C_BF11_CONTIG_254_p75   17 pairs of NGS reads, 0.14%
GGGGACGAGAGATAGATGATAGTGAAGGAACCTCTATCTGAGAGAAGAGTCCGTTGAAACCTTTG
GACTTGAGATAGTAATCAAGGGCGTCGTTAGTCGCACCGATGAGCggcggaggaagcacttccac
caacgccggctgTAGCACCAGCACCGGAGGCGACATCGGAGCAACAATTCCCCATAATTTTTGAT
TTCCCAACAAAAAAAACTGCGGATTCAAAAGTACCTGGATCGAACAACGAGCTCAAAAA

>17C_BF11_CONTIG_222_p76   16 pairs of NGS reads, 0.14%
AAAAACGAGAGATAGATGATAGTGAAGGAACCTCTATCTGAGAGAAGAGTCCGTTGAAACCTTTG
GACTTGAGATAGTAAccaagggcgtcgttagtcgcaccgacggctgtagcaccagcaccggaggc

gacatcggagcaACAATTCCCCATAATTTTTGATTTCCCAACAAAAAAAACTGCGGATTCAAAAG
TACCTGGATCGAACAACGAGCTCAAAA
>17C_BF11_CONTIG_290_p77   16 pairs of NGS reads, 0.14%
TTTTACGAGAGATAGATGATAGTGAAGGAACCTCTATCTGAGAGAAGAGTCCGTTGAAACCTTTG
GACTTGAGATAGTAATCAAGGGCGTCGTTAGTCGCACCGAGAGCGGCGGAGGAAGAACTTCCACC
AACGCCGGCCGGNNNNNGAGAGCGGCGGAGGAAGCACTTCCACCAACGCCGGCTGTAGCACCAGC
ACCGGAGGCGACATCGGAGCAACAATTCCCCATAATTTTTGATTTCCCAACAAAAAAAACTGCGG
ATTCAAAAGTACCTGGATCGAACAACTTTT
>17C_BF11_CONTIG_254_p78   16 pairs of NGS reads, 0.14%
AAAAACGAGAGATAGATGATAGTGAAGGAACCTCTATCTGAGAGAAGAGTCCGTTGAAACCTTTG
GACTTGAGATAGTAATCAAGGGCGTCGTTAGTCGCACCGATGAGCggcggaggaagcacttccac
caacgccggctgTAGCACCAGCACCGGAGGCGACATCGGAGCAACAATTCCCCATAATTTTTGAT
TTCCCAACAAAAAAAACTGCGGATTCAAAAGTACCTGGATCGAACAACGAGCTCAAAAA
>17C_BF11_CONTIG_222_p79   16 pairs of NGS reads, 0.14%
AAAAACGAGAGATAGATGATAGTGAAGGAACCTCTATCTGAGAGAAGAGTCCGTTGAAACCTTTG
GACTTGAGATAGTAAacaagggcgtcgttagtcgcaccgccggctgtagcaccagcaccggaggc
gacatcggagcaACAATTCCCCATAATTTTTGATTTCCCAACAAAAAAAACTGCGGATTCAAAAG
TACCTGGATCGAACAACGAGCTCAAAA
>17C_BF11_CONTIG_254_p80   16 pairs of NGS reads, 0.14%
GGGGACGAGAGATAGATGATAGTGAAGGAACCTCTATCTGAGAGAAGAGTCCGTTGAAACCTTTG
GACTTGAGATAGTAATCAAGGGCGTCGTTAGTCGCACCGAAGAGCggcggaggaagcacttccac
caacgccggctgTAGCACCAGCACCGGAGGCGACATCGGAGCAACAATTCCCCATAATTTTTGAT
TTCCCAACAAAAAAAACTGCGGATTCAAAAGTACCTGGATCGAACAACGAGCTCAAAAA
>17C_BF11_CONTIG_291_p81   16 pairs of NGS reads, 0.14%
GGGGACGAGAGATAGATGATAGTGAAGGAACCTCTATCTGAGAGAAGAGTCCGTTGAAACCTTTG
GACTTGAGATAGTAATCAAGGGCGTCGTTAGTCGCACCGAGAGCGGCGGAGGAAGCACTTCCACC
CACGCCGGCTGGNNNNNGGCGGAGGAAGCACTTCCACCAACGCCGGCTGTAGCACCAGCACCGGA
GGCGACATCGGAGCAACAATTCCCCATAATTTTTGATTTCCCAACAAAAAAAACTGCGGATTCAA
AAGTACCTGGATCGAACAACGAGCTCAAAAA
>17C_BF11_CONTIG_290_p82   16 pairs of NGS reads, 0.14%
TTTTACGAGAGATAGATGATAGTGAAGGAACCTCTATCTGAGAGAAGAGTCCGTTGAAACCTTTG
GACTTGAGATAGTAATCAAGGGCGTCGTTAGTCGCACCGAGAGCGGCGGAGGAAGCACTTCCACC
AACCCCGGCCGGNNNNNGAGAGCGGCGGAGGAAGCACTTCCACCAACGCCGGCTGTAGCACCAGC
ACCGGAGGCGACATCGGAGCAACAATTCCCCATAATTTTTGATTTCCCAACAAAAAAAACTGCGG
ATTCAAAAGTACCTGGATCGAACAACTTTT
>17C_BF11_CONTIG_290_p83   15 pairs of NGS reads, 0.13%
TTTTACGAGAGATAGATGATAGTGAAGGAACCTCTATCTGAGAGAAGAGTCCGTTGAAACCTTTG
GACTTGAGATAGTAATCAAGGGCGTCGTTAGTCGCACCGAGAGCGGCGGAGGAAGCACTTCCACC
CACGCCGGCCGGNNNNNGAGAGCGGCGGAGGAAGCACTTCCACCAACGCCGGCTGTAGCACCAGC
ACCGGAGGCGACATCGGAGCAACAATTCCCCATAATTTTTGATTTCCCAACAAAAAAAACTGCGG
ATTCAAAAGTACCTGGATCGAACAACTTTT
>17C_BF11_CONTIG_289_p84   15 pairs of NGS reads, 0.13%
TTTTACGAGAGATAGATGATAGTGAAGGAACCTCTATCTGAGAGAAGAGTCCGTTGAAACCTTTG
GACTTGAGATAGTAATCAAGGGCGTCGTTAGTCGCACCGAGAGCGGCGGAGGAAACACTTCCACC
AACGCCGGCTGTNNNNNGGAGCGGCGGAGGAAGCACTTCCACCAACGCCGGCTGTAGCACCAGCA
CCGGAGGCGACATCGGAGCAACAATTCCCCATAATTTTTGATTTCCCAACAAAAAAAACTGCGGA
TTCAAAAGTACCTGGATCGAACAACTTTT
>17C_BF11_CONTIG_289_p85   15 pairs of NGS reads, 0.13%
TTTTACGAGAGATAGATGATAGTGAAGGAACCTCTATCTGAGAGAAGAGTCCGTTGAAACCTTTG
GACTTGAGATAGTAATCAAGGGCGTCGTTAGTCGCACCGAGAGCGGCGGAGGAAGCACTTCCCCC
AACGCCGGCTGGNNNNNGGAGCGGCGGAGGAAGCACTTCCACCAACGCCGGCTGTAGCACCAGCA
CCGGAGGCGACATCGGAGCAACAATTCCCCATAATTTTTGATTTCCCAACAAAAAAAACTGCGGA

TTCAAAAGTACCTGGATCGAACAACTTTT
>17C_BF11_CONTIG_289_p86   15 pairs of NGS reads, 0.13%
CCCCACGAGAGATAGATGATAGTGAAGGAACAACGAGCTCAAAAAGGATTCAAAAGTACCTGGAT
CGAACAACGAGCTCAAAAAACATTGCGAGATCGGAAGAGCGGTTCAGCAGGAATGCCGAGACCGA
TCTCGTATGCCGNNNNNGGCGACCACCGAGATCTACACTCTTTCCCTACACGACGCTCTTCCGAT
CTCGCAATGTTCCCCACGAGAGATAGATGATAGTGAAGGAACAACGAGCTCAAAAAGGATTCAAA
AGTACCTGGATCGAACAACGAGCTCAAAA
>17C_BF11_CONTIG_291_p87   15 pairs of NGS reads, 0.13%
CCCCACGAGAGATAGATGATAGTGAAGGAACCTCTATCTGAGAGAAGAGTCCGTTGAAACCTTTG
GACTTGAGATAGTAATCAAGGGCGTCGTTAGTCGCACCGAGAGCGGCGGAGGAAGCACTTCCACC
AACGCCCGCCGGNNNNNGGCGGAGGAAGCACTTCCACCAACGCCGGCTGTAGCACCAGCACCGGA
GGCGACATCGGAGCAACAATTCCCCATAATTTTTGATTTCCCAACAAAAAAAACTGCGGATTCAA
AAGTACCTGGATCGAACAACGAGCTCAAAAA
>17C_BF11_CONTIG_291_p88   15 pairs of NGS reads, 0.13%
GGGGACGAGAGATAGATGATAGTGAAGGAACCTCTATCTGAGAGAAGAGTCCGTTGAAACCTTTG
GACTTGAGATAGTAATCAAGGGCGTCGTTAGTCGCACCGAGAGCGGCGGAGGAAGCACTTCCACC
AAAGCCGGCCGGNNNNNGGCGGAGGAAGCACTTCCACCAACGCCGGCTGTAGCACCAGCACCGGA
GGCGACATCGGAGCAACAATTCCCCATAATTTTTGATTTCCCAACAAAAAAAACTGCGGATTCAA
AAGTACCTGGATCGAACAACGAGCTCAAAAA
>17C_BF11_CONTIG_291_p89   15 pairs of NGS reads, 0.13%
GGGGACGAGAGATAGATGATAGTGAAGGAACCTCTATCTGAGAGAAGAGTCCGTTGAAACCTTTG
GACTTGAGATAGTAATCAAGGGCGTCGTTAGTCGCACCGAGAGCGGCGGAGGAAGCACTTCCACC
AAAGCCGGCTGGNNNNNGGCGGAGGAAGCACTTCCACCAACGCCGGCTGTAGCACCAGCACCGGA
GGCGACATCGGAGCAACAATTCCCCATAATTTTTGATTTCCCAACAAAAAAAACTGCGGATTCAA
AAGTACCTGGATCGAACAACGAGCTCAAAAA
>17C_BF11_CONTIG_290_p90   14 pairs of NGS reads, 0.12%
TTTTACGAGAGATAGATGATAGTGAAGGAACCTCTATCTGAGAGAAGAGTCCGTTGAAACCTTTG
GACTTGAGATAGTAATCAAGGGCGTCGTTAGTCGCACCGAGAGCGGCGGAGGAAGCACTTCCACC
AAAGCCGGCCGGNNNNNGAGAGCGGCGGAGGAAGCACTTCCACCAACGCCGGCTGTAGCACCAGC
ACCGGAGGCGACATCGGAGCAACAATTCCCCATAATTTTTGATTTCCCAACAAAAAAAACTGCGG
ATTCAAAAGTACCTGGATCGAACAACTTTT
>17C_BF11_CONTIG_289_p91   14 pairs of NGS reads, 0.12%
TTTTACGAGAGATAGATGATAGTGAAGGAACCTCTATCTGAGAGAAGAGTCCGTTGAAACCTTTG
GACTTGAGATAGTAATCAAGGGCGTCGTTAGTCGCACCGAGAGCGGCGGAGGAAGCACTTCCACC
AACGCCGGCGGGNNNNNGGAGCGGCGGAGGAAGCACTTCCACCAACGCCGGCTGTAGCACCAGCA
CCGGAGGCGACATCGGAGCAACAATTCCCCATAATTTTTGATTTCCCAACAAAAAAAACTGCGGA
TTCAAAAGTACCTGGATCGAACAACTTTT
>17C_BF11_CONTIG_291_p92   14 pairs of NGS reads, 0.12%
CCCCACGAGAGATAGATGATAGTGAAGGAACCTCTATCTGAGAGAAGAGTCCGTTGAAACCTTTG
GACTTGAGATAGTAATCAAGGGCGTCGTTAGTCGCACCGAGAGCGGCGGAGGAAGCACTTCCACC
AACCCCGGCTGGNNNNNGGCGGAGGAAGCACTTCCACCAACGCCGGCTGTAGCACCAGCACCGGA
GGCGACATCGGAGCAACAATTCCCCATAATTTTTGATTTCCCAACAAAAAAAACTGCGGATTCAA
AAGTACCTGGATCGAACAACGAGCTCAAAAA
>17C_BF11_CONTIG_291_p93   14 pairs of NGS reads, 0.12%
CCCCACGAGAGATAGATGATAGTGAAGGAACCTCTATCTGAGAGAAGAGTCCGTTGAAACCTTTG
GACTTGAGATAGTAATCAAGGGCGTCGTTAGTCGCACCGAGAGCGGCGGAGGAAGCACCTCCACC
AACGCCGGCTGGNNNNNGGCGGAGGAAGCACTTCCACCAACGCCGGCTGTAGCACCAGCACCGGA
GGCGACATCGGAGCAACAATTCCCCATAATTTTTGATTTCCCAACAAAAAAAACTGCGGATTCAA
AAGTACCTGGATCGAACAACGAGCTCAAAAA
>17C_BF11_CONTIG_289_p94   14 pairs of NGS reads, 0.12%
TTTTACGAGAGATAGATGATAGTGAAGGAACCTCTATCTGAGAGAAGAGTCCGTTGAAACCTTTG
GACTTGAGATAGTAATCAAGGGCGTCGTTAGTCGCACCGAGAGCGGCGGAGGGAGCACTTCCACC

AACGCCGGCTGTNNNNNGGAGCGGCGGAGGAAGCACTTCCACCAACGCCGGCTGTAGCACCAGCA
CCGGAGGCGACATCGGAGCAACAATTCCCCATAATTTTTGATTTCCCAACAAAAAAAACTGCGGA
TTCAAAAGTACCTGGATCGAACAACTTTT
>17C_BF11_CONTIG_290_p95   13 pairs of NGS reads, 0.11%
TTTTACGAGAGATAGATGATAGTGAAGGAACCTCTATCTGAGAGAAGAGTCCGTTGAAACCTTTG
GACTTGAGATAGTAATCAAGGGCGTCGTTAGTCGCACCGAGAGCGGCGGAGGGAGCACTTCCACC
AACGCCGGCCGGNNNNNGAGAGCGGCGGAGGAAGCACTTCCACCAACGCCGGCTGTAGCACCAGC
ACCGGAGGCGACATCGGAGCAACAATTCCCCATAATTTTTGATTTCCCAACAAAAAAAACTGCGG
ATTCAAAAGTACCTGGATCGAACAACTTTT
>17C_BF11_CONTIG_289_p96   13 pairs of NGS reads, 0.11%
TTTTACGAGAGATAGATGATAGTGAAGGAACCTCTATCTGAGAGAAGAGTCCGTTGAAACCTTTG
GACTTGAGATAGTAATCAAGGGCGTCGTTAGTCGCACCGAGAGCGGCGGAGGGAGCACTTCCACC
AACGCCGGCTGGNNNNNGGAGCGGCGGAGGAAGCACTTCCACCAACGCCGGCTGTAGCACCAGCA
CCGGAGGCGACATCGGAGCAACAATTCCCCATAATTTTTGATTTCCCAACAAAAAAAACTGCGGA
TTCAAAAGTACCTGGATCGAACAACTTTT
>17C_BF11_CONTIG_223_p97   13 pairs of NGS reads, 0.11%
TTTTACGAGAGATAGATGATAGTGAAGGAACCTCTATCTGAGAGAAGAGTCCGTTGAAACCTTTG
GACTTGAGATAGTAATcaagggggtcgttagtcgcaccgaccggctgtagcaccagcaccggagg
cgacatcggagcaacAATTCCCCATAATTTTTGATTTCCCAACAAAAAAAACTGCGGATTCAAAA
GTACCTGGATCGAACAACGAGCTCAAAA
>17C_BF11_CONTIG_224_p98   13 pairs of NGS reads, 0.11%
AAAAACGAGAGATAGATGATAGTGAAGGAACCTCTATCTGAGAGAAGAGTCCGTTGAAACCTTTG
GACTTGAGATAGTAATCaagggcgtcgttagtcgcaccgatccggctgtagcaccagcaccggag
gcgacatcggagCAACAATTCCCCATAATTTTTGATTTCCCAACAAAAAAAACTGCGGATTCAAA
AGTACCTGGATCGAACAACGAGCTCAAAA
>17C_BF11_CONTIG_291_p99   12 pairs of NGS reads, 0.1%
CCCCACGAGAGATAGATGATAGTGAAGGAACCTCTATCTGAGAGAAGAGTCCGTTGAAACCTTTG
GACTTGAGATAGTAATCAAGGGCGTCGTTAGTCGCACCGAGAGCGGCGGAGGAAGCACTTCCACC
AACGCCCGCTGGNNNNNGGCGGAGGAAGCACTTCCACCAACGCCGGCTGTAGCACCAGCACCGGA
GGCGACATCGGAGCAACAATTCCCCATAATTTTTGATTTCCCAACAAAAAAAACTGCGGATTCAA
AAGTACCTGGATCGAACAACGAGCTCAAAAA
>17C_BF11_CONTIG_291_p100   12 pairs of NGS reads, 0.1%
CCCCACGAGAGATAGATGATAGTGAAGGAACCTCTATCTGAGAGAAGAGTCCGTTGAAACCTTTG
GACTTGAGATAGTAATCAAGGGCGTCGTTAGTCGCACCGAGAGCGGCGGAGGAAGCACTTCCACC
AAAGCCGGCCGGNNNNNGGCGGAGGAAGCACTTCCACCAACGCCGGCTGTAGCACCAGCACCGGA
GGCGACATCGGAGCAACAATTCCCCATAATTTTTGATTTCCCAACAAAAAAAACTGCGGATTCAA
AAGTACCTGGATCGAACAACGAGCTCAAAAA
>17C_BF11_CONTIG_291_p101   12 pairs of NGS reads, 0.1%
GGGGACGAGAGATAGATGATAGTGAAGGAACCTCTATCTGAGAGAAGAGTCCGTTGAAACCTTTG
GACTTGAGATAGTAATCAAGGGCGTCGTTAGTCGCACCGAGAGCGGCGGAGGGAGCACTTCCACC
AACGCCGGCCGGNNNNNGGCGGAGGAAGCACTTCCACCAACGCCGGCTGTAGCACCAGCACCGGA
GGCGACATCGGAGCAACAATTCCCCATAATTTTTGATTTCCCAACAAAAAAAACTGCGGATTCAA
AAGTACCTGGATCGAACAACGAGCTCAAAAA
>17C_BF11_CONTIG_291_p102   12 pairs of NGS reads, 0.1%
CCCCACGAGAGATAGATGATAGTGAAGGAACCTCTATCTGAGAGAAGAGTCCGTTGAAACCTTTG
GACTTGAGATAGTAATCAAGGGCGTCGTTAGTCGCACCGAGAGCGGCGGAGGAAGCACTTCCCCC
AACGCCGGCCGGNNNNNGGCGGAGGAAGCACTTCCACCAACGCCGGCTGTAGCACCAGCACCGGA
GGCGACATCGGAGCAACAATTCCCCATAATTTTTGATTTCCCAACAAAAAAAACTGCGGATTCAA
AAGTACCTGGATCGAACAACGAGCTCAAAAA
>17C_BF11_CONTIG_291_p103   12 pairs of NGS reads, 0.1%
CCCCACGAGAGATAGATGATAGTGAAGGAACCTCTATCTGAGAGAAGAGTCCGTTGAAACCTTTG
GACTTGAGATAGTAATCAAGGGCGTCGTTAGTCGCACCGAGAGCGGCGGAGGAAGCACTTCCACC

AACCCCGGCCGTNNNNNGGCGGAGGAAGCACTTCCACCAACGCCGGCTGTAGCACCAGCACCGGA
GGCGACATCGGAGCAACAATTCCCCATAATTTTTGATTTCCCAACAAAAAAAACTGCGGATTCAA
AAGTACCTGGATCGAACAACGAGCTCAAAAA
>17C_BF11_CONTIG_289_p104   12 pairs of NGS reads, 0.1%
TTTTACGAGAGATAGATGATAGTGAAGGAACCTCTATCTGAGAGAAGAGTCCGTTGAAACCTTTG
GACTTGAGATAGTAATCAAGGGCGTCGTTAGTCGCACCGAGAGCGGCGGAGGAAGCACTTCCACC
AAAGCCGGCCGGNNNNNGGAGCGGCGGAGGAAGCACTTCCACCAACGCCGGCTGTAGCACCAGCA
CCGGAGGCGACATCGGAGCAACAATTCCCCATAATTTTTGATTTCCCAACAAAAAAAACTGCGGA
TTCAAAAGTACCTGGATCGAACAACTTTT
>17C_BF11_CONTIG_219_p105   12 pairs of NGS reads, 0.1%
AAAAACGAGAGATAGATGATAGTGAAGGAACCTCTATCTGAGAGAAGAGTCCGTTGAAACCTTTG
GACTTGAGATAGtaatcaagggcgtcgttagtcgcaccgactgtagcaccagcaccggaggcgac
atcggagcaacaATTCCCCATAATTTTTGATTTCCCAACAAAAAAAACTGCGGATTCAAAAGTAC
CTGGATCGAACAACGAGCTCAAAA
>17C_BF11_CONTIG_290_p106   12 pairs of NGS reads, 0.1%
TTTTACGAGAGATAGATGATAGTGAAGGAACCTCTATCTGAGAGAAGAGTCCGTTGAAACCTTTG
GACTTGAGATAGTAATCAAGGGCGTCGTTAGTCGCACCGAGAGCGGCGGAGGAAGCACTTCCACC
AACCCCGGCCGTANNNNNGGAGCGGCGGAGGAAGCACTTCCACCAACGCCGGCTGTAGCACCAGC
ACCGGAGGCGACATCGGAGCAACAATTCCCCATAATTTTTGATTTCCCAACAAAAAAAACTGCGG
ATTCAAAAGTACCTGGATCGAACAACTTTT
>17C_BF11_CONTIG_291_p107   12 pairs of NGS reads, 0.1%
GGGGACGAGAGATAGATGATAGTGAAGGAACCTCTATCTGAGAGAAGAGTCCGTTGAAACCTTTG
GACTTGAGATAGTAATCAAGGGCGTCGTTAGTCGCACCGAGAGCGGCGGAGGAAGCACCTCCACC
AACGCCCGCCGGNNNNNGGCGGAGGAAGCACTTCCACCAACGCCGGCTGTAGCACCAGCACCGGA
GGCGACATCGGAGCAACAATTCCCCATAATTTTTGATTTCCCAACAAAAAAAACTGCGGATTCAA
AAGTACCTGGATCGAACAACGAGCTCAAAAA

AtBON1 RNP 2
CACGAGAGATAGATGATAGTGAAGGAACCTCTATCTGCGAGAAGAGTCCGTTGAAACCTTTGGAC
TTGAGATAGTAATCAAGGGCGTCGTTAGTCGCACCGAGAGCGGCGGAGGAACCACTTCCACCAAC
GCCGGCTGTAGCACCAGCACCGGAGGCGACATCGGAGCAACAATTCCCCATAATTTTTGATTTCC
CAACAAAAAAAACTGCGGATTCAAAAGTACCTGGATCGAACAAC
>B621_CF5_CONTIG_231_p1   7982 pairs of NGS reads, 87.55%
TTTTTGGATCCACGAGAGATAGATGATAGTGAAGGAACCTCTATCTGCGAGAAGAGTCCGTTGAA
ACCTTTGGACTTGAGATAGTaatcaagggcgtcgttagtcgcaccgaccggctgtagcaccagca
ccggaggcgacatCGGAGCAACAATTCCCCATAATTTTTGATTTCCCAACAAAAAAAACTGCGGA
TTCAAAAGTACCTGGATCGAACAACGAGCTCAAAAA
>B621_CF5_CONTIG_260_p2   939 pairs of NGS reads, 10.29%
TTTTTGGATCCACGAGAGATAGATGATAGTGAAGGAACCTCTATCTGCGAGAAGAGTCCGTTGAA
ACCTTTGGACTTGAGATAGTAATCAAGGGCGTCGTTAGTCGCACCGAGAGCGgcggaggaaccac
ttccaccaacgccGGCTGTAGCACCAGCACCGGAGGCGACATCGGAGCAACAATTCCCCATAATT
TTTGATTTCCCAACAAAAAAAACTGCGGATTCAAAAGTACCTGGATCGAACAACGAGCTCAAAAA
>B621_CF5_CONTIG_230_p3   196 pairs of NGS reads, 2.14%
TTTTTGGATCCACGAGAGATAGATGATAGTGAAGGAACCTCTATCTGCGAGAAGAGTCCGTTGAA
ACCTTTGGACTTGAGATAGTAATcaagggcgtcgttagtcgcaccgccggctgtagcaccagcac
cggaggcgacatCGGAGCAACAATTCCCCATAATTTTTGATTTCCCAACAAAAAAAACTGCGGAT
TCAAAAGTACCTGGATCGAACAACGAGCTCAAAAA

AtBON1 RNP 3
>17C_BF11_CONTIG_224_p1   1898 pairs of NGS reads, 16.63%

AAAAACGAGAGATAGATGATAGTGAAGGAACCTCTATCTGAGAGAAGAGTCCGTTGAAACCTTTG
GACTTGAGATagtaatcaagggcgtcgttagtcgcaccgaccggctgtagcaccagcaccggagg
cgacatcggagcaacaaTTCCCCATAATTTTTGATTTCCCAACAAAAAAAACTGCGGATTCAAAA
GTACCTGGATCGAACAACGAGCTCAAAAA
>17C_BF11_CONTIG_224_p2   1559 pairs of NGS reads, 13.66%
TTTTACGAGAGATAGATGATAGTGAAGGAACCTCTATCTGAGAGAAGAGTCCGTTGAAACCTTTG
GACTTGAGATagtaatcaagggcgtcgttagtcgcaccgaccggctgtagcaccagcaccggagg
cgacatcggagcaacAATTCCCCATAATTTTTGATTTCCCAACAAAAAAAACTGCGGATTCAAAA
GTACCTGGATCGAACAACGAGCTCAAAAA
>17C_BF11_CONTIG_253_p3   733 pairs of NGS reads, 6.42%
CCCCACGAGAGATAGATGATAGTGAAGGAACCTCTATCTGAGAGAAGAGTCCGTTGAAACCTTTG
GACTTGAGATAGTAATCAAGGGCGTCGTTAGTCGCACCGAGAGCggcggaggaagcacttccacc
aacgccggctgtAGCACCAGCACCGGAGGCGACATCGGAGCAACAATTCCCCATAATTTTTGATT
TCCCAACAAAAAAAACTGCGGATTCAAAAGTACCTGGATCGAACAACGAGCTCAAAAA
>17C_BF11_CONTIG_253_p4   520 pairs of NGS reads, 4.55%
GGGGACGAGAGATAGATGATAGTGAAGGAACCTCTATCTGAGAGAAGAGTCCGTTGAAACCTTTG
GACTTGAGATAGTAATCAAGGGCGTCGTTAGTCGCACCGAGAGCggcggaggaagcacttccacc
aacgccggctgtAGCACCAGCACCGGAGGCGACATCGGAGCAACAATTCCCCATAATTTTTGATT
TCCCAACAAAAAAAACTGCGGATTCAAAAGTACCTGGATCGAACAACGAGCTCAAAAA
>17C_BF11_CONTIG_246_p5   1237 pairs of NGS reads, 10.84%
TTTTACGAGAGATAGATGATAGTGAAGGAACCTCTATCTGAGAGAAGAGTCCGTTGAAACCTTTG
GACTTGAGATAGTAATCAAGGGCGTCGTTAGTCGCACCgagagcggcggaggaagcacttccacc
aacgccggctgtagcaccAGCACCGGAGGCGACATCGGAGCAACAATTCCCCATAATTTTTGATT
TCCCAACAAAAAAAACTGCGGATTCAAAAGTACCTGGATCGAACAACTTTT
>17C_BF11_CONTIG_253_p6   358 pairs of NGS reads, 3.13%
AAAAACGAGAGATAGATGATAGTGAAGGAACCTCTATCTGAGAGAAGAGTCCGTTGAAACCTTTG
GACTTGAGATAGTAATCAAGGGCGTCGTTAGTCGCACCGAGAGCggcggaggaagcacttccacc
aacgccggctgtAGCACCAGCACCGGAGGCGACATCGGAGCAACAATTCCCCATAATTTTTGATT
TCCCAACAAAAAAAACTGCGGATTCAAAAGTACCTGGATCGAACAACGAGCTCAAAAA
>17C_BF11_CONTIG_246_p7   466 pairs of NGS reads, 4.08%
TTTTACGAGAGATAGATGATAGTGAAGGAACCTCTATCTGAGAGAAGAGTCCGTTGAAACCTTTG
GACTTGAGATAGTAATCAAGGGCGTCGTTAGTCGCACCgagagcggcggaggaagcacttccacc
aacgccggctggaGCACCAGCACCGGAGGCGACATCGGAGCAACAATTCCCCATAATTTTTGATT
TCCCAACAAAAAAAACTGCGGATTCAAAAGTACCTGGATCGAACAACTTTT
>17C_BF11_CONTIG_252_p8   294 pairs of NGS reads, 2.57%
CCCCACGAGAGATAGATGATAGTGAAGGAACCTCTATCTGAGAGAAGAGTCCGTTGAAACCTTTG
GACTTGAGATAGTAATCAAGGGCGTCGTTAGTCGCACCGAGAGCGgcggaggaacctcatccacc
aacgccggctgtAGCACCAGCACCGGAGGCGACATCGGAGCAACAATTCCCCATAATTTTTGATT
TCCCAACAAAAAAAACTGCGGATTCAAAAGTACCTGGATCGAACAACGAGCTCAAAA
>17C_BF11_CONTIG_291_p9   131 pairs of NGS reads, 1.14%
CCCCACGAGAGATAGATGATAGTGAAGGAACCTCTATCTGAGAGAAGAGTCCGTTGAAACCTTTG
GACTTGAGATAGTAATCAAGGGCGTCGTTAGTCGCACCGAGAGCGGCGGAGGAAGCACTTCCACC
AACGCCGGCCGGNNNNNGGCGGAGGAAGCACTTCCACCAACGCCGGCTGTAGCACCAGCACCGGA
GGCGACATCGGAGCAACAATTCCCCATAATTTTTGATTTCCCAACAAAAAAAACTGCGGATTCAA
AAGTACCTGGATCGAACAACGAGCTCAAAAA
>17C_BF11_CONTIG_246_p10   118 pairs of NGS reads, 1.03%
TTTTACGAGAGATAGATGATAGTGAAGGAACCTCTATCTGAGAGAAGAGTCCGTTGAAACCTTTG
GACTTGAGATAGTAATCAAGGGCGTCGTTAGTCGCACCgagagcggcggaggaagcacttccacc
aacgccggccggaGCACCAGCACCGGAGGCGACATCGGAGCAACAATTCCCCATAATTTTTGATT
TCCCAACAAAAAAAACTGCGGATTCAAAAGTACCTGGATCGAACAACTTTT
>17C_BF11_CONTIG_246_p11   179 pairs of NGS reads, 1.56%
TTTTACGAGAGATAGATGATAGTGAAGGAACCTCTATCTGAGAGAAGAGTCCGTTGAAACCTTTG

GACTTGAGATAGTAATCAAGGGCGTCGTTAGTCGCACCgagagcggcggaggaagcacttccacc
aacgccggccgtaGCACCAGCACCGGAGGCGACATCGGAGCAACAATTCCCCATAATTTTTGATT
TCCCAACAAAAAAAACTGCGGATTCAAAAGTACCTGGATCGAACAACTTTT
>17C_BF11_CONTIG_291_p12   105 pairs of NGS reads, 0.92%
GGGGACGAGAGATAGATGATAGTGAAGGAACCTCTATCTGAGAGAAGAGTCCGTTGAAACCTTTG
GACTTGAGATAGTAATCAAGGGCGTCGTTAGTCGCACCGAGAGCGGCGGAGGAAGCACTTCCACC
AACGCCGGCCGGNNNNNGGCGGAGGAAGCACTTCCACCAACGCCGGCTGTAGCACCAGCACCGGA
GGCGACATCGGAGCAACAATTCCCCATAATTTTTGATTTCCCAACAAAAAAAACTGCGGATTCAA
AAGTACCTGGATCGAACAACGAGCTCAAAAA
>17C_BF11_CONTIG_222_p13   87 pairs of NGS reads, 0.76%
AAAAACGAGAGATAGATGATAGTGAAGGAACCTCTATCTGAGAGAAGAGTCCGTTGAAACCTTTG
GACTTGAGATAGTAAtcaagggcgtcgttagtcgcaccgacggctgtagcaccagcaccggaggc
gacatcggagcaACAATTCCCCATAATTTTTGATTTCCCAACAAAAAAAACTGCGGATTCAAAAG
TACCTGGATCGAACAACGAGCTCAAAA
>17C_BF11_CONTIG_222_p14   83 pairs of NGS reads, 0.72%
AAAAACGAGAGATAGATGATAGTGAAGGAACCTCTATCTGAGAGAAGAGTCCGTTGAAACCTTTG
GACTTGAGATAGTAAtcaagggcgtcgttagtcgcaccgccggctgtagcaccagcaccggaggc
gacatcggagcaACAATTCCCCATAATTTTTGATTTCCCAACAAAAAAAACTGCGGATTCAAAAG
TACCTGGATCGAACAACGAGCTCAAAA
>17C_BF11_CONTIG_290_p15   78 pairs of NGS reads, 0.68%
TTTTACGAGAGATAGATGATAGTGAAGGAACCTCTATCTGAGAGAAGAGTCCGTTGAAACCTTTG
GACTTGAGATAGTAATCAAGGGCGTCGTTAGTCGCACCGAGAGCGGCGGAGGAAGCACTTCCACC
AACGCCGGCCGGANNNNNGGAGCGGCGGAGGAAGCACTTCCACCAACGCCGGCTGTAGCACCAGC
ACCGGAGGCGACATCGGAGCAACAATTCCCCATAATTTTTGATTTCCCAACAAAAAAAACTGCGG
ATTCAAAAGTACCTGGATCGAACAACTTTT
>17C_BF11_CONTIG_252_p16   78 pairs of NGS reads, 0.68%
GGGGACGAGAGATAGATGATAGTGAAGGAACCTCTATCTGAGAGAAGAGTCCGTTGAAACCTTTG
GACTTGAGATAGTAATCAAGGGCGTCGTTAGTCGCACCGAGAGCGgcggaggaacctcatccacc
aacgccggctgtAGCACCAGCACCGGAGGCGACATCGGAGCAACAATTCCCCATAATTTTTGATT
TCCCAACAAAAAAAACTGCGGATTCAAAAGTACCTGGATCGAACAACGAGCTCAAAA
>17C_BF11_CONTIG_170_p17   67 pairs of NGS reads, 0.58%
AAAAGTTGTTCGATCCAGGTACTTTTGaatcatagtggttagggctttggtcccaatgatgacgc
ttgatgctctttttgaacaaaagggtgaagttgccaagtctgttcttgaggagcttgagaaggta
taacatcttgttCCTTCACTATCATCTATCTCTCGTAAAA
>17C_BF11_CONTIG_253_p18   145 pairs of NGS reads, 1.27%
TTTTACGAGAGATAGATGATAGTGAAGGAACCTCTATCTGAGAGAAGAGTCCGTTGAAACCTTTG
GACTTGAGATAGTAATCAAGGGCGTCGTTAGTCGCACCGAGAGCggcggaggaagcacttccacc
aacgccggctgtaGCACCAGCACCGGAGGCGACATCGGAGCAACAATTCCCCATAATTTTTGATT
TCCCAACAAAAAAAACTGCGGATTCAAAAGTACCTGGATCGAACAACGAGCTCAAAAA
>17C_BF11_CONTIG_224_p19   55 pairs of NGS reads, 0.48%
GGGGACGAGAGATAGATGATAGTGAAGGAACCTCTATCTGAGAGAAGAGTCCGTTGAAACCTTTG
GACTTGAGATagtaatcaagggcgtcgttagtcgcaccgaccggctgtagcaccagcaccggagg
cgacatcggagcAACAATTCCCCATAATTTTTGATTTCCCAACAAAAAAAACTGCGGATTCAAAA
GTACCTGGATCGAACAACGAGCTCAAAAA
>17C_BF11_CONTIG_223_p20   50 pairs of NGS reads, 0.43%
AAAAACGAGAGATAGATGATAGTGAAGGAACCTCTATCTGAGAGAAGAGTCCGTTGAAACCTTTG
GACTTGAGATAGTAATaaagggcgtcgttagtcgcaccgaccggctgtagcaccagcaccggagg
cgacatcggagcaacaaTTCCCCATAATTTTTGATTTCCCAACAAAAAAAACTGCGGATTCAAAA
GTACCTGGATCGAACAACGAGCTCAAAA
>17C_BF11_CONTIG_291_p21   50 pairs of NGS reads, 0.43%
AAAAACGAGAGATAGATGATAGTGAAGGAACCTCTATCTGAGAGAAGAGTCCGTTGAAACCTTTG
GACTTGAGATAGTAATCAAGGGCGTCGTTAGTCGCACCGAGAGCGGCGGAGGAAGCACTTCCACC

AACGCCGGCCGGNNNNNGGCGGAGGAAGCACTTCCACCAACGCCGGCTGTAGCACCAGCACCGGA
GGCGACATCGGAGCAACAATTCCCCATAATTTTTGATTTCCCAACAAAAAAAACTGCGGATTCAA
AAGTACCTGGATCGAACAACGAGCTCAAAAA
>17C_BF11_CONTIG_289_p22   40 pairs of NGS reads, 0.35%
CCCCACGAGAGATAGATGATAGTGAAGGAACCTCTATCTGAGAGAAGAGTCCGTTGAAACCTTTG
GACTTGAGATAGTAATCAAGGGCGTCGTTAGTCGCACCGAGAGCGGCGGAGGAACCTCATCCACC
AACGCCGGCCGGNNNNNGCGGAGGAACCTCATCCACCAACGCCGGCTGTAGCACCAGCACCGGAG
GCGACATCGGAGCAACAATTCCCCATAATTTTTGATTTCCCAACAAAAAAAACTGCGGATTCAAA
AGTACCTGGATCGAACAACGAGCTCAAAA
>17C_BF11_CONTIG_221_p23   38 pairs of NGS reads, 0.33%
AAAAACGAGAGATAGATGATAGTGAAGGAACCTCTATCTGAGAGAAGAGTCCGTTGAAACCTTTG
GACTTGAGATAGTAatcaagggcgtcgttagtcgcaccgaggctgtagcaccagcaccggaggcg
acatcggagcaaCAATTCCCCATAATTTTTGATTTCCCAACAAAAAAAACTGCGGATTCAAAAGT
ACCTGGATCGAACAACGAGCTCAAAA
>17C_BF11_CONTIG_223_p24   38 pairs of NGS reads, 0.33%
TTTTACGAGAGATAGATGATAGTGAAGGAACCTCTATCTGAGAGAAGAGTCCGTTGAAACCTTTG
GACTTGAGATAGTAATaaagggcgtcgttagtcgcaccgaccggctgtagcaccagcaccggagg
cgacatcggagcaacAATTCCCCATAATTTTTGATTTCCCAACAAAAAAAACTGCGGATTCAAAA
GTACCTGGATCGAACAACGAGCTCAAAA
>17C_BF11_CONTIG_246_p25   36 pairs of NGS reads, 0.31%
TTTTACGAGAGATAGATGATAGTGAAGGAACCTCTATCTGAGAGAAGAGTCCGTTGAAACCTTTG
GACTTGAGATAGTAATCAAGGGCGTCGTTAGTCGCACCgagagcggcggaggaagcacttccccc
aacgccggctgtaGCACCAGCACCGGAGGCGACATCGGAGCAACAATTCCCCATAATTTTTGATT
TCCCAACAAAAAAAACTGCGGATTCAAAAGTACCTGGATCGAACAACTTTT
>17C_BF11_CONTIG_224_p26   36 pairs of NGS reads, 0.31%
CCCCACGAGAGATAGATGATAGTGAAGGAACCTCTATCTGAGAGAAGAGTCCGTTGAAACCTTTG
GACTTGAGATagtaatcaagggcgtcgttagtcgcaccgaccggctgtagcaccagcaccggagg
cgacatcggagcAACAATTCCCCATAATTTTTGATTTCCCAACAAAAAAAACTGCGGATTCAAAA
GTACCTGGATCGAACAACGAGCTCAAAAA
>17C_BF11_CONTIG_290_p27   35 pairs of NGS reads, 0.3%
TTTTACGAGAGATAGATGATAGTGAAGGAACCTCTATCTGAGAGAAGAGTCCGTTGAAACCTTTG
GACTTGAGATAGTAATCAAGGGCGTCGTTAGTCGCACCGAGAGCGGCGGAGGAAGCACTTCCACC
AACGCCCGCCGGNNNNNGAGAGCGGCGGAGGAAGCACTTCCACCAACGCCGGCTGTAGCACCAGC
ACCGGAGGCGACATCGGAGCAACAATTCCCCATAATTTTTGATTTCCCAACAAAAAAAACTGCGG
ATTCAAAAGTACCTGGATCGAACAACTTTT
>17C_BF11_CONTIG_246_p28   34 pairs of NGS reads, 0.29%
TTTTACGAGAGATAGATGATAGTGAAGGAACCTCTATCTGAGAGAAGAGTCCGTTGAAACCTTTG
GACTTGAGATAGTAATCAAGGGCGTCGTTAGTCGCACCGagagcggcggaggaagcacttccacc
aacgccggctgtagcaccAGCACCGGAGGCGACATCGGAGCAACAATTCCCCATAATTTTTGATT
TCCCAACAAAAAAAATTGCGGATTCAAAAGTACCTGGATCGAACAACTTTT
>17C_BF11_CONTIG_220_p29   33 pairs of NGS reads, 0.28%
AAAAACGAGAGATAGATGATAGTGAAGGAACCTCTATCTGAGAGAAGAGTCCGTTGAAACCTTTG
GACTTGAGATAGTaatcaagggcgtcgttagtcgcacccggctgtagcaccagcaccggaggcga
catcggagcaacAATTCCCCATAATTTTTGATTTCCCAACAAAAAAAACTGCGGATTCAAAAGTA
CCTGGATCGAACAACGAGCTCAAAA
>17C_BF11_CONTIG_291_p30   32 pairs of NGS reads, 0.28%
CCCCACGAGAGATAGATGATAGTGAAGGAACCTCTATCTGAGAGAAGAGTCCGTTGAAACCTTTG
GACTTGAGATAGTAATCAAGGGCGTCGTTAGTCGCACCGAGAGCGGCGGAGGAAGCACCTCCACC
AACGCCGGCCGGNNNNNGGCGGAGGAAGCACTTCCACCAACGCCGGCTGTAGCACCAGCACCGGA
GGCGACATCGGAGCAACAATTCCCCATAATTTTTGATTTCCCAACAAAAAAAACTGCGGATTCAA
AAGTACCTGGATCGAACAACGAGCTCAAAAA
>17C_BF11_CONTIG_222_p31   30 pairs of NGS reads, 0.26%

TTTTACGAGAGATAGATGATAGTGAAGGAACCTCTATCTGAGAGAAGAGTCCGTTGAAACCTTTG
GACTTGAGATAGTAAtcaagggcgtcgttagtcgcaccgccggctgtagcaccagcaccggaggc
gacatcggagcaACAATTCCCCATAATTTTTGATTTCCCAACAAAAAAAACTGCGGATTCAAAAG
TACCTGGATCGAACAACGAGCTCAAAA
>17C_BF11_CONTIG_223_p32   28 pairs of NGS reads, 0.24%
AAAAACGAGAGATAGATGATAGTGAAGGAACCTCTATCTGAGAGAAGAGTCCGTTGAAACCTTTG
GACTTGAGATAGTAATcaaggggtcgttagtcgcaccgaccggctgtagcaccagcaccggagg
cgacatcggagcaacaaTTCCCCATAATTTTTGATTTCCCAACAAAAAAAACTGCGGATTCAAAA
GTACCTGGATCGAACAACGAGCTCAAAA
>17C_BF11_CONTIG_246_p33   28 pairs of NGS reads, 0.24%
TTTTACGAGAGATAGATGATAGTGAAGGAACCTCTATCTGAGAGAAGAGTCCGTTGAAACCTTTG
GACTTGAGATAGTAATCAAGGGCGTCGTTAGTCGCACCgagagcggcggaggaagcacctccacc
aacgccggctggAGCACCAGCACCGGAGGCGACATCGGAGCAACAATTCCCCATAATTTTTGATT
TCCCAACAAAAAAAACTGCGGATTCAAAAGTACCTGGATCGAACAACTTTT
>17C_BF11_CONTIG_246_p34   27 pairs of NGS reads, 0.23%
TTTTACGAGAGATAGATGATAGTGAAGGAACCTCTATCTGAGAGAAGAGTCCGTTGAAACCTTTG
GACTTGAGATAGTAATCAAGGGCGTCGTTAGTCGCACCgagagcggcggagggagcacttccacc
aacgccggctgtAGCACCAGCACCGGAGGCGACATCGGAGCAACAATTCCCCATAATTTTTGATT
TCCCAACAAAAAAAACTGCGGATTCAAAAGTACCTGGATCGAACAACTTTT
>17C_BF11_CONTIG_215_p35   27 pairs of NGS reads, 0.23%
TTTTACGAGAGATAGATGATAGTGAAGGAACCTCTATCTGAGAGAAGAGTCCGTTGAAACCTTTG
GACTTGAGatagtaatcaagggcgtcgttagtccggctgtagcaccagcaccggaggcgacatcg
gagcaacaattcCCCATAATTTTTGATTTCCCAACAAAAAAAACTGCGGATTCAAAAGTACCTGG
ATCGAACAACGAGCTCAAAA
>17C_BF11_CONTIG_222_p36   27 pairs of NGS reads, 0.23%
TTTTACGAGAGATAGATGATAGTGAAGGAACCTCTATCTGAGAGAAGAGTCCGTTGAAACCTTTG
GACTTGAGATAGTAAtcaagggcgtcgttagtcgcaccgacggctgtagcaccagcaccggaggc
gacatcggagcaACAATTCCCCATAATTTTTGATTTCCCAACAAAAAAAACTGCGGATTCAAAAG
TACCTGGATCGAACAACGAGCTCAAAA
>17C_BF11_CONTIG_219_p37   27 pairs of NGS reads, 0.23%
AAAAACGAGAGATAGATGATAGTGAAGGAACCTCTATCTGAGAGAAGAGTCCGTTGAAACCTTTG
GACTTGAGATAGtaatcaagggcgtcgttagtcgcaccggctgtagcaccagcaccggaggcgac
atcggagcaacaATTCCCCATAATTTTTGATTTCCCAACAAAAAAAACTGCGGATTCAAAAGTAC
CTGGATCGAACAACGAGCTCAAAA
>17C_BF11_CONTIG_291_p38   27 pairs of NGS reads, 0.23%
CCCCACGAGAGATAGATGATAGTGAAGGAACCTCTATCTGAGAGAAGAGTCCGTTGAAACCTTTG
GACTTGAGATAGTAATCAAGGGCGTCGTTAGTCGCACCGAGAGCGGCGGAGGAAACACTTCCACC
AACGCCGGCCGGNNNNNNGGCGGAGGAAGCACTTCCACCAACGCCGGCTGTAGCACCAGCACCGGA
GGCGACATCGGAGCAACAATTCCCCATAATTTTTGATTTCCCAACAAAAAAAACTGCGGATTCAA
AAGTACCTGGATCGAACAACGAGCTCAAAAA
>17C_BF11_CONTIG_290_p39   26 pairs of NGS reads, 0.22%
TTTTACGAGAGATAGATGATAGTGAAGGAACCTCTATCTGAGAGAAGAGTCCGTTGAAACCTTTG
GACTTGAGATAGTAATCAAGGGCGTCGTTAGTCGCACCGAGAGCGGCGGAGGAAGCACCTCCACC
AACGCCGGCCGGNNNNNNGAGAGCGGCGGAGGAAGCACTTCCACCAACGCCGGCTGTAGCACCAGC
ACCGGAGGCGACATCGGAGCAACAATTCCCCATAATTTTTGATTTCCCAACAAAAAAAACTGCGG
ATTCAAAAGTACCTGGATCGAACAACTTTT
>17C_BF11_CONTIG_246_p40   26 pairs of NGS reads, 0.22%
TTTTACGAGAGATAGATGATAGTGAAGGAACCTCTATCTGAGAGAAGAGTCCGTTGAAACCTTTG
GACTTGAGATAGTAATCAAGGGCGTCGTTAGTCGCACCgagagcggcggaggaagcacttccacc
aacgcccgctggAGCACCAGCACCGGAGGCGACATCGGAGCAACAATTCCCCATAATTTTTGATT
TCCCAACAAAAAAAACTGCGGATTCAAAAGTACCTGGATCGAACAACTTTT
>17C_BF11_CONTIG_252_p41   25 pairs of NGS reads, 0.21%

AAAAACGAGAGATAGATGATAGTGAAGGAACCTCTATCTGAGAGAAGAGTCCGTTGAAACCTTTG
GACTTGAGATAGTAATCAAGGGCGTCGTTAGTCGCACCGAGAGCGgcggaggaacctcatccacc
aacgccggctgtAGCACCAGCACCGGAGGCGACATCGGAGCAACAATTCCCCATAATTTTTGATT
TCCCAACAAAAAAAACTGCGGATTCAAAAGTACCTGGATCGAACAACGAGCTCAAAA
>17C_BF11_CONTIG_246_p42   25 pairs of NGS reads, 0.21%
TTTTACGAGAGATAGATGATAGTGAAGGAACCTCTATCTGAGAGAAGAGTCCGTTGAAACCTTTG
GACTTGAGATAGTAATCAAGGGCGTCGTTAGTCGCACCgagagcggcggaggaagcacctccacc
aacgccggctgtAGCACCAGCACCGGAGGCGACATCGGAGCAACAATTCCCCATAATTTTTGATT
TCCCAACAAAAAAAACTGCGGATTCAAAAGTACCTGGATCGAACAACTTTT
>17C_BF11_CONTIG_291_p43   25 pairs of NGS reads, 0.21%
GGGGACGAGAGATAGATGATAGTGAAGGAACCTCTATCTGAGAGAAGAGTCCGTTGAAACCTTTG
GACTTGAGATAGTAATCAAGGGCGTCGTTAGTCGCACCGAGAGCGGCGGAGGAAACACTTCCACC
AACGCCGGCTGGNNNNNNGGCGGAGGAAGCACTTCCACCAACGCCGGCTGTAGCACCAGCACCGGA
GGCGACATCGGAGCAACAATTCCCCATAATTTTTGATTTCCCAACAAAAAAAACTGCGGATTCAA
AAGTACCTGGATCGAACAACGAGCTCAAAAA
>17C_BF11_CONTIG_291_p44   24 pairs of NGS reads, 0.21%
CCCCACGAGAGATAGATGATAGTGAAGGAACCTCTATCTGAGAGAAGAGTCCGTTGAAACCTTTG
GACTTGAGATAGTAATCAAGGGCGTCGTTAGTCGCACCGAGAGCGGCGGAGGAAACACTTCCACC
AACGCCGGCTGGNNNNNNGGCGGAGGAAGCACTTCCACCAACGCCGGCTGTAGCACCAGCACCGGA
GGCGACATCGGAGCAACAATTCCCCATAATTTTTGATTTCCCAACAAAAAAAACTGCGGATTCAA
AAGTACCTGGATCGAACAACGAGCTCAAAAA
>17C_BF11_CONTIG_222_p45   23 pairs of NGS reads, 0.2%
AAAAACGAGAGATAGATGATAGTGAAGGAACCTCTATCTGAGAGAAGAGTCCGTTGAAACCTTTG
GACTTGAGATAGTAAccaagggcgtcgttagtcgcaccgccggctgtagcaccagcaccggaggc
gacatcggagcaACAATTCCCCATAATTTTTGATTTCCCAACAAAAAAAACTGCGGATTCAAAAG
TACCTGGATCGAACAACGAGCTCAAAA
>17C_BF11_CONTIG_291_p46   23 pairs of NGS reads, 0.2%
CCCCACGAGAGATAGATGATAGTGAAGGAACCTCTATCTGAGAGAAGAGTCCGTTGAAACCTTTG
GACTTGAGATAGTAATCAAGGGCGTCGTTAGTCGCACCGAGAGCGGCGGAGGAAGCACTTCCACC
CACGCCGGCCGGNNNNNNGGCGGAGGAAGCACTTCCACCAACGCCGGCTGTAGCACCAGCACCGGA
GGCGACATCGGAGCAACAATTCCCCATAATTTTTGATTTCCCAACAAAAAAAACTGCGGATTCAA
AAGTACCTGGATCGAACAACGAGCTCAAAAA
>17C_BF11_CONTIG_289_p47   23 pairs of NGS reads, 0.2%
TTTTACGAGAGATAGATGATAGTGAAGGAACCTCTATCTGAGAGAAGAGTCCGTTGAAACCTTTG
GACTTGAGATAGTAATCAAGGGCGTCGTTAGTCGCACCGAGAGCGGCGGAGGAAACACTTCCACC
AACGCCGGCTGGNNNNNNGGAGCGGCGGAGGAAGCACTTCCACCAACGCCGGCTGTAGCACCAGCA
CCGGAGGCGACATCGGAGCAACAATTCCCCATAATTTTTGATTTCCCAACAAAAAAAACTGCGGA
TTCAAAAGTACCTGGATCGAACAACTTTT
>17C_BF11_CONTIG_291_p48   23 pairs of NGS reads, 0.2%
CCCCACGAGAGATAGATGATAGTGAAGGAACCTCTATCTGAGAGAAGAGTCCGTTGAAACCTTTG
GACTTGAGATAGTAATCAAGGGCGTCGTTAGTCGCACCGAGAGCGGCGGAGGAAGCACTTCCCCC
AACGCCGGCTGGNNNNNNGGCGGAGGAAGCACTTCCACCAACGCCGGCTGTAGCACCAGCACCGGA
GGCGACATCGGAGCAACAATTCCCCATAATTTTTGATTTCCCAACAAAAAAAACTGCGGATTCAA
AAGTACCTGGATCGAACAACGAGCTCAAAAA
>17C_BF11_CONTIG_214_p49   22 pairs of NGS reads, 0.19%
TTTTACGAGAGATAGATGATAGTGAAGGAACCTCTATCTGAGAGAAGAGTCCGTTGAAACCTTTG
GACTTGAgatagtaatcaagggcgtcgttagtcgcaccgagcaccagcaccggaggcgacatcgg
agcaacaattccCCATAATTTTTGATTTCCCAACAAAAAAAACTGCGGATTCAAAAGTACCTGGA
TCGAACAACGAGCTCAAAA
>17C_BF11_CONTIG_291_p50   22 pairs of NGS reads, 0.19%
CCCCACGAGAGATAGATGATAGTGAAGGAACCTCTATCTGAGAGAAGAGTCCGTTGAAACCTTTG
GACTTGAGATAGTAATCAAGGGCGTCGTTAGTCGCACCGAGAGCGGCGGAGGAAGCACTTCCACC

CACGCCGGCTGGNNNNNGGCGGAGGAAGCACTTCCACCAACGCCGGCTGTAGCACCAGCACCGGA
GGCGACATCGGAGCAACAATTCCCCATAATTTTTGATTTCCCAACAAAAAAAACTGCGGATTCAA
AAGTACCTGGATCGAACAACGAGCTCAAAAA
>17C_BF11_CONTIG_290_p51   21 pairs of NGS reads, 0.18%
TTTTACGAGAGATAGATGATAGTGAAGGAACCTCTATCTGAGAGAAGAGTCCGTTGAAACCTTTG
GACTTGAGATAGTAATCAAGGGCGTCGTTAGTCGCACCGAGAGCGGCGGAGGAAGCACTTCCCCC
AACGCCGGCCGGNNNNNGAGAGCGGCGGAGGAAGCACTTCCACCAACGCCGGCTGTAGCACCAGC
ACCGGAGGCGACATCGGAGCAACAATTCCCCATAATTTTTGATTTCCCAACAAAAAAAACTGCGG
ATTCAAAAGTACCTGGATCGAACAACTTTT
>17C_BF11_CONTIG_217_p52   21 pairs of NGS reads, 0.18%
AAAAACGAGAGATAGATGATAGTGAAGGAACCTCTATCTGAGAGAAGAGTCCGTTGAAACCTTTG
GACTTGAGATagtaatcaagggcgtcgttagtcgcaccgaccggctgtagcaccagcaccggagg
cgacatcggagcaacaaTTCCCCATAATTTTTGATTTCCCAACAAAAAAAACTGCGGATTCAAAA
GTACCTGGATCGAACAACTTTT
>17C_BF11_CONTIG_217_p53   21 pairs of NGS reads, 0.18%
TTTTACGAGAGATAGATGATAGTGAAGGAACCTCTATCTGAGAGAAGAGTCCGTTGAAACCTTTG
GACTTGAGATagtaatcaagggcgtcgttagtcgcaccgaccggctgtagcaccagcaccggagg
cgacatcggagcaacAATTCCCCATAATTTTTGATTTCCCAACAAAAAAAACTGCGGATTCAAAA
GTACCTGGATCGAACAACTTTT
>17C_BF11_CONTIG_291_p54   21 pairs of NGS reads, 0.18%
CCCCACGAGAGATAGATGATAGTGAAGGAACCTCTATCTGAGAGAAGAGTCCGTTGAAACCTTTG
GACTTGAGATAGTAATCAAGGGCGTCGTTAGTCGCACCGAGAGCGGCGGAGGAAACACTTCCACC
AACGCCGGCCGTNNNNNGGCGGAGGAAGCACTTCCACCAACGCCGGCTGTAGCACCAGCACCGGA
GGCGACATCGGAGCAACAATTCCCCATAATTTTTGATTTCCCAACAAAAAAAACTGCGGATTCAA
AAGTACCTGGATCGAACAACGAGCTCAAAAA
>17C_BF11_CONTIG_291_p55   21 pairs of NGS reads, 0.18%
GGGGACGAGAGATAGATGATAGTGAAGGAACCTCTATCTGAGAGAAGAGTCCGTTGAAACCTTTG
GACTTGAGATAGTAATCAAGGGCGTCGTTAGTCGCACCGAGAGCGGCGGAGGAAGCACTTCCACC
AACGCCGGCGGGNNNNNGGCGGAGGAAGCACTTCCACCAACGCCGGCTGTAGCACCAGCACCGGA
GGCGACATCGGAGCAACAATTCCCCATAATTTTTGATTTCCCAACAAAAAAAACTGCGGATTCAA
AAGTACCTGGATCGAACAACGAGCTCAAAAA
>17C_BF11_CONTIG_220_p56   20 pairs of NGS reads, 0.17%
TTTTACGAGAGATAGATGATAGTGAAGGAACCTCTATCTGAGAGAAGAGTCCGTTGAAACCTTTG
GACTTGAGATAGTaatcaagggcgtcgttagtcgcacccggctgtagcaccagcaccggaggcga
catcggagcaacAATTCCCCATAATTTTTGATTTCCCAACAAAAAAAACTGCGGATTCAAAAGTA
CCTGGATCGAACAACGAGCTCAAAA
>17C_BF11_CONTIG_221_p57   20 pairs of NGS reads, 0.17%
AAAAACGAGAGATAGATGATAGTGAAGGAACCTCTATCTGAGAGAAGAGTCCGTTGAAACCTTTG
GACTTGAGATAGTAatcaagggcgtcgttagtcgcaccccggctgtagcaccagcaccggaggcg
acatcggagcaaCAATTCCCCATAATTTTTGATTTCCCAACAAAAAAAACTGCGGATTCAAAAGT
ACCTGGATCGAACAACGAGCTCAAAA
>17C_BF11_CONTIG_291_p58   20 pairs of NGS reads, 0.17%
GGGGACGAGAGATAGATGATAGTGAAGGAACCTCTATCTGAGAGAAGAGTCCGTTGAAACCTTTG
GACTTGAGATAGTAATCAAGGGCGTCGTTAGTCGCACCGAGAGCGGCGGAGGAAGCACTTCCACC
AACGCCCGCCGGNNNNNGGCGGAGGAAGCACTTCCACCAACGCCGGCTGTAGCACCAGCACCGGA
GGCGACATCGGAGCAACAATTCCCCATAATTTTTGATTTCCCAACAAAAAAAACTGCGGATTCAA
AAGTACCTGGATCGAACAACGAGCTCAAAAA
>17C_BF11_CONTIG_245_p59   20 pairs of NGS reads, 0.17%
TTTACGAGAGATAGATGATAGTGAAGGAACCTCTATCTGAGAGAAGAGTCCATTGAAACCTTTGG
ACTTGAGATAGTAATCAAGGGCGTCGTTAGTCGCACCGagagcggcggaggaagcacttccacca
acgccggctgtaGCACCAGCACCGGAGGCGACATCGGAGCAACAATTCCCCATAATTTTTGATTT
CCCAACAAAAAAAATTGCGGATTCAAAAGTACCTGGATCGAACAACTTTT

>17C_BF11_CONTIG_291_p60   19 pairs of NGS reads, 0.16%
CCCCACGAGAGATAGATGATAGTGAAGGAACCTCTATCTGAGAGAAGAGTCCGTTGAAACCTTTG
GACTTGAGATAGTAATCAAGGGCGTCGTTAGTCGCACCGAGAGCGGCGGAGGAAGCACTTCCACC
AACGCCGGCGGGNNNNNGGCGGAGGAAGCACTTCCACCAACGCCGGCTGTAGCACCAGCACCGGA
GGCGACATCGGAGCAACAATTCCCCATAATTTTTGATTTCCCAACAAAAAAAACTGCGGATTCAA
AAGTACCTGGATCGAACAACGAGCTCAAAAA
>17C_BF11_CONTIG_290_p61   19 pairs of NGS reads, 0.16%
TTTTACGAGAGATAGATGATAGTGAAGGAACCTCTATCTGAGAGAAGAGTCCGTTGAAACCTTTG
GACTTGAGATAGTAATCAAGGGCGTCGTTAGTCGCACCGAGAGCGGCGGAGGAAACACTTCCACC
AACGCCGGCCGGNNNNNGAGAGCGGCGGAGGAAGCACTTCCACCAACGCCGGCTGTAGCACCAGC
ACCGGAGGCGACATCGGAGCAACAATTCCCCATAATTTTTGATTTCCCAACAAAAAAAACTGCGG
ATTCAAAAGTACCTGGATCGAACAACTTTT
>17C_BF11_CONTIG_254_p62   18 pairs of NGS reads, 0.15%
AAAAACGAGAGATAGATGATAGTGAAGGAACCTCTATCTGAGAGAAGAGTCCGTTGAAACCTTTG
GACTTGAGATAGTAATCAAGGGCGTCGTTAGTCGCACCGAAGAGCggcggaggaagcacttccac
caacgccggctgTAGCACCAGCACCGGAGGCGACATCGGAGCAACAATTCCCCATAATTTTTGAT
TTCCCAACAAAAAAAACTGCGGATTCAAAAGTACCTGGATCGAACAACGAGCTCAAAAA
>17C_BF11_CONTIG_291_p63   18 pairs of NGS reads, 0.15%
CCCCACGAGAGATAGATGATAGTGAAGGAACCTCTATCTGAGAGAAGAGTCCGTTGAAACCTTTG
GACTTGAGATAGTAATCAAGGGCGTCGTTAGTCGCACCGAGAGCGGCGGAGGGAGCACTTCCACC
AACGCCGGCCGGNNNNNGGCGGAGGAAGCACTTCCACCAACGCCGGCTGTAGCACCAGCACCGGA
GGCGACATCGGAGCAACAATTCCCCATAATTTTTGATTTCCCAACAAAAAAAACTGCGGATTCAA
AAGTACCTGGATCGAACAACGAGCTCAAAAA
>17C_BF11_CONTIG_291_p64   18 pairs of NGS reads, 0.15%
GGGGACGAGAGATAGATGATAGTGAAGGAACCTCTATCTGAGAGAAGAGTCCGTTGAAACCTTTG
GACTTGAGATAGTAATCAAGGGCGTCGTTAGTCGCACCGAGAGCGGCGGAGGAAGCACCTCCACC
AACGCCGGCTGGNNNNNGGCGGAGGAAGCACTTCCACCAACGCCGGCTGTAGCACCAGCACCGGA
GGCGACATCGGAGCAACAATTCCCCATAATTTTTGATTTCCCAACAAAAAAAACTGCGGATTCAA
AAGTACCTGGATCGAACAACGAGCTCAAAAA
>17C_BF11_CONTIG_289_p65   18 pairs of NGS reads, 0.15%
TTTTACGAGAGATAGATGATAGTGAAGGAACCTCTATCTGAGAGAAGAGTCCGTTGAAACCTTTG
GACTTGAGATAGTAATCAAGGGCGTCGTTAGTCGCACCGAGAGCGGCGGAGGAAGCACCTCCACC
AACGCCGGCTGGNNNNNGGAGCGGCGGAGGAAGCACTTCCACCAACGCCGGCTGTAGCACCAGCA
CCGGAGGCGACATCGGAGCAACAATTCCCCATAATTTTTGATTTCCCAACAAAAAAAACTGCGGA
TTCAAAAGTACCTGGATCGAACAACTTTT
>17C_BF11_CONTIG_291_p66   18 pairs of NGS reads, 0.15%
GGGGACGAGAGATAGATGATAGTGAAGGAACCTCTATCTGAGAGAAGAGTCCGTTGAAACCTTTG
GACTTGAGATAGTAATCAAGGGCGTCGTTAGTCGCACCGAGAGCGGCGGAGGGAGCACTTCCACC
AACGCCGGCTGGNNNNNGGCGGAGGAAGCACTTCCACCAACGCCGGCTGTAGCACCAGCACCGGA
GGCGACATCGGAGCAACAATTCCCCATAATTTTTGATTTCCCAACAAAAAAAACTGCGGATTCAA
AAGTACCTGGATCGAACAACGAGCTCAAAAA
>17C_BF11_CONTIG_291_p67   18 pairs of NGS reads, 0.15%
CCCCACGAGAGATAGATGATAGTGAAGGAACCTCTATCTGAGAGAAGAGTCCGTTGAAACCTTTG
GACTTGAGATAGTAATCAAGGGCGTCGTTAGTCGCACCGAGAGCGGCGGAGGGAGCACTTCCACC
AACGCCGGCTGGNNNNNGGCGGAGGAAGCACTTCCACCAACGCCGGCTGTAGCACCAGCACCGGA
GGCGACATCGGAGCAACAATTCCCCATAATTTTTGATTTCCCAACAAAAAAAACTGCGGATTCAA
AAGTACCTGGATCGAACAACGAGCTCAAAAA
>17C_BF11_CONTIG_224_p68   18 pairs of NGS reads, 0.15%
AAAAACGAGAGATAGATGATAGTGAAGGAACCTCTATCTGAGAGAAGAGTCCGTTGAAACCTTTG
GACTTGAGATAGTAATCaagggcgtcgttagtcgcaccgaccggctgtagcaccagcaccggagg
cgacatcggagcaacaaTTCCCCATAATTTTTGATTTCCCAACAAAAAAAAACTGCGGATTCAAA
AGTACCTGGATCGAACAACGAGCTCAAAA

>17C_BF11_CONTIG_289_p69   17 pairs of NGS reads, 0.14%
TTTTACGAGAGATAGATGATAGTGAAGGAACCTCTATCTGAGAGAAGAGTCCGTTGAAACCTTTG
GACTTGAGATAGTAATCAAGGGCGTCGTTAGTCGCACCGAGAGCGGCGGAGGAAACACTTCCACC
AACGCCGGCCGGNNNNNGGAGCGGCGGAGGAAGCACTTCCACCAACGCCGGCTGTAGCACCAGCA
CCGGAGGCGACATCGGAGCAACAATTCCCCATAATTTTTGATTTCCCAACAAAAAAAACTGCGGA
TTCAAAAGTACCTGGATCGAACAACTTTT
>17C_BF11_CONTIG_292_p70   17 pairs of NGS reads, 0.14%
TTTTACGAGAGATAGATGATAGTGAAGGAACCTCTATCTGAGAGAAGAGTCCGTTGAAACCTTTG
GACTTGAGATAGTAATCAAGGGCGTCGTTAGTCGCACCGAGAGCGGCGGAGGAAGCACTTCCACC
AACGCCGGCCGGANNNNNGGCGGAGGAAGCACTTCCACCAACGCCGGCTGTAGCACCAGCACCGG
AGGCGACATCGGAGCAACAATTCCCCATAATTTTTGATTTCCCAACAAAAAAAACTGCGGATTCA
AAAGTACCTGGATCGAACAACGAGCTCAAAAA
>17C_BF11_CONTIG_291_p71   17 pairs of NGS reads, 0.14%
CCCCACGAGAGATAGATGATAGTGAAGGAACCTCTATCTGAGAGAAGAGTCCGTTGAAACCTTTG
GACTTGAGATAGTAATCAAGGGCGTCGTTAGTCGCACCGAGAGCGGCGGAGGAAGCACTTCCACC
AAAGCCGGCTGGNNNNNGGCGGAGGAAGCACTTCCACCAACGCCGGCTGTAGCACCAGCACCGGA
GGCGACATCGGAGCAACAATTCCCCATAATTTTTGATTTCCCAACAAAAAAAACTGCGGATTCAA
AAGTACCTGGATCGAACAACGAGCTCAAAAA
>17C_BF11_CONTIG_291_p72   17 pairs of NGS reads, 0.14%
GGGGACGAGAGATAGATGATAGTGAAGGAACCTCTATCTGAGAGAAGAGTCCGTTGAAACCTTTG
GACTTGAGATAGTAATCAAGGGCGTCGTTAGTCGCACCGAGAGCGGCGGAGGAAGCACCTCCACC
AACGCCGGCCGGNNNNNGGCGGAGGAAGCACTTCCACCAACGCCGGCTGTAGCACCAGCACCGGA
GGCGACATCGGAGCAACAATTCCCCATAATTTTTGATTTCCCAACAAAAAAAACTGCGGATTCAA
AAGTACCTGGATCGAACAACGAGCTCAAAAA
>17C_BF11_CONTIG_223_p73   17 pairs of NGS reads, 0.14%
AAAAACGAGAGATAGATGATAGTGAAGGAACCTCTATCTGAGAGAAGAGTCCGTTGAAACCTTTG
GACTTGAGATAGTAATcaagggcgtcgttagtcgccccgaccggctgtagcaccagcaccggagg
cgacatcggagcaacaaTTCCCCATAATTTTTGATTTCCCAACAAAAAAAACTGCGGATTCAAAA
GTACCTGGATCGAACAACGAGCTCAAAA
>17C_BF11_CONTIG_223_p74   17 pairs of NGS reads, 0.14%
TTTTACGAGAGATAGATGATAGTGAAGGAACCTCTATCTGAGAGAAGAGTCCGTTGAAACCTTTG
GACTTGAGATAGTAATcaagggcgtcgttagtcgccccgaccggctgtagcaccagcaccggagg
cgacatcggagcaacAATTCCCCATAATTTTTGATTTCCCAACAAAAAAAACTGCGGATTCAAAA
GTACCTGGATCGAACAACGAGCTCAAAA
>17C_BF11_CONTIG_254_p75   17 pairs of NGS reads, 0.14%
GGGGACGAGAGATAGATGATAGTGAAGGAACCTCTATCTGAGAGAAGAGTCCGTTGAAACCTTTG
GACTTGAGATAGTAATCAAGGGCGTCGTTAGTCGCACCGATGAGCggcggaggaagcacttccac
caacgccggctgTAGCACCAGCACCGGAGGCGACATCGGAGCAACAATTCCCCATAATTTTTGAT
TTCCCAACAAAAAAAACTGCGGATTCAAAAGTACCTGGATCGAACAACGAGCTCAAAAA
>17C_BF11_CONTIG_222_p76   16 pairs of NGS reads, 0.14%
AAAAACGAGAGATAGATGATAGTGAAGGAACCTCTATCTGAGAGAAGAGTCCGTTGAAACCTTTG
GACTTGAGATAGTAAccaagggcgtcgttagtcgcaccgacggctgtagcaccagcaccggaggc
gacatcggagcaACAATTCCCCATAATTTTTGATTTCCCAACAAAAAAAACTGCGGATTCAAAAG
TACCTGGATCGAACAACGAGCTCAAAA
>17C_BF11_CONTIG_290_p77   16 pairs of NGS reads, 0.14%
TTTTACGAGAGATAGATGATAGTGAAGGAACCTCTATCTGAGAGAAGAGTCCGTTGAAACCTTTG
GACTTGAGATAGTAATCAAGGGCGTCGTTAGTCGCACCGAGAGCGGCGGAGGAAGAACTTCCACC
AACGCCGGCCGGNNNNNGAGAGCGGCGGAGGAAGCACTTCCACCAACGCCGGCTGTAGCACCAGC
ACCGGAGGCGACATCGGAGCAACAATTCCCCATAATTTTTGATTTCCCAACAAAAAAAACTGCGG
ATTCAAAAGTACCTGGATCGAACAACTTTT
>17C_BF11_CONTIG_254_p78   16 pairs of NGS reads, 0.14%
AAAAACGAGAGATAGATGATAGTGAAGGAACCTCTATCTGAGAGAAGAGTCCGTTGAAACCTTTG

GACTTGAGATAGTAATCAAGGGCGTCGTTAGTCGCACCGATGAGCggcggaggaagcacttccac
caacgccggctgTAGCACCAGCACCGGAGGCGACATCGGAGCAACAATTCCCCATAATTTTTGAT
TTCCCAACAAAAAAAACTGCGGATTCAAAAGTACCTGGATCGAACAACGAGCTCAAAAA
>17C_BF11_CONTIG_222_p79   16 pairs of NGS reads, 0.14%
AAAAACGAGAGATAGATGATAGTGAAGGAACCTCTATCTGAGAGAAGAGTCCGTTGAAACCTTTG
GACTTGAGATAGTAAacaagggcgtcgttagtcgcaccgccggctgtagcaccagcaccggaggc
gacatcggagcaACAATTCCCCATAATTTTTGATTTCCCAACAAAAAAAACTGCGGATTCAAAAG
TACCTGGATCGAACAACGAGCTCAAAA
>17C_BF11_CONTIG_254_p80   16 pairs of NGS reads, 0.14%
GGGGACGAGAGATAGATGATAGTGAAGGAACCTCTATCTGAGAGAAGAGTCCGTTGAAACCTTTG
GACTTGAGATAGTAATCAAGGGCGTCGTTAGTCGCACCGAAGAGCggcggaggaagcacttccac
caacgccggctgTAGCACCAGCACCGGAGGCGACATCGGAGCAACAATTCCCCATAATTTTTGAT
TTCCCAACAAAAAAAACTGCGGATTCAAAAGTACCTGGATCGAACAACGAGCTCAAAAA
>17C_BF11_CONTIG_291_p81   16 pairs of NGS reads, 0.14%
GGGGACGAGAGATAGATGATAGTGAAGGAACCTCTATCTGAGAGAAGAGTCCGTTGAAACCTTTG
GACTTGAGATAGTAATCAAGGGCGTCGTTAGTCGCACCGAGAGCGGCGGAGGAAGCACTTCCACC
CACGCCGGCTGGNNNNNGGCGGAGGAAGCACTTCCACCAACGCCGGCTGTAGCACCAGCACCGGA
GGCGACATCGGAGCAACAATTCCCCATAATTTTTGATTTCCCAACAAAAAAAACTGCGGATTCAA
AAGTACCTGGATCGAACAACGAGCTCAAAAA
>17C_BF11_CONTIG_290_p82   16 pairs of NGS reads, 0.14%
TTTTACGAGAGATAGATGATAGTGAAGGAACCTCTATCTGAGAGAAGAGTCCGTTGAAACCTTTG
GACTTGAGATAGTAATCAAGGGCGTCGTTAGTCGCACCGAGAGCGGCGGAGGAAGCACTTCCACC
AACCCCGGCCGGNNNNNGAGAGCGGCGGAGGAAGCACTTCCACCAACGCCGGCTGTAGCACCAGC
ACCGGAGGCGACATCGGAGCAACAATTCCCCATAATTTTTGATTTCCCAACAAAAAAAACTGCGG
ATTCAAAAGTACCTGGATCGAACAACTTTT
>17C_BF11_CONTIG_290_p83   15 pairs of NGS reads, 0.13%
TTTTACGAGAGATAGATGATAGTGAAGGAACCTCTATCTGAGAGAAGAGTCCGTTGAAACCTTTG
GACTTGAGATAGTAATCAAGGGCGTCGTTAGTCGCACCGAGAGCGGCGGAGGAAGCACTTCCACC
CACGCCGGCCGGNNNNNGAGAGCGGCGGAGGAAGCACTTCCACCAACGCCGGCTGTAGCACCAGC
ACCGGAGGCGACATCGGAGCAACAATTCCCCATAATTTTTGATTTCCCAACAAAAAAAACTGCGG
ATTCAAAAGTACCTGGATCGAACAACTTTT
>17C_BF11_CONTIG_289_p84   15 pairs of NGS reads, 0.13%
TTTTACGAGAGATAGATGATAGTGAAGGAACCTCTATCTGAGAGAAGAGTCCGTTGAAACCTTTG
GACTTGAGATAGTAATCAAGGGCGTCGTTAGTCGCACCGAGAGCGGCGGAGGAAACACTTCCACC
AACGCCGGCTGTNNNNNGGAGCGGCGGAGGAAGCACTTCCACCAACGCCGGCTGTAGCACCAGCA
CCGGAGGCGACATCGGAGCAACAATTCCCCATAATTTTTGATTTCCCAACAAAAAAAACTGCGGA
TTCAAAAGTACCTGGATCGAACAACTTTT
>17C_BF11_CONTIG_289_p85   15 pairs of NGS reads, 0.13%
TTTTACGAGAGATAGATGATAGTGAAGGAACCTCTATCTGAGAGAAGAGTCCGTTGAAACCTTTG
GACTTGAGATAGTAATCAAGGGCGTCGTTAGTCGCACCGAGAGCGGCGGAGGAAGCACTTCCCCC
AACGCCGGCTGGNNNNNGGAGCGGCGGAGGAAGCACTTCCACCAACGCCGGCTGTAGCACCAGCA
CCGGAGGCGACATCGGAGCAACAATTCCCCATAATTTTTGATTTCCCAACAAAAAAAACTGCGGA
TTCAAAAGTACCTGGATCGAACAACTTTT
>17C_BF11_CONTIG_289_p86   15 pairs of NGS reads, 0.13%
CCCCACGAGAGATAGATGATAGTGAAGGAACAACGAGCTCAAAAAGGATTCAAAAGTACCTGGAT
CGAACAACGAGCTCAAAAAACATTGCGAGATCGGAAGAGCGGTTCAGCAGGAATGCCGAGACCGA
TCTCGTATGCCGNNNNNGGCGACCACCGAGATCTACACTCTTTCCCTACACGACGCTCTTCCGAT
CTCGCAATGTTCCCCACGAGAGATAGATGATAGTGAAGGAACAACGAGCTCAAAAAGGATTCAAA
AGTACCTGGATCGAACAACGAGCTCAAAA
>17C_BF11_CONTIG_291_p87   15 pairs of NGS reads, 0.13%
CCCCACGAGAGATAGATGATAGTGAAGGAACCTCTATCTGAGAGAAGAGTCCGTTGAAACCTTTG
GACTTGAGATAGTAATCAAGGGCGTCGTTAGTCGCACCGAGAGCGGCGGAGGAAGCACTTCCACC

AACGCCCGCCGGNNNNNGGCGGAGGAAGCACTTCCACCAACGCCGGCTGTAGCACCAGCACCGGA
GGCGACATCGGAGCAACAATTCCCCATAATTTTTGATTTCCCAACAAAAAAAACTGCGGATTCAA
AAGTACCTGGATCGAACAACGAGCTCAAAAA
>17C_BF11_CONTIG_291_p88   15 pairs of NGS reads, 0.13%
GGGGACGAGAGATAGATGATAGTGAAGGAACCTCTATCTGAGAGAAGAGTCCGTTGAAACCTTTG
GACTTGAGATAGTAATCAAGGGCGTCGTTAGTCGCACCGAGAGCGGCGGAGGAAGCACTTCCACC
AAAGCCGGCCGGNNNNNGGCGGAGGAAGCACTTCCACCAACGCCGGCTGTAGCACCAGCACCGGA
GGCGACATCGGAGCAACAATTCCCCATAATTTTTGATTTCCCAACAAAAAAAACTGCGGATTCAA
AAGTACCTGGATCGAACAACGAGCTCAAAAA
>17C_BF11_CONTIG_291_p89   15 pairs of NGS reads, 0.13%
GGGGACGAGAGATAGATGATAGTGAAGGAACCTCTATCTGAGAGAAGAGTCCGTTGAAACCTTTG
GACTTGAGATAGTAATCAAGGGCGTCGTTAGTCGCACCGAGAGCGGCGGAGGAAGCACTTCCACC
AAAGCCGGCTGGNNNNNGGCGGAGGAAGCACTTCCACCAACGCCGGCTGTAGCACCAGCACCGGA
GGCGACATCGGAGCAACAATTCCCCATAATTTTTGATTTCCCAACAAAAAAAACTGCGGATTCAA
AAGTACCTGGATCGAACAACGAGCTCAAAAA
>17C_BF11_CONTIG_290_p90   14 pairs of NGS reads, 0.12%
TTTTACGAGAGATAGATGATAGTGAAGGAACCTCTATCTGAGAGAAGAGTCCGTTGAAACCTTTG
GACTTGAGATAGTAATCAAGGGCGTCGTTAGTCGCACCGAGAGCGGCGGAGGAAGCACTTCCACC
AAAGCCGGCCGGNNNNNGAGAGCGGCGGAGGAAGCACTTCCACCAACGCCGGCTGTAGCACCAGC
ACCGGAGGCGACATCGGAGCAACAATTCCCCATAATTTTTGATTTCCCAACAAAAAAAACTGCGG
ATTCAAAAGTACCTGGATCGAACAACTTTT
>17C_BF11_CONTIG_289_p91   14 pairs of NGS reads, 0.12%
TTTTACGAGAGATAGATGATAGTGAAGGAACCTCTATCTGAGAGAAGAGTCCGTTGAAACCTTTG
GACTTGAGATAGTAATCAAGGGCGTCGTTAGTCGCACCGAGAGCGGCGGAGGAAGCACTTCCACC
AACGCCGGCGGGNNNNNGGAGCGGCGGAGGAAGCACTTCCACCAACGCCGGCTGTAGCACCAGCA
CCGGAGGCGACATCGGAGCAACAATTCCCCATAATTTTTGATTTCCCAACAAAAAAAACTGCGGA
TTCAAAAGTACCTGGATCGAACAACTTTT
>17C_BF11_CONTIG_291_p92   14 pairs of NGS reads, 0.12%
CCCCACGAGAGATAGATGATAGTGAAGGAACCTCTATCTGAGAGAAGAGTCCGTTGAAACCTTTG
GACTTGAGATAGTAATCAAGGGCGTCGTTAGTCGCACCGAGAGCGGCGGAGGAAGCACTTCCACC
AACCCCGGCTGGNNNNNGGCGGAGGAAGCACTTCCACCAACGCCGGCTGTAGCACCAGCACCGGA
GGCGACATCGGAGCAACAATTCCCCATAATTTTTGATTTCCCAACAAAAAAAACTGCGGATTCAA
AAGTACCTGGATCGAACAACGAGCTCAAAAA
>17C_BF11_CONTIG_291_p93   14 pairs of NGS reads, 0.12%
CCCCACGAGAGATAGATGATAGTGAAGGAACCTCTATCTGAGAGAAGAGTCCGTTGAAACCTTTG
GACTTGAGATAGTAATCAAGGGCGTCGTTAGTCGCACCGAGAGCGGCGGAGGAAGCACCTCCACC
AACGCCGGCTGGNNNNNGGCGGAGGAAGCACTTCCACCAACGCCGGCTGTAGCACCAGCACCGGA
GGCGACATCGGAGCAACAATTCCCCATAATTTTTGATTTCCCAACAAAAAAAACTGCGGATTCAA
AAGTACCTGGATCGAACAACGAGCTCAAAAA
>17C_BF11_CONTIG_289_p94   14 pairs of NGS reads, 0.12%
TTTTACGAGAGATAGATGATAGTGAAGGAACCTCTATCTGAGAGAAGAGTCCGTTGAAACCTTTG
GACTTGAGATAGTAATCAAGGGCGTCGTTAGTCGCACCGAGAGCGGCGGAGGGAGCACTTCCACC
AACGCCGGCTGTNNNNNGGAGCGGCGGAGGAAGCACTTCCACCAACGCCGGCTGTAGCACCAGCA
CCGGAGGCGACATCGGAGCAACAATTCCCCATAATTTTTGATTTCCCAACAAAAAAAACTGCGGA
TTCAAAAGTACCTGGATCGAACAACTTTT
>17C_BF11_CONTIG_290_p95   13 pairs of NGS reads, 0.11%
TTTTACGAGAGATAGATGATAGTGAAGGAACCTCTATCTGAGAGAAGAGTCCGTTGAAACCTTTG
GACTTGAGATAGTAATCAAGGGCGTCGTTAGTCGCACCGAGAGCGGCGGAGGGAGCACTTCCACC
AACGCCGGCCGGNNNNNGAGAGCGGCGGAGGAAGCACTTCCACCAACGCCGGCTGTAGCACCAGC
ACCGGAGGCGACATCGGAGCAACAATTCCCCATAATTTTTGATTTCCCAACAAAAAAAACTGCGG
ATTCAAAAGTACCTGGATCGAACAACTTTT
>17C_BF11_CONTIG_289_p96   13 pairs of NGS reads, 0.11%

TTTTACGAGAGATAGATGATAGTGAAGGAACCTCTATCTGAGAGAAGAGTCCGTTGAAACCTTTG
GACTTGAGATAGTAATCAAGGGCGTCGTTAGTCGCACCGAGAGCGGCGGAGGGAGCACTTCCACC
AACGCCGGCTGGNNNNNGGAGCGGCGGAGGAAGCACTTCCACCAACGCCGGCTGTAGCACCAGCA
CCGGAGGCGACATCGGAGCAACAATTCCCCATAATTTTTGATTTCCCAACAAAAAAAACTGCGGA
TTCAAAAGTACCTGGATCGAACAACTTTT
>17C_BF11_CONTIG_223_p97   13 pairs of NGS reads, 0.11%
TTTTACGAGAGATAGATGATAGTGAAGGAACCTCTATCTGAGAGAAGAGTCCGTTGAAACCTTTG
GACTTGAGATAGTAATcaagggggtcgttagtcgcaccgaccggctgtagcaccagcaccggagg
cgacatcggagcaacAATTCCCCATAATTTTTGATTTCCCAACAAAAAAAACTGCGGATTCAAAA
GTACCTGGATCGAACAACGAGCTCAAAA
>17C_BF11_CONTIG_224_p98   13 pairs of NGS reads, 0.11%
AAAAACGAGAGATAGATGATAGTGAAGGAACCTCTATCTGAGAGAAGAGTCCGTTGAAACCTTTG
GACTTGAGATAGTAATCaagggcgtcgttagtcgcaccgatccggctgtagcaccagcaccggag
gcgacatcggagCAACAATTCCCCATAATTTTTGATTTCCCAACAAAAAAAACTGCGGATTCAAA
AGTACCTGGATCGAACAACGAGCTCAAAA
>17C_BF11_CONTIG_291_p99   12 pairs of NGS reads, 0.1%
CCCCACGAGAGATAGATGATAGTGAAGGAACCTCTATCTGAGAGAAGAGTCCGTTGAAACCTTTG
GACTTGAGATAGTAATCAAGGGCGTCGTTAGTCGCACCGAGAGCGGCGGAGGAAGCACTTCCACC
AACGCCCGCTGGNNNNNGGCGGAGGAAGCACTTCCACCAACGCCGGCTGTAGCACCAGCACCGGA
GGCGACATCGGAGCAACAATTCCCCATAATTTTTGATTTCCCAACAAAAAAAACTGCGGATTCAA
AAGTACCTGGATCGAACAACGAGCTCAAAAA
>17C_BF11_CONTIG_291_p100   12 pairs of NGS reads, 0.1%
CCCCACGAGAGATAGATGATAGTGAAGGAACCTCTATCTGAGAGAAGAGTCCGTTGAAACCTTTG
GACTTGAGATAGTAATCAAGGGCGTCGTTAGTCGCACCGAGAGCGGCGGAGGAAGCACTTCCACC
AAAGCCGGCCGGNNNNNGGCGGAGGAAGCACTTCCACCAACGCCGGCTGTAGCACCAGCACCGGA
GGCGACATCGGAGCAACAATTCCCCATAATTTTTGATTTCCCAACAAAAAAAACTGCGGATTCAA
AAGTACCTGGATCGAACAACGAGCTCAAAAA
>17C_BF11_CONTIG_291_p101   12 pairs of NGS reads, 0.1%
GGGGACGAGAGATAGATGATAGTGAAGGAACCTCTATCTGAGAGAAGAGTCCGTTGAAACCTTTG
GACTTGAGATAGTAATCAAGGGCGTCGTTAGTCGCACCGAGAGCGGCGGAGGGAGCACTTCCACC
AACGCCGGCCGGNNNNNGGCGGAGGAAGCACTTCCACCAACGCCGGCTGTAGCACCAGCACCGGA
GGCGACATCGGAGCAACAATTCCCCATAATTTTTGATTTCCCAACAAAAAAAACTGCGGATTCAA
AAGTACCTGGATCGAACAACGAGCTCAAAAA
>17C_BF11_CONTIG_291_p102   12 pairs of NGS reads, 0.1%
CCCCACGAGAGATAGATGATAGTGAAGGAACCTCTATCTGAGAGAAGAGTCCGTTGAAACCTTTG
GACTTGAGATAGTAATCAAGGGCGTCGTTAGTCGCACCGAGAGCGGCGGAGGAAGCACTTCCCCC
AACGCCGGCCGGNNNNNGGCGGAGGAAGCACTTCCACCAACGCCGGCTGTAGCACCAGCACCGGA
GGCGACATCGGAGCAACAATTCCCCATAATTTTTGATTTCCCAACAAAAAAAACTGCGGATTCAA
AAGTACCTGGATCGAACAACGAGCTCAAAAA
>17C_BF11_CONTIG_291_p103   12 pairs of NGS reads, 0.1%
CCCCACGAGAGATAGATGATAGTGAAGGAACCTCTATCTGAGAGAAGAGTCCGTTGAAACCTTTG
GACTTGAGATAGTAATCAAGGGCGTCGTTAGTCGCACCGAGAGCGGCGGAGGAAGCACTTCCACC
AACCCCGGCCGTNNNNNGGCGGAGGAAGCACTTCCACCAACGCCGGCTGTAGCACCAGCACCGGA
GGCGACATCGGAGCAACAATTCCCCATAATTTTTGATTTCCCAACAAAAAAAACTGCGGATTCAA
AAGTACCTGGATCGAACAACGAGCTCAAAAA
>17C_BF11_CONTIG_289_p104   12 pairs of NGS reads, 0.1%
TTTTACGAGAGATAGATGATAGTGAAGGAACCTCTATCTGAGAGAAGAGTCCGTTGAAACCTTTG
GACTTGAGATAGTAATCAAGGGCGTCGTTAGTCGCACCGAGAGCGGCGGAGGAAGCACTTCCACC
AAAGCCGGCCGGNNNNNGGAGCGGCGGAGGAAGCACTTCCACCAACGCCGGCTGTAGCACCAGCA
CCGGAGGCGACATCGGAGCAACAATTCCCCATAATTTTTGATTTCCCAACAAAAAAAACTGCGGA
TTCAAAAGTACCTGGATCGAACAACTTTT
>17C_BF11_CONTIG_219_p105   12 pairs of NGS reads, 0.1%

AAAAACGAGAGATAGATGATAGTGAAGGAACCTCTATCTGAGAGAAGAGTCCGTTGAAACCTTTG
GACTTGAGATAGtaatcaagggcgtcgttagtcgcaccgactgtagcaccagcaccggaggcgac
atcggagcaacaATTCCCCATAATTTTTGATTTCCCAACAAAAAAAACTGCGGATTCAAAAGTAC
CTGGATCGAACAACGAGCTCAAAA
>17C_BF11_CONTIG_290_p106   12 pairs of NGS reads, 0.1%
TTTTACGAGAGATAGATGATAGTGAAGGAACCTCTATCTGAGAGAAGAGTCCGTTGAAACCTTTG
GACTTGAGATAGTAATCAAGGGCGTCGTTAGTCGCACCGAGAGCGGCGGAGGAAGCACTTCCACC
AACCCCGGCCGTANNNNNGGAGCGGCGGAGGAAGCACTTCCACCAACGCCGGCTGTAGCACCAGC
ACCGGAGGCGACATCGGAGCAACAATTCCCCATAATTTTTGATTTCCCAACAAAAAAAACTGCGG
ATTCAAAAGTACCTGGATCGAACAACTTTT
>17C_BF11_CONTIG_291_p107   12 pairs of NGS reads, 0.1%
GGGGACGAGAGATAGATGATAGTGAAGGAACCTCTATCTGAGAGAAGAGTCCGTTGAAACCTTTG
GACTTGAGATAGTAATCAAGGGCGTCGTTAGTCGCACCGAGAGCGGCGGAGGAAGCACCTCCACC
AACGCCCGCCGGNNNNNGGCGGAGGAAGCACTTCCACCAACGCCGGCTGTAGCACCAGCACCGGA
GGCGACATCGGAGCAACAATTCCCCATAATTTTTGATTTCCCAACAAAAAAAACTGCGGATTCAA
AAGTACCTGGATCGAACAACGAGCTCAAAAA

AtPDS DNA 1

>PCR4_BH5_CONTIG_212_p1   18405 pairs of NGS reads, 41.99%
CACAGAAACTGTGAACTCAATAGCCTACTTGCCTGCTTTTCCATCCATTCTTTGACTGATAAAcc
atcttgggcctcaacataagcctgaccgccgaccatggctggcaaaagtccaatagcaaacttta
ttttctctggccatgtcagcATCTCGTTGTTCCGCAAAATAGCCCAAATACCTACAACGTAAATC
CAACAGCAACAACCCCC
>PCR4_BH5_CONTIG_213_p2   15750 pairs of NGS reads, 35.93%
CACAGAAACTGTGAACTCAATAGCCTACTTGCCTGCTTTTCCATCCATTCTTTGACTGATAAACC
ATcttgggcctcaacataagcctgaccgccgaccatggctggcaaaagtccaatagcaaacttta
ttttctctggccatgtcagcATCTCGTTGTTCCGCAAAATAGCCCAAATACCTACAACGTAAATC
CAACAGCAACAACTTTTT
>PCR4_BH5_CONTIG_212_p3   6179 pairs of NGS reads, 14.09%
CACAGAAACTGTGAACTCAATAGCCTACTTGCCTGCTTTTCCATCCATTCTTTGACTGATAAACC
ATcttgggcctcaacataagcctgaccgccgaccatggctggcaaaagtccaatagcaaacttta
ttttctctggccatgtcagcATCTCGTTGTTCCGCAAAATAGCCCAAATACCTACAACGTAAATC
CAACAGCAACAACAAAA
>PCR4_BH5_CONTIG_212_p4   289 pairs of NGS reads, 0.65%
CACAGAAACTGTGAACTCAATAGCCTACTTGCCTGCTTTTCCATCCATTCTTTGACTGATAAACC
ATCTTgggcctcaacataagcctgaccgccgaccatggctggcaaaagtccaatagcaaacttta
ttttctctggccatgtcagcATCTCGTTGTTCCGCAAAATAGCCCAAATACCTACAACGTGAATC
CAACAGCAACAACTTTT
>PCR4_BH5_CONTIG_212_p5   211 pairs of NGS reads, 0.48%
CACAGAAACTGTGAACTCAATAGCCTACTTGCCTGCTTTTCCATCCATTCTTTGACTGATAAACC
ATCTTgggcctcaacataagcctgaccgccgaccatggctggcaaaagtccaatagcaaacttta
ttttctctgaccaTGTCAGCATCTCGTTGTTCCGCAAAATAGCCCAAATACCTACAACGTAAATC
CAACAGCAACAACTTTT
>PCR4_BH5_CONTIG_166_p6   214 pairs of NGS reads, 0.48%
CACAGAAACTGTGAACTCAATAGCctacttgcctgcttttccatccattctttgactgataaacc
atcttgggcctcaacataagcctgaccgccgaccacagcatctcgttgttccgcaaaatagccca
aatacctacaacgtAAATCCAACAGCAACAACCCCC
>PCR4_BH5_CONTIG_212_p7   196 pairs of NGS reads, 0.44%
CACAGAAACTGTGAACTCAATAGCCTACTTGCCTGCTTTTCCATCCATTCTTTGACTGATAAACC
ATCTTggacctcaacataagcctgaccgccgaccatggctggcaaaagtccaatagcaaacttta

ttttctctggccATGTCAGCATCTCGTTGTTCCGCAAAATAGCCCAAATACCTACAACGTAAATC
CAACAGCAACAACTTTT
>PCR4_BH5_CONTIG_212_p8   164 pairs of NGS reads, 0.37%
CACAGAAACTGTGAACTCAATAGCCTACTTGCCTGCTTTTCCATCCATTCTTTGACTGATAAACC
ATCTTgggcctcaacataagcctgaccgccgaccatggctggcaaaagtccaatagcaaacttta
ttttctctggccatgtcagcATCTCGTTGTTCCGCAAAATAGCCCAAATACCTTCAACGTAAATC
CAACAGCAACAACTTTT
>PCR4_BH5_CONTIG_164_p9   158 pairs of NGS reads, 0.36%
CACAGAAACTGTGAACTCAATAgcctacttgcctgcttttccatccattctttgactgataaacc
atcttgggcctcaacataagcctgaccgccgaccagcatctcgttgttccgcaaaatagcccaaa
tacctacaacgtAAATCCAACAGCAACAACTTTT
>PCR4_BH5_CONTIG_165_p10   158 pairs of NGS reads, 0.36%
CACAGAAACTGTGAACTCAATAGcctacttgcctgcttttccatccattctttgactgataaacc
atcttgggcctcaacataagcctgaccgccgacccagcatctcgttgttccgcaaaatagcccaa
atacctacaacgtAAATCCAACAGCAACAACCCCC
>PCR4_BH5_CONTIG_166_p11   155 pairs of NGS reads, 0.35%
CACAGAAACTGTGAACTCAATAGCctacttgcctgcttttccatccattctttgactgataaacc
atcttgggcctcaacataagcctgaccgccgaccacagcatctcgttgttccgcaaaatagccca
aatacctacaacgtAAATCCAACAGCAACAACTTTT
>PCR4_BH5_CONTIG_164_p12   149 pairs of NGS reads, 0.33%
CACAGAAACTGTGAACTCAATagcctacttgcctgcttttccatccattctttgactgataaacc
atcttgggcctcaacataagcctgaccgccgaccagcatctcgttgttccgcaaaatagcccaaa
tacctacaacgtAAATCCAACAGCAACAACCCCC
>PCR4_BH5_CONTIG_245_p13   144 pairs of NGS reads, 0.32%
AAAAGTTGTTGCTGTTGGATTTACGTGTTAACTTTTACACTTTATGGTCTCAAAACACTAAACAT
ATGGTTAATGAGGCTATATTTTGTGTAATCTTACTTAatttagacatttctgttttctgtgagt
tgtttcttgaagTGAGTAGTGCTTTGTTTGTGGTCTATAGGAGAAAAGTAAAACATTCAGAAGTC
GATATGGTGAGCTTACGAAGACGTAGGCTATTGAGTTCACAGTTTCTGTG
>PCR4_BH5_CONTIG_165_p14   137 pairs of NGS reads, 0.31%
CACAGAAACTGTGAACTCAATAGcctacttgcctgcttttccatccattctttgactgataaacc
atcttgggcctcaacataagcctgaccgccgacccagcatctcgttgttccgcaaaatagcccaa
atacctacaacgtAAATCCAACAGCAACAACTTTT
>PCR4_BH5_CONTIG_212_p15   89 pairs of NGS reads, 0.2%
CACGAAACTGTGAACTCAATAGCCTACTTGCCTGCTTTTCCATCCATTCTTTGACTGATAAACCA
Tcttgggcctcaacataagcctgaccgccgaccatggctggcaaaagtccaatagcaaactttat
tttctctggccatgtcagcatCTCGTTGTTCCGCAAAATAGCCCAAATACCTACAACGTAAATCC
AACAGCAACAACTTTTT
>PCR4_BH5_CONTIG_211_p16   85 pairs of NGS reads, 0.19%
CACGAAACTGTGAACTCAATAGCCTACTTGCCTGCTTTTCCATCCATTCTTTGACTGATAAAcca
tcttgggcctcaacataagcctgaccgccgaccatggctggcaaaagtccaatagcaaactttat
tttctctggccatgtcagcatCTCGTTGTTCCGCAAAATAGCCCAAATACCTACAACGTAAATCC
AACAGCAACAACCCCC
>PCR4_BH5_CONTIG_202_p17   65 pairs of NGS reads, 0.14%
CACAGAAACTGTGAACTCAATAGCCTACTTGCCTGCTTTTCCATCCATTCTTTgactgataaacc
atcttgggcctcaacataagcctgaccgccgaccatggctggcaaaagtccaatagcaaacttta
ttttctctggccatgtcagcATCTCGTTGTTCCGCAAAATAGCCCAAATACCTACAACGTAAATC
CAACAGC
>PCR4_BH5_CONTIG_211_p18   63 pairs of NGS reads, 0.14%
CCAGAAACTGTGAACTCAATAGCCTACTTGCCTGCTTTTCCATCCATTCTTTGACTGATAAAcca
tcttgggcctcaacataagcctgaccgccgaccatggctggcaaaagtccaatagcaaactttat
tttctctggccaTGTCAGCATCTCGTTGTTCCGCAAAATAGCCCAAATACCTACAACGTAAATCC
AACAGCAACAACCCCC

>PCR4_BH5_CONTIG_211_p19   58 pairs of NGS reads, 0.13%
CACAGAAACTGTGAACTCAATAGCCTACTTGCCTGCTTTTCCATCCATTCTTTGACTGATAAACC
ATCTTgggcctcaacataagcctgaccgccgaccatggctggcaaaagtccaatagcaaacttta
ttttctctggccATGTAGCATCTCGTTGTTCCGCAAAATAGCCCAAATACCTACAACGTAAATCC
AACAGCAACAACCCCC
>PCR4_BH5_CONTIG_212_p20   54 pairs of NGS reads, 0.12%
CACAGAAACTGTGAACTCAATAGCCTACTTGCCTGCTTTTCCATCCATTCTTTGACTGATAAACC
ATCTTgggcctcaacataagcctgaccgccgaccatggctggcaaaagtccaatagcaaacttta
ttttctctggccatgtcagcATCTCGTTGTTCCGCAAAATAGCCCAAATACCTGCAACGTAAATC
CAACAGCAACAACTTTT
>PCR4_BH5_CONTIG_213_p21   48 pairs of NGS reads, 0.1%
CACAGAAACTGTGAACTCAATAGCCTACTTGCCTGCTTTTCCATCCATTCTTTGACTGATAAACC
ATCTTGggcctcaacataagcctgaccgccgaccaatggctggcaaaagtccaatagcaaacttt
atttctctggcCATGTCAGCATCTCGTTGTTCCGCAAAATAGCCCAAATACCTACAACGTAAAT
CCAACAGCAACAACCCCC

AtPDS DNA 2

>PCR5_CF12_CONTIG_213_p1   36820 pairs of NGS reads, 55.4%
CACAGAAACTGTGAACTCAATAGCCTACTTGCCTGCTTTTCCATCCATTCTTTGACTGATAAAcc
atcttgggcctcaacataagcctgaccgccgaccatggctggcaaaagtccaatagcaaacttta
ttttctctggccatgtcagcaTCTCGTTGTTCCGCAAAATAGCCCAAATACCTACAACGTAAATC
CAACAGCAACAACCCCCC
>PCR5_CF12_CONTIG_213_p2   13100 pairs of NGS reads, 19.71%
CACAGAAACTGTGAACTCAATAGCCTACTTGCCTGCTTTTCCATCCATTCTTTGACTGATAAACC
ATcttgggcctcaacataagcctgaccgccgaccatggctggcaaaagtccaatagcaaacttta
ttttctctggccatgtcagcaTCTCGTTGTTCCGCAAAATAGCCCAAATACCTACAACGTAAATC
CAACAGCAACAACTTTTT
>PCR5_CF12_CONTIG_212_p3   7151 pairs of NGS reads, 10.76%
CACAGAAACTGTGAACTCAATAGCCTACTTGCCTGCTTTTCCATCCATTCTTTGACTGATAAACC
ATcttgggcctcaacataagcctgaccgccgaccatggctggcaaaagtccaatagcaaacttta
ttttctctggccatgtcagcaTCTCGTTGTTCCGCAAAATAGCCCAAATACCTACAACGTAAATC
CAACAGCAACAACAAAA
>PCR5_CF12_CONTIG_164_p4   837 pairs of NGS reads, 1.25%
CACAGAAACTGTGAACTCAATagcctacttgcctgcttttccatccattctttgactgataaacc
atcttgggcctcaacataagcctgaccgccgaccagcatctcgttgttccgcaaaatagcccaaa
tacctacaacgtaaatCCAACAGCAACAACCCCC
>PCR5_CF12_CONTIG_165_p5   797 pairs of NGS reads, 1.19%
CACAGAAACTGTGAACTCAATAgcctacttgcctgcttttccatccattctttgactgataaacc
atcttgggcctcaacataagcctgaccgccgacccagcatctcgttgttccgcaaaatagcccaa
atacctacaacgtaaaTCCAACAGCAACAACCCCC
>PCR5_CF12_CONTIG_166_p6   744 pairs of NGS reads, 1.11%
CACAGAAACTGTGAACTCAATAGcctacttgcctgcttttccatccattctttgactgataaacc
atcttgggcctcaacataagcctgaccgccgaccacagcatctcgttgttccgcaaaatagccca
aatacctacaacgtaaATCCAACAGCAACAACCCCC
>PCR5_CF12_CONTIG_212_p7   1539 pairs of NGS reads, 2.31%
CACAGAAACTGTGAACTCAATAGCCTACTTGCCTGCTTTTCCATCCATTCTTTGACTGATAAACC
ATCTTgggcctcaacataagcctgaccgccgaccatggctggtaaaagtccaatagcaaacttta
ttttctctggccaTGTCAGCATCTCGTTGTTCCGCAAAATAGCCCAAATACCTACAACGTAAATC
CAACAGCAACAACCCCC
>PCR5_CF12_CONTIG_212_p8   354 pairs of NGS reads, 0.53%

CACAGAAACTGTGAACTCAATAGCCTACTTGCCTGCTTTTCCATCCATTCTTTGACTGATAAACC
ATCTTgggcctcaacataagcctgaccgccgaccatggctggcaaaagtccaatagcaaacttta
ttttctctggccatgtcagcaTCTCGTTGTTCCGCAAAATAGCCCAAATACCTACAACGTAAAGC
CAACAGCAACAACCCCC
>PCR5_CF12_CONTIG_164_p9   295 pairs of NGS reads, 0.44%
CACAGAAACTGTGAACTCAATagcctacttgcctgcttttccatccattctttgactgataaacc
atcttgggcctcaacataagcctgaccgccgaccagcatctcgttgttccgcaaaatagcccaaa
tacctacaacgtaaatCCAACAGCAACAACTTTT
>PCR5_CF12_CONTIG_165_p10   277 pairs of NGS reads, 0.41%
CACAGAAACTGTGAACTCAATAGcctacttgcctgcttttccatccattctttgactgataaacc
atcttgggcctcaacataagcctgaccgccgacccagcatctcgttgttccgcaaaatagcccaa
atacctacaacgtaaaTCCAACAGCAACAACTTTT
>PCR5_CF12_CONTIG_166_p11   250 pairs of NGS reads, 0.37%
CACAGAAACTGTGAACTCAATAGcctacttgcctgcttttccatccattctttgactgataaacc
atcttgggcctcaacataagcctgaccgccgaccacagcatctcgttgttccgcaaaatagccca
aatacctacaacgtaaATCCAACAGCAACAACTTTT
>PCR5_CF12_CONTIG_212_p12   186 pairs of NGS reads, 0.27%
CACGAAACTGTGAACTCAATAGCCTACTTGCCTGCTTTTCCATCCATTCTTTGACTGATAAAcca
tcttgggcctcaacataagcctgaccgccgaccatggctggcaaaagtccaatagcaaactttat
tttctctggccatgtcagcatCTCGTTGTTCCGCAAAATAGCCCAAATACCTACAACGTAAATCC
AACAGCAACAACCCCCC
>PCR5_CF12_CONTIG_212_p13   262 pairs of NGS reads, 0.39%
CACAGAAACTGTGAACTCAATAGCCTACTTGCCTGCTTTTCCATCCATTCTTTGACTGATAAACC
ATCTTgggcctcaacataagcctgaccgccgaccatggctggtaaaagtccaatagcaaacttta
ttttctctggccaTGTCAGCATCTCGTTGTTCCGCAAAATAGCCCAAATACCTACAACGTAAATC
CAACAGCAACAACTTTT
>PCR5_CF12_CONTIG_212_p14   172 pairs of NGS reads, 0.25%
CACAGAAACTGTGAACTCAATAGCCTACTTGCCTGCTTTTCCATCCATTCTTTGACTGATAAACC
ATCTTgggcctcaacataagcctgaccgccgaccatggctggcaaaagtccaatagcaaacttta
ttttctctggccatgtcagcaTCTCGTTGTTCCGCAAAATAGCCCAAATACCTACAACGTAAACC
CAACAGCAACAACCCCC
>PCR5_CF12_CONTIG_211_p15   150 pairs of NGS reads, 0.22%
CACAGAAACTGTGAACTCAATAGCCTACTTGCCTGCTTTTCCATCCATTCTTTGACTGATAAACC
ATCTtgggcctcaacataagcctgaccgccgaccatggctggcaaaagtccaatagcaaacttta
ttttctctggccATGTAGCATCTCGTTGTTCCGCAAAATAGCCCAAATACCTACAACGTAAATCC
AACAGCAACAACCCCC
>PCR5_CF12_CONTIG_212_p16   139 pairs of NGS reads, 0.2%
CCAGAAACTGTGAACTCAATAGCCTACTTGCCTGCTTTTCCATCCATTCTTTGACTGATAAAcca
tcttgggcctcaacataagcctgaccgccgaccatggctggcaaaagtccaatagcaaactttat
tttctctggccaTGTCAGCATCTCGTTGTTCCGCAAAATAGCCCAAATACCTACAACGTAAATCC
AACAGCAACAACCCCCC
>PCR5_CF12_CONTIG_212_p17   130 pairs of NGS reads, 0.19%
CACAGAAACTGTGAACTCAATAGCCTACTTGCCTGCTTTTCCATCCATTCTTTGACTGATAAACC
ATCTTgggcctcaacataagcctgaccgccgaccatggctggcaaaagtccaatagcaaacttta
ttttctctggccatgtcagcaTCTCGTTGTTCCGCAAAATAGCCCAAATACCTACAACGTAAAGC
CAACAGCAACAACTTTT
>PCR5_CF12_CONTIG_164_p18   118 pairs of NGS reads, 0.17%
CACAGAAACTGTGAACTCAATagcctacttgcctgcttttccatccattctttgactgataaacc
atcttgggcctcaacataagcctgaccgccgaccagcatctcgttgttccgcaaaatagcccaaa
tacctacaacgtaaatCCAACAGCAACAACAAAA
>PCR5_CF12_CONTIG_161_p19   117 pairs of NGS reads, 0.17%
CACAGAAACTGTGAACTCAatagcctacttgcctgcttttccatccattctttgactgataaacc

atcttgggcctcaacataagcctgaccgccgaccatctcgttgttccgcaaaatagcccaaatac
ctacaacgtaaatCCAACAGCAACAACCCCC
>PCR5_CF12_CONTIG_213_p20 114 pairs of NGS reads, 0.17%
CACAGAAACTGTGAACTCAATAGCCTACTTGCCTGCTTTTCCATCCATTCTTTGACTGATAAACC
ATCTTGggcctcaacataagcctgaccgccgaccaatggctggcaaaagtccaatagcaaacttt
atttctctggccATGTCAGCATCTCGTTGTTCCGCAAAATAGCCCAAATACCTACAACGTAAAT
CCAACAGCAACAACCCCC
>PCR5_CF12_CONTIG_165_p21 113 pairs of NGS reads, 0.17%
CACAGAAACTGTGAACTCAATAgcctacttgcctgcttttccatccattctttgactgataaacc
atcttgggcctcaacataagcctgaccgccgacccagcatctcgttgttccgcaaaatagcccaa
atacctacaacgtaaaTCCAACAGCAACAACAAAA
>PCR5_CF12_CONTIG_245_p22 112 pairs of NGS reads, 0.16%
AAAAGTTGTTGCTGTTGGATTTACGTGTTAACTTTTACACTTTATGGTCTCAAAACACTAAACAT
ATGGTTAATGAGGCTATATTTTGTGTAATCTTACttaatttagacattttctgttttctgtgagt
tgtttcttgaagTGAGTAGTGCTTTGTTTGTGGTCTATAGGAGAAAAGTAAAACATTCAGAAGTC
GATATGGTGAGCTTACGAAGACGTAGGCTATTGAGTTCACAGTTTCTGTG
>PCR5_CF12_CONTIG_209_p23 88 pairs of NGS reads, 0.13%
CACAGAAACTGTGAACTCAATAGCCTACTTGCCTGCTTTTCCATCCATTCTTTGACTGATAAACC
ATcttgggcctcaacataagcctgaccgccgaccatggctggcaaaagtccaatagcaaacttta
ttttctctggccatgtcatctCGTTGTTCCGCAAAATAGCCCAAATACCTACAACGTAAATCCAA
CAGCAACAACCCCC
>PCR5_CF12_CONTIG_165_p24 86 pairs of NGS reads, 0.12%
CACAGAAACTGTGAACTCAATAGcctacttgcctgcttttccatccattctttgactgataaacc
atcttgggcctcaacataagcctgaccgccgaccaagcatctcgttgttccgcaaaatagcccaa
atacctacaacgtAAATCCAACAGCAACAACCCCC
>PCR5_CF12_CONTIG_166_p25 81 pairs of NGS reads, 0.12%
CACAGAAACTGTGAACTCAATAGcctacttgcctgcttttccatccattctttgactgataaacc
atcttgggcctcaacataagcctgaccgccgaccacagcatctcgttgttccgcaaaatagccca
aatacctacaacgtaaATCCAACAGCAACAACAAAA
>PCR5_CF12_CONTIG_212_p26 81 pairs of NGS reads, 0.12%
CACAGAACTGTGAACTCAATAGCCTACTTGCCTGCTTTTCCATCCATTCTTTGACTGATAAAcca
tcttgggcctcaacataagcctgaccgccgaccatggctggcaaaagtccaatagcaaactttat
tttctctggccatgtcAGCATCTCGTTGTTCCGCAAAATAGCCCAAATACCTACAACGTAAATCC
AACAGCAACAACCCCCC
>PCR5_CF12_CONTIG_212_p27 80 pairs of NGS reads, 0.12%
CAAGAAACTGTGAACTCAATAGCCTACTTGCCTGCTTTTCCATCCATTCTTTGACTGATAAAcca
tcttgggcctcaacataagcctgaccgccgaccatggctggcaaaagtccaatagcaaactttat
tttctctggccatGTCAGCATCTCGTTGTTCCGCAAAATAGCCCAAATACCTACAACGTAAATCC
AACAGCAACAACCCCCC
>PCR5_CF12_CONTIG_212_p28 77 pairs of NGS reads, 0.11%
CACGAAACTGTGAACTCAATAGCCTACTTGCCTGCTTTTCCATCCATTCTTTGACTGATAAACCA
Tcttgggcctcaacataagcctgaccgccgaccatggctggcaaaagtccaatagcaaactttat
tttctctggccatgtcagcatCTCGTTGTTCCGCAAAATAGCCCAAATACCTACAACGTAAATCC
AACAGCAACAACTTTTT
>PCR5_CF12_CONTIG_211_p29 69 pairs of NGS reads, 0.1%
CACAGAAACTGTGAACTCAATAGCCTACTTGCCTGCTTTTCCATCCATTCTTTGACTGATAAACC
ATCTtgggcctcaacataagcctgaccgccgaccatggctggcaaaagtccaatagcaaacttta
ttttctctggccATGTAGCATCTCGTTGTTCCGCAAAATAGCCCAAATACCTACAACGTAAATCC
AACAGCAACAACTTTT

AtPDS DNA 3

>PCR6_CG1_CONTIG_213_p1   26248 pairs of NGS reads, 46.07%
CACAGAAACTGTGAACTCAATAGCCTACTTGCCTGCTTTTCCATCCATTCTTTGACTGATAaacc
atcttgggcctcaacataagcctgaccgccgaccatggctggcaaaagtccaatagcaaacttta
ttttctctggccatgtcagcATCTCGTTGTTCCGCAAAATAGCCCAAATACCTACAACGTAAATC
CAACAGCAACAACCCCCC
>PCR6_CG1_CONTIG_213_p2   14152 pairs of NGS reads, 24.84%
CACAGAAACTGTGAACTCAATAGCCTACTTGCCTGCTTTTCCATCCATTCTTTGACTGATAAACC
ATcttgggcctcaacataagcctgaccgccgaccatggctggcaaaagtccaatagcaaacttta
ttttctctggccatgtcagcATCTCGTTGTTCCGCAAAATAGCCCAAATACCTACAACGTAAATC
CAACAGCAACAACTTTTT
>PCR6_CG1_CONTIG_212_p3   7389 pairs of NGS reads, 12.97%
CACAGAAACTGTGAACTCAATAGCCTACTTGCCTGCTTTTCCATCCATTCTTTGACTGATAAACC
ATcttgggcctcaacataagcctgaccgccgaccatggctggcaaaagtccaatagcaaacttta
ttttctctggccatgtcagcATCTCGTTGTTCCGCAAAATAGCCCAAATACCTACAACGTAAATC
CAACAGCAACAACAAAA
>PCR6_CG1_CONTIG_166_p4   757 pairs of NGS reads, 1.32%
CACAGAAACTGTGAACTCAATAGcctacttgcctgcttttccatccattctttgactgataaacc
atcttgggcctcaacataagcctgaccgccgaccacagcatctcgttgttccgcaaaatagccca
aatacctacaacgtaaATCCAACAGCAACAACCCCC
>PCR6_CG1_CONTIG_164_p5   694 pairs of NGS reads, 1.21%
CACAGAAACTGTGAACTCAATagcctacttgcctgcttttccatccattctttgactgataaacc
atcttgggcctcaacataagcctgaccgccgaccagcatctcgttgttccgcaaaatagcccaaa
tacctacaacgtaaATCCAACAGCAACAACCCCC
>PCR6_CG1_CONTIG_165_p6   622 pairs of NGS reads, 1.09%
CACAGAAACTGTGAACTCAATAgcctacttgcctgcttttccatccattctttgactgataaacc
atcttgggcctcaacataagcctgaccgccgacccagcatctcgttgttccgcaaaatagcccaa
atacctacaacgtAAATCCAACAGCAACAACCCCC
>PCR6_CG1_CONTIG_245_p7   397 pairs of NGS reads, 0.69%
AAAAGTTGTTGCTGTTGGATTTACGTGTTAACTTTTACACTTTATGGTCTCAAAACACTAAACAT
ATGGTTAATGAGGCTATATTTTGTGTAATCTTACttaatttagacattttctgttttctgtgagt
tgtttcttgaagtGAGTAGTGCTTTGTTTGTGGTCTATAGGAGAAAAGTAAAACATTCAGAAGTC
GATATGGTGAGCTTACGAAGACGTAGGCTATTGAGTTCACAGTTTCTGTG
>PCR6_CG1_CONTIG_212_p8   862 pairs of NGS reads, 1.51%
CACAGAAACTGTGAACTCAATAGCCTACTTGCCTGCTTTTCCATCCATTCTTTGACTGATAAACC
ATCTTgggcctcaacataagcctgaccgccgaccatggctggtaaaagtccaatagcaaacttta
ttttctctggccaTGTCAGCATCTCGTTGTTCCGCAAAATAGCCCAAATACCTACAACGTAAATC
CAACAGCAACAACCCCCC
>PCR6_CG1_CONTIG_165_p9   390 pairs of NGS reads, 0.68%
CACAGAAACTGTGAACTCAATAGcctacttgcctgcttttccatccattctttgactgataaacc
atcttgggcctcaacataagcctgaccgccgacccagcatctcgttgttccgcaaaatagcccaa
atacctacaacgtAAATCCAACAGCAACAACTTTT
>PCR6_CG1_CONTIG_164_p10   377 pairs of NGS reads, 0.66%
CACAGAAACTGTGAACTCAATagcctacttgcctgcttttccatccattctttgactgataaacc
atcttgggcctcaacataagcctgaccgccgaccagcatctcgttgttccgcaaaatagcccaaa
tacctacaacgtaaATCCAACAGCAACAACTTTT
>PCR6_CG1_CONTIG_166_p11   322 pairs of NGS reads, 0.56%
CACAGAAACTGTGAACTCAATAGcctacttgcctgcttttccatccattctttgactgataaacc
atcttgggcctcaacataagcctgaccgccgaccacagcatctcgttgttccgcaaaatagccca
aatacctacaacgtaaATCCAACAGCAACAACTTTT
>PCR6_CG1_CONTIG_212_p12   285 pairs of NGS reads, 0.5%
CACAGAAACTGTGAACTCAATAGCCTACTTGCCTGCTTTTCCATCCATTCTTTGACTGATAAACC

ATCTTgggcctcaacataagcctgaccgccgaccatggctggcaaaagtccaatagcaaacttta
ttttctctggccatgtcagcATCTCGTTGTTCCGCAAAATAGCCCAAATACCTACAACGTAAAGC
CAACAGCAACAACCCCC
>PCR6_CG1_CONTIG_212_p13   271 pairs of NGS reads, 0.47%
CACAGAAACTGTGAACTCAATAGCCTACTTGCCTGCTTTTCCATCCATTCTTTGACTGATAAACC
ATCTTgggcctcaacataagcctgaccgccgaccatggctggtaaaagtccaatagcaaacttta
ttttctctggccaTGTCAGCATCTCGTTGTTCCGCAAAATAGCCCAAATACCTACAACGTAAATC
CAACAGCAACAACTTTT
>PCR6_CG1_CONTIG_211_p14   195 pairs of NGS reads, 0.34%
CACAGAAACTGTGAACTCAATAGCCTACTTGCCTGCTTTTCCATCCATTCTTTGACTGATAAACC
ATCTtgggcctcaacataagcctgaccgccgaccatggctggcaaaagtccaatagcaaacttta
ttttctctggccATGTAGCATCTCGTTGTTCCGCAAAATAGCCCAAATACCTACAACGTAAATCC
AACAGCAACAACCCCC
>PCR6_CG1_CONTIG_213_p15   178 pairs of NGS reads, 0.31%
CACAGAAACTGTGAACTCAATAGCCTACTTGCCTGCTTTTCCATCCATTCTTTGACTGATAAACC
ATCTTGggcctcaacataagcctgaccgccgaccaatggctggcaaaagtccaatagcaaacttt
atttctctggccATGTCAGCATCTCGTTGTTCCGCAAAATAGCCCAAATACCTACAACGTAAAT
CCAACAGCAACAACCCCC
>PCR6_CG1_CONTIG_245_p16   171 pairs of NGS reads, 0.3%
TTTTGTTGTTGCTGTTGGATTTACGTGTTAACTTTTACACTTTATGGTCTCAAAACACTAAACAT
ATGGTTAATGAGGCTATATTTTGTGTAATCTTACttaatttagacattttctgttttctgtgagt
tgtttcttgaagTGAGTAGTGCTTTGTTTGTGGTCTATAGGAGAAAAGTAAAACATTCAGAAGTC
GATATGGTGAGCTTACGAAGACGTAGGCTATTGAGTTCACAGTTTCTGTG
>PCR6_CG1_CONTIG_212_p17   164 pairs of NGS reads, 0.28%
CACAGAAACTGTGAACTCAATAGCCTACTTGCCTGCTTTTCCATCCATTCTTTGACTGATAAACC
ATCTTgggcctcaacataagcctgaccgccgaccatggctggcaaaagtccaatagcaaacttta
ttttctctggccatgtcagcATCTCGTTGTTCCGCAAAATAGCCCAAATACCTACAACGTAAAGC
CAACAGCAACAACTTTT
>PCR6_CG1_CONTIG_164_p18   153 pairs of NGS reads, 0.26%
CACAGAAACTGTGAACTCAATAgcctacttgcctgcttttccatccattctttgactgataaacc
atcttgggcctcaacataagcctgaccgccgaccagcatctcgttgttccgcaaaatagcccaaa
tacctacaacgtaaATCCAACAGCAACAACAAAA
>PCR6_CG1_CONTIG_212_p19   138 pairs of NGS reads, 0.24%
CACGAAACTGTGAACTCAATAGCCTACTTGCCTGCTTTTCCATCCATTCTTTGACTGATAaacca
tcttgggcctcaacataagcctgaccgccgaccatggctggcaaaagtccaatagcaaactttat
tttctctggccatgtcagcatCTCGTTGTTCCGCAAAATAGCCCAAATACCTACAACGTAAATCC
AACAGCAACAACCCCCC
>PCR6_CG1_CONTIG_166_p20   114 pairs of NGS reads, 0.2%
CACAGAAACTGTGAACTCAATAGCctacttgcctgcttttccatccattctttgactgataaacc
atcttgggcctcaacataagcctgaccgccgaccacagcatctcgttgttccgcaaaatagccca
aatacctacaacgtaaATCCAACAGCAACAACAAAA
>PCR6_CG1_CONTIG_165_p21   113 pairs of NGS reads, 0.19%
CACAGAAACTGTGAACTCAATAGcctacttgcctgcttttccatccattctttgactgataaacc
atcttgggcctcaacataagcctgaccgccgacccagcatctcgttgttccgcaaaatagcccaa
atacctacaacgtAAATCCAACAGCAACAACAAAA
>PCR6_CG1_CONTIG_209_p22   99 pairs of NGS reads, 0.17%
CACAGAAACTGTGAACTCAATAGCCTACTTGCCTGCTTTTCCATCCATTCTTTGACTGATAAACC
ATcttgggcctcaacataagcctgaccgccgaccatggctggcaaaagtccaatagcaaacttta
ttttctctggccatgtcatcTCGTTGTTCCGCAAAATAGCCCAAATACCTACAACGTAAATCCAA
CAGCAACAACCCCC
>PCR6_CG1_CONTIG_212_p23   87 pairs of NGS reads, 0.15%
CCAGAAACTGTGAACTCAATAGCCTACTTGCCTGCTTTTCCATCCATTCTTTGACTGATAaacca

tcttgggcctcaacataagcctgaccgccgaccatggctggcaaaagtccaatagcaaactttat
tttctctggccaTGTCAGCATCTCGTTGTTCCGCAAAATAGCCCAAATACCTACAACGTAAATCC
AACAGCAACAACCCCCC
>PCR6_CG1_CONTIG_211_p24   82 pairs of NGS reads, 0.14%
CACAGAAACTGTGAACTCAATAGCCTACTTGCCTGCTTTTCCATCCATTCTTTGACTGATAAACC
ATCTTgggcctcaacataagcctgaccgccgaccatggctggcaaaagtccaatagcaaacttta
ttttctctggccATGTAGCATCTCGTTGTTCCGCAAAATAGCCCAAATACCTACAACGTAAATCC
AACAGCAACAACTTTT
>PCR6_CG1_CONTIG_212_p25   74 pairs of NGS reads, 0.12%
CACGAAACTGTGAACTCAATAGCCTACTTGCCTGCTTTTCCATCCATTCTTTGACTGATAAACCA
Tcttgggcctcaacataagcctgaccgccgaccatggctggcaaaagtccaatagcaaactttat
tttctctggccatgtcagcatCTCGTTGTTCCGCAAAATAGCCCAAATACCTACAACGTAAATCC
AACAGCAACAACTTTTT
>PCR6_CG1_CONTIG_212_p26   72 pairs of NGS reads, 0.12%
CACAGAAACTGTGAACTCAATAGCCTACTTGCCTGCTTTTCCATCCATTCTTTGACTGATAAACC
ATCTTgggcctcaacataagcctgaccgccgaccatggctggcaaaagtccaatagcaaacttta
ttttctctggccatgtcagcATCTCGTTGTTCCGCAAAATAGCCCAAATACCTACAACGTAAACC
CAACAGCAACAACCCCC
>PCR6_CG1_CONTIG_165_p27   72 pairs of NGS reads, 0.12%
CACAGAAACTGTGAACTCAATAGcctacttgcctgcttttccatccattctttgactgataaacc
atcttgggcctcaacataagcctgaccgccgaccaagcatctcgttgttccgcaaaatagcccaa
atacctacaacgTAAATCCAACAGCAACAACCCCC
>PCR6_CG1_CONTIG_161_p28   69 pairs of NGS reads, 0.12%
CACAGAAACTGTGAACTCAatagcctacttgcctgcttttccatccattctttgactgataaacc
atcttgggcctcaacataagcctgaccgccgaccatctcgttgttccgcaaaatagcccaaatac
ctacaacgtaaaTCCAACAGCAACAACCCCC
>PCR6_CG1_CONTIG_165_p29   64 pairs of NGS reads, 0.11%
CACAGAAACTGTGAACTCAATAGcctacttgcctgcttttccatccattctttgactgataaacc
atcttgggcctcaacataagcctgaccgccgaccaagcatctcgttgttccgcaaaatagcccaa
atacctacaacgTAAATCCAACAGCAACAACTTTT
>PCR6_CG1_CONTIG_213_p30   58 pairs of NGS reads, 0.1%
CACAGAAACTGTGAACTCAATAGCCTACTTGCCTGCTTTTCCATCCATTCTTTGACTGATAAACC
ATCTTGggcctcaacataagcctgaccgccgaccaatggctggcaaaagtccaatagcaaacttt
attttctctggccATGTCAGCATCTCGTTGTTCCGCAAAATAGCCCAAATACCTACAACGTAAAT
CCAACAGCAACAACTTTT

AtPDS RNP 1

>CPCR1_NH5_CONTIG_213_p1   6552 pairs of NGS reads, 14.98%
CACAGAAACTGTGAACTCAATAGCCTACTTGCCTGCTTTTCCATCCATTCTTTGACTGATAAACC
Atcttgggcctcaacataagcctgaccgccgaccatggctggcaaaagtccaatagcaaacttta
ttttctctggccatgtCAGCATCTCGTTGTTCCGCAAAATAGCCCAAATACCTACAACGTAAATC
CAACAGCAACAACCCCCC
>CPCR1_NH5_CONTIG_165_p2   3912 pairs of NGS reads, 8.94%
CACAGAAACTGTGAACTCaatagcctacttgcctgcttttccatccattctttgactgataaacc
atcttgggcctcaacataagcctgaccgccgaccagcatctcgttgttccgcaaaatagcccaaa
tacctacaacgtaaatCCAACAGCAACAACCCCCC
>CPCR1_NH5_CONTIG_212_p3   3591 pairs of NGS reads, 8.21%
CACAGAAACTGTGAACTCAATAGCCTACTTGCCTGCTTTTCCATCCATTCTTTGACTGATAAACC
ATcttgggcctcaacataagcctgaccgccgaccatggctggcaaaagtccaatagcaaacttta
ttttctctggccatgtCAGCATCTCGTTGTTCCGCAAAATAGCCCAAATACCTACAACGTAAATC

CAACAGCAACAACTTTT
>CPCR1_NH5_CONTIG_166_p4   3271 pairs of NGS reads, 7.48%
CACAGAAACTGTGAACTCAAtagcctacttgcctgcttttccatccattctttgactgataaacc
atcttgggcctcaacataagcctgaccgccgaccacagcatctcgttgttccgcaaaatagccca
aatacctacaacgtaaATCCAACAGCAACAACCCCC
>CPCR1_NH5_CONTIG_165_p5   2906 pairs of NGS reads, 6.64%
CACAGAAACTGTGAACTCAatagcctacttgcctgcttttccatccattctttgactgataaacc
atcttgggcctcaacataagcctgaccgccgacccagcatctcgttgttccgcaaaatagcccaa
atacctacaacgtaaaTCCAACAGCAACAACCCCC
>CPCR1_NH5_CONTIG_212_p6   2734 pairs of NGS reads, 6.25%
CACAGAAACTGTGAACTCAATAGCCTACTTGCCTGCTTTTCCATCCATTCTTTGACTGATAAACC
ATcttgggcctcaacataagcctgaccgccgaccatggctggcaaaagtccaatagcaaacttta
ttttctctggccatgtCAGCATCTCGTTGTTCCGCAAAATAGCCCAAATACCTACAACGTAAATC
CAACAGCAACAACAAAA
>CPCR1_NH5_CONTIG_164_p7   2463 pairs of NGS reads, 5.63%
CACAGAAACTGTGAACTCAatagcctacttgcctgcttttccatccattctttgactgataaacc
atcttgggcctcaacataagcctgaccgccgaccagcatctcgttgttccgcaaaatagcccaaa
tacctacaacgtaaatCCAACAGCAACAACTTTT
>CPCR1_NH5_CONTIG_166_p8   2108 pairs of NGS reads, 4.82%
CACAGAAACTGTGAACTCAATagcctacttgcctgcttttccatccattctttgactgataaacc
atcttgggcctcaacataagcctgaccgccgaccacagcatctcgttgttccgcaaaatagccca
aatacctacaacgtaaATCCAACAGCAACAACTTTT
>CPCR1_NH5_CONTIG_165_p9   1950 pairs of NGS reads, 4.46%
CACAGAAACTGTGAACTCAAtagcctacttgcctgcttttccatccattctttgactgataaacc
atcttgggcctcaacataagcctgaccgccgacccagcatctcgttgttccgcaaaatagcccaa
atacctacaacgtaaaTCCAACAGCAACAACTTTT
>CPCR1_NH5_CONTIG_164_p10   1637 pairs of NGS reads, 3.74%
CACAGAAACTGTGAACTCAATagcctacttgcctgcttttccatccattctttgactgataaacc
atcttgggcctcaacataagcctgaccgccgaccagcatctcgttgttccgcaaaatagcccaaa
tacctacaacgtaaatCCAACAGCAACAACAAAA
>CPCR1_NH5_CONTIG_166_p11   1367 pairs of NGS reads, 3.12%
CACAGAAACTGTGAACTCAATAGcctacttgcctgcttttccatccattctttgactgataaacc
atcttgggcctcaacataagcctgaccgccgaccacagcatctcgttgttccgcaaaatagccca
aatacctacaacgtaaATCCAACAGCAACAACAAAA
>CPCR1_NH5_CONTIG_165_p12   1199 pairs of NGS reads, 2.74%
CACAGAAACTGTGAACTCAATAgcctacttgcctgcttttccatccattctttgactgataaacc
atcttgggcctcaacataagcctgaccgccgacccagcatctcgttgttccgcaaaatagcccaa
atacctacaacgtaaaTCCAACAGCAACAACAAAA
>CPCR1_NH5_CONTIG_211_p13   942 pairs of NGS reads, 2.15%
CACAGAAACTGTGAACTCAATAGCCTACTTGCCTGCTTTTCCATCCATTCTTTGACTGATAAACC
ATCttgggcctcaacataagcctgaccgccgaccatggctggcaaaagtccaatagcaaacttta
ttttctctggccatgtAGCATCTCGTTGTTCCGCAAAATAGCCCAAATACCTACAACGTAAATCC
AACAGCAACAACCCCC
>CPCR1_NH5_CONTIG_211_p14   618 pairs of NGS reads, 1.41%
CACAGAAACTGTGAACTCAATAGCCTACTTGCCTGCTTTTCCATCCATTCTTTGACTGATAAACC
ATcttgggcctcaacataagcctgaccgccgaccatggctggcaaaagtccaatagcaaacttta
ttttctctggccatgtAGCATCTCGTTGTTCCGCAAAATAGCCCAAATACCTACAACGTAAATCC
AACAGCAACAACTTTT
>CPCR1_NH5_CONTIG_211_p15   442 pairs of NGS reads, 1.01%
CACAGAAACTGTGAACTCAATAGCCTACTTGCCTGCTTTTCCATCCATTCTTTGACTGATAAACC
ATCTtgggcctcaacataagcctgaccgccgaccatggctggcaaaagtccaatagcaaacttta
ttttctctggccatgtAGCATCTCGTTGTTCCGCAAAATAGCCCAAATACCTACAACGTAAATCC

AACAGCAACAACAAAA
>CPCR1_NH5_CONTIG_165_p16   333 pairs of NGS reads, 0.76%
CACAGAAACTGTGAACTCAATAGcctacttgcctgcttttccatccattctttgactgataaacc
atcttgggcctcaacataagcctgaccgccgaccaagcatctcgttgttccgcaaaatagcccaa
atacctacaacgtAAATCCAACAGCAACAACCCCC
>CPCR1_NH5_CONTIG_161_p17   283 pairs of NGS reads, 0.64%
CACAGAAACTGTGAACTCAatagcctacttgcctgcttttccatccattctttgactgataaacc
atcttgggcctcaacataagcctgaccgccgaccatctcgttgttccgcaaaatagcccaaatac
ctacaacgtaaatCCAACAGCAACAACCCCC
>CPCR1_NH5_CONTIG_212_p18   265 pairs of NGS reads, 0.6%
CACAGAAACTGTGAACTCAATAGCCTACTTGCCTGCTTTTCCATCCATTCTTTGACTGATAAACC
ATCTTgggcctcaacataagcctgaccgccgaccaatggctggcaaaagtccaatagcaaacttt
atttctctggccATGTAGCATCTCGTTGTTCCGCAAAATAGCCCAAATACCTACAACGTAAATC
CAACAGCAACAACCCCC
>CPCR1_NH5_CONTIG_165_p19   216 pairs of NGS reads, 0.49%
CACAGAAACTGTGAACTCAATAgcctacttgcctgcttttccatccattctttgactgataaacc
atcttgggcctcaacataagcctgaccgccgaccaagcatctcgttgttccgcaaaatagcccaa
atacctacaacgtAAATCCAACAGCAACAACTTTT
>CPCR1_NH5_CONTIG_161_p20   214 pairs of NGS reads, 0.48%
CACAGAAACTGTGAACTCaatagcctacttgcctgcttttccatccattctttgactgataaacc
atcttgggcctcaacataagcctgaccgccgaccatctcgttgttccgcaaaatagcccaaatac
ctacaacgtaaatCCAACAGCAACAACTTTT
>CPCR1_NH5_CONTIG_212_p21   183 pairs of NGS reads, 0.41%
CACAGAAACTGTGAACTCAATAGCCTACTTGCCTGCTTTTCCATCCATTCTTTGACTGATAAACC
ATCTTgggcctcaacataagcctgaccgccgaccaatggctggcaaaagtccaatagcaaacttt
atttctctggccATGTAGCATCTCGTTGTTCCGCAAAATAGCCCAAATACCTACAACGTAAATC
CAACAGCAACAACTTTT
>CPCR1_NH5_CONTIG_213_p22   181 pairs of NGS reads, 0.41%
CACAGAAACTGTGAACTCAATAGCCTACTTGCCTGCTTTTCCATCCATTCTTTGACTGATAAACC
ATCTTGggcctcaacataagcctgaccgccgaccatggctggcaaaagtccaatagcaaacttta
ttttctctggccatgtACAGCATCTCGTTGTTCCGCAAAATAGCCCAAATACCTACAACGTAAAT
CCAACAGCAACAACCCCC
>CPCR1_NH5_CONTIG_213_p23   152 pairs of NGS reads, 0.34%
CACAGAAACTGTGAACTCAATAGCCTACTTGCCTGCTTTTCCATCCATTCTTTGACTGATAAACC
ATCTTGggcctcaacataagcctgaccgccgaccaatggctggcaaaagtccaatagcaaacttt
atttctctggccATGTCAGCATCTCGTTGTTCCGCAAAATAGCCCAAATACCTACAACGTAAAT
CCAACAGCAACAACCCCC
>CPCR1_NH5_CONTIG_213_p24   141 pairs of NGS reads, 0.32%
CACAGAAACTGTGAACTCAATAGCCTACTTGCCTGCTTTTCCATCCATTCTTTGACTGATAAACC
ATCTTGggcctcaacataagcctgaccgccgaccatggctggcaaaagtccaatagcaaacttta
ttttctctggccatgtTCAGCATCTCGTTGTTCCGCAAAATAGCCCAAATACCTACAACGTAAAT
CCAACAGCAACAACCCCC
>CPCR1_NH5_CONTIG_212_p25   140 pairs of NGS reads, 0.32%
CACAGAAACTGTGAACTCAATAGCCTACTTGCCTGCTTTTCCATCCATTCTTTGACTGATAAACC
ATCTTgggcctcaacataagcctgaccgccgaccaatggctggcaaaagtccaatagcaaacttt
atttctctggccATGTAGCATCTCGTTGTTCCGCAAAATAGCCCAAATACCTACAACGTAAATC
CAACAGCAACAACAAAA
>CPCR1_NH5_CONTIG_213_p26   138 pairs of NGS reads, 0.31%
CACAGAAACTGTGAACTCAATAGCCTACTTGCCTGCTTTTCCATCCATTCTTTGACTGATAAACC
ATCTTgggcctcaacataagcctgaccgccgaccatggctggcaaaagtccaatagcaaacttta
ttttctctggccatgtACAGCATCTCGTTGTTCCGCAAAATAGCCCAAATACCTACAACGTAAAT
CCAACAGCAACAACTTTT

>CPCR1_NH5_CONTIG_211_p27   136 pairs of NGS reads, 0.31%
CACAGAAACTGTGAACTCAATAGCCTACTTGCCTGCTTTTCCATCCATTCTTTGACTGATAAACC
ATCTTgggcctcaacataagcctgaccgccgaccatggctggcaaaagtccaatagcaaacttta
ttttctctggccatgcAGCATCTCGTTGTTCCGCAAAATAGCCCAAATACCTACAACGTAAATCC
AACAGCAACAACCCCC
>CPCR1_NH5_CONTIG_209_p28   126 pairs of NGS reads, 0.28%
CACAGAAACTGTGAACTCAATAGCCTACTTGCCTGCTTTTCCATCCATTCTTTGACTGATAAACC
ATcttgggcctcaacataagcctgaccgccgaccatggctggcaaaagtccaatagcaaacttta
ttttctctggccatgtCATCTCGTTGTTCCGCAAAATAGCCCAAATACCTACAACGTAAATCCAA
CAGCAACAACCCCC
>CPCR1_NH5_CONTIG_213_p29   111 pairs of NGS reads, 0.25%
CACAGAAACTGTGAACTCAATAGCCTACTTGCCTGCTTTTCCATCCATTCTTTGACTGATAAACC
ATCTTgggcctcaacataagcctgaccgccgaccatggctggcaaaagtccaatagcaaacttta
ttttctctggccatgtTCAGCATCTCGTTGTTCCGCAAAATAGCCCAAATACCTACAACGTAAAT
CCAACAGCAACAACTTTT
>CPCR1_NH5_CONTIG_161_p30   110 pairs of NGS reads, 0.25%
CACAGAAACTGTGAACTCAatagcctacttgcctgcttttccatccattctttgactgataaacc
atcttgggcctcaacataagcctgaccgccgaccatctcgttgttccgcaaaatagcccaaatac
ctacaacgtaaatCCAACAGCAACAACAAAA
>CPCR1_NH5_CONTIG_209_p31   109 pairs of NGS reads, 0.24%
CACAGAAACTGTGAACTCAATAGCCTACTTGCCTGCTTTTCCATCCATTCTTTGACTGATAAACC
Atcttgggcctcaacataagcctgaccgccgaccatggctggcaaaagtccaatagcaaacttta
ttttctctggccatgtCATCTCGTTGTTCCGCAAAATAGCCCAAATACCTACAACGTAAATCCAA
CAGCAACAACTTTT
>CPCR1_NH5_CONTIG_165_p32   104 pairs of NGS reads, 0.23%
CACAGAAACTGTGAACTCAATAGcctacttgcctgcttttccatccattctttgactgataaacc
atcttgggcctcaacataagcctgaccgccgaccaagcatctcgttgttccgcaaaatagcccaa
atacctacaacgtAAATCCAACAGCAACAACAAAA
>CPCR1_NH5_CONTIG_208_p33   92 pairs of NGS reads, 0.21%
CACAGAAACTGTGAACTCAATAGCCTACTTGCCTGCTTTTCCATCCATTCTTTGACTGATAAACC
Atcttgggcctcaacataagcctgaccgccgaccatggctggcaaaagtccaatagcaaacttta
ttttctctggccatgtATCTCGTTGTTCCGCAAAATAGCCCAAATACCTACAACGTAAATCCAAC
AGCAACAACCCCC
>CPCR1_NH5_CONTIG_163_p34   92 pairs of NGS reads, 0.21%
CACAGAAACTGTGAACTCAATagcctacttgcctgcttttccatccattctttgactgataaacc
atcttgggcctcaacataagcctgaccgccgaccgcatctcgttgttccgcaaaatagcccaaat
acctacaacgtaAATCCAACAGCAACAACCCCC
>CPCR1_NH5_CONTIG_213_p35   83 pairs of NGS reads, 0.18%
CACAGAAACTGTGAACTCAATAGCCTACTTGCCTGCTTTTCCATCCATTCTTTGACTGATAAACC
ATCTTGggcctcaacataagcctgaccgccgaccatggctggcaaaagtccaatagcaaacttta
ttttctctggccatgtTCAGCATCTCGTTGTTCCGCAAAATAGCCCAAATACCTACAACGTAAAT
CCAACAGCAACAACAAAA
>CPCR1_NH5_CONTIG_213_p36   82 pairs of NGS reads, 0.18%
CACAGAAACTGTGAACTCAATAGCCTACTTGCCTGCTTTTCCATCCATTCTTTGACTGATAAACC
ATCTTGggcctcaacataagcctgaccgccgaccatggctggcaaaagtccaatagcaaacttta
ttttctctggccatgtACAGCATCTCGTTGTTCCGCAAAATAGCCCAAATACCTACAACGTAAAT
CCAACAGCAACAACAAAA
>CPCR1_NH5_CONTIG_214_p37   75 pairs of NGS reads, 0.17%
CACAGAAACTGTGAACTCAATAGCCTACTTGCCTGCTTTTCCATCCATTCTTTGACTGATAAACC
ATCTTGGgcctcaacataagcctgaccgccgaccaatggctggcaaaagtccaatagcaaacttt
attttctctggccATGTACAGCATCTCGTTGTTCCGCAAAATAGCCCAAATACCTACAACGTAAA
TCCAACAGCAACAACCCCC

>CPCR1_NH5_CONTIG_163_p38   74 pairs of NGS reads, 0.16%
CACAGAAACTGTGAACTCAATagcctacttgcctgcttttccatccattctttgactgataaacc
atcttgggcctcaacataagcctgaccgccgacagcatctcgttgttccgcaaaatagcccaaat
acctacaacgtaAATCCAACAGCAACAACCCCC
>CPCR1_NH5_CONTIG_213_p39   72 pairs of NGS reads, 0.16%
CACAGAAACTGTGAACTCAATAGCCTACTTGCCTGCTTTTCCATCCATTCTTTGACTGATAAACC
ATCTTgggcctcaacataagcctgaccgccgaccaatggctggcaaaagtccaatagcaaacttt
atttctctggccATGTCAGCATCTCGTTGTTCCGCAAAATAGCCCAAATACCTACAACGTAAAT
CCAACAGCAACAACTTTT
>CPCR1_NH5_CONTIG_211_p40   71 pairs of NGS reads, 0.16%
CACAGAAACTGTGAACTCAATAGCCTACTTGCCTGCTTTTCCATCCATTCTTTGACTGATAAACC
ATCTtgggcctcaacataagcctgaccgccgaccatggctggcaaaagtccaatagcaaacttta
ttttctctggccatgcAGCATCTCGTTGTTCCGCAAAATAGCCCAAATACCTACAACGTAAATCC
AACAGCAACAACAAAA
>CPCR1_NH5_CONTIG_167_p41   71 pairs of NGS reads, 0.16%
CACAGAAACTGTGAACTCAATAGCCtacttgcctgcttttccatccattctttgactgataaacc
atcttgggcctcaacataagcctgaccgccgaccatcagcatctcgttgttccgcaaaatagccc
aaatacctacaaCGTAAATCCAACAGCAACAACCCCC
>CPCR1_NH5_CONTIG_166_p42   70 pairs of NGS reads, 0.16%
CACAGAAACTGTGAACTCAATAGCctacttgcctgcttttccatccattctttgactgataaacc
atcttgggcctcaacataagcctgaccgccgaccatagcatctcgttgttccgcaaaatagccca
aatacctacaacGTAAATCCAACAGCAACAACTTTT
>CPCR1_NH5_CONTIG_52_p43   70 pairs of NGS reads, 0.16%
aaaagttgttgctgttggatttacgtaggctattgagttcacagtttctgtg
>CPCR1_NH5_CONTIG_212_p44   69 pairs of NGS reads, 0.15%
CACAGAAACTGTGAACTCAATAGCCTACTTGCCTGCTTTTCCATCCATTCTTTGACTGATAAACC
ATCTTgggcctcaacataagcctgaccgccgaccaatggctggcaaaagtccaatagcaaacttt
atttctctggccATGCAGCATCTCGTTGTTCCGCAAAATAGCCCAAATACCTACAACGTAAATC
CAACAGCAACAACCCCC
>CPCR1_NH5_CONTIG_210_p45   69 pairs of NGS reads, 0.15%
CACAGAAACTGTGAACTCAATAGCCTACTTGCCTGCTTTTCCATCCATTCTTTGACTGATAAACC
ATCttgggcctcaacataagcctgaccgccgaccatggctggcaaaagtccaatagcaaacttta
ttttctctggccatgtGCATCTCGTTGTTCCGCAAAATAGCCCAAATACCTACAACGTAAATCCA
ACAGCAACAACCCCC
>CPCR1_NH5_CONTIG_210_p46   67 pairs of NGS reads, 0.15%
CACAGAAACTGTGAACTCAATAGCCTACTTGCCTGCTTTTCCATCCATTCTTTGACTGATAAACC
ATCttgggcctcaacataagcctgaccgccgaccaatggctggcaaaagtccaatagcaaacttt
atttctctggccATGTCATCTCGTTGTTCCGCAAAATAGCCCAAATACCTACAACGTAAATCCA
ACAGCAACAACCCCC
>CPCR1_NH5_CONTIG_163_p47   67 pairs of NGS reads, 0.15%
CACAGAAACTGTGAACTCAAtagcctacttgcctgcttttccatccattctttgactgataaacc
atcttgggcctcaacataagcctgaccgccgaccgcatctcgttgttccgcaaaatagcccaaat
acctacaacgtaAATCCAACAGCAACAACTTTT
>CPCR1_NH5_CONTIG_245_p48   63 pairs of NGS reads, 0.14%
AAAAGTTGTTGCTGTTGGATTTACGTGTTAACTTTTACACTTTATGGTCTCAAAACACTAAACAT
ATGGTTAATGAGGCTATATTTTGTGTAATCTTACTTAAtttagacattttctgttttctgtgagt
tgtttcttgaagTGAGTAGTGCTTTGTTTGTGGTCTATAGGAGAAAAGTAAAACATTCAGAAGTC
GATATGGTGAGCTTACGAAGACGTAGGCTATTGAGTTCACAGTTTCTGTG
>CPCR1_NH5_CONTIG_208_p49   63 pairs of NGS reads, 0.14%
CACAGAAACTGTGAACTCAATAGCCTACTTGCCTGCTTTTCCATCCATTCTTTGACTGATAAACC
Atcttgggcctcaacataagcctgaccgccgaccatggctggcaaaagtccaatagcaaacttta
ttttctctggccatgtATCTCGTTGTTCCGCAAAATAGCCCAAATACCTACAACGTAAATCCAAC

AGCAACAACTTTT
>CPCR1_NH5_CONTIG_209_p50   63 pairs of NGS reads, 0.14%
CACAGAAACTGTGAACTCAATAGCCTACTTGCCTGCTTTTCCATCCATTCTTTGACTGATAAACC
ATcttgggcctcaacataagcctgaccgccgaccatggctggcaaaagtccaatagcaaacttta
ttttctctggccatgtCATCTCGTTGTTCCGCAAAATAGCCCAAATACCTACAACGTAAATCCAA
CAGCAACAACAAAA
>CPCR1_NH5_CONTIG_206_p51   60 pairs of NGS reads, 0.13%
CACAGAAACTGTGAACTCAATAGCCTACTTGCCTGCTTTTCCATCCATTCTTTGACTGATAAACc
atcttgggcctcaacataagcctgaccgccgaccatggctggcaaaagtccaatagcaaacttta
ttttctctggccatgtCTCGTTGTTCCGCAAAATAGCCCAAATACCTACAACGTAAATCCAACAG
CAACAACCCCC
>CPCR1_NH5_CONTIG_207_p52   59 pairs of NGS reads, 0.13%
CACAGAAACTGTGAACTCAATAGCCTACTTGCCTGCTTTTCCATCCATTCTTTGACTGATAAACc
atcttgggcctcaacataagcctgaccgccgaccatggctggcaaaagtccaatagcaaacttta
ttttctctggccatgtTCTCGTTGTTCCGCAAAATAGCCCAAATACCTACAACGTAAATCCAACA
GCAACAACTTTT
>CPCR1_NH5_CONTIG_127_p53   57 pairs of NGS reads, 0.13%
cacagaaactgtgaactcaatagcctacttgcctgcttttccatccattctttgactgataaacc
atctcgttgttccgcaaaatagcccaaatacctacaacgtaaatccaacagcaacaaccccc
>CPCR1_NH5_CONTIG_213_p54   57 pairs of NGS reads, 0.13%
CACAGAAACTGTGAACTCAATAGCCTACTTGCCTGCTTTTCCATCCATTCTTTGACTGATAAACC
ATCTTGggcctcaacataagcctgaccgccgaccaatggctggcaaaagtccaatagcaaacttt
atttctctggccATGTCAGCATCTCGTTGTTCCGCAAAATAGCCCAAATACCTACAACGTAAAT
CCAACAGCAACAACAAAA
>CPCR1_NH5_CONTIG_158_p55   54 pairs of NGS reads, 0.12%
CACAGAAACTGTGAACtcaatagcctacttgcctgcttttccatccattctttgactgataaacc
atcttgggcctcaacataagcctgaccgccgacctcgttgttccgcaaaatagcccaaatacta
caacgtaaatccAACAGCAACAACCCCC
>CPCR1_NH5_CONTIG_214_p56   52 pairs of NGS reads, 0.11%
CACAGAAACTGTGAACTCAATAGCCTACTTGCCTGCTTTTCCATCCATTCTTTGACTGATAAACC
ATCTTGGgcctcaacataagcctgaccgccgaccaatggctggcaaaagtccaatagcaaacttt
atttctctggccATGTTCAGCATCTCGTTGTTCCGCAAAATAGCCCAAATACCTACAACGTAAA
TCCAACAGCAACAACCCCC
>CPCR1_NH5_CONTIG_212_p57   51 pairs of NGS reads, 0.11%
CACAGAAACTGTGAACTCAATAGCCTACTTGCCTGCTTTTCCATCCATTCTTTGACTGATAAACC
ATCTTgggcctcaacataagcctgaccgccgaccacatggccagagaaaataaagtttgctattg
gacttttgccagCCATCAGCATCTCGTTGTTCCGCAAAATAGCCCAAATACCTACAACGTAAATC
CAACAGCAACAACCCCC
>CPCR1_NH5_CONTIG_55_p58   51 pairs of NGS reads, 0.11%
aaaagttgttgctgttggatttacgttgtaggctattgagttcacagtttctgtg
>CPCR1_NH5_CONTIG_208_p59   51 pairs of NGS reads, 0.11%
CACAGAAACTGTGAACTCAATAGCCTACTTGCCTGCTTTTCCATCCATTCTTTGACTGATAAACC
Atcttgggcctcaacataagcctgaccgccgaccatggctggcaaaagtccaatagcaaacttta
ttttctctggccatgtATCTCGTTGTTCCGCAAAATAGCCCAAATACCTACAACGTAAATCCAAC
AGCAACAACAAAA
>CPCR1_NH5_CONTIG_214_p60   48 pairs of NGS reads, 0.1%
CACAGAAACTGTGAACTCAATAGCCTACTTGCCTGCTTTTCCATCCATTCTTTGACTGATAAACC
ATCTTGGgcctcaacataagcctgaccgccgaccaatggctggcaaaagtccaatagcaaacttt
atttctctggccATGTACAGCATCTCGTTGTTCCGCAAAATAGCCCAAATACCTACAACGTAAA
TCCAACAGCAACAACTTTT
>CPCR1_NH5_CONTIG_163_p61   44 pairs of NGS reads, 0.1%
CACAGAAACTGTGAACTCAATagcctacttgcctgcttttccatccattctttgactgataaacc

atcttgggcctcaacataagcctgaccgccgacagcatctcgttgttccgcaaaatagcccaaat
acctacaacgtaAATCCAACAGCAACAACTTTT

AtPDS RNP 2

>CPCR2_NH6_CONTIG_242_p1   18053 pairs of NGS reads, 36.65%
ACGAGAGATAGATGATAGTGAAGGAACCTCTATCTGCGAGAAGAGTCCGTTGAAACCTTTGGACT
TGAGATAGTAATCAAGGGCGTCGTTAGTcgcaccgagagcggcggaggaaccacttccaccaacg
ccggctgtagcaccagcaCCGGAGGCGACATCGGAGCAACAATTCCCCATAATTTTTGATTTCCC
AACAAAAAAAAACTGCGGATTCAAAAGTACCTGGATCGAACAACGCAT
>CPCR2_NH6_CONTIG_242_p2   13118 pairs of NGS reads, 26.63%
ACGAGAGATAGATGATAGTGAAGGAACCTCTATCTGCGAGAAGAGTCCGTTGAAACCTTTGGACT
TGAGATAGTAATCAAGGGCGTCGTTAGTCGCAccgagagcggcggaggaaccacttccaccaacg
ccggctgtagcaccagcaCCGGAGGCGACATCGGAGCAACAATTCCCCATAATTTTTGATTTCCC
AACAAAAAAAAACTGCGGATTCAAAAGTACCTGGATCGAACAACAGCT
>CPCR2_NH6_CONTIG_242_p3   12387 pairs of NGS reads, 25.14%
ACGAGAGATAGATGATAGTGAAGGAACCTCTATCTGCGAGAAGAGTCCGTTGAAACCTTTGGACT
TGAGATAGTAATCAAGGGCGTCGTTAGTCGCAccgagagcggcggaggaaccacttccaccaacg
ccggctgtagcaccagcaCCGGAGGCGACATCGGAGCAACAATTCCCCATAATTTTTGATTTCCC
AACAAAAAAAAACTGCGGATTCAAAAGTACCTGGATCGAACAACCGAT
>CPCR2_NH6_CONTIG_166_p4   1643 pairs of NGS reads, 3.33%
ACGAGAGATAGATGATAGTGAAGgaacaagatgttataccttctcaagctcctcaagaacagact
tggcaacttcaccctttttgttcaaaaagagcatcaagcgtcatcattgggaccaaagccctaacc
actatgattcaaaAGTACCTGGATCGAACAACGCAT
>CPCR2_NH6_CONTIG_166_p5   974 pairs of NGS reads, 1.97%
ACGAGAGATAGATGATAGTGAAGGaacaagatgttataccttctcaagctcctcaagaacagact
tggcaacttcaccctttttgttcaaaaagagcatcaagcgtcatcattgggaccaaagccctaacc
actatgattcaaaAGTACCTGGATCGAACAACCGAT
>CPCR2_NH6_CONTIG_166_p6   796 pairs of NGS reads, 1.61%
ACGAGAGATAGATGATAGTGAAggaacaagatgttataccttctcaagctcctcaagaacagact
tggcaacttcaccctttttgttcaaaaagagcatcaagcgtcatcattgggaccaaagccctaacc
actatgattcaaaAGTACCTGGATCGAACAACAGCT
>CPCR2_NH6_CONTIG_213_p7   262 pairs of NGS reads, 0.53%
ACGAGAGATAGATGATAGTGAAGGAACCTCTATCTGCGAGAAGAGTCCGTTGAAACCTTTGGACT
TGAGATagtaatcaagggcgtcgttagtcgcaccgaccggctgtagcaccagcaccggaggcgac
atcggagcaacaATTCCCCATAATTTTTGATTTCCCAACAAAAAAAAACTGCGGATTCAAAAGTAC
CTGGATCGAACAACGCAT
>CPCR2_NH6_CONTIG_213_p8   164 pairs of NGS reads, 0.33%
ACGAGAGATAGATGATAGTGAAGGAACCTCTATCTGCGAGAAGAGTCCGTTGAAACCTTTGGACT
TGAGATagtaatcaagggcgtcgttagtcgcaccgaccggctgtagcaccagcaccggaggcgac
atcggagcaacaATTCCCCATAATTTTTGATTTCCCAACAAAAAAAAACTGCGGATTCAAAAGTAC
CTGGATCGAACAACAGCT
>CPCR2_NH6_CONTIG_243_p9   132 pairs of NGS reads, 0.26%
ACGAGAGATAGATGATAGTGAAGGAACCTCTATCTGCGAGAAGAGTCCGTTGAAACCTTTGGACT
TGAGATAGTAATCAAGGGCGTCGTTAGTCGCACCGAgagcggcggaggaaccacttccaccaacg
ccggctgtagcaccagcaCCGGAGGCGACATCGGAGCAACAATTCCCCATAATTTTTGATTTCCC
AACAAAAAAAAAACTGCGGATTCAAAAGTACCTGGATCGAACAACGCAT
>CPCR2_NH6_CONTIG_213_p10   115 pairs of NGS reads, 0.23%
ACGAGAGATAGATGATAGTGAAGGAACCTCTATCTGCGAGAAGAGTCCGTTGAAACCTTTGGACT
TGAGATagtaatcaagggcgtcgttagtcgcaccgaccggctgtagcaccagcaccggaggcgac
atcggagcaacaATTCCCCATAATTTTTGATTTCCCAACAAAAAAAAACTGCGGATTCAAAAGTAC
CTGGATCGAACAACCGAT

>CPCR2_NH6_CONTIG_243_p11   111 pairs of NGS reads, 0.22%
ACGAGAGATAGATGATAGTGAAGGAACCTCTATCTGCGAGAAGAGTCCGTTGAAACCTTTGGACT
TGAGATAGTAATCAAGGGCGTCGTTAGTCGCACCGAgagcggcggaggaaccacttccaccaacg
ccggctgtagcaccagcaCCGGAGGCGACATCGGAGCAACAATTCCCCATAATTTTTGATTTCCC
AACAAAAAAAAACTGCGGATTCAAAAGTACCTGGATCGAACAACAGCT
>CPCR2_NH6_CONTIG_243_p12   84 pairs of NGS reads, 0.17%
ACGAGAGATAGATGATAGTGAAGGAACCTCTATCTGCGAGAAGAGTCCGTTGAAACCTTTGGACT
TGAGATAGTAATCAAGGGCGTCGTTAGTCGCACCGAgagcggcggaggaaccacttccaccaacg
ccggctgtagcaccagcaCCGGAGGCGACATCGGAGCAACAATTCCCCATAATTTTTGATTTCCC
AACAAAAAAAAACTGCGGATTCAAAAGTACCTGGATCGAACAACCGAT
>CPCR2_NH6_CONTIG_243_p13   70 pairs of NGS reads, 0.14%
ACGAGAGATAGATGATAGTGAAGGAACCTCTATCTGCGAGAAGAGTCCGTTGAAACCTTTGGACT
TGAGATAGTAATCAAGGGCGTCGTTAGTCGCACCGAgagcggcggaggaaccacttccaccaacg
accggctgtagcACCAGCACCGGAGGCGACATCGGAGCAACAATTCCCCATAATTTTTGATTTCC
CAACAAAAAAAACTGCGGATTCAAAAGTACCTGGATCGAACAACGCAT
>CPCR2_NH6_CONTIG_241_p14   59 pairs of NGS reads, 0.11%
ACGAGAGATAGATGATAGTGAAGGAACCTCTATCTGCGAGAAGAGTCCGTTGAAACCTTTGGACT
TGAGATAGTAATCAAGGGCGTCGTTAGTCGCACCgagagcggcggaggaaccacttccaccaacg
ccggctgtagcaccagcaCCGGAGGCGACATCGGAGCAACAATTCCCCATAATTTTTGATTTCCC
AACAAAAAAAACTGCGGATTCAAAGTACCTGGATCGAACAACGCAT
>CPCR2_NH6_CONTIG_241_p15   54 pairs of NGS reads, 0.1%
ACGAGAGATAGATGATAGTGAAGGAACCTCTATCTGCGAGAAGAGTCCGTTGAAACCTTTGGACT
TGAGATAGTAATCAAGGGCGTCGTTAGTCGCACCgagagcggcggaggaaccacttccaccaacg
ccggctgtagcaccagcaCCGGAGGCGACATCGGAGCAACAATTCCCCATAATTTTTGATTTCCC
AACAAAAAAACTGCGGATTCAAAAGTACCTGGATCGAACAACGCAT

AtPDS RNP 3
>CPCR3_CD12_CONTIG_213_p1   10356 pairs of NGS reads, 16.05%
CACAGAAACTGTGAACTCAATAGCCTACTTGCCTGCTTTTCCATCCATTCTTTGACTGATAAAcc
atcttgggcctcaacataagcctgaccgccgaccatggctggcaaaagtccaatagcaaacttta
ttttctctggccatgtcagCATCTCGTTGTTCCGCAAAATAGCCCAAATACCTACAACGTAAATC
CAACAGCAACAACCCCCC
>CPCR3_CD12_CONTIG_212_p2   7369 pairs of NGS reads, 11.42%
CACAGAAACTGTGAACTCAATAGCCTACTTGCCTGCTTTTCCATCCATTCTTTGACTGATAAACC
ATcttgggcctcaacataagcctgaccgccgaccatggctggcaaaagtccaatagcaaacttta
ttttctctggccatgtcagCATCTCGTTGTTCCGCAAAATAGCCCAAATACCTACAACGTAAATC
CAACAGCAACAACTTTT
>CPCR3_CD12_CONTIG_165_p3   6748 pairs of NGS reads, 10.45%
CACAGAAACTGTGAACTCaatagcctacttgcctgcttttccatccattctttgactgataaacc
atcttgggcctcaacataagcctgaccgccgaccagcatctcgttgttccgcaaaatagcccaaa
tacctacaacgtaaatCCAACAGCAACAACCCCCC
>CPCR3_CD12_CONTIG_167_p4   4669 pairs of NGS reads, 7.23%
CACAGAAACTGTGAACTCAAtagcctacttgcctgcttttccatccattctttgactgataaacc
atcttgggcctcaacataagcctgaccgccgaccacagcatctcgttgttccgcaaaatagccca
aatacctacaacgtaaATCCAACAGCAACAACCCCCC
>CPCR3_CD12_CONTIG_166_p5   4494 pairs of NGS reads, 6.96%
CACAGAAACTGTGAACTCAatagcctacttgcctgcttttccatccattctttgactgataaacc
atcttgggcctcaacataagcctgaccgccgacccagcatctcgttgttccgcaaaatagcccaa
atacctacaacgtaaaTCCAACAGCAACAACCCCCC
>CPCR3_CD12_CONTIG_164_p6   4090 pairs of NGS reads, 6.33%
CACAGAAACTGTGAACTCAatagcctacttgcctgcttttccatccattctttgactgataaacc
atcttgggcctcaacataagcctgaccgccgaccagcatctcgttgttccgcaaaatagcccaaa

tacctacaacgtaaatCCAACAGCAACAACTTTT
>CPCR3_CD12_CONTIG_212_p7   3500 pairs of NGS reads, 5.42%
CACAGAAACTGTGAACTCAATAGCCTACTTGCCTGCTTTTCCATCCATTCTTTGACTGATAAACC
ATcttgggcctcaacataagcctgaccgccgaccatggctggcaaaagtccaatagcaaacttta
ttttctctggccatgtcagCATCTCGTTGTTCCGCAAAATAGCCCAAATACCTACAACGTAAATC
CAACAGCAACAACAAAA
>CPCR3_CD12_CONTIG_166_p8   2939 pairs of NGS reads, 4.55%
CACAGAAACTGTGAACTCAATagcctacttgcctgcttttccatccattctttgactgataaacc
atcttgggcctcaacataagcctgaccgccgaccacagcatctcgttgttccgcaaaatagccca
aatacctacaacgtaaATCCAACAGCAACAACTTTT
>CPCR3_CD12_CONTIG_165_p9   2804 pairs of NGS reads, 4.34%
CACAGAAACTGTGAACTCAAtagcctacttgcctgcttttccatccattctttgactgataaacc
atcttgggcctcaacataagcctgaccgccgacccagcatctcgttgttccgcaaaatagcccaa
atacctacaacgtaaaTCCAACAGCAACAACTTTT
>CPCR3_CD12_CONTIG_164_p10   1874 pairs of NGS reads, 2.9%
CACAGAAACTGTGAACTCAatagcctacttgcctgcttttccatccattctttgactgataaacc
atcttgggcctcaacataagcctgaccgccgaccagcatctcgttgttccgcaaaatagcccaaa
tacctacaacgtaaatCCAACAGCAACAACAAAA
>CPCR3_CD12_CONTIG_166_p11   1514 pairs of NGS reads, 2.34%
CACAGAAACTGTGAACTCAATAGcctacttgcctgcttttccatccattctttgactgataaacc
atcttgggcctcaacataagcctgaccgccgaccacagcatctcgttgttccgcaaaatagccca
aatacctacaacgtaaATCCAACAGCAACAACAAAA
>CPCR3_CD12_CONTIG_165_p12   1356 pairs of NGS reads, 2.1%
CACAGAAACTGTGAACTCAAtagcctacttgcctgcttttccatccattctttgactgataaacc
atcttgggcctcaacataagcctgaccgccgacccagcatctcgttgttccgcaaaatagcccaa
atacctacaacgtaaaTCCAACAGCAACAACAAAA
>CPCR3_CD12_CONTIG_211_p13   860 pairs of NGS reads, 1.33%
CACAGAAACTGTGAACTCAATAGCCTACTTGCCTGCTTTTCCATCCATTCTTTGACTGATAAACC
ATCTtgggcctcaacataagcctgaccgccgaccatggctggcaaaagtccaatagcaaacttta
ttttctctggccATGTAGCATCTCGTTGTTCCGCAAAATAGCCCAAATACCTACAACGTAAATCC
AACAGCAACAACCCCC
>CPCR3_CD12_CONTIG_211_p14   626 pairs of NGS reads, 0.97%
CACAGAAACTGTGAACTCAATAGCCTACTTGCCTGCTTTTCCATCCATTCTTTGACTGATAAACC
ATCTtgggcctcaacataagcctgaccgccgaccatggctggcaaaagtccaatagcaaacttta
ttttctctggccATGTAGCATCTCGTTGTTCCGCAAAATAGCCCAAATACCTACAACGTAAATCC
AACAGCAACAACTTTT
>CPCR3_CD12_CONTIG_161_p15   567 pairs of NGS reads, 0.87%
CACAGAAACTGTGAACTCAatagcctacttgcctgcttttccatccattctttgactgataaacc
atcttgggcctcaacataagcctgaccgccgaccatctcgttgttccgcaaaatagcccaaatac
ctacaacgtaaatCCAACAGCAACAACCCCC
>CPCR3_CD12_CONTIG_165_p16   569 pairs of NGS reads, 0.88%
CACAGAAACTGTGAACTCAATAgcctacttgcctgcttttccatccattctttgactgataaacc
atcttgggcctcaacataagcctgaccgccgaccaagcatctcgttgttccgcaaaatagcccaa
atacctacaacgtAAATCCAACAGCAACAACCCCC
>CPCR3_CD12_CONTIG_211_p17   432 pairs of NGS reads, 0.66%
CACAGAAACTGTGAACTCAATAGCCTACTTGCCTGCTTTTCCATCCATTCTTTGACTGATAAACC
ATCTtgggcctcaacataagcctgaccgccgaccatggctggcaaaagtccaatagcaaacttta
ttttctctggccATGTAGCATCTCGTTGTTCCGCAAAATAGCCCAAATACCTACAACGTAAATCC
AACAGCAACAACAAAA
>CPCR3_CD12_CONTIG_212_p18   344 pairs of NGS reads, 0.53%
CACAGAAACTGTGAACTCAATAGCCTACTTGCCTGCTTTTCCATCCATTCTTTGACTGATAAACC
ATCTtgggcctcaacataagcctgaccgccgaccaatggctggcaaaagtccaatagcaaacttt

atttctctggccATGTAGCATCTCGTTGTTCCGCAAAATAGCCCAAATACCTACAACGTAAATC
CAACAGCAACAACCCCC
>CPCR3_CD12_CONTIG_165_p19   315 pairs of NGS reads, 0.48%
CACAGAAACTGTGAACTCAATagcctacttgcctgcttttccatccattctttgactgataaacc
atcttgggcctcaacataagcctgaccgccgaccaagcatctcgttgttccgcaaaatagcccaa
atacctacaacgtAAATCCAACAGCAACAACTTTT
>CPCR3_CD12_CONTIG_166_p20   273 pairs of NGS reads, 0.42%
CACAGAAACTGTGAACTCAATAGcctacttgcctgcttttccatccattctttgactgataaacc
atcttgggcctcaacataagcctgaccgccgaccatagcatctcgttgttccgcaaaatagccca
aatacctacaacgTAAATCCAACAGCAACAACCCCC
>CPCR3_CD12_CONTIG_161_p21   258 pairs of NGS reads, 0.39%
CACAGAAACTGTGAACTCaatagcctacttgcctgcttttccatccattctttgactgataaacc
atcttgggcctcaacataagcctgaccgccgaccatctcgttgttccgcaaaatagcccaaatac
ctacaacgtaaatCCAACAGCAACAACTTTT
>CPCR3_CD12_CONTIG_213_p22   230 pairs of NGS reads, 0.35%
CACAGAAACTGTGAACTCAATAGCCTACTTGCCTGCTTTTCCATCCATTCTTTGACTGATAAACC
ATCTTGggcctcaacataagcctgaccgccgaccatggctggcaaaagtccaatagcaaacttta
ttttctctggccATGTACAGCATCTCGTTGTTCCGCAAAATAGCCCAAATACCTACAACGTAAAT
CCAACAGCAACAACCCCC
>CPCR3_CD12_CONTIG_212_p23   219 pairs of NGS reads, 0.33%
CACAGAAACTGTGAACTCAATAGCCTACTTGCCTGCTTTTCCATCCATTCTTTGACTGATAAACC
ATCTtgggcctcaacataagcctgaccgccgaccaatggctggcaaaagtccaatagcaaacttt
atttctctggccATGTAGCATCTCGTTGTTCCGCAAAATAGCCCAAATACCTACAACGTAAATC
CAACAGCAACAACTTTT
>CPCR3_CD12_CONTIG_213_p24   210 pairs of NGS reads, 0.32%
CACAGAAACTGTGAACTCAATAGCCTACTTGCCTGCTTTTCCATCCATTCTTTGACTGATAAACC
ATCTTGggcctcaacataagcctgaccgccgaccaatggctggcaaaagtccaatagcaaacttt
atttctctggccATGTCAGCATCTCGTTGTTCCGCAAAATAGCCCAAATACCTACAACGTAAAT
CCAACAGCAACAACCCCC
>CPCR3_CD12_CONTIG_209_p25   189 pairs of NGS reads, 0.29%
CACAGAAACTGTGAACTCAATAGCCTACTTGCCTGCTTTTCCATCCATTCTTTGACTGATAAACC
ATcttgggcctcaacataagcctgaccgccgaccatggctggcaaaagtccaatagcaaacttta
ttttctctggccatgtcatCTCGTTGTTCCGCAAAATAGCCCAAATACCTACAACGTAAATCCAA
CAGCAACAACCCCC
>CPCR3_CD12_CONTIG_213_p26   180 pairs of NGS reads, 0.27%
CACAGAAACTGTGAACTCAATAGCCTACTTGCCTGCTTTTCCATCCATTCTTTGACTGATAAACC
ATCTTGggcctcaacataagcctgaccgccgaccatggctggcaaaagtccaatagcaaacttta
ttttctctggccATGTTCAGCATCTCGTTGTTCCGCAAAATAGCCCAAATACCTACAACGTAAAT
CCAACAGCAACAACCCCC
>CPCR3_CD12_CONTIG_208_p27   166 pairs of NGS reads, 0.25%
CACAGAAACTGTGAACTCAATAGCCTACTTGCCTGCTTTTCCATCCATTCTTTGACTGATAAACC
Atcttgggcctcaacataagcctgaccgccgaccatggctggcaaaagtccaatagcaaacttta
ttttctctggccATGTATCTCGTTGTTCCGCAAAATAGCCCAAATACCTACAACGTAAATCCAAC
AGCAACAACCCCC
>CPCR3_CD12_CONTIG_163_p28   165 pairs of NGS reads, 0.25%
CACAGAAACTGTGAACTCAATagcctacttgcctgcttttccatccattctttgactgataaacc
atcttgggcctcaacataagcctgaccgccgaccgcatctcgttgttccgcaaaatagcccaaat
acctacaacgtaaATCCAACAGCAACAACCCCC
>CPCR3_CD12_CONTIG_161_p29   163 pairs of NGS reads, 0.25%
CACAGAAACTGTGAACTCAatagcctacttgcctgcttttccatccattctttgactgataaacc
atcttgggcctcaacataagcctgaccgccgaccatctcgttgttccgcaaaatagcccaaatac
ctacaacgtaaatCCAACAGCAACAACAAAA

>CPCR3_CD12_CONTIG_213_p30   147 pairs of NGS reads, 0.22%
CACAGAAACTGTGAACTCAATAGCCTACTTGCCTGCTTTTCCATCCATTCTTTGACTGATAAACC
ATCTTgggcctcaacataagcctgaccgccgaccaatggctggcaaaagtccaatagcaaacttt
atttctctggccATGTCAGCATCTCGTTGTTCCGCAAAATAGCCCAAATACCTACAACGTAAAT
CCAACAGCAACAACTTTT
>CPCR3_CD12_CONTIG_165_p31   138 pairs of NGS reads, 0.21%
CACAGAAACTGTGAACTCAATAGcctacttgcctgcttttccatccattctttgactgataaacc
atcttgggcctcaacataagcctgaccgccgaccaagcatctcgttgttccgcaaaatagcccaa
atacctacaacgtAAATCCAACAGCAACAACAAAA
>CPCR3_CD12_CONTIG_211_p32   128 pairs of NGS reads, 0.19%
CACAGAAACTGTGAACTCAATAGCCTACTTGCCTGCTTTTCCATCCATTCTTTGACTGATAAACC
ATCTtgggcctcaacataagcctgaccgccgaccatggctggcaaaagtccaatagcaaacttta
ttttctctggccATGCAGCATCTCGTTGTTCCGCAAAATAGCCCAAATACCTACAACGTAAATCC
AACAGCAACAACCCCC
>CPCR3_CD12_CONTIG_214_p33   126 pairs of NGS reads, 0.19%
CACAGAAACTGTGAACTCAATAGCCTACTTGCCTGCTTTTCCATCCATTCTTTGACTGATAAACC
ATCTTGGgcctcaacataagcctgaccgccgaccaatggctggcaaaagtccaatagcaaacttt
atttctctggccATGTTCAGCATCTCGTTGTTCCGCAAAATAGCCCAAATACCTACAACGTAAA
TCCAACAGCAACAACCCCC
>CPCR3_CD12_CONTIG_166_p34   117 pairs of NGS reads, 0.18%
CACAGAAACTGTGAACTCAATAGcctacttgcctgcttttccatccattctttgactgataaacc
atcttgggcctcaacataagcctgaccgccgaccatagcatctcgttgttccgcaaaatagccca
aatacctacaacgTAAATCCAACAGCAACAACTTTT
>CPCR3_CD12_CONTIG_213_p35   116 pairs of NGS reads, 0.17%
CACAGAAACTGTGAACTCAATAGCCTACTTGCCTGCTTTTCCATCCATTCTTTGACTGATAAACC
ATCTTGggcctcaacataagcctgaccgccgaccatggctggcaaaagtccaatagcaaacttta
ttttctctggccATGTTCAGCATCTCGTTGTTCCGCAAAATAGCCCAAATACCTACAACGTAAAT
CCAACAGCAACAACTTTT
>CPCR3_CD12_CONTIG_214_p36   110 pairs of NGS reads, 0.17%
CACAGAAACTGTGAACTCAATAGCCTACTTGCCTGCTTTTCCATCCATTCTTTGACTGATAAACC
ATCTTGGgcctcaacataagcctgaccgccgaccaatggctggcaaaagtccaatagcaaacttt
atttctctggccATGTACAGCATCTCGTTGTTCCGCAAAATAGCCCAAATACCTACAACGTAAA
TCCAACAGCAACAACCCCC
>CPCR3_CD12_CONTIG_212_p37   107 pairs of NGS reads, 0.16%
CACAGAAACTGTGAACTCAATAGCCTACTTGCCTGCTTTTCCATCCATTCTTTGACTGATAAACC
ATCTTgggcctcaacataagcctgaccgccgaccaatggctggcaaaagtccaatagcaaacttt
atttctctggccATGTAGCATCTCGTTGTTCCGCAAAATAGCCCAAATACCTACAACGTAAATC
CAACAGCAACAACAAAA
>CPCR3_CD12_CONTIG_207_p38   107 pairs of NGS reads, 0.16%
CACAGAAACTGTGAACTCAATAGCCTACTTGCCTGCTTTTCCATCCATTCTTTGACTGATAAACC
atcttgggcctcaacataagcctgaccgccgaccatggctggcaaaagtccaatagcaaacttta
ttttctctggccATGTTCTCGTTGTTCCGCAAAATAGCCCAAATACCTACAACGTAAATCCAACA
GCAACAACCCCC
>CPCR3_CD12_CONTIG_213_p39   105 pairs of NGS reads, 0.16%
CACAGAAACTGTGAACTCAATAGCCTACTTGCCTGCTTTTCCATCCATTCTTTGACTGATAAACC
ATCTTGggcctcaacataagcctgaccgccgaccatggctggcaaaagtccaatagcaaacttta
ttttctctggccATGTACAGCATCTCGTTGTTCCGCAAAATAGCCCAAATACCTACAACGTAAAT
CCAACAGCAACAACTTTT
>CPCR3_CD12_CONTIG_209_p40   103 pairs of NGS reads, 0.15%
CACAGAAACTGTGAACTCAATAGCCTACTTGCCTGCTTTTCCATCCATTCTTTGACTGATAAACC
ATcttgggcctcaacataagcctgaccgccgaccatggctggcaaaagtccaatagcaaacttta
ttttctctggccatgtcagCTCGTTGTTCCGCAAAATAGCCCAAATACCTACAACGTAAATCCAA

CAGCAACAACTTTT
>CPCR3_CD12_CONTIG_211_p41 98 pairs of NGS reads, 0.15%
CACAGAAACTGTGAACTCAATAGCCTACTTGCCTGCTTTTCCATCCATTCTTTGACTGATAAACC
ATCTtgggcctcaacataagcctgaccgccgaccatggctggcaaaagtccaatagcaaacttta
ttttctctggccATGCAGCATCTCGTTGTTCCGCAAAATAGCCCAAATACCTACAACGTAAATCC
AACAGCAACAACTTTT
>CPCR3_CD12_CONTIG_163_p42 93 pairs of NGS reads, 0.14%
CACAGAAACTGTGAACTCAATagcctacttgcctgcttttccatccattctttgactgataaacc
atcttgggcctcaacataagcctgaccgccgaccgcatctcgttgttccgcaaaatagcccaaat
acctacaacgtaaATCCAACAGCAACAACTTTT
>CPCR3_CD12_CONTIG_210_p43 93 pairs of NGS reads, 0.14%
CACAGAAACTGTGAACTCAATAGCCTACTTGCCTGCTTTTCCATCCATTCTTTGACTGATAAACC
ATCttgggcctcaacataagcctgaccgccgaccatggctggcaaaagtccaatagcaaacttta
ttttctctggccATGTGCATCTCGTTGTTCCGCAAAATAGCCCAAATACCTACAACGTAAATCCA
ACAGCAACAACCCCC
>CPCR3_CD12_CONTIG_127_p44 92 pairs of NGS reads, 0.14%
cacagaaactgtgaactcaatagcctacttgcctgcttttccatccattctttgactgataaacc
atctcgttgttccgcaaaatagcccaaatacctacaacgtaaatccaacagcaacaacccccc
>CPCR3_CD12_CONTIG_166_p45 88 pairs of NGS reads, 0.13%
CACAGAAACTGTGAACTCAATAGCctacttgcctgcttttccatccattctttgactgataaacc
atcttgggcctcaacataagcctgaccgccgacctcagcatctcgttgttccgcaaaatagccca
aatacctacaacgTAAATCCAACAGCAACAACCCCC
>CPCR3_CD12_CONTIG_213_p46 84 pairs of NGS reads, 0.13%
CACAGAAACTGTGAACTCAATAGCCTACTTGCCTGCTTTTCCATCCATTCTTTGACTGATAAACC
ATCTTGggcctcaacataagcctgaccgccgaccaatggctggcaaaagtccaatagcaaacttt
atttctctggccATGTCAGCATCTCGTTGTTCCGCAAAATAGCCCAAATACCTACAACGTAAAT
CCAACAGCAACAACAAAA
>CPCR3_CD12_CONTIG_167_p47 84 pairs of NGS reads, 0.13%
CACAGAAACTGTGAACTCAATAGCCtacttgcctgcttttccatccattctttgactgataaacc
atcttgggcctcaacataagcctgaccgccgaccatcagcatctcgttgttccgcaaaatagccc
aaatacctacaacGTAAATCCAACAGCAACAACCCCC
>CPCR3_CD12_CONTIG_201_p48 80 pairs of NGS reads, 0.12%
CACAGAAACTGTGAACTCAATAGCCTACTTGCCTGCTTTTCCATCCATTCTTTGACTGAtaaacc
atcttgggcctcaacataagcctgaccgccgaccatggctggcaaaagtccaatagcaaacttta
ttttctctggccATGTTGTTCCGCAAAATAGCCCAAATACCTACAACGTAAATCCAACAGCAACA
ACCCCC
>CPCR3_CD12_CONTIG_213_p49 79 pairs of NGS reads, 0.12%
CACAGAAACTGTGAACTCAATAGCCTACTTGCCTGCTTTTCCATCCATTCTTTGACTGATAAACC
ATCTTGggcctcaacataagcctgaccgccgaccatggctggcaaaagtccaatagcaaacttta
ttttctctggccATGTTCAGCATCTCGTTGTTCCGCAAAATAGCCCAAATACCTACAACGTAAAT
CCAACAGCAACAACAAAA
>CPCR3_CD12_CONTIG_206_p50 78 pairs of NGS reads, 0.12%
CACAGAAACTGTGAACTCAATAGCCTACTTGCCTGCTTTTCCATCCATTCTTTGACTGATAAACc
atcttgggcctcaacataagcctgaccgccgaccatggctggcaaaagtccaatagcaaacttta
ttttctctggccatgtctcGTTGTTCCGCAAAATAGCCCAAATACCTACAACGTAAATCCAACAG
CAACAACCCCC
>CPCR3_CD12_CONTIG_212_p51 74 pairs of NGS reads, 0.11%
CACAGAAACTGTGAACTCAATAGCCTACTTGCCTGCTTTTCCATCCATTCTTTGACTGATAAACC
ATCTTgggcctcaacataagcctgaccgccgacctatggctggcaaaagtccaatagcaaacttt
atttctctggccATGTAGCATCTCGTTGTTCCGCAAAATAGCCCAAATACCTACAACGTAAATC
CAACAGCAACAACCCCC
>CPCR3_CD12_CONTIG_213_p52 73 pairs of NGS reads, 0.11%

CACAGAAACTGTGAACTCAATAGCCTACTTGCCTGCTTTTCCATCCATTCTTTGACTGATAAACC
ATCTTGggcctcaacataagcctgaccgccgaccatggctggcaaaagtccaatagcaaacttta
ttttctctggccATGTACAGCATCTCGTTGTTCCGCAAAATAGCCCAAATACCTACAACGTAAAT
CCAACAGCAACAACAAAA
>CPCR3_CD12_CONTIG_208_p53   69 pairs of NGS reads, 0.1%
CACAGAAACTGTGAACTCAATAGCCTACTTGCCTGCTTTTCCATCCATTCTTTGACTGATAAACC
Atcttgggcctcaacataagcctgaccgccgaccatggctggcaaaagtccaatagcaaacttta
ttttctctggccATGTATCTCGTTGTTCCGCAAAATAGCCCAAATACCTACAACGTAAATCCAAC
AGCAACAACTTTT
>CPCR3_CD12_CONTIG_209_p54   68 pairs of NGS reads, 0.1%
CACAGAAACTGTGAACTCAATAGCCTACTTGCCTGCTTTTCCATCCATTCTTTGACTGATAAACC
ATcttgggcctcaacataagcctgaccgccgaccatggctggcaaaagtccaatagcaaacttta
ttttctctggccatgtcatCTCGTTGTTCCGCAAAATAGCCCAAATACCTACAACGTAAATCCAA
CAGCAACAACAAAA

AtFAD DNA 1

>PCR10_CF7_CONTIG_201_p1   15897 pairs of NGS reads, 32.8%
AAGCATGAGGCTATAATGATGTCACTGATAAGGTAGGAGAAAGAGCGAGGGattgagcgtttgaa
acaatgcggcgggattgctttcttcagatctcccaccgagaaaggcggtttctcgcacggcacac
gctttgtggtgtcggtttccGATTTCTTGGAAGAAGTAGGAACCGGCATTCTTCCACCTGCACCC
ATCGAT
>PCR10_CF7_CONTIG_201_p2   13875 pairs of NGS reads, 28.63%
AAGCATGAGGCTATAATGATGTCACTGATAAGGTAGGAGAAAGAGCGAGGGATTGAgcgtttgaa
acaatgcggcgggattgctttcttcagatctcccaccgagaaaggcggtttctcgcacggcacac
gctttgtggtgtcggtttccGATTTCTTGGAAGAAGTAGGAACCGGCATTCTTCCACCTGCACCC
ATAGCT
>PCR10_CF7_CONTIG_201_p3   11656 pairs of NGS reads, 24.05%
AAGCATGAGGCTATAATGATGTCACTGATAAGGTAGGAGAAAGAGCGAGGGATTGAgcgtttgaa
acaatgcggcgggattgctttcttcagatctcccaccgagaaaggcggtttctcgcacggcacac
gctttgtggtgtcggtttccGATTTCTTGGAAGAAGTAGGAACCGGCATTCTTCCACCTGCACCC
ATGCAT
>PCR10_CF7_CONTIG_163_p4   1828 pairs of NGS reads, 3.77%
AAGCATGAGGCTATAATGAtgtcactgataaggtaggagaaagagcgagggattgagcgtttgaa
acaaaggcggtttctcgcacggcacacgctttgtggtgtcggtttccgatttcttggaagaagta
ggaaccggcattctTCCACCTGCACCCATCGAT
>PCR10_CF7_CONTIG_163_p5   1319 pairs of NGS reads, 2.72%
AAGCATGAGGCTATAATGATGtcactgataaggtaggagaaagagcgagggattgagcgtttgaa
acaaaggcggtttctcgcacggcacacgctttgtggtgtcggtttccgatttcttggaagaagta
ggaaccggcattctTCCACCTGCACCCATAGCT
>PCR10_CF7_CONTIG_163_p6   1125 pairs of NGS reads, 2.32%
AAGCATGAGGCTATAATGATgtcactgataaggtaggagaaagagcgagggattgagcgtttgaa
acaaaggcggtttctcgcacggcacacgctttgtggtgtcggtttccgatttcttggaagaagta
ggaaccggcattctTCCACCTGCACCCATGCAT
>PCR10_CF7_CONTIG_162_p7   325 pairs of NGS reads, 0.67%
AAGCATGAGGCTATAATGATgtcactgataaggtaggagaaagagcgagggattgagcgtttgaa
acaaggcggtttctcgcacggcacacgctttgtggtgtcggtttccgatttcttggaagaagtag
gaaccggcattctTCCACCTGCACCCATCGAT
>PCR10_CF7_CONTIG_162_p8   231 pairs of NGS reads, 0.47%
AAGCATGAGGCTATAATGATgtcactgataaggtaggagaaagagcgagggattgagcgtttgaa
acaaggcggtttctcgcacggcacacgctttgtggtgtcggtttccgatttcttggaagaagtag
gaaccggcattctTCCACCTGCACCCATAGCT

>PCR10_CF7_CONTIG_192_p9   198 pairs of NGS reads, 0.4%
AAGCATGAGGCTATAATGATGTCCCCCAACATACCAAAACTAGCTGTCACtccaccggtagtagg
agatgtaagaattgatatatagaataacttttacttgattgataatcacataaaaccgaagaaa
ttttagccatttGCATCAAACTTAAACTTCCTTCTTGCATTCTTCCACCTGCACCCATAGCT
>PCR10_CF7_CONTIG_162_p10   183 pairs of NGS reads, 0.37%
AAGCATGAGGCTATAATGATgtcactgataaggtaggagaaagagcgagggattgagcgtttgaa
acaaggcggtttctcgcacggcacacgctttgtggtgtcggtttccgatttcttggaagaagtag
gaaccggcattctTCCACCTGCACCCATGCAT
>PCR10_CF7_CONTIG_192_p11   101 pairs of NGS reads, 0.2%
AAGCATGAGGCTATAATGATGTCCCCCAACATACCAAAACTAGCTGTCACtccaccggtagtagg
agatgtaagaattgatatatagaataacttttacttgattgataatcacataaaaccgaagaaa
ttttagccatttGCATCAAACTTAAACTTCCTTCTTGCATTCTTCCACCTGCACCCATCGAT
>PCR10_CF7_CONTIG_164_p12   65 pairs of NGS reads, 0.13%
AAGCATGAGGCTATAATGATGTcactgataaggtaggagaaagagcgagggattgagcgtttgaa
acataaggcggtttctcgcacggcacacgctttgtggtgtcggtttccgatttcttggaagaagt
aggaaccggcatTCTTCCACCTGCACCCATCGAT
>PCR10_CF7_CONTIG_202_p13   62 pairs of NGS reads, 0.12%
AAGCATGAGGCTATAATGATGTCACTGATAAGGTAGGAGAAAGAGCGAGGGATTGAGCGTttgaa
acaaatgcggcgggattgctttcttcagatctcccaccgagaaaggcggtttctcgcacggcaca
cgctttgtggtgTCGGTTTCCGATTTCTTGGAAGAAGTAGGAACCGGCATTCTTCCACCTGCACC
CATCGAT
>PCR10_CF7_CONTIG_202_p14   61 pairs of NGS reads, 0.12%
AAGCATGAGGCTATAATGATGTCACTGATAAGGTAGGAGAAAGAGCGAGGGATTGAGCGTttgaa
acaatgcggcgggattgctttcttcagatctcccaccgagaaaaggcggtttctcgcacggcaca
cgctttgtggtgTCGGTTTCCGATTTCTTGGAAGAAGTAGGAACCGGCATTCTTCCACCTGCACC
CATCGAT
>PCR10_CF7_CONTIG_202_p15   60 pairs of NGS reads, 0.12%
AAGCATGAGGCTATAATGATGTCACTGATAAGGTAGGAGAAAGAGCGAGGGATTGAGCGTttgaa
acaatgcggcgggattgctttcttcagatctcccaccgagataaggcggtttctcgcacggcaca
cgctttgtggtgTCGGTTTCCGATTTCTTGGAAGAAGTAGGAACCGGCATTCTTCCACCTGCACC
CATCGAT
>PCR10_CF7_CONTIG_127_p16   57 pairs of NGS reads, 0.11%
aagcatgaggctataatgatgtccacaagctgaacagacccatcatgaacctcaaaaagcgtgtc
tgaacaaaagaaaactttagcgagcctgctttcttgcattcttccacctgcacccatcgat
>PCR10_CF7_CONTIG_164_p17   55 pairs of NGS reads, 0.11%
AAGCATGAGGCTATAATGATGTcactgataaggtaggagaaagagcgagggattgagcgtttgaa
acataaggcggtttctcgcacggcacacgctttgtggtgtcggtttccgatttcttggaagaagt
aggaaccggcatTCTTCCACCTGCACCCATAGCT
>PCR10_CF7_CONTIG_164_p18   53 pairs of NGS reads, 0.1%
AAGCATGAGGCTATAATGATGTcactgataaggtaggagaaagagcgagggattgagcgtttgaa
acataaggcggtttctcgcacggcacacgctttgtggtgtcggtttccgatttcttggaagaagt
aggaaccggcatTCTTCCACCTGCACCCATGCAT
>PCR10_CF7_CONTIG_161_p19   47 pairs of NGS reads, 0.09%
AAGCATGAGGCTATAATGAtgtcactgataaggtaggagaaagagcgagggattgagcgtttgaa
acaggcggtttctcgcacggcacacgctttgtggtgtcggtttccgatttcttggaagaagtagg
aaccggcattctTCCACCTGCACCCATAGCT
>PCR10_CF7_CONTIG_202_p20   46 pairs of NGS reads, 0.09%
AAGCATGAGGCTATAATGATGTCACTGATAAGGTAGGAGAAAGAGCGAGGGATTGAGCGTttgaa
acaatgcggcgggattgctttcttcagatctcccaccgagaaaaggcggtttctcgcacggcaca
cgctttgtggtgTCGGTTTCCGATTTCTTGGAAGAAGTAGGAACCGGCATTCTTCCACCTGCACC
CATAGCT
>PCR10_CF7_CONTIG_161_p21   45 pairs of NGS reads, 0.09%

```
AAGCATGAGGCTATAATGAtgtcactgataaggtaggagaaagagcgagggattgagcgtttgaa
acaggcggtttctcgcacggcacacgctttgtggtgtcggtttccgatttcttggaagaagtagg
aaccggcattctTCCACCTGCACCCATCGAT
>PCR10_CF7_CONTIG_202_p22    44 pairs of NGS reads, 0.09%
AAGCATGAGGCTATAATGATGTCACTGATAAGGTAGGAGAAAGAGCGAGGGATTGAGCGTttgaa
acaatgcggcgggattgctttcttcagatctcccaccgagaaaaggcggtttctcgcacggcaca
cgctttgtggtgTCGGTTTCCGATTTCTTGGAAGAAGTAGGAACCGGCATTCTTCCACCTGCACC
CATGCAT
>PCR10_CF7_CONTIG_164_p23    43 pairs of NGS reads, 0.08%
AAGCATGAGGCTATAATGATGTcactgataaggtaggagaaagagcgagggattgagcgtttgaa
acaaaaggcggtttctcgcacggcacacgctttgtggtgtcggtttccgatttcttggaagaagt
aggaaccggcatTCTTCCACCTGCACCCATCGAT
>PCR10_CF7_CONTIG_127_p24    42 pairs of NGS reads, 0.08%
aagcatgaggctataatgatgtccacaagctgaacagacccatcatgaacctcaaaaagcgtgtc
tgaacaaaagaaaacttttagcgagcctgctttcttgcattcttccacctgcacccatgcat
>PCR10_CF7_CONTIG_107_p25    39 pairs of NGS reads, 0.08%
aagcatgaggctataatgatgtcactgataaggtaggagaaagagcgagggattgagcgtttgaa
acaatgcggcgggattgcattcttccacctgcacccatcgat
>PCR10_CF7_CONTIG_200_p26    38 pairs of NGS reads, 0.07%
AAGCATGAGGCTATAATGATGTCACTGATAAGGTAGGAGAAAGAGCGAGGGATTGAGCgtttgaa
acaatgcggcgggattgctttcttcagatctcccaccgagaaggcggtttctcgcacggcacacg
ctttgtggtgtcGGTTTCCGATTTCTTGGAAGAAGTAGGAACCGGCATTCTTCCACCTGCACCCA
TCGAT
>PCR10_CF7_CONTIG_202_p27    37 pairs of NGS reads, 0.07%
AAGCATGAGGCTATAATGATGTCACTGATAAGGTAGGAGAAAGAGCGAGGGATTGAGCGTttgaa
acaaatgcggcgggattgctttcttcagatctcccaccgagaaaggcggtttctcgcacggcaca
cgctttgtggtgTCGGTTTCCGATTTCTTGGAAGAAGTAGGAACCGGCATTCTTCCACCTGCACC
CATAGCT
>PCR10_CF7_CONTIG_202_p28    36 pairs of NGS reads, 0.07%
AAGCATGAGGCTATAATGATGTCACTGATAAGGTAGGAGAAAGAGCGAGGGATTGAGCGTttgaa
acaatgcggcgggattgctttcttcagatctcccaccgagataaggcggtttctcgcacggcaca
cgctttgtggtgTCGGTTTCCGATTTCTTGGAAGAAGTAGGAACCGGCATTCTTCCACCTGCACC
CATAGCT
>PCR10_CF7_CONTIG_200_p29    34 pairs of NGS reads, 0.07%
AAGCATGAGGCTATAATGATGTCACTGATAAGGTAGGAGAAAGAGCGAGGGATTGAGCgtttgaa
acaatgcggcgggattgctttcttcagatctcccaccgagaaggcggtttctcgcacggcacacg
ctttgtggtgtcGGTTTCCGATTTCTTGGAAGAAGTAGGAACCGGCATTCTTCCACCTGCACCCA
TGCAT
>PCR10_CF7_CONTIG_202_p30    32 pairs of NGS reads, 0.06%
AAGCATGAGGCTATAATGATGTCACTGATAAGGTAGGAGAAAGAGCGAGGGATTGAGCGTttgaa
acaaatgcggcgggattgctttcttcagatctcccaccgagaaaggcggtttctcgcacggcaca
cgctttgtggtgTCGGTTTCCGATTTCTTGGAAGAAGTAGGAACCGGCATTCTTCCACCTGCACC
CATGCAT
>PCR10_CF7_CONTIG_202_p31    30 pairs of NGS reads, 0.06%
AAGCATGAGGCTATAATGATGTCACTGATAAGGTAGGAGAAAGAGCGAGGGATTGAGCGTttgaa
acaatgcggcgggattgctttcttcagatctcccaccgagataaggcggtttctcgcacggcaca
cgctttgtggtgTCGGTTTCCGATTTCTTGGAAGAAGTAGGAACCGGCATTCTTCCACCTGCACC
CATGCAT
>PCR10_CF7_CONTIG_202_p32    30 pairs of NGS reads, 0.06%
AAGCATGAGGCTATAATGATGTCACTGATAAGGTAGGAGAAAGAGCGAGGGATTGAGCGTttgaa
acatctcggtgggagatctgaagaaagcaatcccgccgcattaaggcggtttctcgcacggcaca
cgctttgtggtgTCGGTTTCCGATTTCTTGGAAGAAGTAGGAACCGGCATTCTTCCACCTGCACC
```

CATAGCT
>PCR10_CF7_CONTIG_161_p33   27 pairs of NGS reads, 0.05%
AAGCATGAGGCTATAATGAtgtcactgataaggtaggagaaagagcgagggattgagcgtttgaa
acaggcggtttctcgcacggcacacgctttgtggtgtcggtttccgatttcttggaagaagtagg
aaccggcattctTCCACCTGCACCCATGCAT
>PCR10_CF7_CONTIG_200_p34   24 pairs of NGS reads, 0.04%
AAGCATGAGGCTATAATGATGTCACTGATAAGGTAGGAGAAAGAGCGAGGGATTGAGCgtttgaa
acaatgcggcgggattgctttcttcagatctcccaccgagaaaggcggtttctcgcacggcacac
gctttgtggtgtcggtttccGATTTCTTGGAAGAAGTAGGAACCGGCATTCTTCCACCTGCACCC
ATCAT
>PCR10_CF7_CONTIG_164_p35   23 pairs of NGS reads, 0.04%
AAGCATGAGGCTATAATGATGTcactgataaggtaggagaaagagcgagggattgagcgtttgaa
acaaaaggcggtttctcgcacggcacacgctttgtggtgtcggtttccgatttcttggaagaagt
aggaaccggcatTCTTCCACCTGCACCCATAGCT
>PCR10_CF7_CONTIG_200_p36   23 pairs of NGS reads, 0.04%
AAGCATGAGGCTATAATGATGTCACTGATAAGGTAGGAGAAAGAGCGAGGGATTGAGCgtttgaa
acaatgcggcgggattgctttcttcagatctcccaccgagaaaggcggtttctcgcacggcacacg
ctttgtggtgtcGGTTTCCGATTTCTTGGAAGAAGTAGGAACCGGCATTCTTCCACCTGCACCCA
TAGCT
>PCR10_CF7_CONTIG_127_p37   23 pairs of NGS reads, 0.04%
aagcatgaggctataatgatgtccacaagctgaacagacccatcatgaacctcaaaaagcgtgtc
tgaacaaaagaaaactttagcgagcctgctttcttgcattcttccacctgcacccatagct
>PCR10_CF7_CONTIG_200_p38   23 pairs of NGS reads, 0.04%
AAGCATGAGGCTATAATGATGTCACTGATAAGGTAGGAGAAAGAGCGAGGGATTGAGCgtttgaa
acaatgcggcgggattgctttcttcagatctcccaccgagaaaggcggtttctcgcacggcacac
gctttgtggtgtcggtttccGATTTCTTGGAAGAAGTAGGAACCGGCATTCTTCCACCTGCACCC
ATACT
>PCR10_CF7_CONTIG_164_p39   23 pairs of NGS reads, 0.04%
AAGCATGAGGCTATAATGATGTcactgataaggtaggagaaagagcgagggattgagcgtttgaa
acaaaaggcggtttctcgcacggcacacgctttgtggtgtcggtttccgatttcttggaagaagt
aggaaccggcatTCTTCCACCTGCACCCATGCAT
>PCR10_CF7_CONTIG_191_p40   22 pairs of NGS reads, 0.04%
CTATAATGATGTCACTGATAAGGTAGGAGAAAGAGCGAGGGATTGAgcgtttgaaacaatgcggc
gggattgctttcttcagatctcccaccgagaaaggcggtttctcgcacggcacacgctttgtggt
gtcggtttccgatttcTTGGAAGAAGTAGGAACCGGCATTCTTCCACCTGCACCCATAGCT
>PCR10_CF7_CONTIG_246_p41   20 pairs of NGS reads, 0.04%
AAGCATGAGGCTATAATGATGTCAGTTGGTGGATCTCGTCGACGAAAGGTTCTCGGTCAGGATCT
GCTTGGTGATGACAAGCGACAGGGAACCGTTCTCAAGCTcaaggaacttgtcggcattgcgcggc
agaggcaccttgTTCTTGCGGCAGAACAGGATCAGGAAAGCGGCGGCTTCGCCCTGGGTGAAGAC
GAGATCGATCAGCTGCCGGGCACCCGCATTCTTCCACCTGCACCCATAGCT
>PCR10_CF7_CONTIG_189_p42   20 pairs of NGS reads, 0.04%
AAGCATGAGGCTATAATGATGTCAGGTATGAACATTCTTACGGCATGattagtctctctctcaga
ttgaatcgttgatctctcctttgtatggtaacaggataagaatgtggtgactgtgtttagtgcac
caaactattgctACCGGTGTGGAAACATGGCTGCCATTCTTCCACCTGCACCCATAGCT
>PCR10_CF7_CONTIG_190_p43   20 pairs of NGS reads, 0.04%
AAGCATGAGGCTATAATGATGTCACTGATAAGGTAGGAGAAAGagcgagggattgagcgtttgaa
acaatgcggcgggattgctttcttcagatctcccaccgagaaaggcggtttctcgcacggcacac
gctttgtggtgtcggtttccGATTTCTTGGAAGAAGTAGGAACCGGCATTCTTCCACCTG
>PCR10_CF7_CONTIG_202_p44   20 pairs of NGS reads, 0.04%
AAGCATGAGGCTATAATGATGTCACTGATAAGGTAGGAGAAAGAGCGAGGGATTGAGCGTttgaa
acatctcggtgggagatctgaagaaagcaatcccgccgcattaaggcggtttctcgcacggcaca
cgctttgtggtgTCGGTTTCCGATTTCTTGGAAGAAGTAGGAACCGGCATTCTTCCACCTGCACC

CATGCAT
>PCR10_CF7_CONTIG_202_p45   18 pairs of NGS reads, 0.03%
AAGCATGAGGCTATAATGATGTCACTGATAAGGTAGGAGAAAGAGCGAGGGATTGAGCGTttgaa
acatctcggtgggagatctgaagaaagcaatcccgccgcattaaggcggtttctcgcacggcaca
cgctttgtggtgTCGGTTTCCGATTTCTTGGAAGAAGTAGGAACCGGCATTCTTCCACCTGCACC
CATCGAT
>PCR10_CF7_CONTIG_188_p46   18 pairs of NGS reads, 0.03%
AAGCATGAGGCTATAATGATGTGTTTCGTGTTATATTAATGATTTTctaaatagattaaattgtg
ttgaattgggcctaattggattgaataaaggcctgaaaaatcctataaaccacacatcacggcgt
gcgacgtgagtgTTGCGGTCAATGAACGACACGCATTCTTCCACCTGCACCCATCGAT

AtFAD DNA 2

>PCR11_CF8_CONTIG_201_p1   15989 pairs of NGS reads, 28.32%
AAGCATGAGGCTATAATGATGTCACTGATAAGGTAGGAGAAAGAGCGAGGGattgagcgtttgaa
acaatgcggcgggattgctttcttcagatctcccaccgagaaaggcggtttctcgcacggcacac
gctttgtggtgtcggtttccgATTTCTTGGAAGAAGTAGGAACCGGCATTCTTCCACCTGCACCC
ATGCAT
>PCR11_CF8_CONTIG_201_p2   15491 pairs of NGS reads, 27.44%
AAGCATGAGGCTATAATGATGTCACTGATAAGGTAGGAGAAAGAGCGAGGGATTGAgcgtttgaa
acaatgcggcgggattgctttcttcagatctcccaccgagaaaggcggtttctcgcacggcacac
gctttgtggtgtcggtttccgATTTCTTGGAAGAAGTAGGAACCGGCATTCTTCCACCTGCACCC
ATAGCT
>PCR11_CF8_CONTIG_201_p3   15142 pairs of NGS reads, 26.82%
AAGCATGAGGCTATAATGATGTCACTGATAAGGTAGGAGAAAGAGCGAGGGATTGAgcgtttgaa
acaatgcggcgggattgctttcttcagatctcccaccgagaaaggcggtttctcgcacggcacac
gctttgtggtgtcggtttccgATTTCTTGGAAGAAGTAGGAACCGGCATTCTTCCACCTGCACCC
ATCGAT
>PCR11_CF8_CONTIG_163_p4   2415 pairs of NGS reads, 4.27%
AAGCATGAGGCTATAAtgatgtcactgataaggtaggagaaagagcgagggattgagcgtttgaa
acaaaggcggtttctcgcacggcacacgctttgtggtgtcggtttccgatttcttggaagaagta
ggaaccggcattctTCCACCTGCACCCATAGCT
>PCR11_CF8_CONTIG_163_p5   1805 pairs of NGS reads, 3.19%
AAGCATGAGGCTATAATGAtgtcactgataaggtaggagaaagagcgagggattgagcgtttgaa
acaaaggcggtttctcgcacggcacacgctttgtggtgtcggtttccgatttcttggaagaagta
ggaaccggcattctTCCACCTGCACCCATGCAT
>PCR11_CF8_CONTIG_163_p6   1685 pairs of NGS reads, 2.98%
AAGCATGAGGCTATAATGAtgtcactgataaggtaggagaaagagcgagggattgagcgtttgaa
acaaaggcggtttctcgcacggcacacgctttgtggtgtcggtttccgatttcttggaagaagta
ggaaccggcattctTCCACCTGCACCCATCGAT
>PCR11_CF8_CONTIG_162_p7   445 pairs of NGS reads, 0.78%
AAGCATGAGGCTATAATGATgtcactgataaggtaggagaaagagcgagggattgagcgtttgaa
acaaggcggtttctcgcacggcacacgctttgtggtgtcggtttccgatttcttggaagaagtag
gaaccggcattcttCCACCTGCACCCATAGCT
>PCR11_CF8_CONTIG_162_p8   387 pairs of NGS reads, 0.68%
AAGCATGAGGCTATAATGatgtcactgataaggtaggagaaagagcgagggattgagcgtttgaa
acaaggcggtttctcgcacggcacacgctttgtggtgtcggtttccgatttcttggaagaagtag
gaaccggcattcttCCACCTGCACCCATGCAT
>PCR11_CF8_CONTIG_162_p9   345 pairs of NGS reads, 0.61%
AAGCATGAGGCTATAATGATgtcactgataaggtaggagaaagagcgagggattgagcgtttgaa
acaaggcggtttctcgcacggcacacgctttgtggtgtcggtttccgatttcttggaagaagtag
gaaccggcattcttCCACCTGCACCCATCGAT

>PCR11_CF8_CONTIG_192_p10   198 pairs of NGS reads, 0.35%
AAGCATGAGGCTATAATGATGTCCCCCAACATACCAAAACTAGCTGTCACtccaccggtagtagg
agatgtaagaattgatatatagaataacttttacttgattgataatcacataaaaccgaagaaa
ttttagccatttGCATCAAACTTAAACTTCCTTCTTGCATTCTTCCACCTGCACCCATAGCT
>PCR11_CF8_CONTIG_164_p11   156 pairs of NGS reads, 0.27%
AAGCATGAGGCTATAATGATGTcactgataaggtaggagaaagagcgagggattgagcgtttgaa
acataaggcggtttctcgcacggcacacgctttgtggtgtcggtttccgatttcttggaagaagt
aggaaccggcatTCTTCCACCTGCACCCATAGCT
>PCR11_CF8_CONTIG_192_p12   133 pairs of NGS reads, 0.23%
AAGCATGAGGCTATAATGATGTCCCCCAACATACCAAAACTAGCTGTCACtccaccggtagtagg
agatgtaagaattgatatatagaataacttttacttgattgataatcacataaaaccgaagaaa
ttttagccatttGCATCAAACTTAAACTTCCTTCTTGCATTCTTCCACCTGCACCCATCGAT
>PCR11_CF8_CONTIG_164_p13   126 pairs of NGS reads, 0.22%
AAGCATGAGGCTATAATGATGTcactgataaggtaggagaaagagcgagggattgagcgtttgaa
acataaggcggtttctcgcacggcacacgctttgtggtgtcggtttccgatttcttggaagaagt
aggaaccggcatTCTTCCACCTGCACCCATGCAT
>PCR11_CF8_CONTIG_164_p14   121 pairs of NGS reads, 0.21%
AAGCATGAGGCTATAATGATGTcactgataaggtaggagaaagagcgagggattgagcgtttgaa
acataaggcggtttctcgcacggcacacgctttgtggtgtcggtttccgatttcttggaagaagt
aggaaccggcatTCTTCCACCTGCACCCATCGAT
>PCR11_CF8_CONTIG_202_p15   95 pairs of NGS reads, 0.16%
AAGCATGAGGCTATAATGATGTCACTGATAAGGTAGGAGAAAGAGCGAGGGATTGAGCGTttgaa
acaatgcggcgggattgctttcttcagatctcccaccgagaaaaggcggtttctcgcacggcaca
cgctttgtggtgTCGGTTTCCGATTTCTTGGAAGAAGTAGGAACCGGCATTCTTCCACCTGCACC
CATGCAT
>PCR11_CF8_CONTIG_202_p16   78 pairs of NGS reads, 0.13%
AAGCATGAGGCTATAATGATGTCACTGATAAGGTAGGAGAAAGAGCGAGGGATTGAGCGTttgaa
acaatgcggcgggattgctttcttcagatctcccaccgagaaaaggcggtttctcgcacggcaca
cgctttgtggtgTCGGTTTCCGATTTCTTGGAAGAAGTAGGAACCGGCATTCTTCCACCTGCACC
CATAGCT
>PCR11_CF8_CONTIG_202_p17   77 pairs of NGS reads, 0.13%
AAGCATGAGGCTATAATGATGTCACTGATAAGGTAGGAGAAAGAGCGAGGGATTGAGCGTttgaa
acaatgcggcgggattgctttcttcagatctcccaccgagaaaaggcggtttctcgcacggcaca
cgctttgtggtgTCGGTTTCCGATTTCTTGGAAGAAGTAGGAACCGGCATTCTTCCACCTGCACC
CATCGAT
>PCR11_CF8_CONTIG_202_p18   65 pairs of NGS reads, 0.11%
AAGCATGAGGCTATAATGATGTCACTGATAAGGTAGGAGAAAGAGCGAGGGATTGAGCGTttgaa
acaatgcggcgggattgctttcttcagatctcccaccgagataaggcggtttctcgcacggcaca
cgctttgtggtgtCGGTTTCCGATTTCTTGGAAGAAGTAGGAACCGGCATTCTTCCACCTGCACC
CATAGCT
>PCR11_CF8_CONTIG_202_p19   59 pairs of NGS reads, 0.1%
AAGCATGAGGCTATAATGATGTCACTGATAAGGTAGGAGAAAGAGCGAGGGATTGAGCGTttgaa
acaatgcggcgggattgctttcttcagatctcccaccgagataaggcggtttctcgcacggcaca
cgctttgtggtgtCGGTTTCCGATTTCTTGGAAGAAGTAGGAACCGGCATTCTTCCACCTGCACC
CATGCAT
>PCR11_CF8_CONTIG_202_p20   59 pairs of NGS reads, 0.1%
AAGCATGAGGCTATAATGATGTCACTGATAAGGTAGGAGAAAGAGCGAGGGATTGAGCGTttgaa
acaaatgcggcgggattgctttcttcagatctcccaccgagaaaggcggtttctcgcacggcaca
cgctttgtggtgTCGGTTTCCGATTTCTTGGAAGAAGTAGGAACCGGCATTCTTCCACCTGCACC
CATCGAT
>PCR11_CF8_CONTIG_202_p21   56 pairs of NGS reads, 0.09%
AAGCATGAGGCTATAATGATGTCACTGATAAGGTAGGAGAAAGAGCGAGGGATTGAGCGTttgaa

acaaatgcggcgggattgctttcttcagatctcccaccgagaaaggcggtttctcgcacggcaca
cgctttgtggtgTCGGTTTCCGATTTCTTGGAAGAAGTAGGAACCGGCATTCTTCCACCTGCACC
CATAGCT
>PCR11_CF8_CONTIG_164_p22   52 pairs of NGS reads, 0.09%
AAGCATGAGGCTATAATGATGTcactgataaggtaggagaaagagcgagggattgagcgtttgaa
acaaaaggcggtttctcgcacggcacacgctttgtggtgtcggtttccgatttcttggaagaagt
aggaaccggcatTCTTCCACCTGCACCCATCGAT
>PCR11_CF8_CONTIG_202_p23   48 pairs of NGS reads, 0.08%
AAGCATGAGGCTATAATGATGTCACTGATAAGGTAGGAGAAAGAGCGAGGGATTGAGCGTttgaa
acaaatgcggcgggattgctttcttcagatctcccaccgagaaaggcggtttctcgcacggcaca
cgctttgtggtgTCGGTTTCCGATTTCTTGGAAGAAGTAGGAACCGGCATTCTTCCACCTGCACC
CATGCAT
>PCR11_CF8_CONTIG_200_p24   45 pairs of NGS reads, 0.07%
AAGCATGAGGCTATAATGATGTCACTGATAAGGTAGGAGAAAGAGCGAGGGATTGAGCgtttgaa
acaatgcggcgggattgctttcttcagatctcccaccgagaaggcggtttctcgcacggcacacg
ctttgtggtgtcGGTTTCCGATTTCTTGGAAGAAGTAGGAACCGGCATTCTTCCACCTGCACCCA
TAGCT
>PCR11_CF8_CONTIG_202_p25   44 pairs of NGS reads, 0.07%
AAGCATGAGGCTATAATGATGTCACTGATAAGGTAGGAGAAAGAGCGAGGGATTGAGCGTttgaa
acaatgcggcgggattgctttcttcagatctcccaccgagataaggcggtttctcgcacggcaca
cgctttgtggtgtCGGTTTCCGATTTCTTGGAAGAAGTAGGAACCGGCATTCTTCCACCTGCACC
CATCGAT
>PCR11_CF8_CONTIG_200_p26   41 pairs of NGS reads, 0.07%
AAGCATGAGGCTATAATGATGTCACTGATAAGGTAGGAGAAAGAGCGAGGGATTGAGCgtttgaa
acaatgcggcgggattgctttcttcagatctcccaccgagaaggcggtttctcgcacggcacacg
ctttgtggtgtcGGTTTCCGATTTCTTGGAAGAAGTAGGAACCGGCATTCTTCCACCTGCACCCA
TGCAT
>PCR11_CF8_CONTIG_203_p27   39 pairs of NGS reads, 0.06%
AAGCATGAGGCTATAATGATGTCACTGATAAGGTAGGAGAAAGAGCGAGGGATTGAGCGTTtgaa
acaaatgcggcgggattgctttcttcagatctcccaccgagaaaaggcggtttctcgcacggcac
acgctttgtggtGTCGGTTTCCGATTTCTTGGAAGAAGTAGGAACCGGCATTCTTCCACCTGCAC
CCATAGCT
>PCR11_CF8_CONTIG_164_p28   38 pairs of NGS reads, 0.06%
AAGCATGAGGCTATAATGATGTcactgataaggtaggagaaagagcgagggattgagcgtttgaa
acaaaaggcggtttctcgcacggcacacgctttgtggtgtcggtttccgatttcttggaagaagt
aggaaccggcatTCTTCCACCTGCACCCATAGCT
>PCR11_CF8_CONTIG_164_p29   36 pairs of NGS reads, 0.06%
AAGCATGAGGCTATAATGATGTcactgataaggtaggagaaagagcgagggattgagcgtttgaa
acaaaaggcggtttctcgcacggcacacgctttgtggtgtcggtttccgatttcttggaagaagt
aggaaccggcatTCTTCCACCTGCACCCATGCAT
>PCR11_CF8_CONTIG_200_p30   32 pairs of NGS reads, 0.05%
AAGCATGAGGCTATAATGATGTCACTGATAAGGTAGGAGAAAGAGCGAGGGATTGAGCgtttgaa
acaatgcggcgggattgctttcttcagatctcccaccgagaaggcggtttctcgcacggcacacg
ctttgtggtgtcGGTTTCCGATTTCTTGGAAGAAGTAGGAACCGGCATTCTTCCACCTGCACCCA
TCGAT
>PCR11_CF8_CONTIG_161_p31   32 pairs of NGS reads, 0.05%
AAGCATGAGGCTATAATGAtgtcactgataaggtaggagaaagagcgagggattgagcgtttgaa
acaggcggtttctcgcacggcacacgctttgtggtgtcggtttccgatttcttggaagaagtagg
aaccggcattctTCCACCTGCACCCATGCAT
>PCR11_CF8_CONTIG_206_p32   30 pairs of NGS reads, 0.05%
AAGCATGAGGCTATAATGATGTCGTTGAGTACTGAAGAGAAAGCTAAGATGAGGCAACGGTTACt
gagtggtaaaggtgttgaggaggatgatgaagaagagaagaggaagaagaaggggaaaggaaga

atccgaatctggATGTGTTATCTGCTCTTGGAGATAACACATCCAGATTCGCATTCTTCCACCTG
CACCCATCGAT
>PCR11_CF8_CONTIG_161_p33   30 pairs of NGS reads, 0.05%
AAGCATGAGGCTATAATGAtgtcactgataaggtaggagaaagagcgagggattgagcgtttgaa
acaggcggtttctcgcacggcacacgctttgtggtgtcggtttccgatttcttggaagaagtagg
aaccggcattctTCCACCTGCACCCATAGCT
>PCR11_CF8_CONTIG_202_p34   30 pairs of NGS reads, 0.05%
AAGCATGAGGCTATAATGATGTCACTGATAAGGTAGGAGAAAGAGCGAGGGATTGAGCGTttgaa
acatctcggtgggagatctgaagaaagcaatcccgccgcattaaggcggtttctcgcacggcaca
cgctttgtggtgTCGGTTTCCGATTTCTTGGAAGAAGTAGGAACCGGCATTCTTCCACCTGCACC
CATGCAT
>PCR11_CF8_CONTIG_202_p35   29 pairs of NGS reads, 0.05%
AAGCATGAGGCTATAATGATGTCACTGATAAGGTAGGAGAAAGAGCGAGGGATTGAGCGTttgaa
acatctcggtgggagatctgaagaaagcaatcccgccgcattaaggcggtttctcgcacggcaca
cgctttgtggtgTCGGTTTCCGATTTCTTGGAAGAAGTAGGAACCGGCATTCTTCCACCTGCACC
CATAGCT
>PCR11_CF8_CONTIG_155_p36   28 pairs of NGS reads, 0.04%
AAGCATGAGGCTAtaatgatgtcactgataaggtaggagaaagagcgagggattgagcgtttgaa
actttctcgcacggcacacgctttgtggtgtcggtttccgatttcttggaagaagtaggaaccgg
cattcttccaccTGCACCCATAGCT
>PCR11_CF8_CONTIG_203_p37   28 pairs of NGS reads, 0.04%
AAGCATGAGGCTATAATGATGTCACTGATAAGGTAGGAGAAAGAGCGAGGGATTGAGCGTTtgaa
acaaatgcggcgggattgctttcttcagatctcccaccgagaaaaggcggtttctcgcacggcac
acgctttgtggtGTCGGTTTCCGATTTCTTGGAAGAAGTAGGAACCGGCATTCTTCCACCTGCAC
CCATCGAT
>PCR11_CF8_CONTIG_202_p38   27 pairs of NGS reads, 0.04%
AAGCATGAGGCTATAATGATGTCACTGATAAGGTAGGAGAAAGAGCGAGGGATTGAGCGTttgaa
acatctcggtgggagatctgaagaaagcaatcccgccgcattaaggcggtttctcgcacggcaca
cgctttgtggtgTCGGTTTCCGATTTCTTGGAAGAAGTAGGAACCGGCATTCTTCCACCTGCACC
CATCGAT
>PCR11_CF8_CONTIG_203_p39   25 pairs of NGS reads, 0.04%
AAGCATGAGGCTATAATGATGTCATCCTTGTTGGATCTGCAACCAACTTTGTTAGTCTGCTttca
tatctattatcttaacttgtatggccgctgaatttccctttggtttgcagcaagttcctgatgag
attgaaagagtgTTAGTTGGGATACAAGCTTATTTGAGCATCCGAAAGAATTCTTCCACCTGCAC
CCATGCAT
>PCR11_CF8_CONTIG_161_p40   24 pairs of NGS reads, 0.04%
AAGCATGAGGCTATAATGAtgtcactgataaggtaggagaaagagcgagggattgagcgtttgaa
acaggcggtttctcgcacggcacacgctttgtggtgtcggtttccgatttcttggaagaagtagg
aaccggcattctTCCACCTGCACCCATCGAT
>PCR11_CF8_CONTIG_200_p41   24 pairs of NGS reads, 0.04%
AAGCATGAGGCTATAATGATGTCACTGATAAGGTAGGAGAAAGAGCGAGGGATTGAGCgtttgaa
acaatgcggcgggattgctttcttcagatctcccaccgagaaaggcggtttctcgcacggcacac
gctttgtggtgtcggtttccgATTTCTTGGAAGAAGTAGGAACCGGCATTCTTCCACCTGCACCC
ATCAT
>PCR11_CF8_CONTIG_203_p42   23 pairs of NGS reads, 0.04%
AAGCATGAGGCTATAATGATGTCACTGATAAGGTAGGAGAAAGAGCGAGGGATTGAGCGTTtgaa
acaaatgcggcgggattgctttcttcagatctcccaccgagataaggcggtttctcgcacggcac
acgctttgtggtGTCGGTTTCCGATTTCTTGGAAGAAGTAGGAACCGGCATTCTTCCACCTGCAC
CCATAGCT
>PCR11_CF8_CONTIG_200_p43   23 pairs of NGS reads, 0.04%
AAGCATGAGGCTATAATGATGTCACTGATAAGGTAGGAGAAAGAGCGAGGGATTGAGCgtttgaa
acaatgcggcgggattgctttcttcagatctcccaccgagaaaggcggtttctcgcacggcacac

gctttgtggtgtcggtttccgATTTCTTGGAAGAAGTAGGAACCGGCATTCTTCCACCTGCACCC
ATACT
>PCR11_CF8_CONTIG_203_p44   22 pairs of NGS reads, 0.03%
AAGCATGAGGCTATAATGATGTCACTGATAAGGTAGGAGAAAGAGCGAGGGATTGAGCGTTtgaa
acaaatgcggcgggattgctttcttcagatctcccaccgagataaggcggtttctcgcacggcac
acgctttgtggtGTCGGTTTCCGATTTCTTGGAAGAAGTAGGAACCGGCATTCTTCCACCTGCAC
CCATCGAT
>PCR11_CF8_CONTIG_193_p45   20 pairs of NGS reads, 0.03%
AAGCATGAGGCTATAATGATGTCACTGATAAGGTAGGAGAAAGAGCGAGGGattgagcgtttgaa
acaatgcggcgggattgctttcttcagatctcccaccgagattctcgcacggcacacgctttgtg
gtgtcggtttccGATTTCTTGGAAGAAGTAGGAACCGGCATTCTTCCACCTGCACCCATGCAT
>PCR11_CF8_CONTIG_203_p46   19 pairs of NGS reads, 0.03%
AAGCATGAGGCTATAATGATGTCACTGATAAGGTAGGAGAAAGAGCGAGGGATTGAGCGTTtgaa
acaaatgcggcgggattgctttcttcagatctcccaccgagaaaaggcggtttctcgcacggcac
acgctttgtggtGTCGGTTTCCGATTTCTTGGAAGAAGTAGGAACCGGCATTCTTCCACCTGCAC
CCATGCAT
>PCR11_CF8_CONTIG_173_p47   19 pairs of NGS reads, 0.03%
AAGCATGAGGCTATAATGATGTCTGCGGGCCatatctccggtggccacctcaaccccgccgtcac
cctcggtctactcttgggtggccacatcagcgtattccgtgcattcctttattggattgatcaat
tgttggcctcctCCGCAGCATTCTTCCACCTGCACCCATAGCT
>PCR11_CF8_CONTIG_203_p48   18 pairs of NGS reads, 0.03%
AAGCATGAGGCTATAATGATGTCACTGATAAGGTAGGAGAAAGAGCGAGGGATTGAGCGTTtgaa
acaaatgcggcgggattgctttcttcagatctcccaccgagataaggcggtttctcgcacggcac
acgctttgtggtGTCGGTTTCCGATTTCTTGGAAGAAGTAGGAACCGGCATTCTTCCACCTGCAC
CCATGCAT
>PCR11_CF8_CONTIG_188_p49   18 pairs of NGS reads, 0.03%
AAGCATGAGGCTATAATGATGTGTTTCGTGTTATATTAATGATTTTctaaatagattaaattgtg
ttgaattgggcctaattggattgaataaaggcctgaaaaatcctataaaccacacatcacggcgt
gcgacgtgagtgTTGCGGTCAATGAACGACACGCATTCTTCCACCTGCACCCATAGCT
>PCR11_CF8_CONTIG_192_p50   16 pairs of NGS reads, 0.02%
AAGCATGAGGCTATAATGATGTCCCCCAACATACCAAAACTAGCTGTCACtccaccggtagtagg
agatgtaagaattgatatatagaataacttttttacttgattgataatcacataaaaccgaagaaa
ttttagccatttGCATCAAACTTAAACTTCCTTCTTGCATTCTTCCACCTGCACCCATGCAT

AtFAD DNA 3

>PCR12_CF9_CONTIG_202_p1   18290 pairs of NGS reads, 28.7%
AAAGCATGAGGCTATAATGATGTCACTGATAAGGTAGGAGAAAGAGCGAGGGattgagcgtttga
aacaatgcggcgggattgctttcttcagatctcccaccgagaaaggcggtttctcgcacggcaca
cgctttgtggtgtcggtTTCCGATTTCTTGGAAGAAGTAGGAACCGGCATTCTTCCACCTGCACC
CATCGAT
>PCR12_CF9_CONTIG_202_p2   17619 pairs of NGS reads, 27.65%
AAAGCATGAGGCTATAATGATGTCACTGATAAGGTAGGAGAAAGAGCGAGGGATTGAgcgtttga
aacaatgcggcgggattgctttcttcagatctcccaccgagaaaggcggtttctcgcacggcaca
cgctttgtggtgtcggtTTCCGATTTCTTGGAAGAAGTAGGAACCGGCATTCTTCCACCTGCACC
CATGCAT
>PCR12_CF9_CONTIG_202_p3   16992 pairs of NGS reads, 26.66%
AAAGCATGAGGCTATAATGATGTCACTGATAAGGTAGGAGAAAGAGCGAGGGATTGAgcgtttga
aacaatgcggcgggattgctttcttcagatctcccaccgagaaaggcggtttctcgcacggcaca
cgctttgtggtgtcggtTTCCGATTTCTTGGAAGAAGTAGGAACCGGCATTCTTCCACCTGCACC
CATAGCT

>PCR12_CF9_CONTIG_163_p4   1812 pairs of NGS reads, 2.84%
AAGCATGAGGCTATAAtgatgtcactgataaggtaggagaaagagcgagggattgagcgtttgaa
acaaaggcggtttctcgcacggcacacgctttgtggtgtcggtttccgatttcttggaagaagta
ggaaccggcattctTCCACCTGCACCCATGCAT
>PCR12_CF9_CONTIG_163_p5   1745 pairs of NGS reads, 2.73%
AAGCATGAGGCTATAATGAtgtcactgataaggtaggagaaagagcgagggattgagcgtttgaa
acaaaggcggtttctcgcacggcacacgctttgtggtgtcggtttccgatttcttggaagaagta
ggaaccggcattctTCCACCTGCACCCATCGAT
>PCR12_CF9_CONTIG_163_p6   1696 pairs of NGS reads, 2.66%
AAGCATGAGGCTATAATGATgtcactgataaggtaggagaaagagcgagggattgagcgtttgaa
acaaaggcggtttctcgcacggcacacgctttgtggtgtcggtttccgatttcttggaagaagta
ggaaccggcattctTCCACCTGCACCCATAGCT
>PCR12_CF9_CONTIG_162_p7   291 pairs of NGS reads, 0.45%
AAGCATGAGGCTATAATGATgtcactgataaggtaggagaaagagcgagggattgagcgtttgaa
acaaggcggtttctcgcacggcacacgctttgtggtgtcggtttccgatttcttggaagaagtag
gaaccggcattctTCCACCTGCACCCATCGAT
>PCR12_CF9_CONTIG_162_p8   284 pairs of NGS reads, 0.44%
AAGCATGAGGCTATAATGATgtcactgataaggtaggagaaagagcgagggattgagcgtttgaa
acaaggcggtttctcgcacggcacacgctttgtggtgtcggtttccgatttcttggaagaagtag
gaaccggcattctTCCACCTGCACCCATGCAT
>PCR12_CF9_CONTIG_162_p9   285 pairs of NGS reads, 0.44%
AAGCATGAGGCTATAATGATgtcactgataaggtaggagaaagagcgagggattgagcgtttgaa
acaaggcggtttctcgcacggcacacgctttgtggtgtcggtttccgatttcttggaagaagtag
gaaccggcattctTCCACCTGCACCCATAGCT
>PCR12_CF9_CONTIG_201_p10   445 pairs of NGS reads, 0.69%
AAGCATGAGGCTATAATGATGTCATTGATAAGGTAGGAGAAAGAGCGAGGGattgagcgtttgaa
acaatgcggcgggattgctttcttcagatctcccaccgagaaaggcggtttctcgcacggcacac
gctttgtggtgtCGGTTTCCGATTTCTTGGAAGAAGTAGGAACCGGCATTCTTCCACCTGCACCC
ATCGAT
>PCR12_CF9_CONTIG_201_p11   405 pairs of NGS reads, 0.63%
AAGCATGAGGCTATAATGATGTCATTGATAAGGTAGGAGAAAGAGCGAGGGATTGAgcgtttgaa
acaatgcggcgggattgctttcttcagatctcccaccgagaaaggcggtttctcgcacggcacac
gctttgtggtgtCGGTTTCCGATTTCTTGGAAGAAGTAGGAACCGGCATTCTTCCACCTGCACCC
ATAGCT
>PCR12_CF9_CONTIG_201_p12   225 pairs of NGS reads, 0.35%
AAGCATGAGGCTATAATGCTGTCACTGATAAGGTAGGAGAAAGAGCGAGGGattgagcgtttgaa
acaatgcggcgggattgctttcttcagatctcccaccgagaaaggcggtttctcgcacggcacac
gctttgtggtgtCGGTTTCCGATTTCTTGGAAGAAGTAGGAACCGGCATTCTTCCACCTGCACCC
ATCGAT
>PCR12_CF9_CONTIG_201_p13   294 pairs of NGS reads, 0.46%
AAGCATGAGGCTATAATGATGTCATTGATAAGGTAGGAGAAAGAGCGAGGGATTGAgcgtttgaa
acaatgcggcgggattgctttcttcagatctcccaccgagaaaggcggtttctcgcacggcacac
gctttgtggtgtCGGTTTCCGATTTCTTGGAAGAAGTAGGAACCGGCATTCTTCCACCTGCACCC
ATGCAT
>PCR12_CF9_CONTIG_201_p14   192 pairs of NGS reads, 0.3%
AAGCATGAGGCTATAATGCTGTCACTGATAAGGTAGGAGAAAGAGCGAGGGATTGAgcgtttgaa
acaatgcggcgggattgctttcttcagatctcccaccgagaaaggcggtttctcgcacggcacac
gctttgtggtgtCGGTTTCCGATTTCTTGGAAGAAGTAGGAACCGGCATTCTTCCACCTGCACCC
ATGCAT
>PCR12_CF9_CONTIG_201_p15   185 pairs of NGS reads, 0.29%
AAGCATGAGGCTATAATGCTGTCACTGATAAGGTAGGAGAAAGAGCGAGGGATTGAgcgtttgaa
acaatgcggcgggattgctttcttcagatctcccaccgagaaaggcggtttctcgcacggcacac

gctttgtggtgtCGGTTTCCGATTTCTTGGAAGAAGTAGGAACCGGCATTCTTCCACCTGCACCC
ATAGCT
>PCR12_CF9_CONTIG_192_p16   182 pairs of NGS reads, 0.28%
AAGCATGAGGCTATAATGATGTCCCCCAACATACCAAAACTAGCTGTCACtccaccggtagtagg
agatgtaagaattgatatatagaataacttttacttgattgataatcacataaaaccgaagaaa
ttttagccatttGCATCAAACTTAAACTTCCTTCTTGCATTCTTCCACCTGCACCCATAGCT
>PCR12_CF9_CONTIG_164_p17   117 pairs of NGS reads, 0.18%
AAGCATGAGGCTATAATGATGTcactgataaggtaggagaaagagcgagggattgagcgtttgaa
acataaggcggtttctcgcacggcacacgctttgtggtgtcggtttccgatttcttggaagaagt
aggaaccggcatTCTTCCACCTGCACCCATGCAT
>PCR12_CF9_CONTIG_164_p18   117 pairs of NGS reads, 0.18%
AAGCATGAGGCTATAATGATGTcactgataaggtaggagaaagagcgagggattgagcgtttgaa
acataaggcggtttctcgcacggcacacgctttgtggtgtcggtttccgatttcttggaagaagt
aggaaccggcatTCTTCCACCTGCACCCATAGCT
>PCR12_CF9_CONTIG_164_p19   106 pairs of NGS reads, 0.16%
AAGCATGAGGCTATAATGATGTcactgataaggtaggagaaagagcgagggattgagcgtttgaa
acataaggcggtttctcgcacggcacacgctttgtggtgtcggtttccgatttcttggaagaagt
aggaaccggcatTCTTCCACCTGCACCCATCGAT
>PCR12_CF9_CONTIG_202_p20   90 pairs of NGS reads, 0.14%
AAGCATGAGGCTATAATGATGTCACTGATAAGGTAGGAGAAAGAGCGAGGGATTGAGCGTttgaa
acaatgcggcgggattgctttcttcagatctcccaccgagaaaaggcggtttctcgcacggcaca
cgctttgtggtgTCGGTTTCCGATTTCTTGGAAGAAGTAGGAACCGGCATTCTTCCACCTGCACC
CATCGAT
>PCR12_CF9_CONTIG_202_p21   76 pairs of NGS reads, 0.11%
AAGCATGAGGCTATAATGATGTCACTGATAAGGTAGGAGAAAGAGCGAGGGATTGAGCGTttgaa
acaaatgcggcgggattgctttcttcagatctcccaccgagaaaggcggtttctcgcacggcaca
cgctttgtggtgTCGGTTTCCGATTTCTTGGAAGAAGTAGGAACCGGCATTCTTCCACCTGCACC
CATCGAT
>PCR12_CF9_CONTIG_202_p22   74 pairs of NGS reads, 0.11%
AAGCATGAGGCTATAATGATGTCACTGATAAGGTAGGAGAAAGAGCGAGGGATTGAGCGTttgaa
acaatgcggcgggattgctttcttcagatctcccaccgagataaggcggtttctcgcacggcaca
cgctttgtggtgtCGGTTTCCGATTTCTTGGAAGAAGTAGGAACCGGCATTCTTCCACCTGCACC
CATCGAT
>PCR12_CF9_CONTIG_192_p23   71 pairs of NGS reads, 0.11%
AAGCATGAGGCTATAATGATGTCCCCCAACATACCAAAACTAGCTGTCACtccaccggtagtagg
agatgtaagaattgatatatagaataacttttacttgattgataatcacataaaaccgaagaaa
ttttagccatttGCATCAAACTTAAACTTCCTTCTTGCATTCTTCCACCTGCACCCATCGAT
>PCR12_CF9_CONTIG_202_p24   69 pairs of NGS reads, 0.1%
AAGCATGAGGCTATAATGATGTCACTGATAAGGTAGGAGAAAGAGCGAGGGATTGAGCGTttgaa
acaatgcggcgggattgctttcttcagatctcccaccgagaaaaggcggtttctcgcacggcaca
cgctttgtggtgTCGGTTTCCGATTTCTTGGAAGAAGTAGGAACCGGCATTCTTCCACCTGCACC
CATGCAT
>PCR12_CF9_CONTIG_202_p25   67 pairs of NGS reads, 0.1%
AAGCATGAGGCTATAATGATGTCACTGATAAGGTAGGAGAAAGAGCGAGGGATTGAGCGTttgaa
acaatgcggcgggattgctttcttcagatctcccaccgagataaggcggtttctcgcacggcaca
cgctttgtggtgtCGGTTTCCGATTTCTTGGAAGAAGTAGGAACCGGCATTCTTCCACCTGCACC
CATAGCT
>PCR12_CF9_CONTIG_200_p26   66 pairs of NGS reads, 0.1%
AAGCATGAGGCTATAATGATGTCACTGATAAGGTAGGAGAAAGAGCGAGGGATTGAGCgtttgaa
acaatgcggcgggattgctttcttcagatctcccaccgagaaggcggtttctcgcacggcacacg
ctttgtggtgtcGGTTTCCGATTTCTTGGAAGAAGTAGGAACCGGCATTCTTCCACCTGCACCCA
TAGCT

>PCR12_CF9_CONTIG_202_p27   64 pairs of NGS reads, 0.1%
AAGCATGAGGCTATAATGATGTCACTGATAAGGTAGGAGAAAGAGCGAGGGATTGAGCGTttgaa
acaatgcggcgggattgctttcttcagatctcccaccgagaaaaggcggtttctcgcacggcaca
cgctttgtggtgTCGGTTTCCGATTTCTTGGAAGAAGTAGGAACCGGCATTCTTCCACCTGCACC
CATAGCT
>PCR12_CF9_CONTIG_202_p28   61 pairs of NGS reads, 0.09%
AAGCATGAGGCTATAATGATGTCACTGATAAGGTAGGAGAAAGAGCGAGGGATTGAGCGTttgaa
acaatgcggcgggattgctttcttcagatctcccaccgagataaggcggtttctcgcacggcaca
cgctttgtggtgtCGGTTTCCGATTTCTTGGAAGAAGTAGGAACCGGCATTCTTCCACCTGCACC
CATGCAT
>PCR12_CF9_CONTIG_202_p29   58 pairs of NGS reads, 0.09%
AAGCATGAGGCTATAATGATGTCACTGATAAGGTAGGAGAAAGAGCGAGGGATTGAGCGTttgaa
acaaatgcggcgggattgctttcttcagatctcccaccgagaaaggcggtttctcgcacggcaca
cgctttgtggtgTCGGTTTCCGATTTCTTGGAAGAAGTAGGAACCGGCATTCTTCCACCTGCACC
CATAGCT
>PCR12_CF9_CONTIG_202_p30   49 pairs of NGS reads, 0.07%
AAGCATGAGGCTATAATGATGTCACTGATAAGGTAGGAGAAAGAGCGAGGGATTGAGCGTttgaa
acaaatgcggcgggattgctttcttcagatctcccaccgagaaaggcggtttctcgcacggcaca
cgctttgtggtgTCGGTTTCCGATTTCTTGGAAGAAGTAGGAACCGGCATTCTTCCACCTGCACC
CATGCAT
>PCR12_CF9_CONTIG_203_p31   49 pairs of NGS reads, 0.07%
AAGCATGAGGCTATAATGATGTCATCCTTGTTGGATCTGCAACCAACTTTGTTAGTCTGCTttca
tatctattatcttaacttgtatggccgctgaatttccctttggtttgcagcaagttcctgatgag
attgaaagagtgTTAGTTGGGATACAAGCTTATTTGAGCATCCGAAAGAATTCTTCCACCTGCAC
CCATGCAT
>PCR12_CF9_CONTIG_200_p32   48 pairs of NGS reads, 0.07%
AAGCATGAGGCTATAATGATGTCACTGATAAGGTAGGAGAAAGAGCGAGGGATTGAGCgtttgaa
acaatgcggcgggattgctttcttcagatctcccaccgagaaggcggtttctcgcacggcacacg
ctttgtggtgtcGGTTTCCGATTTCTTGGAAGAAGTAGGAACCGGCATTCTTCCACCTGCACCCA
TGCAT
>PCR12_CF9_CONTIG_200_p33   41 pairs of NGS reads, 0.06%
AAGCATGAGGCTATAATGATGTCACTGATAAGGTAGGAGAAAGAGCGAGGGATTGAGCgtttgaa
acaatgcggcgggattgctttcttcagatctcccaccgagaaggcggtttctcgcacggcacacg
ctttgtggtgtcGGTTTCCGATTTCTTGGAAGAAGTAGGAACCGGCATTCTTCCACCTGCACCCA
TCGAT
>PCR12_CF9_CONTIG_188_p34   41 pairs of NGS reads, 0.06%
AAGCATGAGGCTATAATGATGTGTTTCGTGTTATATTAATGATTTTctaaatagattaaattgtg
ttgaattgggcctaattggattgaataaaggcctgaaaaatcctataaaccacacatcacggcgt
gcgacgtgagtgTTGCGGTCAATGAACGACACGCATTCTTCCACCTGCACCCATGCAT
>PCR12_CF9_CONTIG_201_p35   39 pairs of NGS reads, 0.06%
AAGCATGAGGCTATAATGGTGTCACTGATAAGGTAGGAGAAAGAGCGAGGGattgagcgtttgaa
acaatgcggcgggattgctttcttcagatctcccaccgagaaaggcggtttctcgcacggcacac
gctttgtggtgtCGGTTTCCGATTTCTTGGAAGAAGTAGGAACCGGCATTCTTCCACCTGCACCC
ATCGAT
>PCR12_CF9_CONTIG_203_p36   38 pairs of NGS reads, 0.05%
AAGCATGAGGCTATAATGATGTCACTGATAAGGTAGGAGAAAGAGCGAGGGATTGAGCGTTtgaa
acaaatgcggcgggattgctttcttcagatctcccaccgagataaggcggtttctcgcacggcac
acgctttgtggtGTCGGTTTCCGATTTCTTGGAAGAAGTAGGAACCGGCATTCTTCCACCTGCAC
CCATAGCT
>PCR12_CF9_CONTIG_161_p37   37 pairs of NGS reads, 0.05%
AAGCATGAGGCTATAATGAtgtcactgataaggtaggagaaagagcgagggattgagcgtttgaa
acaggcggtttctcgcacggcacacgctttgtggtgtcggtttccgatttcttggaagaagtagg

aaccggcattctTCCACCTGCACCCATCGAT
>PCR12_CF9_CONTIG_201_p38   36 pairs of NGS reads, 0.05%
AAGCATGAGGCTATAATGGTGTCACTGATAAGGTAGGAGAAAGAGCGAGGGATTGAgcgtttgaa
acaatgcggcgggattgctttcttcagatctcccaccgagaaaggcggtttctcgcacggcacac
gctttgtggtgtCGGTTTCCGATTTCTTGGAAGAAGTAGGAACCGGCATTCTTCCACCTGCACCC
ATAGCT
>PCR12_CF9_CONTIG_161_p39   33 pairs of NGS reads, 0.05%
AAGCATGAGGCTATAATGAtgtcactgataaggtaggagaaagagcgagggattgagcgtttgaa
acaggcggtttctcgcacggcacacgctttgtggtgtcggtttccgatttcttggaagaagtagg
aaccggcattctTCCACCTGCACCCATGCAT
>PCR12_CF9_CONTIG_164_p40   33 pairs of NGS reads, 0.05%
AAGCATGAGGCTATAATGATGTcactgataaggtaggagaaagagcgagggattgagcgtttgaa
acaaaaggcggtttctcgcacggcacacgctttgtggtgtcggtttccgatttcttggaagaagt
aggaaccggcatTCTTCCACCTGCACCCATCGAT
>PCR12_CF9_CONTIG_202_p41   32 pairs of NGS reads, 0.05%
AAGCATGAGGCTATAATGATGTCACTGATAAGGTAGGAGAAAGAGCGAGGGATTGAGCGTttgaa
acatctcggtgggagatctgaagaaagcaatcccgccgcattaaggcggtttctcgcacggcaca
cgctttgtggtgTCGGTTTCCGATTTCTTGGAAGAAGTAGGAACCGGCATTCTTCCACCTGCACC
CATCGAT
>PCR12_CF9_CONTIG_188_p42   31 pairs of NGS reads, 0.04%
AAGCATGAGGCTATAATGATGTGTTTCGTGTTATATTAATGATTTTctaaatagattaaattgtg
ttgaattgggcctaattggattgaataaaggcctgaaaaatcctataaaccacacatcacggcgt
gcgacgtgagtgTTGCGGTCAATGAACGACACGCATTCTTCCACCTGCACCCATCGAT
>PCR12_CF9_CONTIG_164_p43   30 pairs of NGS reads, 0.04%
AAGCATGAGGCTATAATGATGTcactgataaggtaggagaaagagcgagggattgagcgtttgaa
acaaaaggcggtttctcgcacggcacacgctttgtggtgtcggtttccgatttcttggaagaagt
aggaaccggcatTCTTCCACCTGCACCCATAGCT
>PCR12_CF9_CONTIG_161_p44   30 pairs of NGS reads, 0.04%
AAGCATGAGGCTATAATGAtgtcactgataaggtaggagaaagagcgagggattgagcgtttgaa
acaggcggtttctcgcacggcacacgctttgtggtgtcggtttccgatttcttggaagaagtagg
aaccggcattctTCCACCTGCACCCATAGCT
>PCR12_CF9_CONTIG_201_p45   30 pairs of NGS reads, 0.04%
AAAGCATGAGGCTATAATGATGTCACTGATAAGGTAGGAGAAAGAGCGAGGGATTGAGCgtttga
aacaatgcggcgggattgctttcttcagatctcccaccgagaaaggcggtttctcgcacggcaca
cgctttgtggtgtcggtTTCCGATTTCTTGGAAGAAGTAGGAACCGGCATTCTTCCACCTGCACC
CATACT
>PCR12_CF9_CONTIG_201_p46   30 pairs of NGS reads, 0.04%
AAGCATGAGGCTATAATGGTGTCACTGATAAGGTAGGAGAAAGAGCGAGGGATTGAgcgtttgaa
acaatgcggcgggattgctttcttcagatctcccaccgagaaaggcggtttctcgcacggcacac
gctttgtggtgtCGGTTTCCGATTTCTTGGAAGAAGTAGGAACCGGCATTCTTCCACCTGCACCC
ATGCAT
>PCR12_CF9_CONTIG_164_p47   28 pairs of NGS reads, 0.04%
AAGCATGAGGCTATAATGATGTcactgataaggtaggagaaagagcgagggattgagcgtttgaa
acaaaaggcggtttctcgcacggcacacgctttgtggtgtcggtttccgatttcttggaagaagt
aggaaccggcatTCTTCCACCTGCACCCATGCAT
>PCR12_CF9_CONTIG_203_p48   27 pairs of NGS reads, 0.04%
AAGCATGAGGCTATAATGATGTCACTGATAAGGTAGGAGAAAGAGCGAGGGATTGAGCGTTtgaa
acaaatgcggcgggattgctttcttcagatctcccaccgagaaaggcggtttctcgcacggcac
acgctttgtggtGTCGGTTTCCGATTTCTTGGAAGAAGTAGGAACCGGCATTCTTCCACCTGCAC
CCATGCAT
>PCR12_CF9_CONTIG_202_p49   26 pairs of NGS reads, 0.04%
AAGCATGAGGCTATAATGATGTCACTGATAAGGTAGGAGAAAGAGCGAGGGATTGAGCGTttgaa

acatctcggtgggagatctgaagaaagcaatcccgccgcattaaggcggtttctcgcacggcaca
cgctttgtggtgTCGGTTTCCGATTTCTTGGAAGAAGTAGGAACCGGCATTCTTCCACCTGCACC
CATAGCT
>PCR12_CF9_CONTIG_201_p50   23 pairs of NGS reads, 0.03%
AAAGCATGAGGCTATAATGATGTCACTGATAAGGTAGGAGAAAGAGCGAGGGATTGAGCgtttga
aacaatgcggcgggattgctttcttcagatctcccaccgagaaaggcggtttctcgcacggcaca
cgctttgtggtgtcggtTTCCGATTTCTTGGAAGAAGTAGGAACCGGCATTCTTCCACCTGCACC
CATCAT
>PCR12_CF9_CONTIG_173_p51   22 pairs of NGS reads, 0.03%
AAGCATGAGGCTATAATGATGTCTGCGGGCCatatctccggtggccacctcaaccccgccgtcac
cctcggtctactcttgggtggccacatcagcgtattccgtgcattcctttattggattgatcaat
tgttggcctcctCCGCAGCATTCTTCCACCTGCACCCATGCAT
>PCR12_CF9_CONTIG_202_p52   21 pairs of NGS reads, 0.03%
AAGCATGAGGCTATAATGATGTCACTGATAAGGTAGGAGAAAGAGCGAGGGATTGAGCGTttgaa
acatctcggtgggagatctgaagaaagcaatcccgccgcattaaggcggtttctcgcacggcaca
cgctttgtggtgTCGGTTTCCGATTTCTTGGAAGAAGTAGGAACCGGCATTCTTCCACCTGCACC
CATGCAT
>PCR12_CF9_CONTIG_199_p53   20 pairs of NGS reads, 0.03%
AAGCATGAGGCTATAATGATGTCACTGATAAGGTAGGAGAAAGAGCGAGGGATTGAGcgtttgaa
acaatgcggcgggattgctttcttcagatctcccaccgagaggcggtttctcgcacggcacacgc
tttgtggtgtcgGTTTCCGATTTCTTGGAAGAAGTAGGAACCGGCATTCTTCCACCTGCACCCAT
CGAT
>PCR12_CF9_CONTIG_201_p54   20 pairs of NGS reads, 0.03%
AAGCATGAGGCTATAATGATGTCACTGATAAGGTAGGAGAAAGAGCGAGGGATTGAGCGtttgaa
acaatgcggcgggattgctttcttcagatctcccaccgagaaaggcggtttctcgaacggcacac
gctttgtggtgtCGGTTTCCGATTTCTTGGAAGAAGTAGGAACCGGCATTCTTCCACCTGCACCC
ATCGAT
>PCR12_CF9_CONTIG_188_p55   20 pairs of NGS reads, 0.03%
AAGCATGAGGCTATAATGATGTGTTTCGTGTTATATTAATGATTTTctaaatagattaaattgtg
ttgaattgggcctaattggattgaataaaggcctgaaaaatcctataaaccacacatcacggcgt
gcgacgtgagtgTTGCGGTCAATGAACGACACGCATTCTTCCACCTGCACCCATAGCT
>PCR12_CF9_CONTIG_148_p56   18 pairs of NGS reads, 0.02%
AAGCATgaggctataatgatgtcactgataaggtaggagaaagagcgagggattgagcggtttct
cgcacggcacacgctttgtggtgtcggtttccgatttcttggaagaagtaggaaccggcattctt
ccacctgcacccATGCAT
>PCR12_CF9_CONTIG_203_p57   18 pairs of NGS reads, 0.02%
AAGCATGAGGCTATAATGATGTCACTGATAAGGTAGGAGAAAGAGCGAGGGATTGAGCGTTtgaa
acaaatgcggcgggattgctttcttcagatctcccaccgagaaaaggcggtttctcgcacggcac
acgctttgtggtGTCGGTTTCCGATTTCTTGGAAGAAGTAGGAACCGGCATTCTTCCACCTGCAC
CCATCGAT
>PCR12_CF9_CONTIG_203_p58   17 pairs of NGS reads, 0.02%
AAGCATGAGGCTATAATGATGTCATCCTTGTTGGATCTGCAACCAACTTTGTTAGTCTGCTttca
tatctattatcttaacttgtatggccgctgaatttccctttggtttgcagcaagttcctgatgag
attgaaagagtgTTAGTTGGGATACAAGCTTATTTGAGCATCCGAAAGAATTCTTCCACCTGCAC
CCATCGAT
>PCR12_CF9_CONTIG_203_p59   16 pairs of NGS reads, 0.02%
AAGCATGAGGCTATAATGATGTCACTGATAAGGTAGGAGAAAGAGCGAGGGATTGAGCGTTtgaa
acaaatgcggcgggattgctttcttcagatctcccaccgagaaaaggcggtttctcgcacggcac
acgctttgtggtGTCGGTTTCCGATTTCTTGGAAGAAGTAGGAACCGGCATTCTTCCACCTGCAC
CCATAGCT
>PCR12_CF9_CONTIG_203_p60   16 pairs of NGS reads, 0.02%
AAGCATGAGGCTATAATGATGTCACTGATAAGGTAGGAGAAAGAGCGAGGGATTGAGCGTTtgaa

acaaatgcggcgggattgctttcttcagatctcccaccgagataaggcggtttctcgcacggcac
acgctttgtggtGTCGGTTTCCGATTTCTTGGAAGAAGTAGGAACCGGCATTCTTCCACCTGCAC
CCATCGAT
>PCR12_CF9_CONTIG_202_p61   15 pairs of NGS reads, 0.02%
AAGCATGAGGCTATAATGATGTCACTGATAAGGTAGGAGAAAGAGCGAGGGATTGAGCGTttgaa
acaatgcggcgggattgctttcttcagatctcccaccgagagaaggcggtttctcgcacggcaca
cgctttgtggtgTCGGTTTCCGATTTCTTGGAAGAAGTAGGAACCGGCATTCTTCCACCTGCACC
CATAGCT
>PCR12_CF9_CONTIG_201_p62   15 pairs of NGS reads, 0.02%
AAGCATGAGGCTATAATGATGTCACTGATAAGGTAGGAGAAAGAGCGAGGGATTGAGCGtttgaa
acaatgcggcgggattgctttcttcagatctcccaccgagaaaggcggtttctcgaacggcacac
gctttgtggtgtCGGTTTCCGATTTCTTGGAAGAAGTAGGAACCGGCATTCTTCCACCTGCACCC
ATGCAT

AtFAD RNP 1

>DPCR1_CH3_CONTIG_163_p1   7974 pairs of NGS reads, 17.43%
AAGCATGAGGCTATAatgatgtcactgataaggtaggagaaagagcgagggattgagcgtttgaa
acaaaggcggtttctcgcacggcacacgctttgtggtgtcggtttccgatttcttggaagaagta
ggaaccggcattctTCCACCTGCACCCATAGCT
>DPCR1_CH3_CONTIG_201_p2   6298 pairs of NGS reads, 13.77%
AAGCATGAGGCTATAATGATGTCACTGATAAGGTAGGAGAAAGAGCGAGGGAttgagcgtttgaa
acaatgcggcgggattgctttcttcagatctcccaccgagaaaggcggtttctcgcacggcacac
gctttgtggtgtcggTTTCCGATTTCTTGGAAGAAGTAGGAACCGGCATTCTTCCACCTGCACCC
ATAGCT
>DPCR1_CH3_CONTIG_163_p3   5991 pairs of NGS reads, 13.1%
AAGCATGAGGCTATAATGatgtcactgataaggtaggagaaagagcgagggattgagcgtttgaa
acaaaggcggtttctcgcacggcacacgctttgtggtgtcggtttccgatttcttggaagaagta
ggaaccggcattctTCCACCTGCACCCATCGAT
>DPCR1_CH3_CONTIG_163_p4   5575 pairs of NGS reads, 12.19%
AAGCATGAGGCTATAATGatgtcactgataaggtaggagaaagagcgagggattgagcgtttgaa
acaaaggcggtttctcgcacggcacacgctttgtggtgtcggtttccgatttcttggaagaagta
ggaaccggcattctTCCACCTGCACCCATGCAT
>DPCR1_CH3_CONTIG_201_p5   4739 pairs of NGS reads, 10.36%
AAGCATGAGGCTATAATGATGTCACTGATAAGGTAGGAGAAAGAGCGAGGGATTGAgcgtttgaa
acaatgcggcgggattgctttcttcagatctcccaccgagaaaggcggtttctcgcacggcacac
gctttgtggtgtcggTTTCCGATTTCTTGGAAGAAGTAGGAACCGGCATTCTTCCACCTGCACCC
ATGCAT
>DPCR1_CH3_CONTIG_201_p6   4613 pairs of NGS reads, 10.08%
AAGCATGAGGCTATAATGATGTCACTGATAAGGTAGGAGAAAGAGCGAGGGATTGAgcgtttgaa
acaatgcggcgggattgctttcttcagatctcccaccgagaaaggcggtttctcgcacggcacac
gctttgtggtgtcggTTTCCGATTTCTTGGAAGAAGTAGGAACCGGCATTCTTCCACCTGCACCC
ATCGAT
>DPCR1_CH3_CONTIG_162_p7   1721 pairs of NGS reads, 3.76%
AAGCATGAGGCTATAAtgatgtcactgataaggtaggagaaagagcgagggattgagcgtttgaa
acaaggcggtttctcgcacggcacacgctttgtggtgtcggtttccgatttcttggaagaagtag
gaaccggcattcttCCACCTGCACCCATAGCT
>DPCR1_CH3_CONTIG_162_p8   1279 pairs of NGS reads, 2.79%
AAGCATGAGGCTATAATGatgtcactgataaggtaggagaaagagcgagggattgagcgtttgaa
acaaggcggtttctcgcacggcacacgctttgtggtgtcggtttccgatttcttggaagaagtag
gaaccggcattcttCCACCTGCACCCATCGAT
>DPCR1_CH3_CONTIG_162_p9   1183 pairs of NGS reads, 2.58%

AAGCATGAGGCTATAATGatgtcactgataaggtaggagaaagagcgagggattgagcgtttgaa
acaaggcggtttctcgcacggcacacgctttgtggtgtcggtttccgatttcttggaagaagtag
gaaccggcattcttCCACCTGCACCCATGCAT
>DPCR1_CH3_CONTIG_200_p10   383 pairs of NGS reads, 0.83%
AAGCATGAGGCTATAATGATGTCACTGATAAGGTAGGAGAAAGAGCGAGGGATTGAGCgtttgaa
acaatgcggcgggattgctttcttcagatctcccaccgagaaggcggtttctcgcacggcacacg
ctttgtggtgtcgGTTTCCGATTTCTTGGAAGAAGTAGGAACCGGCATTCTTCCACCTGCACCCA
TAGCT
>DPCR1_CH3_CONTIG_200_p11   334 pairs of NGS reads, 0.73%
AAGCATGAGGCTATAATGATGTCACTGATAAGGTAGGAGAAAGAGCGAGGGATTGAGcgtttgaa
acaatgcggcgggattgctttcttcagatctcccaccgagaaggcggtttctcgcacggcacacg
ctttgtggtgtcgGTTTCCGATTTCTTGGAAGAAGTAGGAACCGGCATTCTTCCACCTGCACCCA
TGCAT
>DPCR1_CH3_CONTIG_202_p12   303 pairs of NGS reads, 0.66%
AAGCATGAGGCTATAATGATGTCACTGATAAGGTAGGAGAAAGAGCGAGGGATTGAGCGTttgaa
acaatgcggcgggattgctttcttcagatctcccaccgagaaaaggcggtttctcgcacggcaca
cgctttgtggtgtCGGTTTCCGATTTCTTGGAAGAAGTAGGAACCGGCATTCTTCCACCTGCACC
CATAGCT
>DPCR1_CH3_CONTIG_200_p13   288 pairs of NGS reads, 0.62%
AAGCATGAGGCTATAATGATGTCACTGATAAGGTAGGAGAAAGAGCGAGGGATTGAGCgtttgaa
acaatgcggcgggattgctttcttcagatctcccaccgagaaggcggtttctcgcacggcacacg
ctttgtggtgtcgGTTTCCGATTTCTTGGAAGAAGTAGGAACCGGCATTCTTCCACCTGCACCCA
TCGAT
>DPCR1_CH3_CONTIG_202_p14   273 pairs of NGS reads, 0.59%
AAGCATGAGGCTATAATGATGTCACTGATAAGGTAGGAGAAAGAGCGAGGGATTGAGCGTttgaa
acaatgcggcgggattgctttcttcagatctcccaccgagataaggcggtttctcgcacggcaca
cgctttgtggtgtCGGTTTCCGATTTCTTGGAAGAAGTAGGAACCGGCATTCTTCCACCTGCACC
CATAGCT
>DPCR1_CH3_CONTIG_164_p15   226 pairs of NGS reads, 0.49%
AAGCATGAGGCTATAATGATGTcactgataaggtaggagaaagagcgagggattgagcgtttgaa
acataaggcggtttctcgcacggcacacgctttgtggtgtcggtttccgatttcttggaagaagt
aggaaccggcattCTTCCACCTGCACCCATAGCT
>DPCR1_CH3_CONTIG_202_p16   222 pairs of NGS reads, 0.48%
AAGCATGAGGCTATAATGATGTCACTGATAAGGTAGGAGAAAGAGCGAGGGATTGAGCGTttgaa
acaatgcggcgggattgctttcttcagatctcccaccgagaaaaggcggtttctcgcacggcaca
cgctttgtggtgtCGGTTTCCGATTTCTTGGAAGAAGTAGGAACCGGCATTCTTCCACCTGCACC
CATGCAT
>DPCR1_CH3_CONTIG_202_p17   215 pairs of NGS reads, 0.47%
AAGCATGAGGCTATAATGATGTCACTGATAAGGTAGGAGAAAGAGCGAGGGATTGAGCGTttgaa
acaatgcggcgggattgctttcttcagatctcccaccgagaaaaggcggtttctcgcacggcaca
cgctttgtggtgtCGGTTTCCGATTTCTTGGAAGAAGTAGGAACCGGCATTCTTCCACCTGCACC
CATCGAT
>DPCR1_CH3_CONTIG_161_p18   211 pairs of NGS reads, 0.46%
AAGCATGAGGCTATAATGAtgtcactgataaggtaggagaaagagcgagggattgagcgtttgaa
acaggcggtttctcgcacggcacacgctttgtggtgtcggtttccgatttcttggaagaagtagg
aaccggcattcttCCACCTGCACCCATAGCT
>DPCR1_CH3_CONTIG_164_p19   207 pairs of NGS reads, 0.45%
AAGCATGAGGCTATAATGATGTcactgataaggtaggagaaagagcgagggattgagcgtttgaa
acataaggcggtttctcgcacggcacacgctttgtggtgtcggtttccgatttcttggaagaagt
aggaaccggcattCTTCCACCTGCACCCATGCAT
>DPCR1_CH3_CONTIG_161_p20   184 pairs of NGS reads, 0.4%
AAGCATGAGGCTATAATGAtgtcactgataaggtaggagaaagagcgagggattgagcgtttgaa

acaggcggtttctcgcacggcacacgctttgtggtgtcggtttccgatttcttggaagaagtagg
aaccggcattcttCCACCTGCACCCATCGAT
>DPCR1_CH3_CONTIG_164_p21 177 pairs of NGS reads, 0.38%
AAGCATGAGGCTATAATGATGTcactgataaggtaggagaaagagcgagggattgagcgtttgaa
acataaggcggtttctcgcacggcacacgctttgtggtgtcggtttccgatttcttggaagaagt
aggaaccggcattCTTCCACCTGCACCCATCGAT
>DPCR1_CH3_CONTIG_161_p22 153 pairs of NGS reads, 0.33%
AAGCATGAGGCTATAATGAtgtcactgataaggtaggagaaagagcgagggattgagcgtttgaa
acaggcggtttctcgcacggcacacgctttgtggtgtcggtttccgatttcttggaagaagtagg
aaccggcattcttCCACCTGCACCCATGCAT
>DPCR1_CH3_CONTIG_202_p23 152 pairs of NGS reads, 0.33%
AAGCATGAGGCTATAATGATGTCACTGATAAGGTAGGAGAAAGAGCGAGGGATTGAGCGTttgaa
acaatgcggcgggattgctttcttcagatctcccaccgagataaggcggtttctcgcacggcaca
cgctttgtggtgtCGGTTTCCGATTTCTTGGAAGAAGTAGGAACCGGCATTCTTCCACCTGCACC
CATGCAT
>DPCR1_CH3_CONTIG_202_p24 137 pairs of NGS reads, 0.29%
AAGCATGAGGCTATAATGATGTCACTGATAAGGTAGGAGAAAGAGCGAGGGATTGAGCGTttgaa
acaatgcggcgggattgctttcttcagatctcccaccgagataaggcggtttctcgcacggcaca
cgctttgtggtgtCGGTTTCCGATTTCTTGGAAGAAGTAGGAACCGGCATTCTTCCACCTGCACC
CATCGAT
>DPCR1_CH3_CONTIG_202_p25 125 pairs of NGS reads, 0.27%
AAGCATGAGGCTATAATGATGTCACTGATAAGGTAGGAGAAAGAGCGAGGGATTGAGCGTttgaa
acaaatgcggcgggattgctttcttcagatctcccaccgagaaaggcggtttctcgcacggcaca
cgctttgtggtgTCGGTTTCCGATTTCTTGGAAGAAGTAGGAACCGGCATTCTTCCACCTGCACC
CATAGCT
>DPCR1_CH3_CONTIG_202_p26 112 pairs of NGS reads, 0.24%
AAGCATGAGGCTATAATGATGTCACTGATAAGGTAGGAGAAAGAGCGAGGGATTGAGCGTttgaa
acaaatgcggcgggattgctttcttcagatctcccaccgagaaaggcggtttctcgcacggcaca
cgctttgtggtgTCGGTTTCCGATTTCTTGGAAGAAGTAGGAACCGGCATTCTTCCACCTGCACC
CATGCAT
>DPCR1_CH3_CONTIG_164_p27 86 pairs of NGS reads, 0.18%
AAGCATGAGGCTATAATGATGTcactgataaggtaggagaaagagcgagggattgagcgtttgaa
acaaaaggcggtttctcgcacggcacacgctttgtggtgtcggtttccgatttcttggaagaagt
aggaaccggcatTCTTCCACCTGCACCCATAGCT
>DPCR1_CH3_CONTIG_202_p28 80 pairs of NGS reads, 0.17%
AAGCATGAGGCTATAATGATGTCACTGATAAGGTAGGAGAAAGAGCGAGGGATTGAGCGTttgaa
acaaatgcggcgggattgctttcttcagatctcccaccgagaaaggcggtttctcgcacggcaca
cgctttgtggtgTCGGTTTCCGATTTCTTGGAAGAAGTAGGAACCGGCATTCTTCCACCTGCACC
CATCGAT
>DPCR1_CH3_CONTIG_164_p29 75 pairs of NGS reads, 0.16%
AAGCATGAGGCTATAATGATGTcactgataaggtaggagaaagagcgagggattgagcgtttgaa
acaaaaggcggtttctcgcacggcacacgctttgtggtgtcggtttccgatttcttggaagaagt
aggaaccggcatTCTTCCACCTGCACCCATGCAT
>DPCR1_CH3_CONTIG_164_p30 74 pairs of NGS reads, 0.16%
AAGCATGAGGCTATAATGATGTcactgataaggtaggagaaagagcgagggattgagcgtttgaa
acaaaaggcggtttctcgcacggcacacgctttgtggtgtcggtttccgatttcttggaagaagt
aggaaccggcatTCTTCCACCTGCACCCATCGAT
>DPCR1_CH3_CONTIG_202_p31 73 pairs of NGS reads, 0.15%
AAGCATGAGGCTATAATGATGTCACTGATAAGGTAGGAGAAAGAGCGAGGGATTGAGCGTttgaa
acatctcggtgggagatctgaagaaagcaatcccgccgcattaaggcggtttctcgcacggcaca
cgctttgtggtgTCGGTTTCCGATTTCTTGGAAGAAGTAGGAACCGGCATTCTTCCACCTGCACC
CATAGCT

>DPCR1_CH3_CONTIG_192_p32   71 pairs of NGS reads, 0.15%
AAGCATGAGGCTATAATGATGTCCCCCAACATACCAAAACTAGCTGTCACtccaccggtagtagg
agatgtaagaattgatatatagaataacttttacttgattgataatcacataaaaccgaagaaa
ttttagccatttGCATCAAACTTAAACTTCCTTCTTGCATTCTTCCACCTGCACCCATAGCT
>DPCR1_CH3_CONTIG_199_p33   70 pairs of NGS reads, 0.15%
AAGCATGAGGCTATAATGATGTCACTGATAAGGTAGGAGAAAGAGCGAGGGATTGAGcgtttgaa
acaatgcggcgggattgctttcttcagatctcccaccgagaggcggtttctcgcacggcacacgc
tttgtggtgtcgGTTTCCGATTTCTTGGAAGAAGTAGGAACCGGCATTCTTCCACCTGCACCCAT
AGCT
>DPCR1_CH3_CONTIG_203_p34   58 pairs of NGS reads, 0.12%
AAGCATGAGGCTATAATGATGTCACTGATAAGGTAGGAGAAAGAGCGAGGGATTGAGCGTTtgaa
acaaatgcggcgggattgctttcttcagatctcccaccgagaaaaggcggtttctcgcacggcac
acgctttgtggtGTCGGTTTCCGATTTCTTGGAAGAAGTAGGAACCGGCATTCTTCCACCTGCAC
CCATAGCT
>DPCR1_CH3_CONTIG_201_p35   53 pairs of NGS reads, 0.11%
AAGCATGAGGCTATAATGATGTCACTGATAAGGTAGGAGAAAGAGCGAGGGATTGAGCGtttgaa
acaaatgcggcgggattgctttcttcagatctcccaccgagaaggcggtttctcgcacggcacac
gctttgtggtgtCGGTTTCCGATTTCTTGGAAGAAGTAGGAACCGGCATTCTTCCACCTGCACCC
ATAGCT
>DPCR1_CH3_CONTIG_203_p36   51 pairs of NGS reads, 0.11%
AAGCATGAGGCTATAATGATGTCACTGATAAGGTAGGAGAAAGAGCGAGGGATTGAGCGTTtgaa
acaaatgcggcgggattgctttcttcagatctcccaccgagataaggcggtttctcgcacggcac
acgctttgtggtGTCGGTTTCCGATTTCTTGGAAGAAGTAGGAACCGGCATTCTTCCACCTGCAC
CCATAGCT
>DPCR1_CH3_CONTIG_199_p37   48 pairs of NGS reads, 0.1%
AAGCATGAGGCTATAATGATGTCACTGATAAGGTAGGAGAAAGAGCGAGGGATTGAGcgtttgaa
acaatgcggcgggattgctttcttcagatctcccaccgagaggcggtttctcgcacggcacacgc
tttgtggtgtcgGTTTCCGATTTCTTGGAAGAAGTAGGAACCGGCATTCTTCCACCTGCACCCAT
GCAT
>DPCR1_CH3_CONTIG_202_p38   48 pairs of NGS reads, 0.1%
AAGCATGAGGCTATAATGATGTCACTGATAAGGTAGGAGAAAGAGCGAGGGATTGAGCGTttgaa
acatctcggtgggagatctgaagaaagcaatcccgccgcattaaggcggtttctcgcacggcaca
cgctttgtggtgTCGGTTTCCGATTTCTTGGAAGAAGTAGGAACCGGCATTCTTCCACCTGCACC
CATGCAT
>DPCR1_CH3_CONTIG_159_p39   42 pairs of NGS reads, 0.09%
AAGCATGAGGCTATAATgatgtcactgataaggtaggagaaagagcgagggattgagcgtttgaa
aggcggtttctcgcacggcacacgctttgtggtgtcggtttccgatttcttggaagaagtaggaa
ccggcattcttcCACCTGCACCCATAGCT
>DPCR1_CH3_CONTIG_201_p40   38 pairs of NGS reads, 0.08%
AAGCATGAGGCTATAATGATGTCACTGATAAGGTAGGAGAAAGAGCGAGGGATTGAGCGtttgaa
acaaatgcggcgggattgctttcttcagatctcccaccgagaaggcggtttctcgcacggcacac
gctttgtggtgtCGGTTTCCGATTTCTTGGAAGAAGTAGGAACCGGCATTCTTCCACCTGCACCC
ATGCAT
>DPCR1_CH3_CONTIG_202_p41   38 pairs of NGS reads, 0.08%
AAGCATGAGGCTATAATGATGTCACTGATAAGGTAGGAGAAAGAGCGAGGGATTGAGCGTttgaa
acatctcggtgggagatctgaagaaagcaatcccgccgcattaaggcggtttctcgcacggcaca
cgctttgtggtgTCGGTTTCCGATTTCTTGGAAGAAGTAGGAACCGGCATTCTTCCACCTGCACC
CATCGAT
>DPCR1_CH3_CONTIG_201_p42   38 pairs of NGS reads, 0.08%
AAGCATGAGGCTATAATGATGTCACTGATAAGGTAGGAGAAAGAGCGAGGGATTGAGCGtttgaa
acaaatgcggcgggattgctttcttcagatctcccaccgagaaggcggtttctcgcacggcacac
gctttgtggtgtCGGTTTCCGATTTCTTGGAAGAAGTAGGAACCGGCATTCTTCCACCTGCACCC

ATCGAT
>DPCR1_CH3_CONTIG_203_p43   37 pairs of NGS reads, 0.08%
AAGCATGAGGCTATAATGATGTCACTGATAAGGTAGGAGAAAGAGCGAGGGATTGAGCGTTtgaa
acaaatgcggcgggattgctttcttcagatctcccaccgagataaggcggtttctcgcacggcac
acgctttgtggtGTCGGTTTCCGATTTCTTGGAAGAAGTAGGAACCGGCATTCTTCCACCTGCAC
CCATGCAT
>DPCR1_CH3_CONTIG_203_p44   36 pairs of NGS reads, 0.07%
AAGCATGAGGCTATAATGATGTCACTGATAAGGTAGGAGAAAGAGCGAGGGATTGAGCGTTtgaa
acaaatgcggcgggattgctttcttcagatctcccaccgagaaaaggcggtttctcgcacggcac
acgctttgtggtGTCGGTTTCCGATTTCTTGGAAGAAGTAGGAACCGGCATTCTTCCACCTGCAC
CCATGCAT
>DPCR1_CH3_CONTIG_199_p45   36 pairs of NGS reads, 0.07%
AAGCATGAGGCTATAATGATGTCACTGATAAGGTAGGAGAAAGAGCGAGGGATTGAGcgtttgaa
acaatgcggcgggattgctttcttcagatctcccaccgagaggcggtttctcgcacggcacacgc
tttgtggtgtcgGTTTCCGATTTCTTGGAAGAAGTAGGAACCGGCATTCTTCCACCTGCACCCAT
CGAT
>DPCR1_CH3_CONTIG_200_p46   36 pairs of NGS reads, 0.07%
AAGCATGAGGCTATAATGATGTCACTGATAAGGTAGGAGAAAGAGCGAGGGATTGAGCgtttgaa
acatgcggcgggattgctttcttcagatctcccaccgagaaaggcggtttctcgcacggcacacg
ctttgtggtgtcGGTTTCCGATTTCTTGGAAGAAGTAGGAACCGGCATTCTTCCACCTGCACCCA
TAGCT
>DPCR1_CH3_CONTIG_202_p47   31 pairs of NGS reads, 0.06%
AAGCATGAGGCTATAATGATGTCACTGATAAGGTAGGAGAAAGAGCGAGGGATTGAGCGTttgaa
acaatgcggcgggattgctttcttcagatctcccaccgagacaaggcggtttctcgcacggcaca
cgctttgtggtgTCGGTTTCCGATTTCTTGGAAGAAGTAGGAACCGGCATTCTTCCACCTGCACC
CATAGCT
>DPCR1_CH3_CONTIG_192_p48   30 pairs of NGS reads, 0.06%
AAGCATGAGGCTATAATGATGTCCCCCAACATACCAAAACTAGCTGTCACtccaccggtagtagg
agatgtaagaattgatatatagaataacttttacttgattgataatcacataaaaccgaagaaa
ttttagccatttGCATCAAACTTAAACTTCCTTCTTGCATTCTTCCACCTGCACCCATGCAT
>DPCR1_CH3_CONTIG_160_p49   30 pairs of NGS reads, 0.06%
AAGCATGAGGCTATAATGatgtcactgataaggtaggagaaagagcgagggattgagcgtttgaa
aaggcggtttctcgcacggcacacgctttgtggtgtcggtttccgatttcttggaagaagtagga
accggcattcttCCACCTGCACCCATCGAT
>DPCR1_CH3_CONTIG_160_p50   28 pairs of NGS reads, 0.06%
AAGCATGAGGCTATAATGatgtcactgataaggtaggagaaagagcgagggattgagcgtttgaa
aaggcggtttctcgcacggcacacgctttgtggtgtcggtttccgatttcttggaagaagtagga
accggcattcttCCACCTGCACCCATGCAT
>DPCR1_CH3_CONTIG_203_p51   27 pairs of NGS reads, 0.05%
AAGCATGAGGCTATAATGATGTCACTGATAAGGTAGGAGAAAGAGCGAGGGATTGAGCGTTtgaa
acaaatgcggcgggattgctttcttcagatctcccaccgagataaggcggtttctcgcacggcac
acgctttgtggtGTCGGTTTCCGATTTCTTGGAAGAAGTAGGAACCGGCATTCTTCCACCTGCAC
CCATCGAT
>DPCR1_CH3_CONTIG_160_p52   26 pairs of NGS reads, 0.05%
AAGCATGAGGCTATAATGatgtcactgataaggtaggagaaagagcgagggattgagcgtttgaa
aaggcggtttctcgcacggcacacgctttgtggtgtcggtttccgatttcttggaagaagtagga
accggcattcttCCACCTGCACCCATAGCT
>DPCR1_CH3_CONTIG_127_p53   24 pairs of NGS reads, 0.05%
aagcatgaggctataatgatgtccacaagctgaacagacccatcatgaacctcaaaaagcgtgtc
tgaacaaaagaaaactttagcgagcctgctttcttgcattcttccacctgcacccatagct
>DPCR1_CH3_CONTIG_161_p54   24 pairs of NGS reads, 0.05%
AAGCATGAGGCTATAATGAtgtcactgataaggtaggagaaagagcgagggattgagcgtttgaa

aaaggcggtttctcgcacggcacacgctttgtggtgtcggtttccgatttcttggaagaagtagg
aaccggcattctTCCACCTGCACCCATAGCT
>DPCR1_CH3_CONTIG_203_p55   23 pairs of NGS reads, 0.05%
AAGCATGAGGCTATAATGATGTCACTGATAAGGTAGGAGAAAGAGCGAGGGATTGAGCGTTtgaa
acaaatgcggcgggattgctttcttcagatctcccaccgagaaaaggcggtttctcgcacggcac
acgctttgtggtGTCGGTTTCCGATTTCTTGGAAGAAGTAGGAACCGGCATTCTTCCACCTGCAC
CCATCGAT
>DPCR1_CH3_CONTIG_192_p56   22 pairs of NGS reads, 0.04%
AAGCATGAGGCTATAATGATGTCCCCCAACATACCAAAACTAGCTGTCACtccaccggtagtagg
agatgtaagaattgatatatagaataacttttacttgattgataatcacataaaaccgaagaaa
ttttagccatttGCATCAAACTTAAACTTCCTTCTTGCATTCTTCCACCTGCACCCATCGAT
>DPCR1_CH3_CONTIG_157_p57   21 pairs of NGS reads, 0.04%
AAGCATGAGGCTATAatgatgtcactgataaggtaggagaaagagcgagggattgagcgtttgaa
acggtttctcgcacggcacacgctttgtggtgtcggtttccgatttcttggaagaagtaggaacc
ggcattcttccaCCTGCACCCATGCAT

AtFAD RNP 2

>DPCR2_CH4_CONTIG_163_p1   7106 pairs of NGS reads, 15.67%
AAGCATGAGGCTAtaatgatgtcactgataaggtaggagaaagagcgagggattgagcgtttgaa
acaaaggcggtttctcgcacggcacacgctttgtggtgtcggtttccgatttcttggaagaagta
ggaaccggcattcttcCACCTGCACCCATAGCT
>DPCR2_CH4_CONTIG_163_p2   5955 pairs of NGS reads, 13.13%
AAGCATGAGGCTATAATGatgtcactgataaggtaggagaaagagcgagggattgagcgtttgaa
acaaaggcggtttctcgcacggcacacgctttgtggtgtcggtttccgatttcttggaagaagta
ggaaccggcattcttcCACCTGCACCCATCGAT
>DPCR2_CH4_CONTIG_163_p3   5788 pairs of NGS reads, 12.76%
AAGCATGAGGCTATAATGatgtcactgataaggtaggagaaagagcgagggattgagcgtttgaa
acaaaggcggtttctcgcacggcacacgctttgtggtgtcggtttccgatttcttggaagaagta
ggaaccggcattcttcCACCTGCACCCATGCAT
>DPCR2_CH4_CONTIG_201_p4   5782 pairs of NGS reads, 12.75%
AAGCATGAGGCTATAATGATGTCACTGATAAGGTAGGAGAAAGAGCGAGGGAttgagcgtttgaa
acaatgcggcgggattgctttcttcagatctcccaccgagaaaggcggtttctcgcacggcacac
gctttgtggtgtcgGTTTCCGATTTCTTGGAAGAAGTAGGAACCGGCATTCTTCCACCTGCACCC
ATAGCT
>DPCR2_CH4_CONTIG_201_p5   4967 pairs of NGS reads, 10.95%
AAGCATGAGGCTATAATGATGTCACTGATAAGGTAGGAGAAAGAGCGAGGGATTGAgcgtttgaa
acaatgcggcgggattgctttcttcagatctcccaccgagaaaggcggtttctcgcacggcacac
gctttgtggtgtcgGTTTCCGATTTCTTGGAAGAAGTAGGAACCGGCATTCTTCCACCTGCACCC
ATGCAT
>DPCR2_CH4_CONTIG_201_p6   4797 pairs of NGS reads, 10.57%
AAGCATGAGGCTATAATGATGTCACTGATAAGGTAGGAGAAAGAGCGAGGGATTGAgcgtttgaa
acaatgcggcgggattgctttcttcagatctcccaccgagaaaggcggtttctcgcacggcacac
gctttgtggtgtcgGTTTCCGATTTCTTGGAAGAAGTAGGAACCGGCATTCTTCCACCTGCACCC
ATCGAT
>DPCR2_CH4_CONTIG_162_p7   1632 pairs of NGS reads, 3.59%
AAGCATGAGGCTATAATGAtgtcactgataaggtaggagaaagagcgagggattgagcgtttgaa
acaaggcggtttctcgcacggcacacgctttgtggtgtcggtttccgatttcttggaagaagtag
gaaccggcattcttCCACCTGCACCCATAGCT
>DPCR2_CH4_CONTIG_162_p8   1412 pairs of NGS reads, 3.11%
AAGCATGAGGCTATAATGAtgtcactgataaggtaggagaaagagcgagggattgagcgtttgaa
acaaggcggtttctcgcacggcacacgctttgtggtgtcggtttccgatttcttggaagaagtag

gaaccggcattcttCCACCTGCACCCATCGAT
>DPCR2_CH4_CONTIG_162_p9   1321 pairs of NGS reads, 2.91%
AAGCATGAGGCTATAATGatgtcactgataaggtaggagaaagagcgagggattgagcgtttgaa
acaaggcggtttctcgcacggcacacgctttgtggtgtcggtttccgatttcttggaagaagtag
gaaccggcattcttCCACCTGCACCCATGCAT
>DPCR2_CH4_CONTIG_200_p10   353 pairs of NGS reads, 0.77%
AAGCATGAGGCTATAATGATGTCACTGATAAGGTAGGAGAAAGAGCGAGGGATTGAGCgtttgaa
acaatgcggcgggattgctttcttcagatctcccaccgagaaggcggtttctcgcacggcacacg
ctttgtggtgtcGGTTTCCGATTTCTTGGAAGAAGTAGGAACCGGCATTCTTCCACCTGCACCCA
TAGCT
>DPCR2_CH4_CONTIG_202_p11   257 pairs of NGS reads, 0.56%
AAGCATGAGGCTATAATGATGTCACTGATAAGGTAGGAGAAAGAGCGAGGGATTGAGCGTttgaa
acaatgcggcgggattgctttcttcagatctcccaccgagaaaaggcggtttctcgcacggcaca
cgctttgtggtgTCGGTTTCCGATTTCTTGGAAGAAGTAGGAACCGGCATTCTTCCACCTGCACC
CATAGCT
>DPCR2_CH4_CONTIG_200_p12   267 pairs of NGS reads, 0.58%
AAGCATGAGGCTATAATGATGTCACTGATAAGGTAGGAGAAAGAGCGAGGGATTGAGcgtttgaa
acaatgcggcgggattgctttcttcagatctcccaccgagaaggcggtttctcgcacggcacacg
ctttgtggtgtcGGTTTCCGATTTCTTGGAAGAAGTAGGAACCGGCATTCTTCCACCTGCACCCA
TGCAT
>DPCR2_CH4_CONTIG_200_p13   233 pairs of NGS reads, 0.51%
AAGCATGAGGCTATAATGATGTCACTGATAAGGTAGGAGAAAGAGCGAGGGATTGAGCgtttgaa
acaatgcggcgggattgctttcttcagatctcccaccgagaaggcggtttctcgcacggcacacg
ctttgtggtgtcGGTTTCCGATTTCTTGGAAGAAGTAGGAACCGGCATTCTTCCACCTGCACCCA
TCGAT
>DPCR2_CH4_CONTIG_202_p14   215 pairs of NGS reads, 0.47%
AAGCATGAGGCTATAATGATGTCACTGATAAGGTAGGAGAAAGAGCGAGGGATTGAGCGTttgaa
acaatgcggcgggattgctttcttcagatctcccaccgagataaggcggtttctcgcacggcaca
cgctttgtggtgtCGGTTTCCGATTTCTTGGAAGAAGTAGGAACCGGCATTCTTCCACCTGCACC
CATAGCT
>DPCR2_CH4_CONTIG_202_p15   207 pairs of NGS reads, 0.45%
AAGCATGAGGCTATAATGATGTCACTGATAAGGTAGGAGAAAGAGCGAGGGATTGAGCGTttgaa
acaatgcggcgggattgctttcttcagatctcccaccgagaaaaggcggtttctcgcacggcaca
cgctttgtggtgTCGGTTTCCGATTTCTTGGAAGAAGTAGGAACCGGCATTCTTCCACCTGCACC
CATCGAT
>DPCR2_CH4_CONTIG_164_p16   215 pairs of NGS reads, 0.47%
AAGCATGAGGCTATAATGATGTcactgataaggtaggagaaagagcgagggattgagcgtttgaa
acataaggcggtttctcgcacggcacacgctttgtggtgtcggtttccgatttcttggaagaagt
aggaaccggcattCTTCCACCTGCACCCATAGCT
>DPCR2_CH4_CONTIG_202_p17   206 pairs of NGS reads, 0.45%
AAGCATGAGGCTATAATGATGTCACTGATAAGGTAGGAGAAAGAGCGAGGGATTGAGCGTttgaa
acaatgcggcgggattgctttcttcagatctcccaccgagaaaaggcggtttctcgcacggcaca
cgctttgtggtgTCGGTTTCCGATTTCTTGGAAGAAGTAGGAACCGGCATTCTTCCACCTGCACC
CATGCAT
>DPCR2_CH4_CONTIG_202_p18   193 pairs of NGS reads, 0.42%
AAGCATGAGGCTATAATGATGTCACTGATAAGGTAGGAGAAAGAGCGAGGGATTGAGCGTttgaa
acaatgcggcgggattgctttcttcagatctcccaccgagataaggcggtttctcgcacggcaca
cgctttgtggtgtCGGTTTCCGATTTCTTGGAAGAAGTAGGAACCGGCATTCTTCCACCTGCACC
CATGCAT
>DPCR2_CH4_CONTIG_164_p19   190 pairs of NGS reads, 0.41%
AAGCATGAGGCTATAATGATGTcactgataaggtaggagaaagagcgagggattgagcgtttgaa
acataaggcggtttctcgcacggcacacgctttgtggtgtcggtttccgatttcttggaagaagt

aggaaccggcattCTTCCACCTGCACCCATCGAT
>DPCR2_CH4_CONTIG_161_p20   189 pairs of NGS reads, 0.41%
AAGCATGAGGCTATAATGAtgtcactgataaggtaggagaaagagcgagggattgagcgtttgaa
acaggcggtttctcgcacggcacacgctttgtggtgtcggtttccgatttcttggaagaagtagg
aaccggcattcttCCACCTGCACCCATAGCT
>DPCR2_CH4_CONTIG_202_p21   183 pairs of NGS reads, 0.4%
AAGCATGAGGCTATAATGATGTCACTGATAAGGTAGGAGAAAGAGCGAGGGATTGAGCGTttgaa
acaatgcggcgggattgctttcttcagatctcccaccgagataaggcggtttctcgcacggcaca
cgctttgtggtgtCGGTTTCCGATTTCTTGGAAGAAGTAGGAACCGGCATTCTTCCACCTGCACC
CATCGAT
>DPCR2_CH4_CONTIG_164_p22   178 pairs of NGS reads, 0.39%
AAGCATGAGGCTATAATGATGTcactgataaggtaggagaaagagcgagggattgagcgtttgaa
acataaggcggtttctcgcacggcacacgctttgtggtgtcggtttccgatttcttggaagaagt
aggaaccggcattCTTCCACCTGCACCCATGCAT
>DPCR2_CH4_CONTIG_161_p23   177 pairs of NGS reads, 0.39%
AAGCATGAGGCTATAATGAtgtcactgataaggtaggagaaagagcgagggattgagcgtttgaa
acaggcggtttctcgcacggcacacgctttgtggtgtcggtttccgatttcttggaagaagtagg
aaccggcattcttCCACCTGCACCCATGCAT
>DPCR2_CH4_CONTIG_161_p24   166 pairs of NGS reads, 0.36%
AAGCATGAGGCTATAATGAtgtcactgataaggtaggagaaagagcgagggattgagcgtttgaa
acaggcggtttctcgcacggcacacgctttgtggtgtcggtttccgatttcttggaagaagtagg
aaccggcattcttCCACCTGCACCCATCGAT
>DPCR2_CH4_CONTIG_202_p25   136 pairs of NGS reads, 0.29%
AAGCATGAGGCTATAATGATGTCACTGATAAGGTAGGAGAAAGAGCGAGGGATTGAGCGTttgaa
acaaatgcggcgggattgctttcttcagatctcccaccgagaaaggcggtttctcgcacggcaca
cgctttgtggtgTCGGTTTCCGATTTCTTGGAAGAAGTAGGAACCGGCATTCTTCCACCTGCACC
CATCGAT
>DPCR2_CH4_CONTIG_164_p26   120 pairs of NGS reads, 0.26%
AAGCATGAGGCTATAATGATGTcactgataaggtaggagaaagagcgagggattgagcgtttgaa
acaaaaggcggtttctcgcacggcacacgctttgtggtgtcggtttccgatttcttggaagaagt
aggaaccggcattCTTCCACCTGCACCCATAGCT
>DPCR2_CH4_CONTIG_202_p27   114 pairs of NGS reads, 0.25%
AAGCATGAGGCTATAATGATGTCACTGATAAGGTAGGAGAAAGAGCGAGGGATTGAGCGTttgaa
acaaatgcggcgggattgctttcttcagatctcccaccgagaaaggcggtttctcgcacggcaca
cgctttgtggtgTCGGTTTCCGATTTCTTGGAAGAAGTAGGAACCGGCATTCTTCCACCTGCACC
CATAGCT
>DPCR2_CH4_CONTIG_127_p28   94 pairs of NGS reads, 0.2%
aagcatgaggctataatgatgtccacaagctgaacagacccatcatgaacctcaaaaagcgtgtc
tgaacaaaagaaaactttagcgagcctgctttcttgcattcttccacctgcacccatcgat
>DPCR2_CH4_CONTIG_202_p29   93 pairs of NGS reads, 0.2%
AAGCATGAGGCTATAATGATGTCACTGATAAGGTAGGAGAAAGAGCGAGGGATTGAGCGTttgaa
acaaatgcggcgggattgctttcttcagatctcccaccgagaaaggcggtttctcgcacggcaca
cgctttgtggtgTCGGTTTCCGATTTCTTGGAAGAAGTAGGAACCGGCATTCTTCCACCTGCACC
CATGCAT
>DPCR2_CH4_CONTIG_164_p30   82 pairs of NGS reads, 0.18%
AAGCATGAGGCTATAATGATGTcactgataaggtaggagaaagagcgagggattgagcgtttgaa
acaaaaggcggtttctcgcacggcacacgctttgtggtgtcggtttccgatttcttggaagaagt
aggaaccggcattCTTCCACCTGCACCCATGCAT
>DPCR2_CH4_CONTIG_202_p31   77 pairs of NGS reads, 0.16%
AAGCATGAGGCTATAATGATGTCACTGATAAGGTAGGAGAAAGAGCGAGGGATTGAGCGTttgaa
acatctcggtgggagatctgaagaaagcaatcccgccgcattaaggcggtttctcgcacggcaca
cgctttgtggtgTCGGTTTCCGATTTCTTGGAAGAAGTAGGAACCGGCATTCTTCCACCTGCACC

```
CATGCAT
>DPCR2_CH4_CONTIG_199_p32    76 pairs of NGS reads, 0.16%
AAGCATGAGGCTATAATGATGTCACTGATAAGGTAGGAGAAAGAGCGAGGGATTGAGcgtttgaa
acaatgcggcgggattgctttcttcagatctcccaccgagaggcggtttctcgcacggcacacgc
tttgtggtgtcgGTTTCCGATTTCTTGGAAGAAGTAGGAACCGGCATTCTTCCACCTGCACCCAT
AGCT
>DPCR2_CH4_CONTIG_202_p33    76 pairs of NGS reads, 0.16%
AAGCATGAGGCTATAATGATGTCACTGATAAGGTAGGAGAAAGAGCGAGGGATTGAGCGTttgaa
acatctcggtgggagatctgaagaaagcaatcccgccgcattaaggcggtttctcgcacggcaca
cgctttgtggtgTCGGTTTCCGATTTCTTGGAAGAAGTAGGAACCGGCATTCTTCCACCTGCACC
CATAGCT
>DPCR2_CH4_CONTIG_164_p34    72 pairs of NGS reads, 0.15%
AAGCATGAGGCTATAATGATGTcactgataaggtaggagaaagagcgagggattgagcgtttgaa
acaaaaggcggtttctcgcacggcacacgctttgtggtgtcggtttccgatttcttggaagaagt
aggaaccggcattCTTCCACCTGCACCCATCGAT
>DPCR2_CH4_CONTIG_202_p35    69 pairs of NGS reads, 0.15%
AAGCATGAGGCTATAATGATGTCACTGATAAGGTAGGAGAAAGAGCGAGGGATTGAGCGTttgaa
acatctcggtgggagatctgaagaaagcaatcccgccgcattaaggcggtttctcgcacggcaca
cgctttgtggtgTCGGTTTCCGATTTCTTGGAAGAAGTAGGAACCGGCATTCTTCCACCTGCACC
CATCGAT
>DPCR2_CH4_CONTIG_199_p36    68 pairs of NGS reads, 0.14%
AAGCATGAGGCTATAATGATGTCACTGATAAGGTAGGAGAAAGAGCGAGGGATTGAGcgtttgaa
acaatgcggcgggattgctttcttcagatctcccaccgagaggcggtttctcgcacggcacacgc
tttgtggtgtcgGTTTCCGATTTCTTGGAAGAAGTAGGAACCGGCATTCTTCCACCTGCACCCAT
GCAT
>DPCR2_CH4_CONTIG_127_p37    68 pairs of NGS reads, 0.14%
aagcatgaggctataatgatgtccacaagctgaacagacccatcatgaacctcaaaaagcgtgtc
tgaacaaaagaaaacttttagcgagcctgctttcttgcattcttccacctgcacccatgcat
>DPCR2_CH4_CONTIG_201_p38    64 pairs of NGS reads, 0.14%
AAGCATGAGGCTATAATGATGTCACTGATAAGGTAGGAGAAAGAGCGAGGGATTGAGCGtttgaa
acaaatgcggcgggattgctttcttcagatctcccaccgagaaggcggtttctcgcacggcacac
gctttgtggtgtCGGTTTCCGATTTCTTGGAAGAAGTAGGAACCGGCATTCTTCCACCTGCACCC
ATAGCT
>DPCR2_CH4_CONTIG_159_p39    53 pairs of NGS reads, 0.11%
AAGCATGAGGCTATAATgatgtcactgataaggtaggagaaagagcgagggattgagcgtttgaa
aggcggtttctcgcacggcacacgctttgtggtgtcggtttccgatttcttggaagaagtaggaa
ccggcattcttcCACCTGCACCCATAGCT
>DPCR2_CH4_CONTIG_201_p40    49 pairs of NGS reads, 0.1%
AAGCATGAGGCTATAATGATGTCACTGATAAGGTAGGAGAAAGAGCGAGGGATTGAGCGtttgaa
acaaatgcggcgggattgctttcttcagatctcccaccgagaaggcggtttctcgcacggcacac
gctttgtggtgtCGGTTTCCGATTTCTTGGAAGAAGTAGGAACCGGCATTCTTCCACCTGCACCC
ATGCAT
>DPCR2_CH4_CONTIG_159_p41    42 pairs of NGS reads, 0.09%
AAGCATGAGGCTATAATgatgtcactgataaggtaggagaaagagcgagggattgagcgtttgaa
aggcggtttctcgcacggcacacgctttgtggtgtcggtttccgatttcttggaagaagtaggaa
ccggcattcttcCACCTGCACCCATGCAT
>DPCR2_CH4_CONTIG_160_p42    41 pairs of NGS reads, 0.09%
AAGCATGAGGCTATAATGatgtcactgataaggtaggagaaagagcgagggattgagcgtttgaa
aaggcggtttctcgcacggcacacgctttgtggtgtcggtttccgatttcttggaagaagtagga
accggcattcttCCACCTGCACCCATGCAT
>DPCR2_CH4_CONTIG_127_p43    38 pairs of NGS reads, 0.08%
aagcatgaggctataatgatgtccacaagctgaacagacccatcatgaacctcaaaaagcgtgtc
```

tgaacaaaagaaaacttttagcgagcctgctttcttgcattcttccacctgcacccatagct
>DPCR2_CH4_CONTIG_135_p44   37 pairs of NGS reads, 0.08%
aagcatgaggctataatgatgtcactgataaggtaggagaaaggcggtttctcgcacggcacacg
ctttgtggtgtcggtttccgatttcttggaagaagtaggaaccggcattcttccacctgcaccca
tgcat
>DPCR2_CH4_CONTIG_203_p45   35 pairs of NGS reads, 0.07%
AAGCATGAGGCTATAATGATGTCACTGATAAGGTAGGAGAAAGAGCGAGGGATTGAGCGTTtgaa
acaaatgcggcgggattgctttcttcagatctcccaccgagataaggcggtttctcgcacggcac
acgctttgtggtGTCGGTTTCCGATTTCTTGGAAGAAGTAGGAACCGGCATTCTTCCACCTGCAC
CCATAGCT
>DPCR2_CH4_CONTIG_199_p46   34 pairs of NGS reads, 0.07%
AAGCATGAGGCTATAATGATGTCACTGATAAGGTAGGAGAAAGAGCGAGGGATTGAGcgtttgaa
acaatgcggcgggattgctttcttcagatctcccaccgagaggcggtttctcgcacggcacacgc
tttgtggtgtcgGTTTCCGATTTCTTGGAAGAAGTAGGAACCGGCATTCTTCCACCTGCACCCAT
CGAT
>DPCR2_CH4_CONTIG_124_p47   34 pairs of NGS reads, 0.07%
aagcatgaggctataatgatgtcactgataaggcggtttctcgcacggcacacgctttgtggtgt
cggtttccgatttcttggaagaagtaggaaccggcattcttccacctgcacccatcgat
>DPCR2_CH4_CONTIG_202_p48   34 pairs of NGS reads, 0.07%
AAGCATGAGGCTATAATGATGTCACTGATAAGGTAGGAGAAAGAGCGAGGGATTGAGCGTttgaa
acaatgcggcgggattgctttcttcagatctcccaccgagacaaggcggtttctcgcacggcaca
cgctttgtggtgTCGGTTTCCGATTTCTTGGAAGAAGTAGGAACCGGCATTCTTCCACCTGCACC
CATAGCT
>DPCR2_CH4_CONTIG_174_p49   33 pairs of NGS reads, 0.07%
AAGCATGAGGCTATAATGATGTCTGTGTAGGAagactttcagctcaaggactcgtggtcgataac
ggctctactcgccgtggccacaaggcgttgcgctatccgcaacatacgctggctgggctgatggc
tgaaggggtggtATGCCGGCATTCTTCCACCTGCACCCATAGCT
>DPCR2_CH4_CONTIG_203_p50   32 pairs of NGS reads, 0.07%
AAGCATGAGGCTATAATGATGTCACTGATAAGGTAGGAGAAAGAGCGAGGGATTGAGCGTTtgaa
acaaatgcggcgggattgctttcttcagatctcccaccgagaaaaggcggtttctcgcacggcac
acgctttgtggtGTCGGTTTCCGATTTCTTGGAAGAAGTAGGAACCGGCATTCTTCCACCTGCAC
CCATAGCT
>DPCR2_CH4_CONTIG_192_p51   30 pairs of NGS reads, 0.06%
AAGCATGAGGCTATAATGATGTCCCCCAACATACCAAAACTAGCTGTCACtccaccggtagtagg
agatgtaagaattgatatatagaataacttttacttgattgataatcacataaaaccgaagaaa
ttttagccatttGCATCAAACTTAAACTTCCTTCTTGCATTCTTCCACCTGCACCCATAGCT
>DPCR2_CH4_CONTIG_199_p52   30 pairs of NGS reads, 0.06%
AAGCATGAGGCTATAATGATGTCACTGATAAGGTAGGAGAAAGAGCGAGGGATTGAGcgtttgaa
acaatgcggcgggattgctttcttcagatctcccaccgaaaggcggtttctcgcacggcacacgc
tttgtggtgtcgGTTTCCGATTTCTTGGAAGAAGTAGGAACCGGCATTCTTCCACCTGCACCCAT
AGCT
>DPCR2_CH4_CONTIG_135_p53   30 pairs of NGS reads, 0.06%
aagcatgaggctataatgatgtcactgataaggtaggagaaaggcggtttctcgcacggcacacg
ctttgtggtgtcggtttccgatttcttggaagaagtaggaaccggcattcttccacctgcaccca
tcgat
>DPCR2_CH4_CONTIG_201_p54   29 pairs of NGS reads, 0.06%
AAGCATGAGGCTATAATGATGTCACTGATAAGGTAGGAGAAAGAGCGAGGGATTGAGCGtttgaa
acaaatgcggcgggattgctttcttcagatctcccaccgagaaggcggtttctcgcacggcacac
gctttgtggtgtCGGTTTCCGATTTCTTGGAAGAAGTAGGAACCGGCATTCTTCCACCTGCACCC
ATCGAT
>DPCR2_CH4_CONTIG_159_p55   29 pairs of NGS reads, 0.06%
AAGCATGAGGCTATAATgatgtcactgataaggtaggagaaagagcgagggattgagcgtttgaa

aggcggtttctcgcacggcacacgctttgtggtgtcggtttccgatttcttggaagaagtaggaa
ccggcattcttcCACCTGCACCCATCGAT
>DPCR2_CH4_CONTIG_202_p56   28 pairs of NGS reads, 0.06%
AAGCATGAGGCTATAATGATGTCACTGATAAGGTAGGAGAAAGAGCGAGGGATTGAGCGTttgaa
acaatgcggcgggattgctttcttcagatctcccaccgagacaaggcggtttctcgcacggcaca
cgctttgtggtgTCGGTTTCCGATTTCTTGGAAGAAGTAGGAACCGGCATTCTTCCACCTGCACC
CATGCAT
>DPCR2_CH4_CONTIG_160_p57   26 pairs of NGS reads, 0.05%
AAGCATGAGGCTATAATGatgtcactgataaggtaggagaaagagcgagggattgagcgtttgaa
aaggcggtttctcgcacggcacacgctttgtggtgtcggtttccgatttcttggaagaagtagga
accggcattcttCCACCTGCACCCATAGCT
>DPCR2_CH4_CONTIG_203_p58   26 pairs of NGS reads, 0.05%
AAGCATGAGGCTATAATGATGTCACTGATAAGGTAGGAGAAAGAGCGAGGGATTGAGCGTTtgaa
acaaatgcggcgggattgctttcttcagatctcccaccgagaaaaggcggtttctcgcacggcac
acgctttgtggtGTCGGTTTCCGATTTCTTGGAAGAAGTAGGAACCGGCATTCTTCCACCTGCAC
CCATCGAT
>DPCR2_CH4_CONTIG_160_p59   26 pairs of NGS reads, 0.05%
AAGCATGAGGCTATAATGatgtcactgataaggtaggagaaagagcgagggattgagcgtttgaa
aaggcggtttctcgcacggcacacgctttgtggtgtcggtttccgatttcttggaagaagtagga
accggcattcttCCACCTGCACCCATCGAT
>DPCR2_CH4_CONTIG_203_p60   25 pairs of NGS reads, 0.05%
AAGCATGAGGCTATAATGATGTCACTGATAAGGTAGGAGAAAGAGCGAGGGATTGAGCGTTtgaa
acaaatgcggcgggattgctttcttcagatctcccaccgagaaaaggcggtttctcgcacggcac
acgctttgtggtGTCGGTTTCCGATTTCTTGGAAGAAGTAGGAACCGGCATTCTTCCACCTGCAC
CCATGCAT
>DPCR2_CH4_CONTIG_158_p61   25 pairs of NGS reads, 0.05%
AAGCATGAGGCTATAAtgatgtcactgataaggtaggagaaagagcgagggattgagcgtttgaa
ggcggtttctcgcacggcacacgctttgtggtgtcggtttccgatttcttggaagaagtaggaac
cggcattcttccACCTGCACCCATGCAT
>DPCR2_CH4_CONTIG_203_p62   21 pairs of NGS reads, 0.04%
AAGCATGAGGCTATAATGATGTCACTGATAAGGTAGGAGAAAGAGCGAGGGATTGAGCGTTtgaa
acaaatgcggcgggattgctttcttcagatctcccaccgagataaggcggtttctcgcacggcac
acgctttgtggtGTCGGTTTCCGATTTCTTGGAAGAAGTAGGAACCGGCATTCTTCCACCTGCAC
CCATGCAT
>DPCR2_CH4_CONTIG_161_p63   21 pairs of NGS reads, 0.04%
AAGCATGAGGCTATAATGAtgtcactgataaggtaggagaaagagcgagggattgagcgtttgaa
aaaggcggtttctcgcacggcacacgctttgtggtgtcggtttccgatttcttggaagaagtagg
aaccggcattctTCCACCTGCACCCATAGCT
>DPCR2_CH4_CONTIG_124_p64   21 pairs of NGS reads, 0.04%
aagcatgaggctataatgatgtcactgataaggcggtttctcgcacggcacacgctttgtggtgt
cggtttccgatttcttggaagaagtaggaaccggcattcttccacctgcacccatagct
>DPCR2_CH4_CONTIG_201_p65   19 pairs of NGS reads, 0.04%
AAGCATGAGGCTATAATGATGTCACTGATAAGGTAGGAGAAAGAGCGAGGGATTGAGCGtttgaa
acatgcggcgggattgctttcttcagatctcccaccgagaaaaggcggtttctcgcacggcacac
gctttgtggtgtCGGTTTCCGATTTCTTGGAAGAAGTAGGAACCGGCATTCTTCCACCTGCACCC
ATGCAT
>DPCR2_CH4_CONTIG_202_p66   19 pairs of NGS reads, 0.04%
AAGCATGAGGCTATAATGATGTCACTGATAAGGTAGGAGAAAGAGCGAGGGATTGAGCGTttgaa
acaatgcggcgggattgctttcttcagatctcccaccgagacaaggcggtttctcgcacggcaca
cgctttgtggtgTCGGTTTCCGATTTCTTGGAAGAAGTAGGAACCGGCATTCTTCCACCTGCACC
CATCGAT
>DPCR2_CH4_CONTIG_200_p67   18 pairs of NGS reads, 0.03%

AAGCATGAGGCTATAATGATGTCACTGATAAGGTAGGAGAAAGAGCGAGGGATTGAGCgtttgaa
aatgcggcgggattgctttcttcagatctcccaccgagataaggcggtttctcgcacggcacacg
ctttgtggtgtcGGTTTCCGATTTCTTGGAAGAAGTAGGAACCGGCATTCTTCCACCTGCACCCA
TGCAT
>DPCR2_CH4_CONTIG_161_p68 17 pairs of NGS reads, 0.03%
AAGCATGAGGCTATAATGAtgtcactgataaggtaggagaaagagcgagggattgagcgtttgaa
aaaggcggtttctcgcacggcacacgctttgtggtgtcggtttccgatttcttggaagaagtagg
aaccggcattctTCCACCTGCACCCATCGAT
>DPCR2_CH4_CONTIG_199_p69 16 pairs of NGS reads, 0.03%
AAGCATGAGGCTATAATGATGTCACTGATAAGGTAGGAGAAAGAGCGAGGGATTGAGcgtttgaa
acaatgcggcgggattgctttcttcagatctcccaccgaaaggcggtttctcgcacggcacacgc
tttgtggtgtcgGTTTCCGATTTCTTGGAAGAAGTAGGAACCGGCATTCTTCCACCTGCACCCAT
GCAT
>DPCR2_CH4_CONTIG_161_p70 16 pairs of NGS reads, 0.03%
AAGCATGAGGCTATAATGAtgtcactgataaggtaggagaaagagcgagggattgagcgtttgaa
aaaggcggtttctcgcacggcacacgctttgtggtgtcggtttccgatttcttggaagaagtagg
aaccggcattctTCCACCTGCACCCATGCAT
>DPCR2_CH4_CONTIG_135_p71 14 pairs of NGS reads, 0.03%
aagcatgaggctataatgatgtcactgataaggtaggagaaaggcggtttctcgcacggcacacg
ctttgtggtgtcggtttccgatttcttggaagaagtaggaaccggcattcttccacctgcaccca
tagct
>DPCR2_CH4_CONTIG_203_p72 14 pairs of NGS reads, 0.03%
AAGCATGAGGCTATAATGATGTCACTGATAAGGTAGGAGAAAGAGCGAGGGATTGAGCGTTtgaa
acaaatgcggcgggattgctttcttcagatctcccaccgagataaggcggtttctcgcacggcac
acgctttgtggtGTCGGTTTCCGATTTCTTGGAAGAAGTAGGAACCGGCATTCTTCCACCTGCAC
CCATCGAT
>DPCR2_CH4_CONTIG_189_p73 14 pairs of NGS reads, 0.03%
AAGCATGAGGCTATAATGATGTCACTGATAAGGTAGGAGAAAGAGCGagggattgagcgtttgaa
gtcacatattttcaagtccttttgtgtgaaaacaaacattattacctcgaatgtggacaagtgac
aattttcataagACCAGTACCAATTCTTTGTTTGCATTCTTCCACCTGCACCCATGCAT
>DPCR2_CH4_CONTIG_201_p74 14 pairs of NGS reads, 0.03%
AAGCATGAGGCTATAATGATGTCACTGATAAGGTAGGAGAAAGAGCGAGGGATTGAGCGtttgaa
actctcggtgggagatctgaagaaagcaatcccgccgcattaaggcggtttctcgcacggcacac
gctttgtggtgtCGGTTTCCGATTTCTTGGAAGAAGTAGGAACCGGCATTCTTCCACCTGCACCC
ATAGCT
>DPCR2_CH4_CONTIG_193_p75 14 pairs of NGS reads, 0.03%
AAGCATGAGGCTATAATGATGTCACTGATAAGGTAGGAGAAAGAGCGAGGGattgagcgtttgaa
acaatgcggcgggattgctttcttcagatctcccaccgagattctcgcacggcacacgctttgtg
gtgtcggtttccGATTTCTTGGAAGAAGTAGGAACCGGCATTCTTCCACCTGCACCCATGCAT
>DPCR2_CH4_CONTIG_157_p76 14 pairs of NGS reads, 0.03%
AAGCATGAGGCTATAatgatgtcactgataaggtaggagaaagagcgagggattgagcgtttaag
gcggtttctcgcacggcacacgctttgtggtgtcggtttccgatttcttggaagaagtaggaacc
ggcattcttccaCCTGCACCCATCGAT
>DPCR2_CH4_CONTIG_151_p77 14 pairs of NGS reads, 0.03%
AAGCATGAGgctataatgatgtcactgataaggtaggagaaagagcgagggattgagcgtttgaa
acacgcacggcacacgctttgtggtgtcggtttccgatttcttggaagaagtaggaaccggcatt
cttccacctgcaCCCATCGAT
>DPCR2_CH4_CONTIG_173_p78 14 pairs of NGS reads, 0.03%
AAGCATGAGGCTATAATGATGTCACTGATAAggtaggagaaagagcgagggattgagcgttatgc
ggcgggattgctttcttcagatctcccaccgacacacgctttgtggtgtcggtttccgatttctt
ggaagaagtaggAACCGGCATTCTTCCACCTGCACCCATGCAT
>DPCR2_CH4_CONTIG_194_p79 13 pairs of NGS reads, 0.02%

AAGCATGAGGCTATAATGATGTCACTGATAAGGTAGGAGAAAGAGCGAGGGAttgagcgtttgaa
acaatgcggcgggattgctttcttcagatctcccaccgagatttctcgcacggcacacgctttgt
ggtgtcggtttcCGATTTCTTGGAAGAAGTAGGAACCGGCATTCTTCCACCTGCACCCATGCAT
>DPCR2_CH4_CONTIG_202_p80   13 pairs of NGS reads, 0.02%
AAGCATGAGGCTATAATGATGTCACTGATAAGGTAGGAGAAAGAGCGAGGGATTGAGCGTttgaa
acaatgcggcgggattgctttcttcagatctcccaccgagagaaggcggtttctcgcacggcaca
cgctttgtggtgTCGGTTTCCGATTTCTTGGAAGAAGTAGGAACCGGCATTCTTCCACCTGCACC
CATAGCT
>DPCR2_CH4_CONTIG_200_p81   13 pairs of NGS reads, 0.02%
AAGCATGAGGCTATAATGATGTCACTGATAAGGTAGGAGAAAGAGCGAGGGATTGAGCgtttgaa
acatgcggcgggattgctttcttcagatctcccaccgagaaaggcggtttctcgcacggcacacg
ctttgtggtgtcGGTTTCCGATTTCTTGGAAGAAGTAGGAACCGGCATTCTTCCACCTGCACCCA
TCGAT
>DPCR2_CH4_CONTIG_164_p82   13 pairs of NGS reads, 0.02%
AAGCATGAGGCTATAATGATGTcactgataaggtaggagaaagagcgagggattgagcgtttgaa
acagaaggcggtttctcgcacggcacacgctttgtggtgtcggtttccgatttcttggaagaagt
aggaaccggcatTCTTCCACCTGCACCCATCGAT
>DPCR2_CH4_CONTIG_124_p83   13 pairs of NGS reads, 0.02%
aagcatgaggctataatgatgtcactgataaggcggtttctcgcacggcacacgctttgtggtgt
cggtttccgatttcttggaagaagtaggaaccggcattcttccacctgcacccatgcat
>DPCR2_CH4_CONTIG_201_p84   13 pairs of NGS reads, 0.02%
AAGCATGAGGCTATAATGATGTCACTGATAAGGTAGGAGAAAGAGCGAGGGATTGAGCGTttgaa
acatctcggtgggagatctgaagaaagcaatcccgccgcataaggcggtttctcgcacggcacac
gctttgtggtgtCGGTTTCCGATTTCTTGGAAGAAGTAGGAACCGGCATTCTTCCACCTGCACCC
ATAGCT

AtFAD RNP 3

>DPCR3_CH5_CONTIG_163_p1   7582 pairs of NGS reads, 15.3%
AAGCATGAGGCTAtaatgatgtcactgataaggtaggagaaagagcgagggattgagcgtttgaa
acaaaggcggtttctcgcacggcacacgctttgtggtgtcggtttccgatttcttggaagaagta
ggaaccggcattcttCCACCTGCACCCATGCAT
>DPCR3_CH5_CONTIG_201_p2   6396 pairs of NGS reads, 12.91%
AAGCATGAGGCTATAATGATGTCACTGATAAGGTAGGAGAAAGAGCGAGGGattgagcgtttgaa
acaatgcggcgggattgctttcttcagatctcccaccgagaaaggcggtttctcgcacggcacac
gctttgtggtgtcggTTTCCGATTTCTTGGAAGAAGTAGGAACCGGCATTCTTCCACCTGCACCC
ATGCAT
>DPCR3_CH5_CONTIG_163_p3   5825 pairs of NGS reads, 11.75%
AAGCATGAGGCTATAATGatgtcactgataaggtaggagaaagagcgagggattgagcgtttgaa
acaaaggcggtttctcgcacggcacacgctttgtggtgtcggtttccgatttcttggaagaagta
ggaaccggcattcttCCACCTGCACCCATCGAT
>DPCR3_CH5_CONTIG_201_p4   5773 pairs of NGS reads, 11.65%
AAGCATGAGGCTATAATGATGTCACTGATAAGGTAGGAGAAAGAGCGAGGGATTGAgcgtttgaa
acaatgcggcgggattgctttcttcagatctcccaccgagaaaggcggtttctcgcacggcacac
gctttgtggtgtcggTTTCCGATTTCTTGGAAGAAGTAGGAACCGGCATTCTTCCACCTGCACCC
ATCGAT
>DPCR3_CH5_CONTIG_201_p5   5653 pairs of NGS reads, 11.41%
AAGCATGAGGCTATAATGATGTCACTGATAAGGTAGGAGAAAGAGCGAGGGATTGAgcgtttgaa
acaatgcggcgggattgctttcttcagatctcccaccgagaaaggcggtttctcgcacggcacac
gctttgtggtgtcggTTTCCGATTTCTTGGAAGAAGTAGGAACCGGCATTCTTCCACCTGCACCC
ATAGCT
>DPCR3_CH5_CONTIG_163_p6   5283 pairs of NGS reads, 10.66%

AAGCATGAGGCTATAATGatgtcactgataaggtaggagaaagagcgagggattgagcgtttgaa
acaaaggcggtttctcgcacggcacacgctttgtggtgtcggtttccgatttcttggaagaagta
ggaaccggcattcttCCACCTGCACCCATAGCT
>DPCR3_CH5_CONTIG_162_p7   1778 pairs of NGS reads, 3.58%
AAGCATGAGGCTATAAtgatgtcactgataaggtaggagaaagagcgagggattgagcgtttgaa
acaaggcggtttctcgcacggcacacgctttgtggtgtcggtttccgatttcttggaagaagtag
gaaccggcattcttCCACCTGCACCCATGCAT
>DPCR3_CH5_CONTIG_162_p8   1398 pairs of NGS reads, 2.82%
AAGCATGAGGCTATAATGATgtcactgataaggtaggagaaagagcgagggattgagcgtttgaa
acaaggcggtttctcgcacggcacacgctttgtggtgtcggtttccgatttcttggaagaagtag
gaaccggcattcttCCACCTGCACCCATCGAT
>DPCR3_CH5_CONTIG_162_p9   1271 pairs of NGS reads, 2.56%
AAGCATGAGGCTATAATGATgtcactgataaggtaggagaaagagcgagggattgagcgtttgaa
acaaggcggtttctcgcacggcacacgctttgtggtgtcggtttccgatttcttggaagaagtag
gaaccggcattcttCCACCTGCACCCATAGCT
>DPCR3_CH5_CONTIG_200_p10   523 pairs of NGS reads, 1.05%
AAGCATGAGGCTATAATGATGTCACTGATAAGGTAGGAGAAAGAGCGAGGGATTGAGCgtttgaa
acaatgcggcgggattgctttcttcagatctcccaccgagaaggcggtttctcgcacggcacacg
ctttgtggtgtcggTTTCCGATTTCTTGGAAGAAGTAGGAACCGGCATTCTTCCACCTGCACCCA
TGCAT
>DPCR3_CH5_CONTIG_200_p11   396 pairs of NGS reads, 0.79%
AAGCATGAGGCTATAATGATGTCACTGATAAGGTAGGAGAAAGAGCGAGGGATTGAGCgtttgaa
acaatgcggcgggattgctttcttcagatctcccaccgagaaggcggtttctcgcacggcacacg
ctttgtggtgtcggTTTCCGATTTCTTGGAAGAAGTAGGAACCGGCATTCTTCCACCTGCACCCA
TCGAT
>DPCR3_CH5_CONTIG_202_p12   393 pairs of NGS reads, 0.79%
AAGCATGAGGCTATAATGATGTCACTGATAAGGTAGGAGAAAGAGCGAGGGATTGAGCGtttgaa
acaatgcggcgggattgctttcttcagatctcccaccgagaaaaggcggtttctcgcacggcaca
cgctttgtggtgtCGGTTTCCGATTTCTTGGAAGAAGTAGGAACCGGCATTCTTCCACCTGCACC
CATGCAT
>DPCR3_CH5_CONTIG_200_p13   359 pairs of NGS reads, 0.72%
AAGCATGAGGCTATAATGATGTCACTGATAAGGTAGGAGAAAGAGCGAGGGATTGAGCgtttgaa
acaatgcggcgggattgctttcttcagatctcccaccgagaaggcggtttctcgcacggcacacg
ctttgtggtgtcggTTTCCGATTTCTTGGAAGAAGTAGGAACCGGCATTCTTCCACCTGCACCCA
TAGCT
>DPCR3_CH5_CONTIG_202_p14   317 pairs of NGS reads, 0.63%
AAGCATGAGGCTATAATGATGTCACTGATAAGGTAGGAGAAAGAGCGAGGGATTGAGCGTttgaa
acaatgcggcgggattgctttcttcagatctcccaccgagaaaaggcggtttctcgcacggcaca
cgctttgtggtgtCGGTTTCCGATTTCTTGGAAGAAGTAGGAACCGGCATTCTTCCACCTGCACC
CATCGAT
>DPCR3_CH5_CONTIG_202_p15   330 pairs of NGS reads, 0.66%
AAGCATGAGGCTATAATGATGTCACTGATAAGGTAGGAGAAAGAGCGAGGGATTGAGCGTttgaa
acaatgcggcgggattgctttcttcagatctcccaccgagaaaaggcggtttctcgcacggcaca
cgctttgtggtgtCGGTTTCCGATTTCTTGGAAGAAGTAGGAACCGGCATTCTTCCACCTGCACC
CATAGCT
>DPCR3_CH5_CONTIG_202_p16   267 pairs of NGS reads, 0.53%
AAGCATGAGGCTATAATGATGTCACTGATAAGGTAGGAGAAAGAGCGAGGGATTGAGCGTttgaa
acaatgcggcgggattgctttcttcagatctcccaccgagataaggcggtttctcgcacggcaca
cgctttgtggtgtCGGTTTCCGATTTCTTGGAAGAAGTAGGAACCGGCATTCTTCCACCTGCACC
CATGCAT
>DPCR3_CH5_CONTIG_164_p17   236 pairs of NGS reads, 0.47%
AAGCATGAGGCTATAATGATGTcactgataaggtaggagaaagagcgagggattgagcgtttgaa

acataaggcggtttctcgcacggcacacgctttgtggtgtcggtttccgatttcttggaagaagt
aggaaccggcatTCTTCCACCTGCACCCATGCAT
>DPCR3_CH5_CONTIG_202_p18   227 pairs of NGS reads, 0.45%
AAGCATGAGGCTATAATGATGTCACTGATAAGGTAGGAGAAAGAGCGAGGGATTGAGCGTttgaa
acaatgcggcgggattgctttcttcagatctcccaccgagataaggcggtttctcgcacggcaca
cgctttgtggtgtCGGTTTCCGATTTCTTGGAAGAAGTAGGAACCGGCATTCTTCCACCTGCACC
CATCGAT
>DPCR3_CH5_CONTIG_202_p19   208 pairs of NGS reads, 0.41%
AAGCATGAGGCTATAATGATGTCACTGATAAGGTAGGAGAAAGAGCGAGGGATTGAGCGTttgaa
acaatgcggcgggattgctttcttcagatctcccaccgagataaggcggtttctcgcacggcaca
cgctttgtggtgtCGGTTTCCGATTTCTTGGAAGAAGTAGGAACCGGCATTCTTCCACCTGCACC
CATAGCT
>DPCR3_CH5_CONTIG_127_p20   186 pairs of NGS reads, 0.37%
aagcatgaggctataatgatgtccacaagctgaacagacccatcatgaacctcaaaaagcgtgtc
tgaacaaaagaaaactttagcgagcctgctttcttgcattcttccacctgcacccatagct
>DPCR3_CH5_CONTIG_202_p21   178 pairs of NGS reads, 0.35%
AAGCATGAGGCTATAATGATGTCACTGATAAGGTAGGAGAAAGAGCGAGGGATTGAGCGTttgaa
acaaatgcggcgggattgctttcttcagatctcccaccgagaaaggcggtttctcgcacggcaca
cgctttgtggtgTCGGTTTCCGATTTCTTGGAAGAAGTAGGAACCGGCATTCTTCCACCTGCACC
CATGCAT
>DPCR3_CH5_CONTIG_164_p22   174 pairs of NGS reads, 0.35%
AAGCATGAGGCTATAATGATGTcactgataaggtaggagaaagagcgagggattgagcgtttgaa
acataaggcggtttctcgcacggcacacgctttgtggtgtcggtttccgatttcttggaagaagt
aggaaccggcatTCTTCCACCTGCACCCATCGAT
>DPCR3_CH5_CONTIG_127_p23   171 pairs of NGS reads, 0.34%
aagcatgaggctataatgatgtccacaagctgaacagacccatcatgaacctcaaaaagcgtgtc
tgaacaaaagaaaactttagcgagcctgctttcttgcattcttccacctgcacccatcgat
>DPCR3_CH5_CONTIG_164_p24   158 pairs of NGS reads, 0.31%
AAGCATGAGGCTATAATGATGTcactgataaggtaggagaaagagcgagggattgagcgtttgaa
acataaggcggtttctcgcacggcacacgctttgtggtgtcggtttccgatttcttggaagaagt
aggaaccggcatTCTTCCACCTGCACCCATAGCT
>DPCR3_CH5_CONTIG_161_p25   140 pairs of NGS reads, 0.28%
AAGCATGAGGCTATAATGAtgtcactgataaggtaggagaaagagcgagggattgagcgtttgaa
acaggcggtttctcgcacggcacacgctttgtggtgtcggtttccgatttcttggaagaagtagg
aaccggcattctTCCACCTGCACCCATGCAT
>DPCR3_CH5_CONTIG_192_p26   138 pairs of NGS reads, 0.27%
AAGCATGAGGCTATAATGATGTCCCCCAACATACCAAAACTAGCTGTCACtccaccggtagtagg
agatgtaagaattgatatatagaataacttttacttgattgataatcacataaaaccgaagaaa
ttttagccatttGCATCAAACTTAAACTTCCTTCTTGCATTCTTCCACCTGCACCCATAGCT
>DPCR3_CH5_CONTIG_161_p27   122 pairs of NGS reads, 0.24%
AAGCATGAGGCTATAATGAtgtcactgataaggtaggagaaagagcgagggattgagcgtttgaa
acaggcggtttctcgcacggcacacgctttgtggtgtcggtttccgatttcttggaagaagtagg
aaccggcattctTCCACCTGCACCCATCGAT
>DPCR3_CH5_CONTIG_161_p28   116 pairs of NGS reads, 0.23%
AAGCATGAGGCTATAATGAtgtcactgataaggtaggagaaagagcgagggattgagcgtttgaa
acaggcggtttctcgcacggcacacgctttgtggtgtcggtttccgatttcttggaagaagtagg
aaccggcattctTCCACCTGCACCCATAGCT
>DPCR3_CH5_CONTIG_202_p29   113 pairs of NGS reads, 0.22%
AAGCATGAGGCTATAATGATGTCACTGATAAGGTAGGAGAAAGAGCGAGGGATTGAGCGTttgaa
acaaatgcggcgggattgctttcttcagatctcccaccgagaaaggcggtttctcgcacggcaca
cgctttgtggtgTCGGTTTCCGATTTCTTGGAAGAAGTAGGAACCGGCATTCTTCCACCTGCACC
CATAGCT

>DPCR3_CH5_CONTIG_202_p30   111 pairs of NGS reads, 0.22%
AAGCATGAGGCTATAATGATGTCACTGATAAGGTAGGAGAAAGAGCGAGGGATTGAGCGTttgaa
acaaatgcggcgggattgctttcttcagatctcccaccgagaaaggcggtttctcgcacggcaca
cgctttgtggtgTCGGTTTCCGATTTCTTGGAAGAAGTAGGAACCGGCATTCTTCCACCTGCACC
CATCGAT
>DPCR3_CH5_CONTIG_202_p31   87 pairs of NGS reads, 0.17%
AAGCATGAGGCTATAATGATGTCACTGATAAGGTAGGAGAAAGAGCGAGGGATTGAGCGTttgaa
acatctcggtgggagatctgaagaaagcaatcccgccgcattaaggcggtttctcgcacggcaca
cgctttgtggtgTCGGTTTCCGATTTCTTGGAAGAAGTAGGAACCGGCATTCTTCCACCTGCACC
CATGCAT
>DPCR3_CH5_CONTIG_202_p32   83 pairs of NGS reads, 0.16%
AAGCATGAGGCTATAATGATGTCACTGATAAGGTAGGAGAAAGAGCGAGGGATTGAGCGTttgaa
acatctcggtgggagatctgaagaaagcaatcccgccgcattaaggcggtttctcgcacggcaca
cgctttgtggtgTCGGTTTCCGATTTCTTGGAAGAAGTAGGAACCGGCATTCTTCCACCTGCACC
CATAGCT
>DPCR3_CH5_CONTIG_127_p33   78 pairs of NGS reads, 0.15%
aagcatgaggctataatgatgtccacaagctgaacagacccatcatgaacctcaaaaagcgtgtc
tgaacaaaagaaaactttagcgagcctgctttcttgcattcttccacctgcacccatgcat
>DPCR3_CH5_CONTIG_199_p34   75 pairs of NGS reads, 0.15%
AAGCATGAGGCTATAATGATGTCACTGATAAGGTAGGAGAAAGAGCGAGGGATTGAGcgtttgaa
acaatgcggcgggattgctttcttcagatctcccaccgagaggcggtttctcgcacggcacacgc
tttgtggtgtcgGTTTCCGATTTCTTGGAAGAAGTAGGAACCGGCATTCTTCCACCTGCACCCAT
GCAT
>DPCR3_CH5_CONTIG_164_p35   74 pairs of NGS reads, 0.14%
AAGCATGAGGCTATAATGATGTcactgataaggtaggagaaagagcgagggattgagcgtttgaa
acaaaaggcggtttctcgcacggcacacgctttgtggtgtcggtttccgatttcttggaagaagt
aggaaccggcatTCTTCCACCTGCACCCATCGAT
>DPCR3_CH5_CONTIG_201_p36   66 pairs of NGS reads, 0.13%
AAGCATGAGGCTATAATGATGTCACTGATAAGGTAGGAGAAAGAGCGAGGGATTGAGCGtttgaa
acaaatgcggcgggattgctttcttcagatctcccaccgagaaggcggtttctcgcacggcacac
gctttgtggtgtCGGTTTCCGATTTCTTGGAAGAAGTAGGAACCGGCATTCTTCCACCTGCACCC
ATGCAT
>DPCR3_CH5_CONTIG_202_p37   66 pairs of NGS reads, 0.13%
AAGCATGAGGCTATAATGATGTCACTGATAAGGTAGGAGAAAGAGCGAGGGATTGAGCGTttgaa
acatctcggtgggagatctgaagaaagcaatcccgccgcattaaggcggtttctcgcacggcaca
cgctttgtggtgTCGGTTTCCGATTTCTTGGAAGAAGTAGGAACCGGCATTCTTCCACCTGCACC
CATCGAT
>DPCR3_CH5_CONTIG_199_p38   66 pairs of NGS reads, 0.13%
AAGCATGAGGCTATAATGATGTCACTGATAAGGTAGGAGAAAGAGCGAGGGATTGAGcgtttgaa
acaatgcggcgggattgctttcttcagatctcccaccgagaggcggtttctcgcacggcacacgc
tttgtggtgtcgGTTTCCGATTTCTTGGAAGAAGTAGGAACCGGCATTCTTCCACCTGCACCCAT
CGAT
>DPCR3_CH5_CONTIG_164_p39   65 pairs of NGS reads, 0.13%
AAGCATGAGGCTATAATGATGTcactgataaggtaggagaaagagcgagggattgagcgtttgaa
acaaaaggcggtttctcgcacggcacacgctttgtggtgtcggtttccgatttcttggaagaagt
aggaaccggcatTCTTCCACCTGCACCCATGCAT
>DPCR3_CH5_CONTIG_203_p40   58 pairs of NGS reads, 0.11%
AAGCATGAGGCTATAATGATGTCACTGATAAGGTAGGAGAAAGAGCGAGGGATTGAGCGTTtgaa
acaaatgcggcgggattgctttcttcagatctcccaccgagaaaaggcggtttctcgcacggcac
acgctttgtggtGTCGGTTTCCGATTTCTTGGAAGAAGTAGGAACCGGCATTCTTCCACCTGCAC
CCATGCAT
>DPCR3_CH5_CONTIG_201_p41   57 pairs of NGS reads, 0.11%

AAGCATGAGGCTATAATGATGTCACTGATAAGGTAGGAGAAAGAGCGAGGGATTGAGCGtttgaa
acaaatgcggcgggattgctttcttcagatctcccaccgagaaggcggtttctcgcacggcacac
gctttgtggtgtCGGTTTCCGATTTCTTGGAAGAAGTAGGAACCGGCATTCTTCCACCTGCACCC
ATCGAT
>DPCR3_CH5_CONTIG_199_p42   48 pairs of NGS reads, 0.09%
AAGCATGAGGCTATAATGATGTCACTGATAAGGTAGGAGAAAGAGCGAGGGATTGAGcgtttgaa
acaatgcggcgggattgctttcttcagatctcccaccgagaggcggtttctcgcacggcacacgc
tttgtggtgtcgGTTTCCGATTTCTTGGAAGAAGTAGGAACCGGCATTCTTCCACCTGCACCCAT
AGCT
>DPCR3_CH5_CONTIG_159_p43   45 pairs of NGS reads, 0.09%
AAGCATGAGGCTATAATgatgtcactgataaggtaggagaaagagcgagggattgagcgtttgaa
aggcggtttctcgcacggcacacgctttgtggtgtcggtttccgatttcttggaagaagtaggaa
ccggcattcttcCACCTGCACCCATGCAT
>DPCR3_CH5_CONTIG_203_p44   45 pairs of NGS reads, 0.09%
AAGCATGAGGCTATAATGATGTCACTGATAAGGTAGGAGAAAGAGCGAGGGATTGAGCGTTtgaa
acaaatgcggcgggattgctttcttcagatctcccaccgagataaggcggtttctcgcacggcac
acgctttgtggtGTCGGTTTCCGATTTCTTGGAAGAAGTAGGAACCGGCATTCTTCCACCTGCAC
CCATCGAT
>DPCR3_CH5_CONTIG_201_p45   42 pairs of NGS reads, 0.08%
AAGCATGAGGCTATAATGATGTCACTGATAAGGTAGGAGAAAGAGCGAGGGATTGAGCGtttgaa
acaaatgcggcgggattgctttcttcagatctcccaccgagaaggcggtttctcgcacggcacac
gctttgtggtgtCGGTTTCCGATTTCTTGGAAGAAGTAGGAACCGGCATTCTTCCACCTGCACCC
ATAGCT
>DPCR3_CH5_CONTIG_164_p46   40 pairs of NGS reads, 0.08%
AAGCATGAGGCTATAATGATGTcactgataaggtaggagaaagagcgagggattgagcgtttgaa
acaaaaggcggtttctcgcacggcacacgctttgtggtgtcggtttccgatttcttggaagaagt
aggaaccggcatTCTTCCACCTGCACCCATAGCT
>DPCR3_CH5_CONTIG_159_p47   40 pairs of NGS reads, 0.08%
AAGCATGAGGCTATAATgatgtcactgataaggtaggagaaagagcgagggattgagcgtttgaa
aggcggtttctcgcacggcacacgctttgtggtgtcggtttccgatttcttggaagaagtaggaa
ccggcattcttcCACCTGCACCCATCGAT
>DPCR3_CH5_CONTIG_203_p48   37 pairs of NGS reads, 0.07%
AAGCATGAGGCTATAATGATGTCACTGATAAGGTAGGAGAAAGAGCGAGGGATTGAGCGTTtgaa
acaaatgcggcgggattgctttcttcagatctcccaccgagaaaaggcggtttctcgcacggcac
acgctttgtggtGTCGGTTTCCGATTTCTTGGAAGAAGTAGGAACCGGCATTCTTCCACCTGCAC
CCATCGAT
>DPCR3_CH5_CONTIG_107_p49   36 pairs of NGS reads, 0.07%
aagcatgaggctataatgatgtcactgataaggtaggagaaagagcgagggattgagcgtttgaa
acaatgcggcgggattgcattcttccacctgcacccatcgat
>DPCR3_CH5_CONTIG_200_p50   35 pairs of NGS reads, 0.07%
AAGCATGAGGCTATAATGATGTCACTGATAAGGTAGGAGAAAGAGCGAGGGATTGAGCgtttgaa
acatgcggcgggattgctttcttcagatctcccaccgagaaaggcggtttctcgcacggcacacg
ctttgtggtgtcGGTTTCCGATTTCTTGGAAGAAGTAGGAACCGGCATTCTTCCACCTGCACCCA
TAGCT
>DPCR3_CH5_CONTIG_203_p51   33 pairs of NGS reads, 0.06%
AAGCATGAGGCTATAATGATGTCACTGATAAGGTAGGAGAAAGAGCGAGGGATTGAGCGTTtgaa
acaaatgcggcgggattgctttcttcagatctcccaccgagataaggcggtttctcgcacggcac
acgctttgtggtGTCGGTTTCCGATTTCTTGGAAGAAGTAGGAACCGGCATTCTTCCACCTGCAC
CCATAGCT
>DPCR3_CH5_CONTIG_203_p52   33 pairs of NGS reads, 0.06%
AAGCATGAGGCTATAATGATGTCACTGATAAGGTAGGAGAAAGAGCGAGGGATTGAGCGTTtgaa
acaaatgcggcgggattgctttcttcagatctcccaccgagaaaaggcggtttctcgcacggcac

acgctttgtggtGTCGGTTTCCGATTTCTTGGAAGAAGTAGGAACCGGCATTCTTCCACCTGCAC
CCATAGCT
>DPCR3_CH5_CONTIG_160_p53   30 pairs of NGS reads, 0.06%
AAGCATGAGGCTATAATGatgtcactgataaggtaggagaaagagcgagggattgagcgtttgaa
aaggcggtttctcgcacggcacacgctttgtggtgtcggtttccgatttcttggaagaagtagga
accggcattcttCCACCTGCACCCATAGCT
>DPCR3_CH5_CONTIG_159_p54   28 pairs of NGS reads, 0.05%
AAGCATGAGGCTATAATGatgtcactgataaggtaggagaaagagcgagggattgagcgtttgaa
aggcggtttctcgcacggcacacgctttgtggtgtcggtttccgatttcttggaagaagtaggaa
ccggcattcttcCACCTGCACCCATAGCT
>DPCR3_CH5_CONTIG_229_p55   26 pairs of NGS reads, 0.05%
AAGCATGAGGCTATAATGATGTCAACATGTCATAGTAAGAGAAATCATAGAGAGAGAGAAATGTA
CCAGTTATCTGTGAGATAGATTgtacgggatttgaagacaaggctatagacttatcaggagatgg
aaagacagtattTGATTGGAGCTTGGAACTTAGATAAGAGATCATCTGTTGAAGTTGATGTGGTG
ACATGTCTGCATTCTTCCACCTGCACCCATGCAT
>DPCR3_CH5_CONTIG_134_p56   25 pairs of NGS reads, 0.05%
aagcatgaggctataatgatgtcttagagctaagccaccgttctccatcgccaccaccgcatatt
cctacggacgctccgacatcggagctcgcattcgaaatgtttagcattcttccacctgcacccat
agct
>DPCR3_CH5_CONTIG_202_p57   24 pairs of NGS reads, 0.04%
AAGCATGAGGCTATAATGATGTCACTGATAAGGTAGGAGAAAGAGCGAGGGATTGAGCGTttgaa
acaatgcggcgggattgctttcttcagatctcccaccgagagaaggcggtttctcgcacggcaca
cgctttgtggtgTCGGTTTCCGATTTCTTGGAAGAAGTAGGAACCGGCATTCTTCCACCTGCACC
CATCGAT
>DPCR3_CH5_CONTIG_160_p58   24 pairs of NGS reads, 0.04%
AAGCATGAGGCTATAATGatgtcactgataaggtaggagaaagagcgagggattgagcgtttgaa
aaggcggtttctcgcacggcacacgctttgtggtgtcggtttccgatttcttggaagaagtagga
accggcattcttCCACCTGCACCCATGCAT
>DPCR3_CH5_CONTIG_200_p59   24 pairs of NGS reads, 0.04%
AAGCATGAGGCTATAATGATGTCACTGATAAGGTAGGAGAAAGAGCGAGGGATTGAGCgtttgaa
acatgcggcgggattgctttcttcagatctcccaccgagaaaggcggtttctcgcacggcacacg
ctttgtggtgtcGGTTTCCGATTTCTTGGAAGAAGTAGGAACCGGCATTCTTCCACCTGCACCCA
TCGAT
>DPCR3_CH5_CONTIG_124_p60   24 pairs of NGS reads, 0.04%
aagcatgaggctataatgatgtcactgataaggcggtttctcgcacggcacacgctttgtggtgt
cggtttccgatttcttggaagaagtaggaaccggcattcttccacctgcacccatcgat
>DPCR3_CH5_CONTIG_160_p61   24 pairs of NGS reads, 0.04%
AAGCATGAGGCTATAATGatgtcactgataaggtaggagaaagagcgagggattgagcgtttgaa
aaggcggtttctcgcacggcacacgctttgtggtgtcggtttccgatttcttggaagaagtagga
accggcattcttCCACCTGCACCCATCGAT
>DPCR3_CH5_CONTIG_133_p62   23 pairs of NGS reads, 0.04%
aagcatgaggctataatgatgtcactgataaggtaggagaaagagcgagggattgagcgtttgaa
acgcggcgggattgcttgtttgcttgtttgaagtaggaaccggcattcttccacctgcacccatg
cat
>DPCR3_CH5_CONTIG_202_p63   22 pairs of NGS reads, 0.04%
AAGCATGAGGCTATAATGATGTCACTGATAAGGTAGGAGAAAGAGCGAGGGATTGAGCGTttgaa
acaatgcggcgggattgctttcttcagatctcccaccgagagaaggcggtttctcgcacggcaca
cgctttgtggtgTCGGTTTCCGATTTCTTGGAAGAAGTAGGAACCGGCATTCTTCCACCTGCACC
CATGCAT
>DPCR3_CH5_CONTIG_203_p64   21 pairs of NGS reads, 0.04%
AAGCATGAGGCTATAATGATGTCACTGATAAGGTAGGAGAAAGAGCGAGGGATTGAGCGTTtgaa
acaaatgcggcgggattgctttcttcagatctcccaccgagataaggcggtttctcgcacggcac

acgctttgtggtGTCGGTTTCCGATTTCTTGGAAGAAGTAGGAACCGGCATTCTTCCACCTGCAC
CCATGCAT
>DPCR3_CH5_CONTIG_135_p65   21 pairs of NGS reads, 0.04%
aagcatgaggctataatgatgtcactgataaggtaggagaaaggcggtttctcgcacggcacacg
ctttgtggtgtcggtttccgatttcttggaagaagtaggaaccggcattcttccacctgcaccca
tagct
>DPCR3_CH5_CONTIG_199_p66   21 pairs of NGS reads, 0.04%
AAGCATGAGGCTATAATGATGTCACTGATAAGGTAGGAGAAAGAGCGAGGGATTGAGcgtttgaa
acaatgcggcgggattgctttcttcagatctcccaccgaaaggcggtttctcgcacggcacacgc
tttgtggtgtcgGTTTCCGATTTCTTGGAAGAAGTAGGAACCGGCATTCTTCCACCTGCACCCAT
GCAT
>DPCR3_CH5_CONTIG_200_p67   21 pairs of NGS reads, 0.04%
AAGCATGAGGCTATAATGATGTCACTGATAAGGTAGGAGAAAGAGCGAGGGATTGAGCgtttgaa
acatgcggcgggattgctttcttcagatctcccaccgagaaaggcggtttctcgcacggcacacg
ctttgtggtgtcGGTTTCCGATTTCTTGGAAGAAGTAGGAACCGGCATTCTTCCACCTGCACCCA
TGCAT
>DPCR3_CH5_CONTIG_202_p68   21 pairs of NGS reads, 0.04%
AAGCATGAGGCTATAATGATGTCACTGATAAGGTAGGAGAAAGAGCGAGGGATTGAGCGTttgaa
acaatgcggcgggattgctttcttcagatctcccaccgagacaaggcggtttctcgcacggcaca
cgctttgtggtgTCGGTTTCCGATTTCTTGGAAGAAGTAGGAACCGGCATTCTTCCACCTGCACC
CATAGCT
>DPCR3_CH5_CONTIG_199_p69   20 pairs of NGS reads, 0.04%
AAGCATGAGGCTATAATGATGTCACTGATAAGGTAGGAGAAAGAGCGAGGGATTGAGcgtttgaa
acatgcggcgggattgctttcttcagatctcccaccgagaaggcggtttctcgcacggcacacgc
tttgtggtgtcgGTTTCCGATTTCTTGGAAGAAGTAGGAACCGGCATTCTTCCACCTGCACCCAT
CGAT
